# CDKL3 is a targetable regulator of cell cycle progression in cancers

**DOI:** 10.1172/JCI178428

**Published:** 2024-07-04

**Authors:** Haijiao Zhang, Jiahui Lin, Shaoqin Zheng, Lanjing Ma, Zhongqiu Pang, Hongyi Yin, Chengcheng Meng, Yinuo Wang, Qing Han, Xi Zhang, Zexu Li, Liu Cao, Lijun Liu, Teng Fei, Daming Gao, Liang Yang, Xueqiang Peng, Chen Ding, Shixue Wang, Ren Sheng

**Affiliations:** 1 College of Life and Health Sciences, Northeastern University, Shenyang, China.; 2 Department of Pathology, the Fourth People’s Hospital of Shenyang, Shenyang, China.; 3 College of Sciences, Northeastern University, Shenyang, China.; 4 College of Basic Medical Science, China Medical University, Shenyang, China.; 5 State Key Laboratory of Cell Biology, CAS Center for Excellence in Molecular Cell Science, Shanghai Institute of Biochemistry and Cell Biology, Chinese Academy of Sciences, Shanghai, China.; 6 Department of General Surgery, the Fourth Affiliated Hospital, China Medical University, Shenyang, China.; 7 CAS Key Laboratory of High-Performance Synthetic Rubber and its Composite Materials, Changchun Institute of Applied Chemistry, Chinese Academy of Sciences, Changchun, China.

**Keywords:** Cell biology, Therapeutics, Cancer, Cell cycle, Drug therapy

## Abstract

Cell cycle regulation is largely abnormal in cancers. Molecular understanding and therapeutic targeting of the aberrant cell cycle are essential. Here, we identified that an underappreciated serine/threonine kinase, cyclin-dependent kinase–like 3 (CDKL3), crucially drives rapid cell cycle progression and cell growth in cancers. With regard to mechanism, CDKL3 localizes in the nucleus and associates with specific cyclin to directly phosphorylate retinoblastoma (Rb) for quiescence exit. In parallel, CDKL3 prevents the ubiquitin-proteasomal degradation of cyclin-dependent kinase 4 (CDK4) by direct phosphorylation on T172 to sustain G_1_ phase advancement. The crucial function of CDKL3 in cancers was demonstrated both in vitro and in vivo. We also designed, synthesized, and characterized a first-in-class CDKL3-specific inhibitor, HZ1. HZ1 exhibits greater potency than CDK4/6 inhibitor in pan-cancer treatment by causing cell cycle arrest and overcomes acquired resistance to CDK4/6 inhibitor. In particular, CDKL3 has significant clinical relevance in colon cancer, and the effectiveness of HZ1 was demonstrated by murine and patient-derived cancer models. Collectively, this work presents an integrated paradigm of cancer cell cycle regulation and suggests CDKL3 targeting as a feasible approach in cancer treatment.

## Abstract 2

CDKL3 contributes to aberrant and oncogenic cell cycle, and its inhibition antagonizes cancer growth in multiple models.

## Graphical abstract

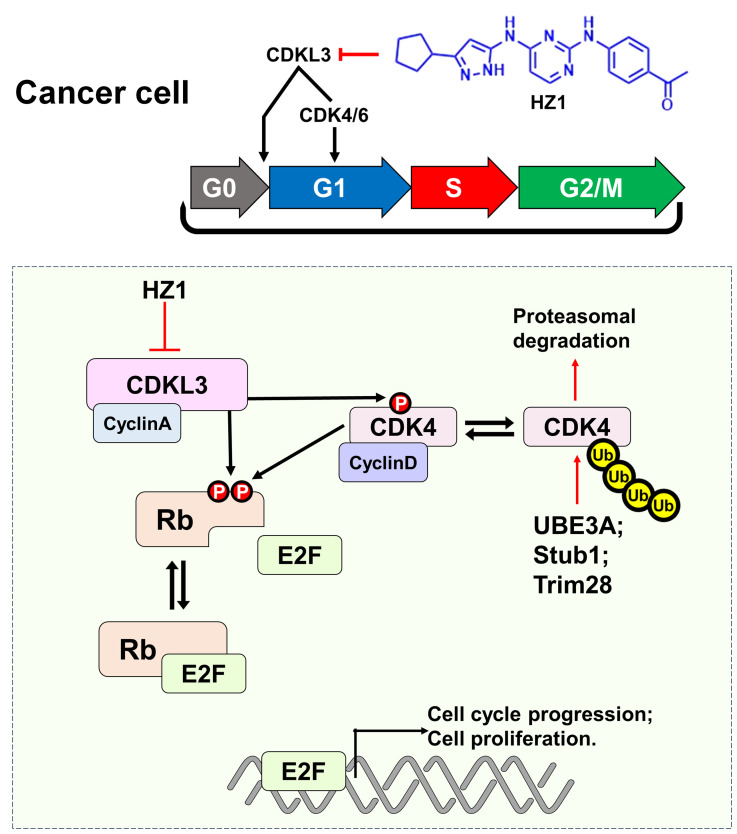

## Introduction

The cell cycle consists of multiple modules and machineries to regulate cell division in a precise manner (1, 2). A complete cell cycle is composed of G_1_, S, G_2_, and M phases sequentially. The progression of the cell cycle is primarily governed by cyclins and cyclin-dependent kinases (CDKs) at various stages (2, 3). For instance, the pair of CDK4/6–cyclin D is known to maintain the G_1_ phase progression (4). At the G_1_/S phase checkpoint, the combination of CDK1/2–cyclin E/A takes over the responsibility for G_1_-to-S transition (4, 5). In both processes, the phosphorylation of retinoblastoma (Rb) protein by CDKs is essentially required (3, 4). The phosphorylated Rb (i.e., on S807/S811) can dissociate with the transcription factor E2F, leading to the expression of multiple cell cycle–related genes that are required in the ensuing phases (6).

Besides active cycling, cells can exit the cell cycle and stay in a resting/quiescent state called G_0_ phase. Cells in this state are generally found nondividing and nongrowing (7). The regulation of G_0_ entry and exit remains obscure. Earlier literature suggested CDK3–cyclin C to support the G_0_-to-G_1_ transition by phosphorylation of Rb (8). However, CDK3 in most laboratory mice harbors the natural loss-of-function mutation with no phenotypic defects (2, 4). Hence, the biological importance of CDK3 in G_0_ phase exit was undermined by this phenomenon. Even though new factors have been reported to regulate this transition in recent years (9, 10), the understanding of this process is still largely lacking (7).

The cell cycle is often dysregulated in cancer (2, 7). The cell cycle entry and exit of normal cells occur on a clear schedule. However, various types of (epi)genetic changes allow the cancer cells to bypass the resting phase (7). Thereby, in cancers, an accelerated and uncontrolled cell cycle is widely seen, which favors infinite mitogenic proliferation (7). Targeting of cell cycle progression has been shown to be effective in cancer therapy (11, 12). Given the central role of CDKs in cell cycle regulation and their enzymatic nature, multiple small-molecule inhibitors of CDKs have been designed and tested in clinical trials or have been clinically approved in cancer treatment (11, 12). However, clinical feedback pointed out the challenge of acquired drug resistance to these inhibitors (13). The discovery of new targets to overcome such resistance is hence of greater necessity and value.

CDKL3 belongs to the cyclin-dependent kinase–like (CDKL) kinase subfamily, which is part of the CMGC Ser/Thr protein kinase superfamily. CDKL kinases share the conserved α-helix on the kinase domain of CDK as the putative cyclin-binding site (14–16). However, whether CDKL could bind cyclins or function via cyclins is unknown. In fact, the CDKL family is overall underexplored in terms of both function and mechanism. CDKL5 has been shown to be related to neurological disorders, whereas CDKL1 was reported to regulate cilia formation (17–19). Though CDKL3 was reported to associate with cancer progression (20–22), the mechanism requires further scrutiny with rigorous evidence.

Here, we report that CDKL3 directly promotes cell cycle progression in cancer. Two parallel regulatory paths exist molecularly. First, CDKL3 couples with cyclin A2 to directly lead to Rb phosphorylation and G_0_-to-G_1_ transition. Ablation of CDKL3 results in cell cycle exit and growth retardation in cancers. Also, CDKL3 can phosphorylate CDK4 on T172 with the assistance of cyclin A2. This leads to the consequence that CDK4 avoids ubiquitin-proteasome–dependent degradation, thereby sustaining G_1_ phase progression. E3 ubiquitin ligases such as Trim28 cause CDK4 ubiquitination in the absence of T172 phosphorylation. Moreover, we rationally designed and characterized the small-molecule inhibitor HZ1 specifically against CDKL3. HZ1 showed strong tumor-suppressing effect with IC_50_ at nanomolar range and the potential to overcome resistance to CDK4/6 inhibitor, and manifested satisfactory tumor clearance in laboratory animals and patient-derived samples. Taken together, we have discovered a series of mechanistic findings on the critical role of CDKL3 in cell cycle regulation and revealed the cyclin-dependent function of the CDKL kinase subfamily. Besides its value for basic biomedical research, this work also presents and proposes an alternative cancer therapeutic approach by targeting of CDKL3-mediated cancer cell cycle progression.

## Results

### CDKL3 loss abrogates cancer cell growth by impeding G_0_-to-G_1_ transition.

**Figure 1:**
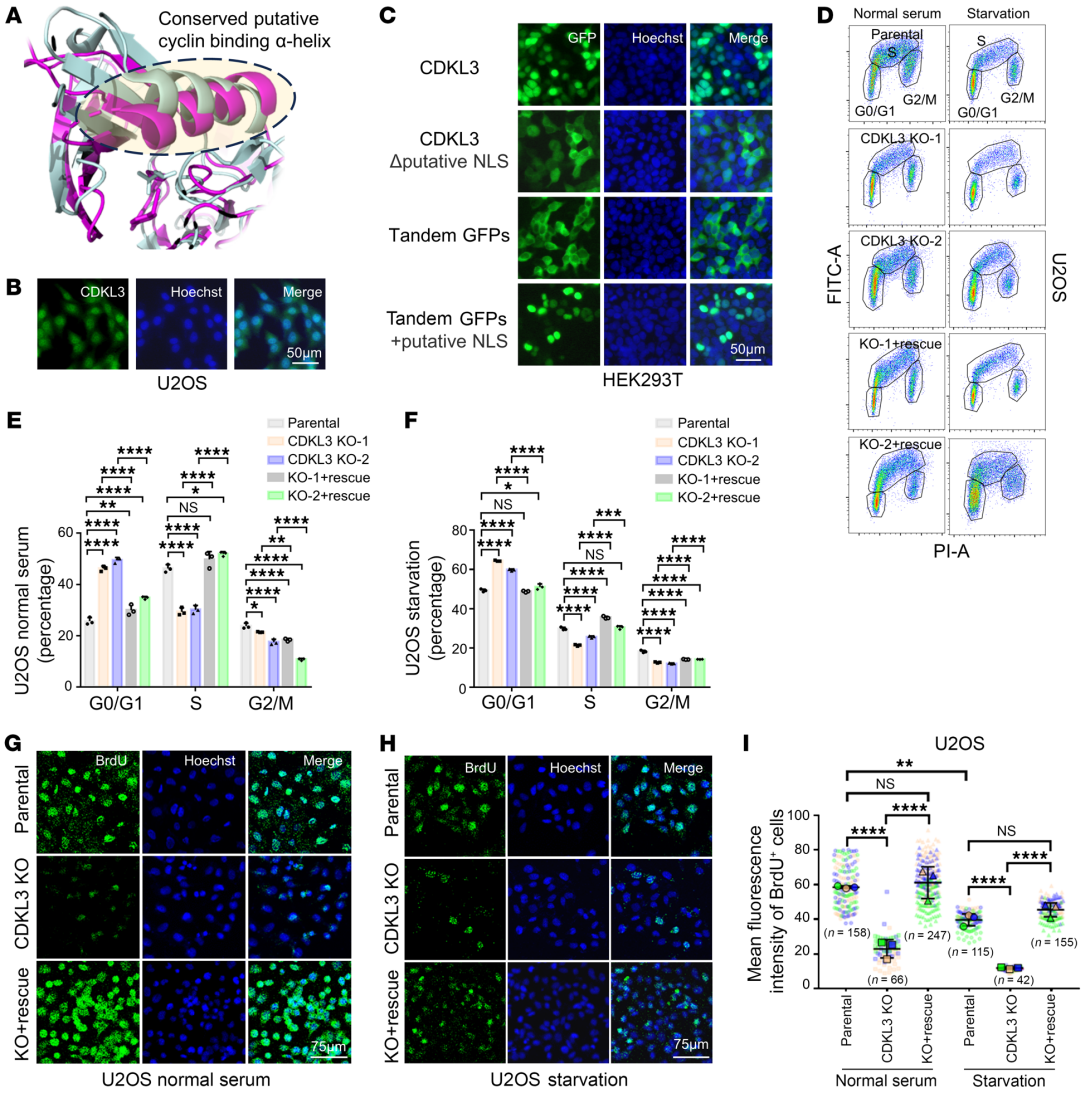
CDKL3 loss causes cell cycle arrest in cancers. (**A**) Overlay of the kinase domains of CDK1 (pale gray; Protein Data Bank ID [PDBID]: 4YC3) and CDKL3 (purple; PDBID: 3ZDU). (**B**) Immunostaining showing the nuclear localization of endogenous CDKL3. (**C**) Fluorescence images of GFP-labeled proteins verifying the NLS function of CDKL3. (**D**) Representative flow cytometry results of U2OS cells with BrdU-FITC/propidium iodide (PI) dual staining. (**E** and **F**) Statistical analysis of the flow cytometry results in **D** showing that CDKL3 ablation significantly increased G_0_/G_1_ percentage. Error bars indicate ± SD, *n* = 3, by 1-way ANOVA. CDKL3 KO-1 was abbreviated as CDKL3 KO in sequel experiments. (**G** and **H**) Representative immunofluorescent images of BrdU in U2OS cells under normal (**G**) or starvation (**H**) conditions. (**I**) SuperPlot analysis of the number of BrdU-positive cells and mean BrdU intensity per cell in **G** and **H**. Error bars indicate ± SD, triplicated, by 1-way ANOVA. The *n* values in the panel represent the total number of cells. All images in the same panel are under the same amplification scales. *P < 0.05; **P < 0.01; ***P < 0.001; ****P < 0.0001.

Similarly to CDK family kinases, CDKL kinases contain the conserved putative cyclin-binding α-helix (14–16, 19) (Figure 1A). CDKL3 also has a potential nucleus localization sequence (NLS) conserved among different species (Supplemental Figure 1A; supplemental material available online with this article; https://doi.org/10.1172/JCI178428DS1). We speculated that CDKL3 might be ready to localize in the nucleus and regulate the cell cycle in a manner reminiscent of CDKs. CDKL3’s subcellular location was inspected to test this assumption. Immunofluorescence has demonstrated that endogenous CDKL3 localizes largely in the nucleus with a small amount in the cytoplasm (Figure 1B and Supplemental Figure 1, B and C). This nuclear localization was largely independent of cell cycle phase (Supplemental Figure 1D). We found that CDKL3 could no longer effectively dwell in the nucleus after internal deletion of the putative NLS (Figure 1C). After the insertion of the NLS of CDKL3, tandem green fluorescent proteins (GFPs), which could only stay in the cytosol, became able to translocate into the nucleus (Figure 1C).

Next, we sought to determine whether CDKL3 affected cell cycle progression. CRISPR/Cas9 system was used to generate multiple CDKL3-knockout (KO) cancer cell lines (Supplemental Figure 1, E–G). According to the flow cytometry experiment of BrdU/propidium iodide (PI) dual staining, KO of CDKL3 significantly raised the fraction of G_0_/G_1_ phase while significantly decreasing the percentage of S phase under both normal and serum starvation conditions (Figure 1, D–F, and Supplemental Figure 1, H–K). The recovery of CDKL3 effectively reversed the situation (Figure 1, D–F). Immunofluorescence intensity of BrdU incorporation per cell and the number of BrdU-positive cells supported the stalled G_0_/G_1_-to-S transition (Figure 1, G–I, and Supplemental Figure 1, L–O) (23).

**Figure 2:**
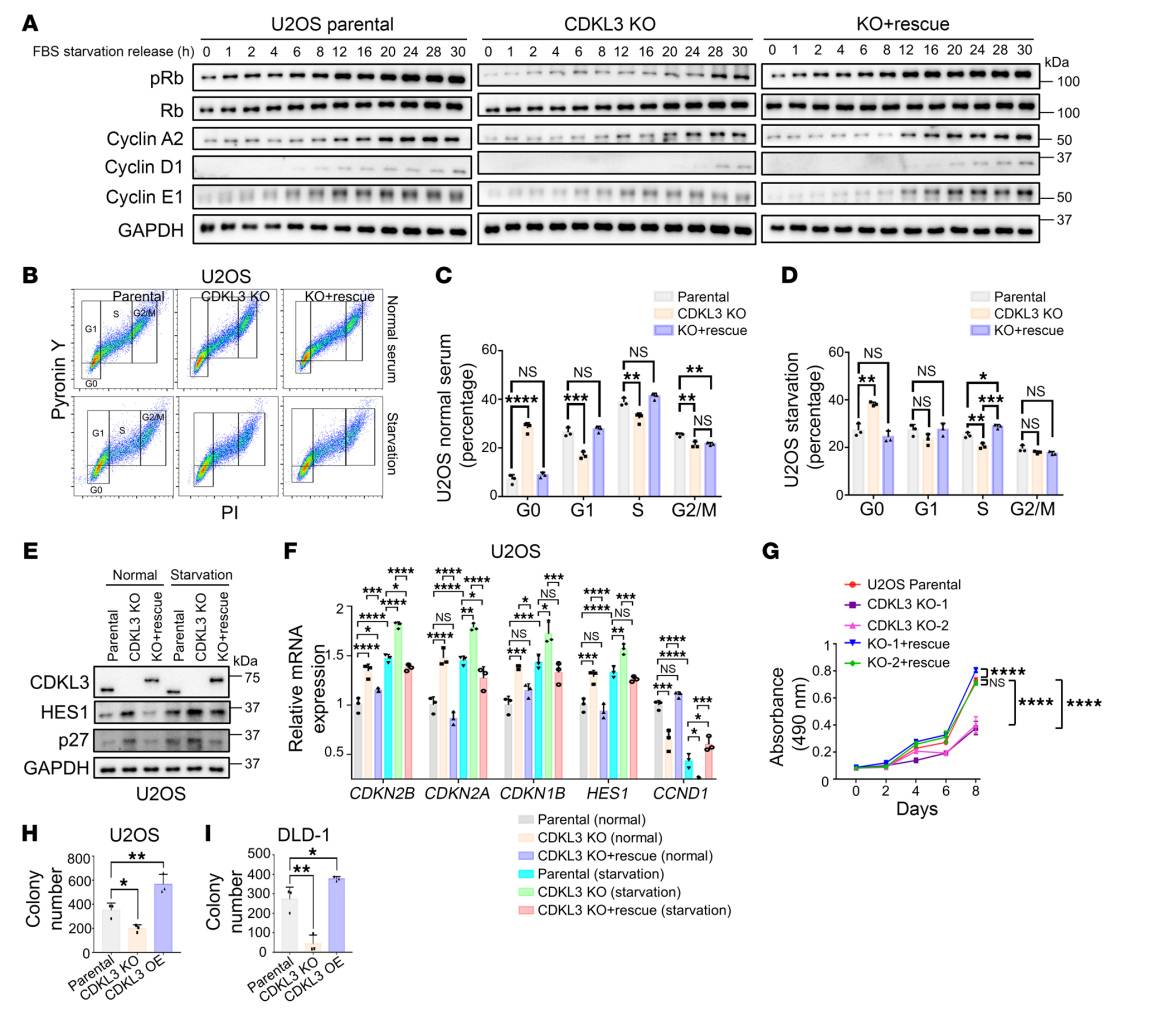
CDKL3 loss retards cancer cell growth by impeding G_0_-to-G_1_ transition. (**A**) Immunoblotting of multiple cell cycle–related proteins after serum starvation and release. pRb: pS807/pS811 Rb. (**B**) Representative flow cytometry results of U2OS cells with pyronin Y/PI dual staining. (**C** and **D**) Statistical analysis of **B** showing that CDKL3 ablation significantly increased G_0_ proportion. Error bars indicate ± SD, *n* = 3, by 1-way ANOVA. (**E**) Immunoblotting showing that CDKL3 ablation or starvation increased the protein levels of the G_0_ markers HES1 and p27 in U2OS cells. (**F**) Quantitative real-time PCR assay showing that the transcription of G_0_ marker genes (*CDKN2B*, *CDKN2A*, *CDKN1B*, and *HES1*) increased in the absence of CDKL3 in U2OS cells. *CCND1* was used as the marker of G_1_ phase. Error bars indicate ± SD, *n* = 3, by 1-way ANOVA. (**G**) MTT assay showing the growth of U2OS cells. Error bars indicate ± SD, *n* = 3, by 2-way ANOVA. (**H** and **I**) Statistical analyses of the colony numbers under 3D culturing of U2OS (**H**) and DLD-1 (**I**) cells. Error bars indicate ± SD, *n* = 3, by 1-way ANOVA. *P < 0.05; **P < 0.01; ***P < 0.001; ****P < 0.0001.

We then had a closer look at G_0_/G_1_ phase. By serum starvation, cells can be synchronized to G_0_ phase, the release of which was able to reveal the process of cell cycle reentry from quiescence (24). In this assay, we visualized that upon CDKL3 KO the appearance of phosphorylated Rb (pRb) and cyclin D1 was substantially delayed and diminished (Figure 2A and Supplemental Figure 1, P–R). Even after release for 30 hours, both pRb and cyclin D1 showed minimal levels when CDKL3 was ablated (Figure 2A and Supplemental Figure 1, P–R). These served as strong markers of cell cycle progression from G_0_/G_1_ toward S phase (2, 3). Later, we further discriminated G_0_ and G_1_ phases by pyronin Y/PI staining (8, 10). The flow cytometry result showed that in multiple cell lines CDKL3 ablation caused a markedly increased proportion of G_0_-phased cells, with strong statistical significance (Figure 2, B–D, and Supplemental Figure 1, S–V). The protein and transcription levels of several G_0_ phase markers also increased after CDKL3 ablation, which supported the flow cytometry data (Figure 2, E and F, and Supplemental Figure 1, W–Z) (25, 26). Moreover, depletion of CDKL3 resulted in evident growth defects in these cancer cell lines, which was in accordance with the cell cycle arrest (Figure 2G and Supplemental Figure 1, AA and AB). Further evidence to support that CDKL3 positively regulated cancer cell growth was provided by the 3D colony formation (Figure 2, H and I, and Supplemental Figure 1, AC–AE). Taken together, these results demonstrate that CDKL3 promotes the cell cycle and cell growth in multiple cancer cells. CDKL3 loss leads to severe delay of G_0_-to-G_1_ and G_1_-to-S transitions and thus prevents cancer cell cycle progression.

### CDKL3 phosphorylates Rb for cell cycle entry when coupling with cyclin A2.

**Figure 3:**
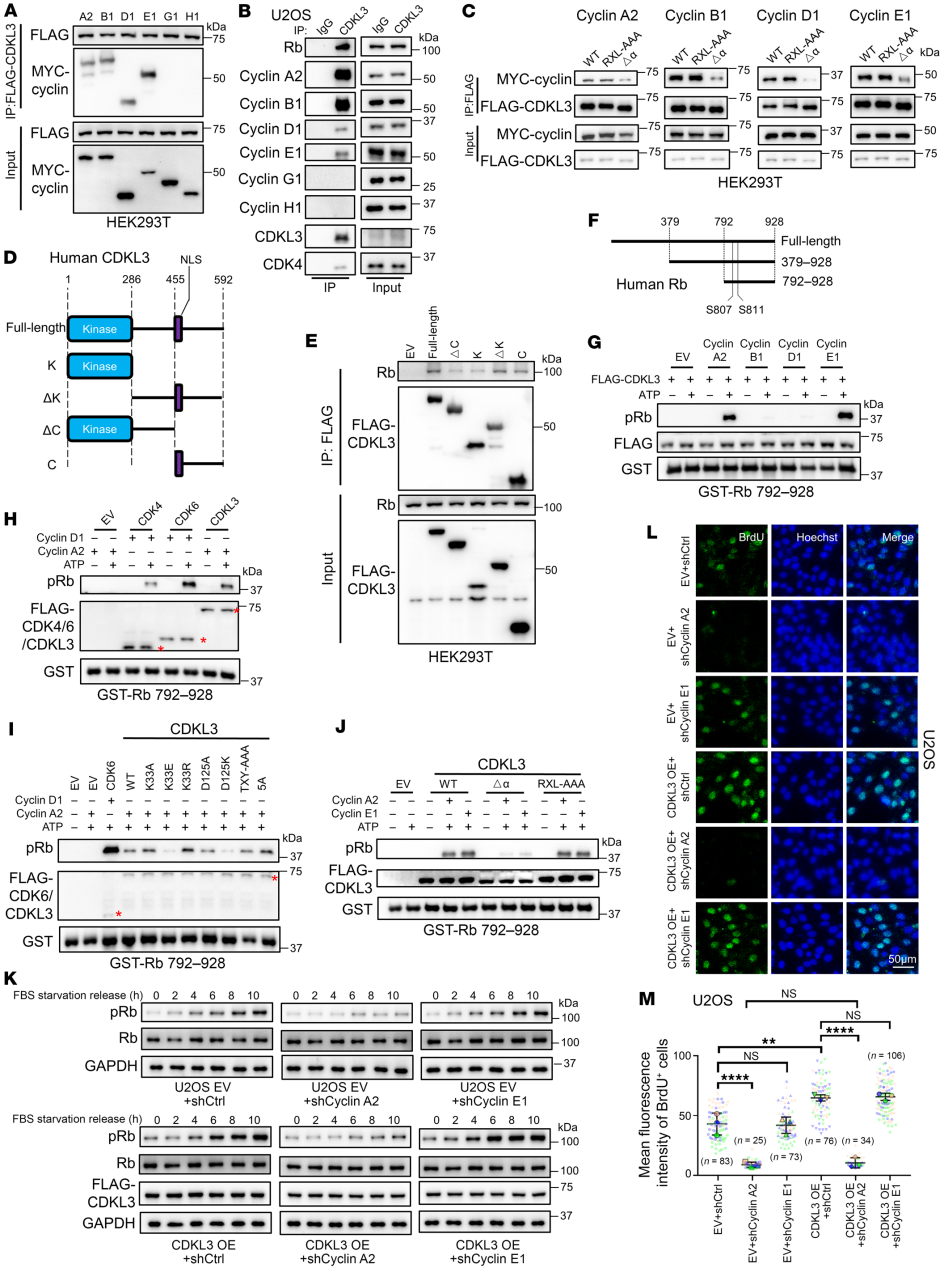
CDKL3 phosphorylates Rb for cell cycle entry when coupling with cyclin A2. (**A**) Co-IP assay showing that exogenous CDKL3 binds to cyclins A2, B1, D1, and E1. (**B**) Co-IP assay showing that endogenous CDKL3 binds to endogenous cyclins A2, B1, D1, and E1, as well as Rb and CDK4. (**C**) Co-IP assay showing that the truncation of the putative cyclin-binding α-helix on the kinase domain of CDKL3 (Δα) abrogated CDKL3 binding to cyclins. RXL-AAA: R148A/T149A/L150A/R510A/K511A/L512A. (**D**) Schematic drawing of the segments of CDKL3. (**E**) Co-IP assay showing that the carboxyl region of CDKL3 binds to Rb. (**F**) Schematic drawing of the commonly used segments of Rb in vitro. S807 and S811 are the major phosphorylation sites. (**G**) In vitro kinase assay showing that CDKL3 phosphorylates Rb in the presence of cyclins A2 and E1. (**H**) In vitro kinase assay showing that CDKL3 phosphorylates Rb to a similar extent compared with CDK4/6. (**I**) In vitro kinase assay showing that CDKL3 K33E and D125K mutants lost the capacity to phosphorylate Rb. TXY-AAA: T158A/D159A/Y160A; 5A: K33A/D125A/T158A/D159A/Y160A. (**J**) In vitro kinase assay showing that CDKL3 Δα mutant lost the capacity of Rb phosphorylation. (**K**) Immunoblotting assay showing that CDKL3 promoted the initial Rb phosphorylation depending on cyclin A2 after serum starvation and release in U2OS cells. (**L**) Representative images of immunofluorescence of BrdU in U2OS cells. (**M**) SuperPlot analysis of the number of BrdU-positive cells and mean BrdU intensity per cell in **L**. Error bars indicate ± SD, triplicated, by 1-way ANOVA. The *n* values in the panel represent the total number of cells. **P < 0.01; ****P < 0.0001. Red asterisks represent the target protein bands.

We further interrogated the mechanism of cell cycle regulation by CDKL3. As CDKL3 was positively correlated with pRb level, we hypothesized Rb as a direct substrate of CDKL3 (Figure 2A and Supplemental Figure 1, Q and R). Because of the presence of the conserved α-helix on the kinase domain, we first questioned whether CDKL3 could interact with any cyclin before further investigation. We observed that several cyclins, including A2, B1, D1, and E1, bound CDKL3 both exogenously and endogenously through coimmunoprecipitation (co-IP) (Figure 3, A and B, and Supplemental Figure 2A). The binding between CDKL3 and cyclin A2 remained unchanged at different cell cycle phases, as the binding of other cyclins to CDKL3 moderately fluctuated (Supplemental Figure 2B). When the conserved α-helix was truncated, binding was greatly impaired (Figure 3C). However, point mutation of RXL motif, another motif suggested to be involved in cyclin binding (27, 28), showed minimal difference in binding compared with wild-type (WT) CDKL3 (Figure 3C). In addition, we demonstrated that endogenous CDKL3 can bind to Rb (Figure 3B). The mapping study pointed out that the carboxyl terminal of CDKL3, which also contained the NLS motif, was primarily involved in Rb binding (Figure 3, D and E). CDK inhibitors such as p21, p27, and p16 were shown to be incapable of interacting with CDKL3 (Supplemental Figure 2C) (2).

To determine whether Rb was the direct substrate of CDKL3, we used an in vitro kinase assay. Two truncations (aa 379–928 and 792–928) containing the phosphorylation sites (S807/S811) were widely accepted in vitro because the size of full-length Rb was too large for bacterial expression (Figure 3F) (6, 29, 30). We constructed both truncations and purified the expressed protein from *E*. *coli*. In the absence of cyclin, it was evident that CDKL3 was unable to phosphorylate Rb (Figure 3G). Introduction of cyclins A2 and E1 into the reaction system could lead to substantial phosphorylation of Rb on S807/S811 by CDKL3, whereas B1 and D1 behaved otherwise (Figure 3G and Supplemental Figure 2D). The CDKL3–cyclin A2 pair was comparable to the traditional CDK4/6–cyclin D1 coupling in Rb phosphorylation strength in vitro (Figure 3H and Supplemental Figure 2E).

We intended to evaluate the functionality of a kinase-dead mutant to confirm the contribution of CDKL3’s kinase activity to Rb phosphorylation. However, the activation site of CDKL3 was unidentified, and the loss-of-function mutant thus remained to be determined. We made a number of point mutations on the putative ATP binding site (K33), the conserved aspartic acid (D125), and the mitogen-activated protein kinase–mimicking (MAPK-mimicking) activation loop (T158/Y160) of CDKL3 (Supplemental Figure 2F). Via in vitro kinase assay, it was discovered that K33E and D125K both led to the loss of CDKL3 kinase activity (Figure 3I). Meanwhile, the conserved α-helix truncation mutant (Δα), which lost cyclin binding capacity, was unable to phosphorylate Rb in vitro in the presence of cyclin (Figure 3J). Neither did the combinatory mutant of K33E/D125K or Δα promote Rb phosphorylation nor restore the cell cycle progression in CDKL3-KO cells (Supplemental Figure 2G). We next determined whether both cyclins A2 and E1 could cooperate with CDKL3 for cell cycle regulation in a cell-based model. In U2OS cells where CDKL3 was ectopically expressed, phosphorylation of Rb was apparently accelerated after serum starvation and release (Figure 3K). This effect, however, could only be neutralized by the depletion of cyclin A2 instead of E1 (Figure 3K and Supplemental Figure 2, H–K). Only when cyclin A2 was depleted did BrdU incorporation, a clear indication of cell cycle progression into S phase, become impaired (Figure 3, L and M) (23). Since CDK2 also requires cyclin A2 to function at G_1_/S checkpoint, we further interrogated the relationship between CDKL3 and CDK2. The in vitro competition assay revealed that cyclin A2 had higher affinity toward CDKL3 than CDK2 (Supplemental Figure 2, L and M). Since the amount of cyclin A2 is low at cell quiescence, we hypothesize that cyclin A2 preferably interacts with CDKL3 for cell cycle entry. When cyclin A2 gradually becomes abundant along with the progression of the cell cycle, CDK2 then will receive sufficient cyclin A2 for activation, hence functioning as the critical factor in G_1_-to-S transition (Supplemental Figure 2N).

Together, these results indicate that CDKL3 serves as the kinase that can directly phosphorylate Rb on the conventional S807/S811 sites. In this event, CDKL3 uses the conserved putative cyclin-binding α-helix in its kinase domain to interact with cyclins, in particular cyclin A2, to enable the phosphorylation of Rb for cell cycle initiation. Following the initiation event, classical CDK-cyclin pairs then take over the responsibility to sustain the hyperphosphorylation of Rb and eventually overcome the G_1_/S phase checkpoint.

### CDKL3 phosphorylates CDK4 on T172 to promote CDK4 stability via K48-linked polyubiquitination prevention.

**Figure 4:**
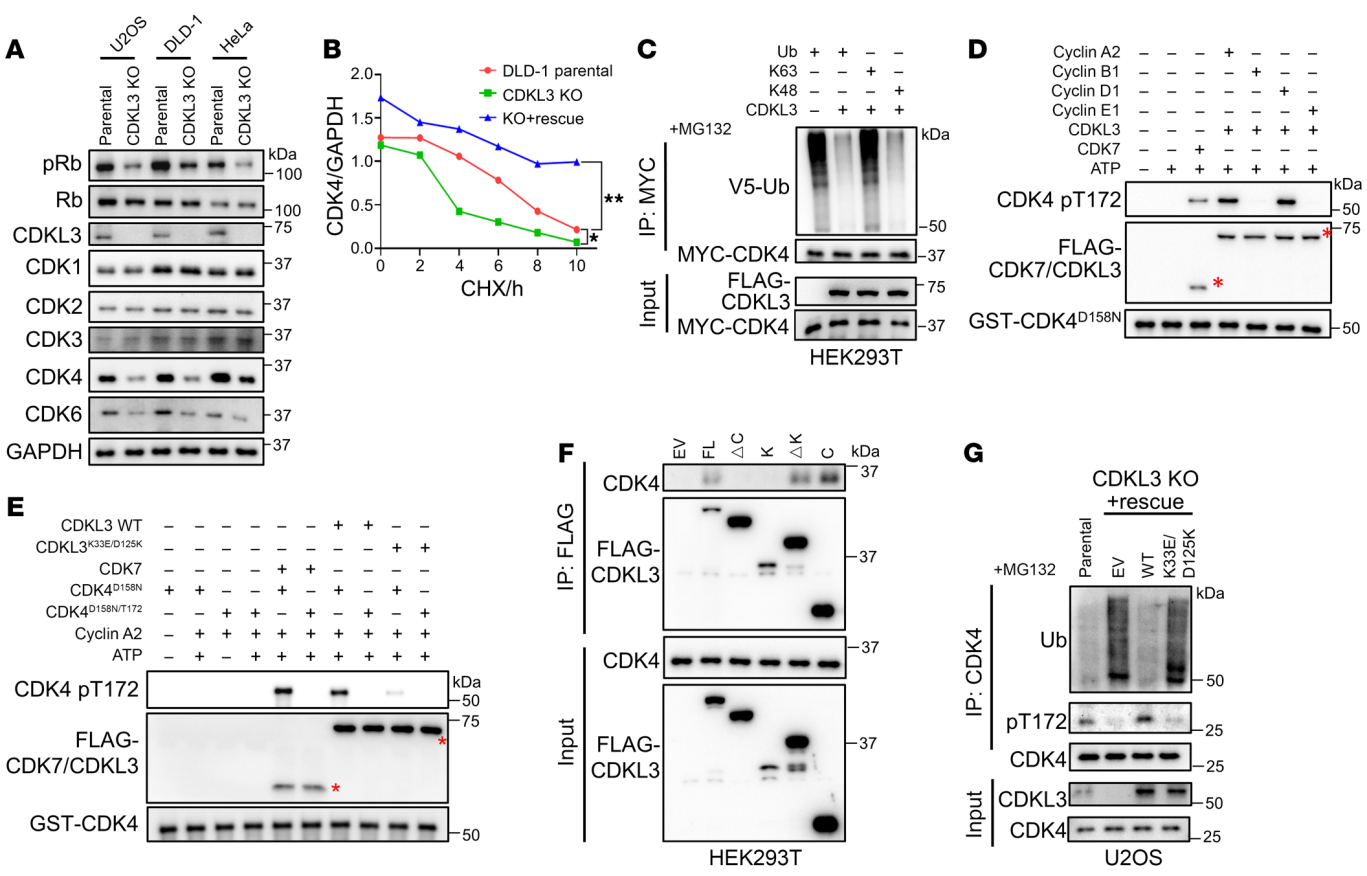
CDKL3 phosphorylates CDK4 on T172 to stabilize CDK4. (**A**) CDKL3 knockout reduced the protein levels of endogenous CDK4 and CDK6 in multiple cell lines. The levels of CDK1/2/3 remained unaffected. (**B**) CHX-blocking assay of endogenous CDK4 protein showing that CDK4 protein stability was positively correlated with CDKL3 level. Error bars indicate ± SD, by 1-way ANOVA. (**C**) Ubiquitination assay of CDK4 showing that the presence of CDKL3 specifically reduced K48-linked polyubiquitination of CDK4. K63: a Ub mutant with all Lys mutated to Arg except Lys63. K48: a Ub mutant with all Lys mutated to Arg except Lys48. (**D** and **E**) In vitro kinase assay showing that CDKL3 phosphorylates CDK4 on T172 in the presence of cyclins A2 and D1 (**D**). K33E/D125K mutant lost the capacity (**E**). The kinase-dead mutant of CDK4 (D158N) was used as substrate to avoid self-phosphorylation. (**F**) Co-IP assay revealing that the carboxyl region of CDKL3 is primarily involved in CDK4 binding. (**G**) Ubiquitination assay of endogenous CDK4 in the presence of WT CDKL3 and K33E/D125K. K33E/D125K lost the capacity to reduce CDK4 ubiquitination. MG132 pretreatment was performed to maintain the same protein level. Red asterisks represent the target protein bands. *P < 0.05; **P < 0.01.

We found an unexpected but intriguing result when we monitored pRb. In all CDKL3-KO cell lines, CDKL3 ablation remarkably reduced the endogenous CDK4 protein levels (Figure 4A). After multiple attempts, we realized the pattern was highly reproducible. To investigate it, we first checked the transcription of CDK4. The quantitative real-time PCR results showed that CDKL3 ablation did not affect CDK4 transcriptionally (Supplemental Figure 3A). Alternatively, a cycloheximide-blocking (CHX-blocking) experiment was used to determine the protein stability of CDK4. It appeared that CDK4 protein stability and CDKL3 had a strong positive correlation, supporting our finding in Figure 4A (Figure 4B and Supplemental Figure 3B). Further demonstration of protein ubiquitination revealed that the presence of CDKL3 markedly reduced the polyubiquitination of CDK4 (Figure 4C). The particular change of polyubiquitination on CDK4 was K48-linked (Figure 4C), which agreed with the well-known biological function of K48-linked polyubiquitination in protein degradation (31).

We used the in vitro kinase assay to determine whether CDK4 was the direct substrate of CDKL3. According to previous research, CDK7 was able to phosphorylate CDK4 on a conserved threonine, T172 (32, 33). This event was reported to promote CDK4 activity and vitally control cell cycle. We therefore interrogated whether CDKL3 could phosphorylate the same site. Enlightened by our earlier finding, we tested the 4 cyclins that could bind CDKL3 by in vitro kinase assay and used CDK7 as the stringent control. The data showed that cyclins A2 and D1 could both assist WT CDKL3 to directly phosphorylate CDK4 on T172 (the kinase-inactive CDK4 was used as the substrate to avoid self-phosphorylation) (34), while the kinase-dead mutant K33E/D125K of CDKL3 failed to accomplish this (Figure 4, D and E). Co-IP assays also showed that the carboxyl region of CDKL3 interacted with CDK4, fulfilling the requirement of potential kinase-substrate relationship (Figure 3B and Figure 4F). These observations were further consolidated by data showing that the kinase-dead mutant of CDKL3 was unable to phosphorylate CDK4 or to reduce CDK4 ubiquitination endogenously (Figure 4G and Supplemental Figure 3C).

**Figure 5:**
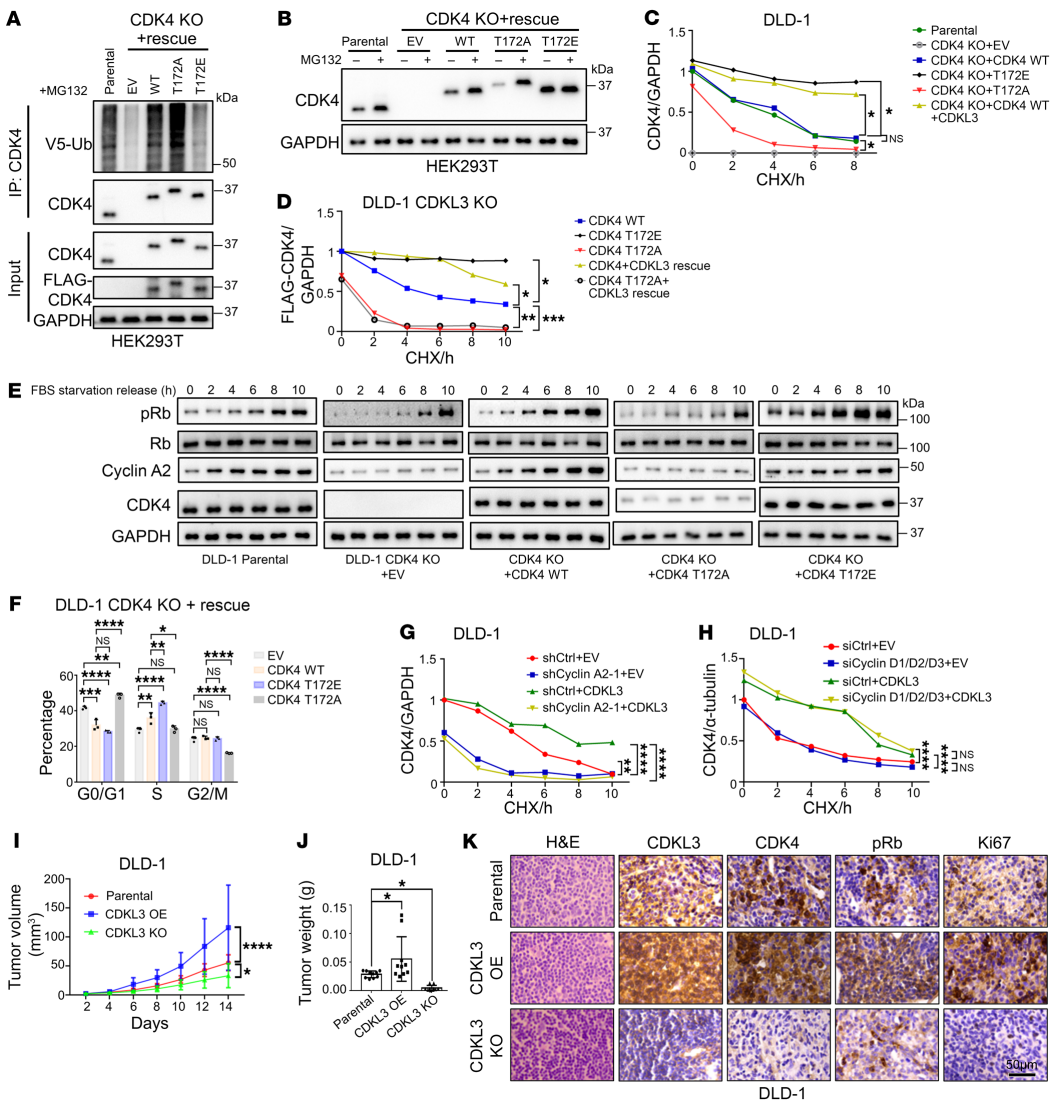
Phosphorylation on T172 prevents the ubiquitin-proteasomal degradation of CDK4 and secures the function of CDK4. (**A**) Ubiquitination assay of CDK4 WT, T172A, and T172E in CDK4-knockout and -rescued HEK293T cells. Ubiquitination was enhanced for T172A and reduced for T172E. MG132 pretreatment was performed to maintain the same protein level. The rescue of CDK4 WT, T172A, and T172E was approximately at the same level as the endogenous CDK4. (**B**) Immunoblotting assay showing that MG132 treatment stabilizes CDK4 T172A in CDK4-knockout and -rescued HEK293T cells. CDK4 T172E was insensitive to MG132 treatment. (**C**) CHX-blocking assay showing that CDK4 T172E had high protein stability whereas T172A had low protein stability in CDK4-knockout and -rescued DLD-1 cells. (**D**) CHX-blocking assay showing that CDK4 T172E remained stable when CDKL3 was ablated. The stability of T172A was unaffected by the presence of CDKL3. (**E**) Immunoblotting assay showing that CDK4 T172A failed to promote a high level of Rb phosphorylation after serum starvation and release in CDK4-knockout and -rescued DLD-1 cells. (**F**) Statistical analysis of the flow cytometry results showing that CDK4 T172A failed to promote G_1_ phase progression. Error bars indicate ± SD, *n* = 3, by 1-way ANOVA. (**G** and **H**) CHX-blocking assay showing that the stabilization of CDK4 by CDKL3 was dependent on cyclin A2 (**G**) instead of cyclin D (**H**). (**I** and **J**) Quantitative analyses of the tumor volume (**I**) and weight (**J**) of tumors formed by the subcutaneously transplanted DLD-1 cells. (**I**) Error bars indicate ± SD, *n* = 10, by 2-way ANOVA. (**J**) Error bars indicate ± SD, *n* = 10, by 1-way ANOVA. (**K**) Representative IHC staining and H&E staining images of subcutaneously transplanted DLD-1 cells. *P < 0.05; **P < 0.01; ***P < 0.001; ****P < 0.0001. All CHX-blocking assays were analyzed by 1-way ANOVA.

According to these clues, we proposed that phosphorylation on T172 of CDK4 could promote its protein stability by avoidance of ubiquitination. By the knockout-and-rescue approach, we observed that the phosphorylation-gain-mimicking mutant T172E showed minimal ubiquitination, whereas the phosphorylation-loss-mimicking mutant T172A was highly polyubiquitinated (Figure 5A and Supplemental Figure 3D). Treatment with the proteasome inhibitor MG132 could substantially stabilize T172A to the same extent as WT and T172E, which verified the involvement of the proteasomal degradation pathway (Figure 5B and Supplemental Figure 3D). From the CHX-blocking assay, it was seen that T172E was markedly stabler than CDK4 WT, whereas T172A only maintained a basal protein level, indicating its low protein stability (Figure 5C and Supplemental Figure 3, E–G). T172E in CDKL3-KO cells appeared as stable as WT CDK4 in CDKL3-rescued cells (Figure 5D and Supplemental Figure 3H). T172A, on the other hand, showed imminent protein degradation in either the presence or the absence of CDKL3 (Figure 5D and Supplemental Figure 3H). In CDK4-KO cells, rescue of T172A failed to recover G_1_ progression as shown by both immunoblotting and flow cytometry, which further illustrated the functional importance of the phosphorylation (Figure 5, E and F, and Supplemental Figure 3I). Regarding the functional relevance of cyclins in the cellular context, depletion of cyclin A2 was incapable of stabilizing CDK4 even in the presence of surplus CDKL3 (Figure 5G and Supplemental Figure 3J). Since the depletion of cyclin D1 was reported to cause strong compensation by cyclins D2 and D3 (35–37), the loss-of-function study of D1 was involuted owing to the technical limitation. We therefore knocked down all 3 to verify the involvement of cyclin D (Supplemental Figure 3K). The CHX-blocking data revealed that depletion of cyclin D did not affect the stability of CDK4 with either endogenous or ectopically expressed CDKL3 (Figure 5H and Supplemental Figure 3L).

Combining the findings on the phosphorylation of Rb and CDK4 by CDKL3, we tested the regulatory role of CDKL3 in cancer cell proliferation in vivo. In the nude mice, we transplanted the parental, CDKL3-KO, and CDKL3-overexpressed (CDKL3-OE) strains of DLD-1 cells. It was convincingly shown that transplanted CDKL3-KO DLD-1 cells hardly developed into tumors (Figure 5, I and J, and Supplemental Figure 3M). Overexpression of CDKL3, on the other hand, considerably enlarged the tumors in vivo compared with the parental group (Figure 5, I and J, and Supplemental Figure 3M). As expected, this effect could be effectively neutralized by the knockout of CDK4 (Supplemental Figure 3, N–P). In the transplanted tumors, we also observed that pRb and CDK4 were markedly reduced when CDKL3 was depleted by both immunohistochemistry (IHC) and immunoblotting (Figure 5K and Supplemental Figure 3Q). Conversely, pRb and CDK4 levels were further elevated under CDKL3-overexpression conditions (Figure 5K and Supplemental Figure 3Q). These data demonstrated that CDKL3 caused cancer cell proliferation by promoting cell cycle progression in vivo, which concurred with the in vitro conclusions. Taken together, we found that CDKL3 directly phosphorylates T172 on CDK4. This process requires cyclin A2 and prevents the ubiquitin-proteasomal degradation of CDK4. Consequently, CDKL3 can assist the cell cycle progression and cell proliferation in cancer via dual paths — CDK4 stabilization and Rb phosphorylation in parallel. CDKL3 therefore can both trigger G_0_-to-G_1_ transition and sustain G_1_ progression.

### Trim28 ubiquitinates CDK4 for protein degradation in the absence of T172 phosphorylation.

**Figure 6:**
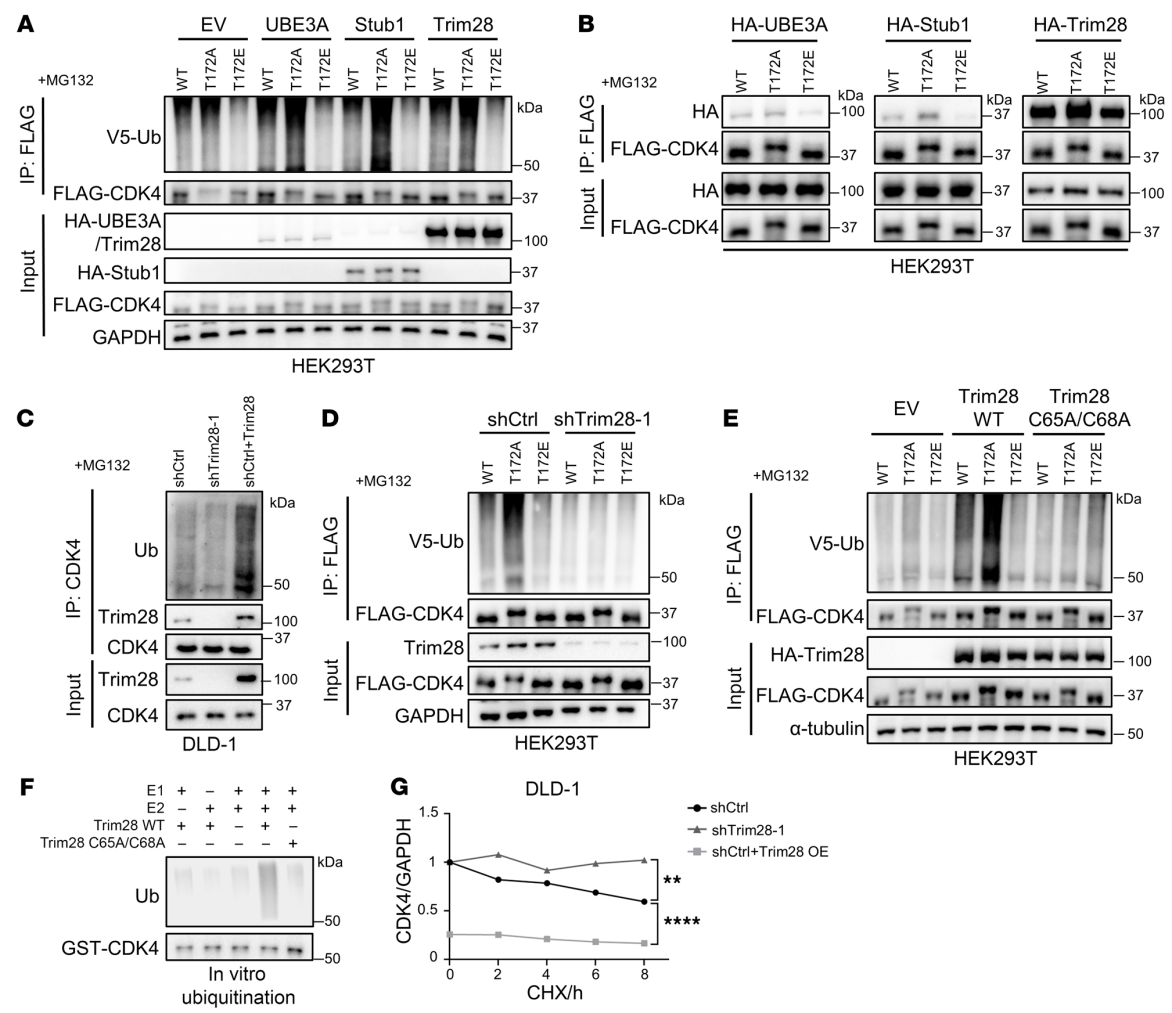
Trim28 ubiquitinates CDK4 for protein degradation in the absence of T172 phosphorylation. (**A**) Ubiquitination assay showing that Trim28, UBE3A, and Stub1 ubiquitinated CDK4 WT and T172A instead of T172E. MG132 pretreatment was performed to maintain the same protein level. (**B**) Co-IP assay of CDK4 and CDK4 mutants with UBE3A, Stub1, and Trim28 showing the stronger binding of T172A and weaker binding of T172E with the E3 ligases. MG132 pretreatment was performed to maintain the same protein level. (**C**) Ubiquitination assay of endogenous CDK4 under Trim28-knockdown and overexpression conditions. Trim28 was positively correlated with the ubiquitination of CDK4. MG132 was added to maintain the same protein level. (**D**) Ubiquitination assay showing that the ubiquitination of CDK4 (WT and mutants) was diminished under Trim28-knockdown condition. MG132 pretreatment was performed to maintain the same protein level. (**E**) Ubiquitination assay showing that the ubiquitination of CDK4 by Trim28 required the enzymatic activity of Trim28. C65A/C68A: Trim28 enzyme-dead mutant. MG132 pretreatment was performed to maintain the same protein level. (**F**) In vitro ubiquitination assay showing that Trim28 required ligase activity to ubiquitinate CDK4. (**G**) Statistical analysis of CHX-blocking assay showing that endogenous CDK4 stability was negatively correlated with Trim28. By 1-way ANOVA. **P < 0.01; ****P < 0.0001.

We next sought the specific protein that senses CDK4 phosphorylation and governs CDK4 ubiquitination. Stub1 and UBE3A, 2 E3 ubiquitin ligases, have been linked to CDK4 ubiquitination in prior research (38, 39). We verified that CDK4 WT and T172A can be ubiquitinated by both ligases, with T172A exhibiting a higher polyubiquitination level than WT CDK4 (Figure 6A). When T172 was mutated to glutamic acid, neither Stub1 nor UBE3A could be operative (Figure 6A). From a mass spectrometry data set on protein-protein interaction (40), we also identified a nuclear-localized and CDK4-binding E3 ligase, Trim28 (41), to be capable of CDK4 ubiquitination (Figure 6A). Like Stub1 and UBE3A, Trim28 failed to ubiquitinate CDK4 T172E while being active on CDK4 WT and T172A (Figure 6A). The co-IP assay showed that T172E had poorer binding capacity with all 3 E3 ligases (Figure 6B), suggesting that the phosphorylation of T172 might not be favored by these E3 ligases in terms of substrate recognition.

Trim28 was further characterized. Depletion of Trim28 attenuated the overall polyubiquitination of CDK4 (Figure 6, C and D, and Supplemental Figure 4, A–D). Additionally, Trim28 enzyme-dead mutant (42) (C65A/C68A) failed to ubiquitinate CDK4, supporting the requirement for the ligase activity (Figure 6E). The direct enzyme-substrate relationship between Trim28 and CDK4 was further validated by in vitro ubiquitination assay, in which CDK4 was purified from bacteria with zero basal ubiquitination background (Figure 6F). Endogenously, Trim28 depletion reduced CDK4 ubiquitination and increased CDK4 protein stability, whereas overexpressed Trim28 further diminished CDK4 stability (Figure 6G and Supplemental Figure 4E). With regard to function, perturbation of Trim28 (overexpression or depletion), however, did not affect cancer cell growth or cell cycle (Supplemental Figure 4, F–H). We hypothesized that this was due to the redundancy or compensation of CDK6 or other E3 ligases. Also, by literature search, we realized that Trim28 had several cell cycle–related proteins as substrates (such as Rb and cyclin A2) (41, 43, 44). Thus, Trim28 could exert complicated influence on the cell cycle, which is likely context dependent. Nevertheless, this mystery does not affect the claim of CDK4 being a direct substrate of Trim28. CDK4 ubiquitination (and ensuing degradation) is sensitive to the phosphorylation on T172, but unspecific to a particular E3 ubiquitin ligase.

### CDK inhibitors do not affect CDKL3 kinase activity.

**Figure 7:**
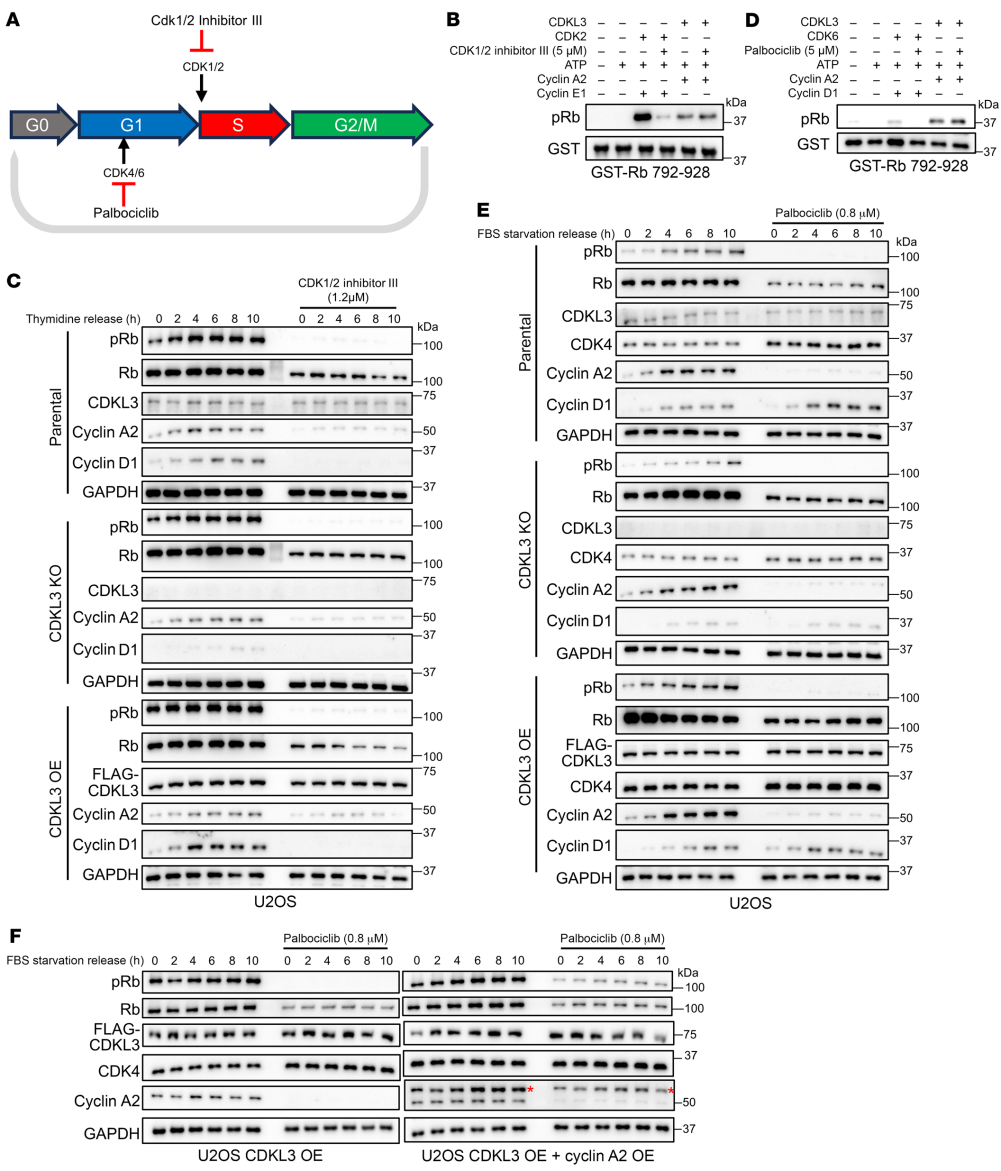
CDK inhibitors do not affect CDKL3 kinase activity. (**A**) Schematic drawing of cell cycle inhibition by CDK4/6 inhibitor and CDK1/2 inhibitor. Cdk1/2 inhibitor III: CDK1/2 inhibitor. Palbociclib: CDK4/6 inhibitor. (**B**) In vitro kinase assay showing that CDK2 inhibitor had no effect on the kinase activity of CDKL3 when phosphorylating Rb. (**C**) Immunoblotting of multiple cell cycle–related proteins with double thymidine blocking and release and CDK1/2 inhibitor III treatment (1.2 μM) under parental, CDKL3 KO, or overexpression conditions in U2OS cells. CDKL3 cannot affect G_1_-to-S transition. (**D**) In vitro kinase assay showing that CDK4/6 inhibitor had no effect on the kinase activity of CDKL3 when phosphorylating Rb. (**E**) Immunoblotting of multiple cell cycle–related proteins after serum starvation and release and palbociclib treatment (0.8 μM) under parental, CDKL3 KO, or overexpression conditions in U2OS cells. CDKL3 cannot compensate the inhibition of CDK4/6. (**F**) Immunoblotting assay showing that CDKL3 can partially maintain Rb phosphorylation and G_1_ progression upon overexpression of cyclin A2 but cannot fully compensate the function of CDK4/6 after serum starvation and release in U2OS cells. Red asterisks represent the overexpressed Myc-tagged cyclin A2.

We next sought to find a small molecule to block CDKL3 in order for cell cycle arrest. To date, CDK4/6 inhibitors and CDK1/2 inhibitors are widely used in clinical therapies or under clinical trials because of their pan-cancer killing effects (Figure 7A) (11, 12). We thus tested the possible inhibitory effect of these inhibitors on CDKL3. From the in vitro kinase assay, we observed that CDK2 inhibitor (Cdk1/2 inhibitor III) (45) could effectively inhibit CDK2 but exerted no effect on CDKL3 when phosphorylating Rb (Figure 7B). Since CDK1/2 primarily governs the entry of S phase from G_1_, we used double thymidine blocking to synchronize cells right at the G_1_/S checkpoint to explore this process (46). The results supported our earlier discovery that CDKL3 primarily functioned in G_0_-to-G_1_ transition and G_1_ phase. CDKL3 ablation or overexpression had minimal impact on S phase entry of the cancer cells (Figure 7C and Supplemental Figure 5, A–C). In all circumstances, the treatment of CDK2 inhibitor led to the complete disappearance of pRb and cyclin D1 (Figure 7C and Supplemental Figure 5, A–C). These data validate that CDKL3 cannot replace the function of CDK1/2 at G_1_-to-S transition.

Meanwhile, we tested CDK4/6 inhibitor (47) in vitro. The data demonstrated that the CDK4/6 inhibitor palbociclib could only effectively target CDK4 rather than CDKL3 for inhibition (Figure 7D). To observe G_0_-to-G_1_ transition and the progression of G_1_ phase, serum starvation was used to synchronize cells at G_0_. In the cancer cells, the accumulation of pRb halted when CDKL3 was ablated and accelerated with ectopic CDKL3 (Figure 7E and Supplemental Figure 5, D–F). However, in all conditions, treatment with CDK4/6 inhibitor resulted in the elimination of pRb and cyclin A2 (Figure 7E and Supplemental Figure 5, D–F). This came as a surprise because, according to our hypothesis, CDKL3 functions prior to CDK4/6; as a result, Rb phosphorylation by CDKL3 should appear shortly after serum release in the presence of CDK4/6 inhibitor but would be unlikely to augment further in an extended period. From literature reading and the data analysis, we also noticed a well-documented side effect that cyclin A2 can be ameliorated by CDK4/6 inhibitor treatment (48, 49). It was very likely that CDK4/6 inhibition disabled E2F for expression of *CCNA2* (encoding cyclin A2). Though the kinase activity of CDKL3 was not affected by palbociclib, it required cyclin A2 to function (Figure 3K). Thus, why Rb remained unphosphorylated could be explained (Figure 7E and Supplemental Figure 5, D–F). When we ectopically expressed cyclin A2, CDKL3 indeed could partially sustain Rb phosphorylation and G_1_ progression (Figure 7F). But CDKL3 could hardly compensate in full the function of CDK4/6 in sustaining the substantial Rb phosphorylation required for cell cycle advancement.

Together, these results suggest that neither CDK1/2 nor CDK4/6 inhibitor affects CDKL3 kinase activity. CDKL3 cannot replace the function of CDK1/2 in G_1_/S transition or that of CDK4/6 in maintaining adequate Rb phosphorylation in G_1_. The design and characterization of a first-in-class CDKL3 inhibitor is necessary, which can potentially provide an alternative therapeutic path in cancer treatment by causing cell cycle arrest.

### Design and characterization of CDKL3 inhibitor.

**Figure 8:**
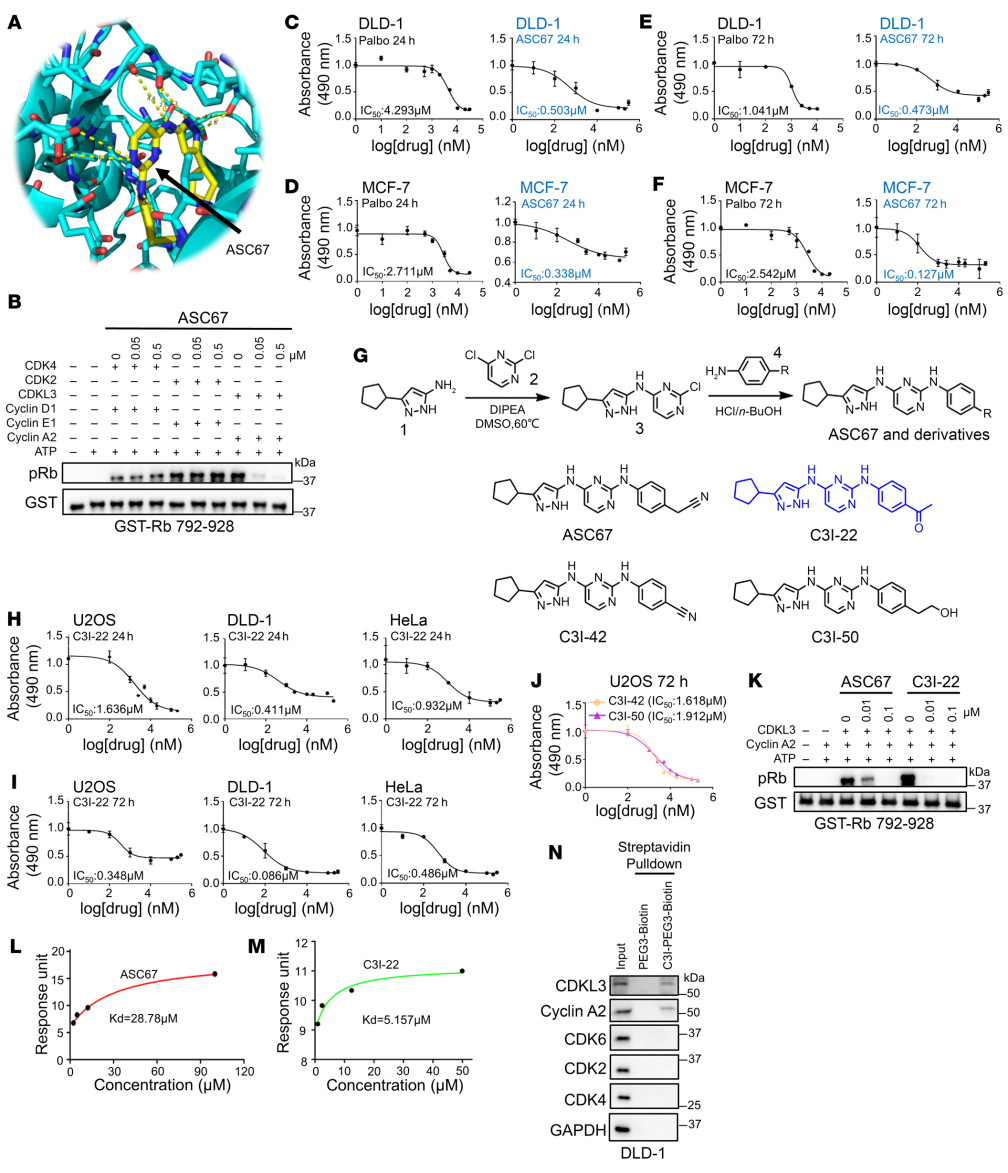
Design and characterization of CDKL3 inhibitor. (**A**) Structure of the kinase domain of CDKL3 with ASC67. Yellow dashes represent the potential hydrogen bonds between CDKL3 and ASC67. PDBID: 3ZDU. (**B**) In vitro kinase assay showing that ASC67 specifically inhibited the kinase activity of CDKL3 to phosphorylate Rb dose-dependently. (**C**–**F**) Tumor-suppressing effects of palbociclib and ASC67 treatments under different conditions. MCF-7 is an estrogen-responsive breast cancer cell line that is used as a direct comparison. (**G**) The synthesis route and chemical structures of ASC67, C3I-22, C3I-42, and C3I-50. ASC67 and derivatives were synthesized via 2 steps. Compounds 1 and 2 reacted in the presence of DIPEA to obtain intermediate [3]. Then different aniline derivatives [4] could be substituted for intermediate [3] to generate ASC67 and its derivatives. (**H**–**J**) Tumor-suppressing effects of C3I-22 in different cancer cells at 24 hours (**H**) and 72 hours (**I**) and other derivatives at 72 hours (**J**). (**K**) In vitro kinase assay showing that C3I-22 has stronger CDKL3-inhibitory function than ASC67. (**L** and **M**) Dissociation constants of CDKL3/ASC67 (**L**) and CDKL3/C3I-22 (**M**) binding acquired from surface plasmon resonance. (**N**) Cellular pull-down assay showing that C3I inhibitor has great selectivity in cells. All IC_50_ analysis was triplicated.

On the basis of a previous publication, a potential CDKL3 inhibitor candidate caught our attention. From a chemical screening study, it was discovered that a compound named ASC67 could bind to the catalytic pocket of the CDKL3 kinase domain with satisfactory selectivity over other related kinases, yet the functional demonstration was absent (Figure 8A) (19). Thus, we intended to generate an efficient CDKL3 inhibitor based on this chemical backbone for cell cycle arrest. As the first step, the original compound (ASC67) was synthesized, and the performance was evaluated (50). The addition of ASC67 could gradient-dependently diminish the phosphorylation of Rb by CDKL3 while not influencing CDK4 or CDK2 in vitro (Figure 8B). The effectiveness of this chemical was then evaluated using a variety of cancer cell lines, including estrogen-responsive (ER^+^) breast cancer, for which palbociclib is administrated clinically (51). All cell lines that were treated with ASC67 for 24 and 72 hours showed significant tumor-suppressing effects, with IC_50_ values with a median of 500 nM (Figure 8, C–F, and Supplemental Figure 6, A–F). As a direct comparison, the clinically approved CDK4/6 inhibitor had IC_50_ values ranging from 1 to 5 μM in different cell lines (Figure 8, C–F, and Supplemental Figure 6, A–F) (47, 52–55). This information made it evident that ASC67 holds considerable promise in cancer treatment and can be used as a backbone compound for further optimization.

We further conducted rational design to enhance the affinity and effectiveness of CDKL3 inhibitor based on ASC67. From the structural analysis, we realized that the central pyrimidine group of ASC67 forms multiple key hydrogen bonds with CDKL3, which was not recommended to be replaced (Figure 8A). And the cyclopentane group forms π-π interaction with the side chain of Phe80 to stabilize the hydrophobic pocket residence (Figure 8A). We hence focused on the cyan group of this molecule and proposed several polar functional groups for substitution (Figure 8G). All molecules were produced by organic synthesis. After tests, a few replacements showed lessened CDKL3 inhibitory impact. Fortunately, C3I-22, one of the compounds, had a much lower IC_50_ value for clearing cancer cells compared with the backbone molecule (Figure 8, H–J). From these data, we could see that C3I-22 had increased the effectiveness 3- to 4-fold compared with ASC67 (Figure 8, H–J, and Supplemental Figure 6, A and D). Consistently, C3I-22 also showed a lower threshold for substrate phosphorylation in vitro (Figure 8K). Biophysical detection (by surface plasmon resonance assay) clearly indicated that the binding affinity between CDKL3 and C3I-22 was 5-fold higher than that between CDKL3 and ASC67 (Figure 8, L and M, and Supplemental Figure 6, G and H). To test the cellular selectivity of the ASC67 derivatives, we designed and synthesized orthogonal polyethylene glycol–linked (PEG-linked) biotinylated derivatives (C3I-PEG3-biotin) for cellular content pull-down assay by streptavidin resins (Supplemental Figure 6I). After treatment of the cells, the protein bound to C3I-PEG3-biotin was harvested after cell lysis and dialysis. It showed that cellular CDKL3 could be effectively captured by C3I-PEG3-biotin instead of CDK2 and CDK4/6 (Figure 8N). Hence, we argue that the selectivity of ASC67 derivatives is adequate. We further genetically modulated CDKL3, CDK4, and Rb in the cells to examine whether C3I-22 restrained the cell cycle specifically through CDKL3-mediated events. As expected, cells with ectopic expression of CDKL3 and CDK4 both required a greater amount of C3I-22 to suppress cell growth. Knockout of CDK4 or increased amount of Rb led to reduced IC_50_ value; depletion of CDKL3 or Rb made the cells insensitive to C3I-22 treatment (Table 1; Supplemental Figure 1, E and F; Supplemental Figure 3E; and Supplemental Figure 6, J–S). These results thus further verified the specificity of C3I-22 and our mechanistic model. Together, our findings demonstrated the feasibility of CDKL3 inhibitors and the improved potency of C3I-22 with satisfactory specificity.

### C3I-22 (HZ1) antagonizes cancer growth via cell cycle arrest in multiple models.

**Figure 9:**
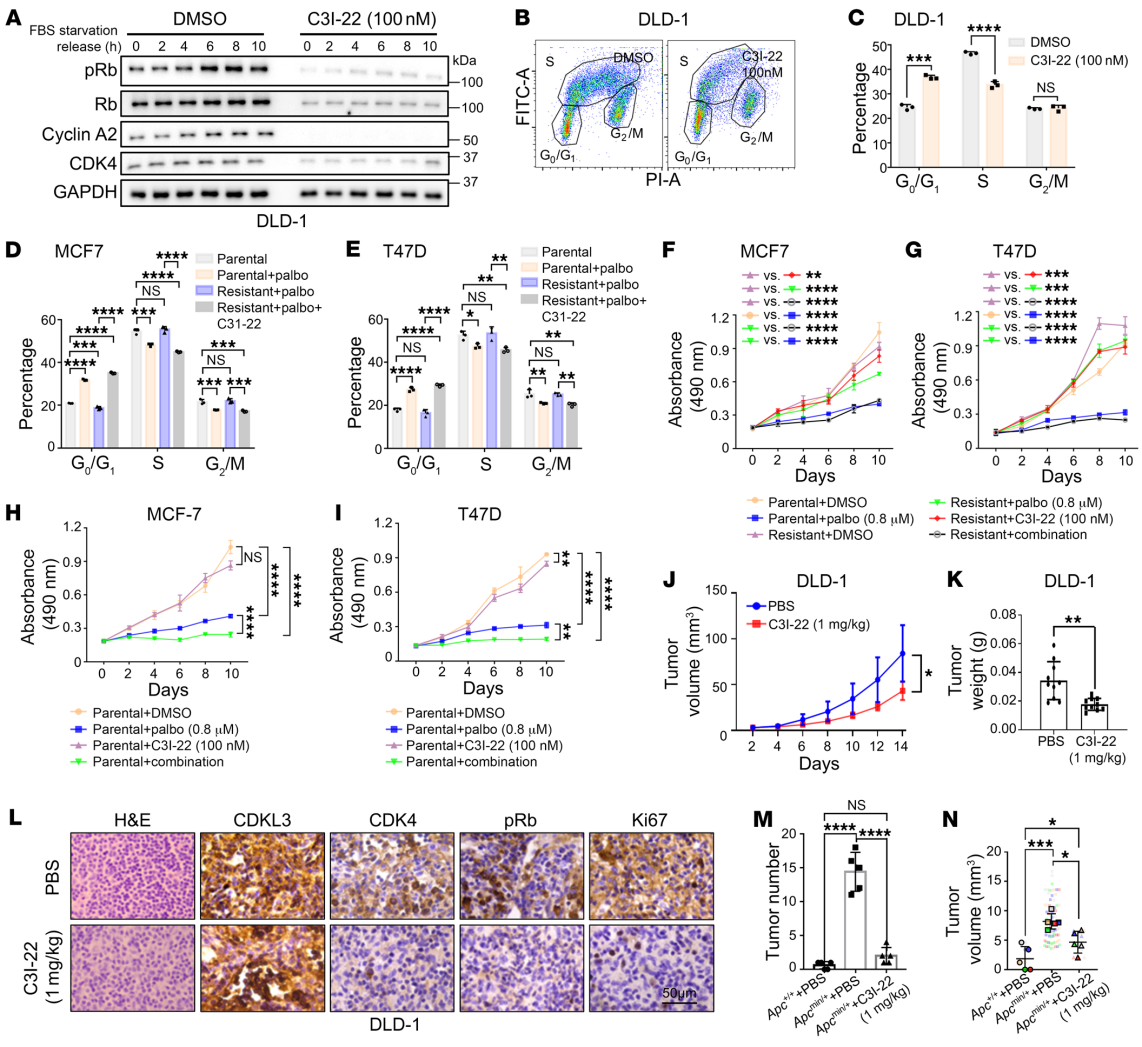
C3I-22 (HZ1) antagonizes cancer growth via cell cycle arrest. (**A**) Immunoblotting showing C3I-22 treatment reduced pRb and CDK4 levels after serum starvation and release in DLD-1. (**B** and **C**) Flow cytometry results of BrdU/PI dual staining showing C3I-22 treatment caused cell cycle arrest in DLD-1. (**B**) Representative result. (**C**) Statistical analysis. Error bars indicate ± SD, *n* = 3, by 2-tailed Student’s *t* test. (**D** and **E**) Flow cytometry of BrdU/PI dual staining showing that combinatorial palbociclib and C3I-22 treatment effectively caused cell cycle arrest at G_0_/G_1_ phase in resistant MCF-7 (**D**) and T47D (**E**). Error bars indicate ± SD, *n* = 3, by 1-way ANOVA. (**F** and **G**) MTT assay showing C3I-22 treatment abolished the acquired palbociclib resistance in MCF-7 (**F**) and T47D (**G**). Error bars mean ± SD, *n* = 3, by 2-way ANOVA. (**H** and **I**) MTT assay showing C3I-22 treatment sensitized MCF-7 (**H**) and T47D (**I**) to palbociclib. Error bars indicate ± SD, *n* = 3, by 2-way ANOVA. (**J** and **K**) Quantitative analyses of tumor volume (**J**) and weight (**K**) of subcutaneously transplanted DLD-1 under different treatments. (**J**) Error bars indicate ± SD, *n* = 10, by 2-way ANOVA. (**K**) Error bars indicate ± SD, *n* = 10, by 2-tailed Student’s *t* test. (**L**) Representative IHC and H&E staining of subcutaneously transplanted DLD-1. (**M** and **N**) Tumor number (**M**) and volume (**N**) in intestines of *Apc^min/+^* mice under different treatments. (**M**) Error bars indicate ± SD, *n* = 5, by 1-way ANOVA. (**N**) Error bars indicate ± SD, 5 mice per group, by 1-way ANOVA; *Apc^+/+^*+PBS, *n* = 3; *Apc^min/+^*+PBS, *n* = 71; *Apc^min/+^*+C3I-22, *n* = 10. Palbo, palbociclib. Images in the same panel are under the same amplification scales. *P < 0.05; **P < 0.01; ***P < 0.001; ****P < 0.0001.

Besides the cell growth suppression effect, we further examined C3I-22 molecularly. In the cellular context, C3I-22 treatment reduced the levels of pRb and CDK4 (Figure 9A and Supplemental Figure 7A) and caused the failure of rapid cell cycle progression in cancer cells as shown by flow cytometry (Figure 9, B and C, and Supplemental Figure 7, B and C). Besides, the treatment of C3I-22 could overcome the acquired drug resistance to CDK4/6 inhibitor in ER^+^ breast cancer (Table 2 and Supplemental Figure 7D). The cell cycle of the resistant cell lines showed high sensitivity to C3I-22 treatment, thus further promising therapeutic value of C3I-22 (Figure 9, D and E, and Supplemental Figure 7, E and F). Further interrogation revealed that C3I-22 could not only remove the existing resistance to CDK4/6 inhibitor but also sensitize the cells to CDK4/6 inhibitor (Figure 9, F–I) (56). We reason that C3I-22 could act on a different phase (G_0_-to-G_1_ transition) from CDK4/6 inhibitor and meanwhile suppress the elevated CDK4/6 protein level, which was often the cause of acquired resistance (13, 57). On a different note, improved potency was not seen in the compounds that were further developed based on C3I-22 (Supplemental Figure 7, G and H). Thus, we focused on C3I-22 for functional tests in this study.

We examined C3I-22’s ability to inhibit tumors in vivo. We first tested the therapeutic effect of C3I-22 in the xenograft model. Administration of a very low dosage of C3I-22 could already effectively clear the tumor burden of the transplanted DLD-1 cells and mitigate the levels of pRb and CDK4, and within the period of treatment the mice displayed no severe liver and kidney toxicity (Figure 9, J–L, and Supplemental Figure 7, I–K). Another mouse model was also used to demonstrate the efficacy of this inhibitor. In *Apc^min/+^* mice, a well-documented model that forms spontaneous colorectal cancer (58, 59), treatment of C3I-22 significantly reduced tumor number and volume (Figure 9, M and N, and Supplemental Figure 7, L and M). Histological data also showed that the intestinal architecture was restored and the proliferation marker Ki67 was diminished after treatment (Supplemental Figure 7N). Thus, we eventually renamed C3I-22 as HZ1 for further characterization. Together, in sum, we have rationally designed and developed a first-in-class CDKL3 inhibitor, HZ1. HZ1 showed substantial promise as a therapy for cancer, since it effectively blocks CDKL3-mediated cell cycle progression both in vitro and in vivo.

### HZ1 has strong clinical implications in colon cancer treatment.

**Figure 10:**
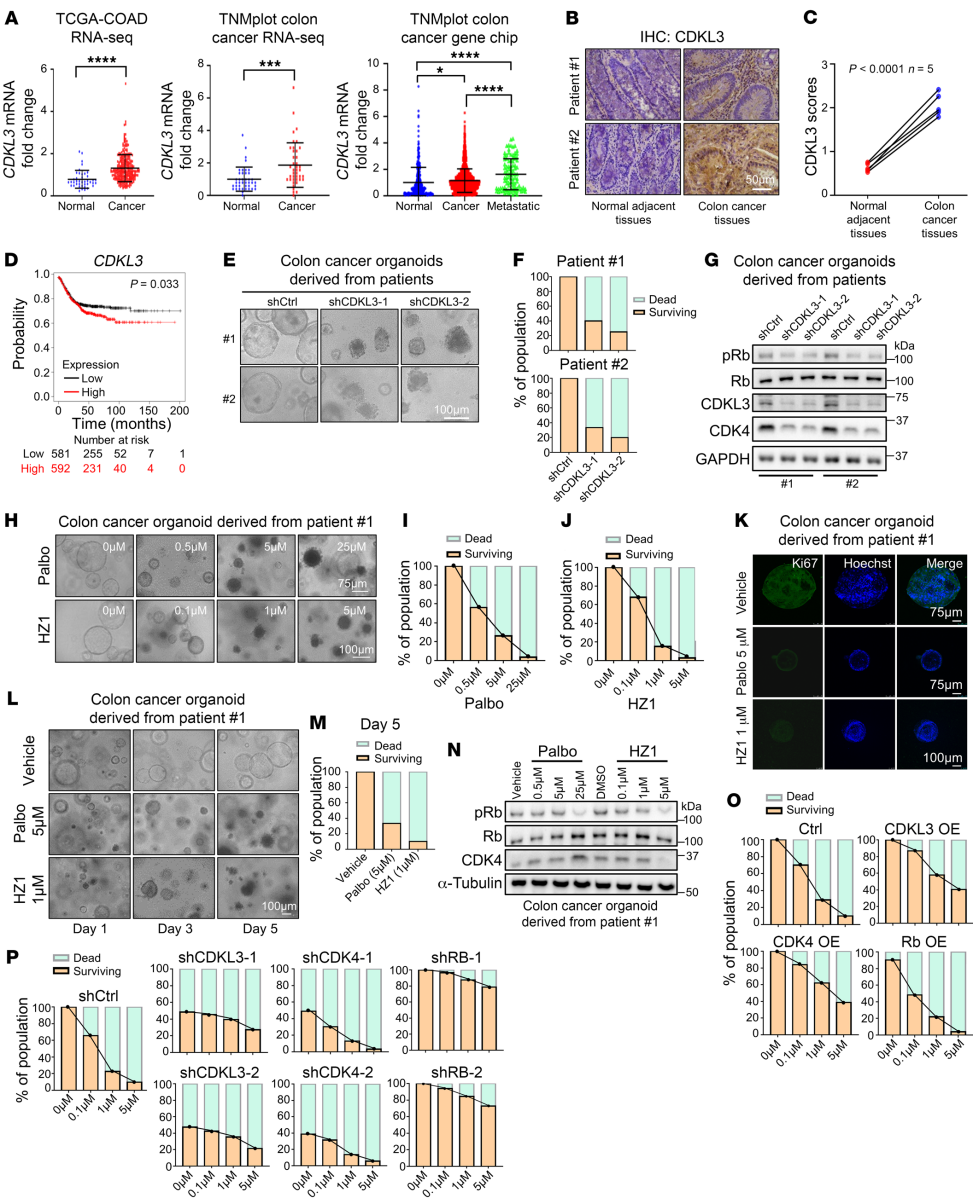
HZ1 has strong clinical implications in colon cancer treatment. (**A**) Multiple transcriptome databases show that *CDKL3* had higher expression in colon cancer than normal tissues. Error bars indicate ± SD, by 2-tailed Student’s *t* test (if 2 sets of data) or 1-way ANOVA (if 3 sets of data). Left: normal *n* = 41, cancer *n* = 282; middle: normal *n* = 41, cancer *n* = 41; right: normal *n* = 377, cancer *n* = 1,450, metastatic *n* = 99. (**B**) Representative IHC staining images showing higher CDKL3 protein level in colon cancer tissues. (**C**) CDKL3 IHC score in colon cancerous and normal adjacent tissues. Error bars indicate ± SD, *n* = 5, by 2-tailed Student’s *t* test. (**D**) Correlation analysis between *CDKL3* expression and poor prognosis in patients with colon cancer, by Kaplan-Meier Plotter (61). (**E** and **F**) Representative images (**E**) and quantification (**F**) of patient-derived colon cancer organoids (PDCCOs) after CDKL3 depletion. (**G**) Immunoblotting assay showing that CDKL3 depletion reduced the levels of pRb and CDK4 in PDCCOs. (**H**) Representative images of PDCCOs under treatments. (**I** and **J**) Quantification of percentage of surviving and dead organoids under palbociclib (**I**) and HZ1 (**J**) treatments in **H**. (**K**) Representative immunofluorescence images of PDCCOs under palbociclib and HZ1 treatment. Ki67: proliferation marker. (**L**) Representative images of PDCCOs under palbociclib and HZ1 treatment at different time points and different dosages. (**M**) Quantification of percentage of surviving and dead organoids under treatments at day 5. (**N**) Immunoblotting assay showing that HZ1 decreased the levels of pRb and CDK4 in patient-derived colon cancer organoids. (**O** and **P**) Quantification of percentage of surviving and dead organoids under CDKL3/CDK4/Rb overexpression (**O**) or depletion (**P**) conditions. Palbo, palbociclib. All images in the same panel are under the same amplification scales unless specified. *P < 0.05; ***P < 0.001; ****P < 0.0001.

We analyzed multiple clinical cancer databases to further demonstrate the potential clinical importance of CDKL3. The transcriptome databases showed that CDKL3 was much more highly expressed in colon cancer tissue than in normal adjacent tissues (Figure 10A) (60). This was verified by IHC images of patients with colon cancer (Figure 10, B and C, and Supplemental Table 1). Significant difference between cancer and normal tissues was also seen for CDK4 and pRb (Supplemental Figure 8, A–D). Both CDK4 and pRb positively correlated with CDKL3 in the colon cancer tissues (Supplemental Figure 8, E and F). Additionally, a strong correlation existed between a high level of CDKL3 and poor prognosis for patients with colon cancer (Figure 10D) (61). All these clues together suggested that CDKL3 was not favored in colon cancer.

Patient-derived organoids are widely acknowledged and welcomed for both fundamental and translational cancer research owing to their strong clinical relevance (62). We collected primary colon cancer samples from the patients and cultured them according to previously described protocols (62). After 7 days of culturing, 2 of 3 samples successfully formed structured organoids that could be stably maintained (Supplemental Figure 8G). We first depleted CDKL3 in the organoids to observe the effects (Supplemental Figure 8, H and I). After CDKL3 depletion, the organoids dissociated and failed to survive (Figure 10, E and F). Immunoblotting verified that both CDK4 and pRb levels clearly dwindled (Figure 10G). Afterward, we tested the potency of HZ1 on these samples. With the increased administered doses, the colon cancer organoids clearly showed a rising proportion of dissociation and death (Figure 10, H–J, and Supplemental Figure 8, J–L). Treatment with HZ1 could eradicate almost 100% of the patient tumor organoids with 72 hours at the concentration of 5 μM (Figure 10, H–J, and Supplemental Figure 8, J–L). The proliferation marker Ki67 vanished after 24 hours of HZ1 treatment as shown by immunofluorescence (Figure 10K). Under 1 μM HZ1 administration for 5 days, 80%–90% of colon cancer organoids died (Figure 10, L and M, and Supplemental Figure 8, M and N). Similar to the data obtained from cell lines, HZ1 markedly decreased the levels of pRb and CDK4 (Figure 10N and Supplemental Figure 8O), hence effectively triggering cell cycle arrest and tumor death. We also interrogated the response of the cancer organoids to HZ1 with the genetic modulations of CDKL3, CDK4, or Rb. Consistent with the cell line data, cancer organoids became more sensitive to HZ1 with the depletion of CDK4 or increased expression of Rb (Figure 10, O and P, and Supplemental Figure 8, P–V). Forced expression of CDKL3 and CDK4 both required higher HZ1 concentration to inhibit organoid growth (Figure 10O and Supplemental Figure 8, R–U). Organoids with CDKL3 or Rb depletion were insensitive to the treatment (Figure 10P and Supplemental Figure 8, P–T and V). Collectively, we have established a robust clinical connection between CDKL3 and colon cancer overall. A CDKL3 inhibitor such as HZ1 could efficiently clear the tumor burden derived from the colon cancer patient. These results strongly implicate that HZ1 and a CDKL3-targeting strategy have great translational potential in the clinic.

## Discussion

In this work, we identified CDKL3 as a crucial regulator of cell cycle progression in cancer, phosphorylating Rb to initiate the cell cycle from quiescence and preserving CDK4 to maintain G_1_ phase advancement. These 2 events both contribute to the accelerated cell cycle that is required for rapid growth in cancers. The proposed small-molecule inhibitor against CDKL3 was shown to have great potency in cancer treatment. We discovered that CDKL3 may function in pair with cyclin A2 at the G_0_-to-G_1_ transition. Unlike cyclin D, cyclin A2 is present in G_0_ phase, which is readily engaged by CDKL3. Therefore, CDKL3–cyclin A2 together run the first leg in the “relay race” of the cell cycle. CDKL3 loss caused severely delayed G_0_-to-G_1_ transition and alleviated cancer cell growth.

Meanwhile, CDKL3 can give a big push to the “second-leg runner,” CDK4. Through direct phosphorylation on CDK4 T172, CDKL3 prevents ubiquitin-proteasomal degradation of CDK4 in cancer cells, thus preserving a sufficient amount of CDK4 for G_1_ phase “highway racing.” Though phosphorylation of T172 was uncovered previously as a demonstration of CDK4 activation (32, 33), the underlying mechanism remained obscure. It was found in this work that phosphorylation of T172 can stabilize CDK4 by precluding the recognition of E3 ubiquitin ligases including UBE3A, Stub1, and Trim28. Among them, Trim28 is identified as an E3 ligase of CDK4 in this work, which causes the K48-linked polyubiquitination of CDK4 in the absence of T172 phosphorylation.

As a member of the CDKL kinase subfamily, both the functional and the molecular understanding of CDKL3 is underexplored. We have provided direct evidence that CDKL kinase can enter the nucleus and function inside. Also, we discovered that the conserved putative cyclin-binding α-helix on CDKL kinase in fact binds to cyclins and is functionally required. After rational design and synthesis, we also presented the potent CDKL3 inhibitor HZ1 for cancer treatment. This inhibitor is first-in-class, and has very low dosage requirement in cancer growth repression both in vitro and in vivo. After testing on patient-derived samples, we believe that HZ1 genuinely provides a promising perspective in cancer therapy. HZ1 can also be used together with other inhibitors as combinatory therapy and, more importantly, may overcome resistance to CDK4/6 inhibitors.

For future directions, the functional importance can be strengthened by transgenic animals. In a separate study, we have generated *Cdkl3*-floxed mice and discovered the important role of *Cdkl3* in fatty liver diseases. We are generating cancer model mice with conditional knockout of *Cdkl3* to validate the cell cycle–promoting effect of CDKL3. Also, the question of how CDKL3 is regulated appears very intriguing to us, which is the next goal to pursue. Further design and tests of CDKL3 inhibitors based on HZ1 should be conducted for higher potency and specificity. Possible off-target effects of these molecules should be carefully examined in the preclinical models.

## Methods

Further information can be found in Supplemental Methods.

### Sex as a biological variable

In this study, sex was not considered as a biological variable. All mice used in this study were male.

### Cell lines

This study used HEK293T (ATCC, catalog CRL-11268), U2OS (ATCC, catalog HTB-96), DLD-1 (National Collection of Authenticated Cell Cultures, China, catalog TCHu134), HeLa (ATCC, catalog CCL-2), MDA-MB-231 (ATCC, catalog HTB-26), MCF-7 (ATCC, catalog HTB-22), and T47D (ATCC, catalog HTB-133) cells. All cell lines (parental and genetically modified) were cultured in DMEM (Gibco) medium supplemented with 10% fetal bovine serum and 100 mg/mL of penicillin/streptomycin/glutamine (Gibco) in humidified incubators with 5% CO_2_ at 37°C. All cell information is listed in Supplemental Table 2.

### Animal work and treatment

BALB/cA nude mice (strain NO 13001A) were purchased from Beijing HFK Bioscience Co. Ltd. C57BL/6J WT (strain NO.N000013) and C57BL/6J *Apc^min/+^* mice (strain NO.T001457) were purchased from GemPharmatech. The nucleotides encoding the 850th amino acid of the *Apc* gene were mutated to a stop codon, resulting in early termination of translation. After crossing with C57BL/6J WT mice, we generated mice with the genotypes *Apc^+/+^* and *Apc^min/+^*. Examples of mouse genotyping are shown in Supplemental Figure 7L, and the primers are listed in Supplemental Table 2.

### Antibodies and immunoblotting

Cells were prepared by passive lysis buffer as described above, resuspended in SDS loading buffer, and boiled at 95°C for 5 minutes to prepare protein samples. Protein samples were separated using 6%–10% SDS-PAGE gels, and PVDF membranes (Millipore) were used for transfer. Then, PVDF membranes were blocked in 3% BSA diluted with TBS-T buffer, and incubated with indicated primary antibodies at 1:1,000 dilution overnight at 4°C. After washing 3 times with TBS-T for 10 minutes each time, the membranes were treated with corresponding secondary antibodies at 1:5,000 dilution at room temperature for 45 minutes. Before exposure, the membranes were washed 3 times with TBS-T buffer for 10 minutes each time. The Tanon chemiluminescence substrate kit and Tanon 5200 Chemiluminescence Imaging System were used. Protein levels were quantified by ImageJ. All antibody information is listed in Supplemental Table 2. The polyclonal CDKL3 antibody was customized and produced by ABclonal. All other antibodies were commercial.

### Statistics

ImageJ was used to quantify the results of immunoblotting, BrdU immunofluorescence, and colony formation assay. GraphPad Prism 8 software was used for statistical analyses. *P* values less than 0.05 were considered statistically significant.

#### BrdU immunofluorescence results analysis.

Three fields were randomly selected and about 100 cells were counted per field to calculate the proportion of BrdU-positive cells and mean BrdU intensity per cell. Experimental results are shown as the mean ± SD, and the *n* value of each set is indicated in each figure panel. One-way ANOVA was performed for statistical analysis.

#### Flow cytometry analysis.

Flow cytometry analysis was performed using FlowJo version 10.0.7r2. Experimental results are shown as the mean ± SD, *n* = 3 replicates, and 2-tailed Student’s *t* test (if 2 sets of data) or 1-way ANOVA (if 3 or more sets) was performed for statistical analysis.

#### Quantification of protein immunoblotting results.

The results of immunoblotting were quantified with ImageJ. The protein expression values in CHX-blocking assay were normalized to those of GAPDH or tubulin. The protein expression values of pRb were normalized to those of Rb. Two-tailed Student’s *t* test was performed for statistical analysis.

#### MTT assay.

For MTT assay, OD_490_ values at each time point were plotted in GraphPad Prism. Experimental results are shown as the mean ± SD, *n* = 3 replicates, and 2-way ANOVA was performed for statistical analysis.

#### Quantitative real-time PCR.

For quantitative real-time PCR, gene expression values were normalized to those of GAPDH. Data processing was performed using the 2^–ΔΔCt^ method. Experimental results are shown as the mean ± SD, *n* = 3 replicates, and 1-way ANOVA was performed for statistical analysis.

#### Colony number.

We used ImageJ (NIH) to analyze the colony formation results. Experimental results are shown as the mean ± SD, *n* = 3 replicates, and 1-way ANOVA was performed for statistical analysis.

#### Tumor volumes of athymic nude mice.

Tumor volume was calculated using the formula: ½(length × width^2^). Images were drawn after the tumor volumes were obtained in combination with the corresponding time points. Experimental results are shown as the mean ± SD, *n* = 10, and 2-way ANOVA was performed for statistical analysis.

#### Tumor weight of athymic nude mice.

The tumor weight was measured after sacrifice of the nude mice and dissection of the tumor. Images were drawn based on the weight results. Experimental results are shown as the mean ± SD, *n* = 10, and 2-tailed Student’s *t* test (if 2 sets of data) or 1-way ANOVA (if 3 sets) was performed for statistical analysis.

#### Tumor number and volume in the intestines of Apc^min/+^ mice.

After the end of the experiment, the intestines of *Apc^min/+^* mice under different treatments were removed. The number and volume of different groups were recorded and measured. For tumor number, experimental results are shown as means ± SD, *n* = 5 per group, by 1-way ANOVA. For tumor volume, experimental results are shown as means ± SD, by 1-way ANOVA. For the *Apc*^+/+^ plus PBS group, *n* = 3; for the *Apc^min/+^* plus PBS group, *n* = 71; for the *Apc^min/+^* plus C3I-22 group, *n* = 10. SuperPlot analysis was performed according to the instructions (63).

#### Semiquantitative scoring of IHC results.

For IHC, experimental results are shown as the mean ± SD, *n* = 5 replicates, and 2-tailed Student’s *t* test was performed for statistical analysis.

#### Transcriptome database analysis.

The expressions of CDKL3 mRNA level were acquired from TNMplot (60) and The Cancer Genome Atlas (TCGA) data sets. After normalization, *P* values were tested with 2-tailed Student’s *t* test or 1-way ANOVA with post hoc Tukey’s post hoc test between groups using GraphPad Prism 8.

#### Analysis of expression correlation of CDKL3 with CDK4 and pRb.

IHC scores were used for correlation analysis by 2-tailed Spearman’s correlation, *n* = 5.

### Study approval

The procedures performed on patient tissue samples, including organoid culturing and IHC staining, were in accordance with the standards of ethical approval number EC-2023-KS-O43 (China Medical University). The usage of human colon cancer samples was approved by The Ethics Committee of the Fourth Affiliated Hospital of China Medical University (Shenyang, China). Written informed consent of the patients was received prior to participation. General patient information is listed in Supplemental Table 1.

Production of mice and all mouse-related research protocols in the study were approved by the Institutional Animal Care and Use Committee of Northeastern University under protocols NEU-EC-2022A019S and NEU-EC-2022A048. In this study, all animals received humane care according to the criteria outlined in the *Guide for the Care and Use of Laboratory Animals* (National Academies Press, 2011).

### Data availability

All data are available in this article. All the data used to generate graphs are provided in the Supporting Data Values file.

## Author contributions

RS and SW designed this study. HZ, JL, SZ, LM, ZP, HY, CM, YW, QH, and ZL performed the experiments and analyzed the data. XZ, LC, LL, TF, DG, LY, XP, CD, SW, and RS wrote and revised the manuscript. SW and RS oversaw this project. HZ focused on the mechanistic study and JL focused on the biology; both share equal contribution as co–first authors. HZ started and followed through the whole study, and JL joined the study 2 years later than HZ; hence, HZ is named before JL.

## Supplementary Material

Supplemental data

Unedited blot and gel images

Supporting data values

## Figures and Tables

**Table 2:**
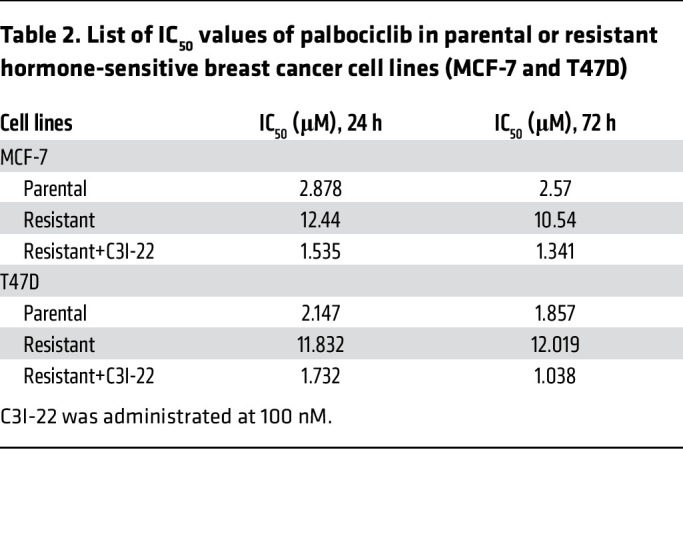
List of IC_50_ values of palbociclib in parental or resistant hormone-sensitive breast cancer cell lines (MCF-7 and T47D)

**Table 1:**
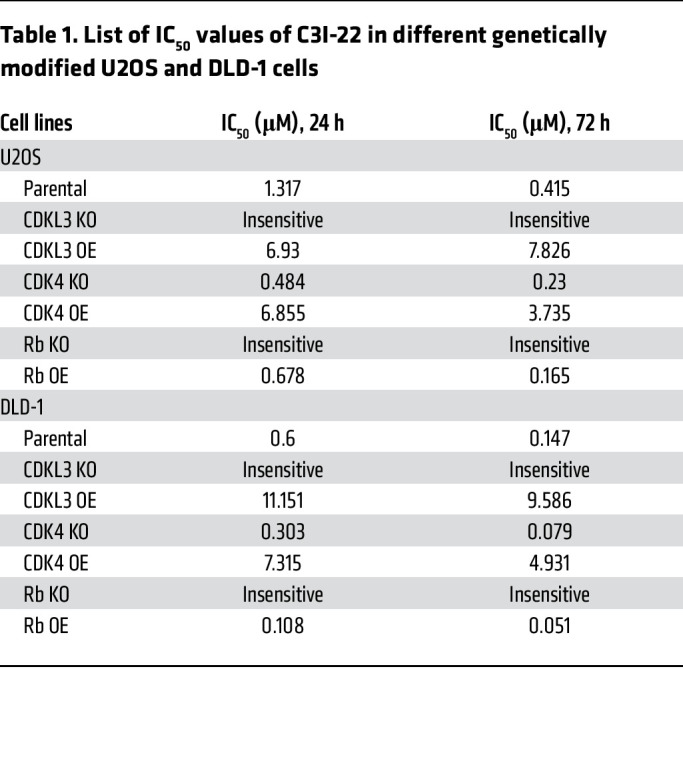
List of IC_50_ values of C3I-22 in different genetically modified U2OS and DLD-1 cells

## Version 1 07/04/2024

In-Press Preview

## Version 2 08/15/2024

Electronic publication
