# Supplementary material for: CDKL3 is a targetable regulator of cell cycle progression in cancers
Source: J Clin Invest. 2024 Jul 4;134(16):e178428. doi: 10.1172/JCI178428 (PMC11324297; doi:10.1172/JCI178428)
Supplement: Supplemental data [file jci-134-178428-s137.pdf]

CDKL3 is a targetable regulator of cell cycle progression in cancers

Haijiao Zhang, Jiahui Lin, Shaoqin Zheng, Lanjing Ma, Zhongqiu Pang, Hongyi Yin, Chengcheng Meng, Yinuo Wang, Qing Han, Xi Zhang, Zexu Li, Liu Cao, Lijun Liu, Teng Fei, Daming Gao, Liang Yang, Xueqiang Peng, Chen Ding, Shixue Wang, Ren Sheng

Supplemental Materials

Supplemental Methods

Supplemental Figure 1 to 8

Supplemental Table 1 and 2

References

Supplemental Methods

Subcutaneous tumor transplantation

DLD-1 cells were trypsinized and resuspended by PBS and held at 4°C. A density of 1×10^6 DLD-1 cells were subcutaneously injected into nude mice (Beijing HFK Bioscience CO., LTD) at per site. Trypan blue was used to determine the number and viability of the designed cells.

Clones and constructs

All plasmids were listed in Supplemental Table 2. Plasmids were transformed in *E.coli* NEB®5α strain for amplification and extracted by OMEGA Endo-free Plasmid Mini Kit. Thermo Nanodrop 2000 was used to determine the concentrations of all plasmids.

Flow cytometry assay

To analyze DNA content, cells were incubated with 50μM BrdU in media for 1 hour, fixed with 70% ethanol and kept at 4°C for at least 30 minutes. The cells were washed twice with cold PBS, followed by 1.5M HCl , 0.5% Triton X-100 in PBS for 30 minutes at room temperature. The cells were washed twice with cold PBS again, and then resuspended in 0.1M NaB_4O_7 (pH 8.5), and washed twice again with cold PBS. BrdU immunolabeling was performed with anti-BrdU mAb (Cell Signaling Technology, 1:200) for 1 hour. The cells were washed twice with cold PBS, and then bound with a fluorescent secondary antibody (Invitrogen,1:500) for 30 minutes. Cells were treated with 200 μg/mL RNase A and 20mg/mL of propidium iodide (PI) in PBS at 37°C for 45 minutes. Cell cycle analysis was performed using BD LSR-Fortessa flow cytometer. For the quantitative analysis, the unstained cells were used to determine the proper forward scattering (FSC) and side scattering (SSC) at the beginning. Next, PI-A (area) /PI-W (width) was used to circle the single cells. The 2N-centered cluster with low BrdU staining is considered as G0/G1 cluster (1-3). The 4N-centered cluster is considered as G2/M cluster. The contents between 2N and 4N and with high BrdU staining are considered as S phase.

For combined RNA and DNA analysis, freshly ethanol-fixed cells were resuspended in 1mL of 20mg/mL of PI and 1μg/mL Pyronin Y in PBS, and incubated for 40 minutes at 37°C. Cell cycle analysis was performed using BD LSR-Fortessa flow cytometer, and analysis was performed using FlowJo version 10.0.7r2. For the quantitative analysis, the G0/G1 and G2/M clusters were separated based on the DNA content at 2N and 4N, respectively. The contents in between are considered as S phase (4-6). At the left boundary of S phase, a horizonal line was drawn to show the bottom-left corner of S phase and to separate high and low RNA-contents. High-RNA-content cells in the top-left quadrant (adjacent to S-phased cells) were considered as G1-phased cells which had prepared sufficient RNA to enter S phase. Botton-left quadrant cells were defined as G0 cells due to the low RNA content and lack of continuity with S-phased cells.

Cycloheximide-blocking assay

Cells were cultured in 24-well plates and treated with cycloheximide (CHX) 300μg/mL at the indicated times. After washed by PBS, cells were prepared for immunoblotting as described above.

Knockdown assay using small interfering RNAs (siRNAs)

After DLD-1 cells grew to 70% to 80% confluence in 6-well plates with DMEM

supplemented with 10% FBS, transfection solution was configured as follows: *CCND1/CCND2/CCND3* siRNA were added into 60 μL reagent A (final concentration of each siRNA was 20 nM), and 8 μL reagent B (D-Nano Therapeutics) was added into the solution. After mixing, the solution was incubated for 5 minutes at RT. Appropriate volume of growth medium was added into the solution to a final volume of 200 μL. The transfection solution was added into plates. Cells were incubated in humidified incubators with 5% CO_2 at 37°C for 48 hours.

Cell transfection

Transfection was done using Neofect. Transient cDNA transfection was performed using Neofect according to the specification. Plasmids were diluted using DMEM and mixed with Neofect. The complexes were incubated for 20 minutes at room temperature (RT) and added to HEK293T cells via growth medium. After 24 hours, the medium was replaced by fresh medium.

Lentivirus production and infection

For lentivirus production, psPAX2 vector (Addgene), pCMV-VSV-G (Addgene), the desired customized Lenti-EF1α-puro plasmids, pLKO plasmids, Lenti-Cas9-puro sgCDKL3, sgCDK4 and other lentiviral vector-based plasmids were co-transfected in HEK293T cell by the ratio of 5:1:5 in mass (ng). Medium with lentivirus was collected and used to infect corresponding cells.

For lentiviral infection, 0.5-1mL medium with lentivirus was collected and added to 1mL fresh medium with polybrene (Santa Cruz Biotech, 1:1,000). After 48 hours, resistance selection was done by refreshing the medium with puromycin or blastomycin (Invivogen).

Cell line generation

CDKL3 KO cell line: At least three sgRNA sequences were designed and cloned into the Lenti-Cas9-puro vector (Addgene). U2OS, DLD-1 and HeLa cell pools stably expressing different Lenti-Cas9-puro-sgCDKL3 were generated after lentivirus infection and puromycin resistance selection. Subsequently, immunoblotting was determined by CDKL3 antibodies to determine cell pools. Other KO cell lines were generated by the same approach.

Knockdown assay using small interfering RNAs (siRNAs)

After DLD-1 cells grew to 70% to 80% confluence in 6-well plates with DMEM supplemented with 10% FBS, transfection solution was configured as follows: *CCND1/CCND2/CCND3* siRNA were added into 60 μL reagent A (final concentration of each siRNA was 20 nM), and 8 μL reagent B (D-Nano Therapeutics) was added into the solution. After mixing, the solution was incubated for 5 minutes at RT. Appropriate volume of growth medium was added into the solution to a final volume of 200 μL. The transfection solution was added into plates. Cells were incubated in humidified incubators with 5% CO_2 at 37°C for 48 hours.

Co-immunoprecipitation

For co-immunoprecipitation, total lysates of cells were incubated with Protein G-agarose (or followed by CDK4 antibody incubation, 1:100), α-FLAG-agarose, α-MYC-agarose or α-HA-agarose overnight at 4°C. The next day, the resins were washed thoroughly for 5 times with PLB, incubated with shaking for 10 minutes each time at

4°C, resuspended in SDS loading buffer, boiled at 95°C for 5 minutes, and used for the immunoblotting.

In vitro kinase assay

FLAG-CDKL3, MYC-cyclin A2, B1, D1 and E1 were transfected in HEK293T cells for 48 hours. And the cells were starved in FBS-free medium for 8 hours. The cells were then lysed and the expressed proteins were enriched on α-FLAG-agarose or α-MYC-agarose. Next, corresponding peptides (500μg/mL) were added to the agarose-containing solution for elution. The substrate required for the reaction was obtained as described (see in vitro protein purification). A 30μL reaction system (0.1mM ATP, 2mM DTT, 50mM HEPES, 0.01M $MgCl_2$, suitable volume of substrate and kinase) was incubated at 37°C for 30 minutes, and terminated by 95°C boiling for 5 minutes. The samples were then subjected to immunoblot.

Colon cancer patient-derived organoids culture

Fresh colon cancer patient tumor tissues were obtained by surgery (Department of General Surgery, Fourth Affiliated Hospital, China Medical University, Shenyang 110032, China) and stored in the Advanced DMEM/F-12 (Gibco). The general protocol was followed as described (7).

Immunofluorescence assay

Cells were seeded onto glass coverslips and fixed by 4% paraformaldehyde (PFA) in PBS for 15 minutes at 37°C. They were then incubated in 0.5% Triton X-100 in PBS for 30 minutes at RT, followed by anti-CDKL3 antibody (1:400 in the buffer, 12 to 16 hours, 4°C), and bound with a fluorescent secondary antibody for 30 minutes. Rinsed by PBS 3 times, coverslips were incubated with Hoechst (Invitrogen) at RT. Cells were visualized by inverted confocal microscopy. Regarding the immunofluorescence of BrdU, cells were incubated with 10μM BrdU in media for 1 hour and then fixed in 4% PFA and kept at 4°C for 15 minutes. The cells were washed twice with PBS, followed by 1M HCl , 0.2% Triton X-100 in PBS for 20 minutes at RT, and then resuspended the cells with 0.1M NaB_4O_7 (pH 8.5), allowing them to stand for 30 minutes. The cells were washed twice with cold PBS. BrdU immunolabeling was performed with anti-BrdU mAb (Cell Signaling Technology, 1:200) for 1 hour. The cells were washed twice with cold PBS, and then bound with secondary antibody (Invitrogen,1:500) for 30 minutes. Then, fluorescence images were taken using a Leica TCS SP8.

Ubiquitination assay

For ubiquitination assay, indicated V5-ubiquitin plasmids and corresponding plasmids were co-transfected in HEK293T cells for 48 hours. Before lysed by PLB, cells were treated with MG132 (10μM) for 6 hours to maintain the same protein levels.

Reverse transcription and quantitative real-time PCR

For RT-qPCR, total RNA from cells was isolated and obtained by UNIQ-10 Column Trizol Total RNA Isolation Kit (Sangon Biotech). Total RNA was reverse transcribed into cDNA using MonScript™ RTIII All-in-One Mix (Monad) following the manufacturer's specification. Quantitative RT-PCR were performed using MonAmp™ ChemoHS qPCR Mix (Monad). All primers were designed based on the primer bank of Massachusetts General Hospital (https://pga.mgh.harvard.edu/cgi-bin/primerbank). Primer sequences are listed in Supplemental Table 2. All experiments were performed

in triplicate. The expression values were normalized to those of *GAPDH*.

Double thymidine block

After the cells grew to 30% to 40% confluence in 24-well plates with DMEM supplemented with 10% FBS, thymidine was added at the concentration of 2mM. After cultured at 37°C for 16 hours, cells were gently rinsed with PBS to remove thymidine and DMEM supplemented with 10% FBS was added. After 9 hours, second round of thymidine was added at a concentration of 2mM for another 16 hours. At this point cells were at G1/S boundary. To release, cells were washed with PBS and incubated with fresh media. After incubating for the corresponding time, cells were collected for immunoblotting.

FBS starvation release assay

After the cells grew to 40% to 60% confluence, the cells were gently rinsed 3 times with PBS to remove FBS contents from the cell surface, and FBS was withdrawn for 24 hours. Then, medium containing FBS were replaced as indicated.

Chemical treatments

Cdk1/2 Inhibitor III was dissolved in DMSO with a concentration of 25mM as stock solution. Cells were treated at a final concentration of 1.2μM. 5μM was used for in vitro kinase assay.

Palbociclib was dissolved in DMSO with a concentration of 100mM as stock solution. Cells were treated with Palbociclib at a final concentration of 0.8μM. 5μM was used for in vitro kinase assay.

Thymidine was dissolved in ddH_2O with a concentration of 200mM. Cells were treated with Thymidine at a final concentration of 2mM.

ASC67 was dissolved in DMSO with a concentration of 300mM as stock solution. For IC_{50} assay, cells were treated at a concentration range from 0 to 300μM. For in vitro kinase assay, the concentration ranging from 0 to 0.5μM was used.

The inhibitor C3I-22 (HZ1) was dissolved in DMSO with a concentration of 300mM as stock solution. For IC_{50} assay, cells were treated with a concentration range from 0 to 300μM. For in vitro kinase assay, the concentration ranging from 0 to 0.1μM was used. Organoids derived from colon cancer patients were treated with a concentration range from 0 to 5μM. Balb/cA-nude and C57BL/6J-*Apc*$^{min/+}$ mice were treated with a concentration of 1mg/kg (suspended in PBS) through oral gavage.

Other C3I inhibitors were dissolved in DMSO with a concentration of 200mM as stock solution.

In vitro protein purification

GST-tagged proteins were transformed into *E. coli* BL21 competent cells, and then protein expression was induced by adding IPTG (Beyotime Biotechnology) at 18°C for 6 to 10 hours. The bacterial culture was centrifuged (600xg, 10 min, 4°C), and the pellets were resuspended in 20mL lysis buffer (50mM Tris-HCl, 300mM NaCl, and 10% (v/v) glycerol, 1mM PMSF, pH 7.9) and sonicated with 50% power on a SCIENTZ JY92-IIN sonicator for 20 minutes (5 seconds per time, with an interval of 5 seconds). The solution was centrifuged again after sonication (10,000xg, 15 min, 4°C) and the supernatant was collected. An appropriate amount of GST resins was added to the supernatant and the mixture was rotated for 2 to 3 hours at 4°C. The beads were

washed 3 times and the volume to 1mL with TBS was set. The preparation of His-tagged protein was similar to GST-tagged protein. Ni-NTA resin was used for enrichment of His-tagged proteins, followed by protein elution with imidazole (200mM). And the imidazole was removed by overnight dialysis in TBS at 4°C.

In vitro ubiquitination assay

The ubiquitination assay was performed using the ubiquitination kit (Enzo Life Science). A 50μL reaction mixture was prepared as follow: 10× ubiquitination buffer, 50mM DTT, 0.1M Mg-ATP, 20×E1 (His6-tagged recombinant human ubiquitin-activating enzyme), 10×E2 (His6-tagged recombinant human ubiquitin-conjugating enzyme UbcH5b), 1μM E3 (His-Trim28 or His-Trim28 C65/68A,), 20×biotinylated ubiquitin, and appropriate target protein (GST-CDK4). After incubating for 1 hour at 37°C, the reaction was terminated by 95°C for 5 minutes. The samples were then subjected to immunoblotting.

In vitro competition assay

For in vitro competition assay, His-tagged and GST-tagged protein were prepared as previously mentioned. Ni-NTA and GST resins were used to enrich corresponding His-tagged and GST-tagged proteins for 2 hours at 4°C, respectively. Then GST-tagged proteins were eluted by reduced glutathione from the GST resins and dialyzed overnight. The appropriate amount of the first GST-tagged protein was incubated with Ni-NTA resin enriched by His-tagged cyclin A2 at 4°C for 2 hours and then different concentrations of another GST-tagged protein was added into the mixture and incubated at 4°C for 2 hours for the competition. After removing the supernatant by centrifugation, the Ni-NTA beads were then washed with TBS for 3 times, incubated with shaking for 5 minutes each time at 4°C, resuspended in SDS loading buffer, boiled at 95°C for 5 minutes, and used for the immunoblotting.

Methyl thiazolyl tetrazolium (MTT) assay for cell proliferation

Cells were seeded on 96-well plates at a density of 1,500 cells per well in triplicate and incubated for 1-8 days. After seeding, cell density was measured on Day 2, 4, 6, and 8 by adding 10μL thiazolyl blue tetrazolium (MTT; Sigma) at a dose of 0.5mg/mL per well. After 37°C incubation of 4 hours, MTT was removed and 100μL DMSO per well was added for another 10 minutes incubation. Plates ready for testing were measured by Biotek Synergy H1 plate reader at OD_{490}. According to OD_{490} values, the growth curves were plotted. Statistical analysis was done by two-way ANOVA in Prism 8.0.

Colony formation assay

The U2OS and DLD-1 cells were seeded in soft agar in 6-well plates at a density of 1,500 cells per well and cultured for 14 days for colony formation. After the clones grew into certain sizes and numbers, the wells were washed with PBS and fixed with 4% PFA at 37°C for 20 minutes. The wells were stained with 0.5% crystal violet for 30 minutes at RT, gently washed with ddH_2O several times to remove the background and photographed by digital camera. All assays were performed in triplicate.

Establishment of Palbociclib-resistant cells

MCF7 and T47D cells were cultured in DMEM medium (containing 10% FBS) supplemented with different concentrations of Palbociclib for over 3 months. The concentration of Palbociclib ranged from 100 nM to 10 μM. For all experiments, the resistant cells were cultured with fresh DMEM medium (with 10% FBS) without

Palbociclib for 72h before treatment.

Synthesis of compounds

Synthesis of 2-chloro-*N*-(3-cyclopentyl-1*H*-pyrazol-5-yl)pyrimidin-4-amine (3):

To a solution of 2,4-dichloropyrimidine (2) (447 mg, 3.0 mmol, 1.0 eq) in anhydrous DMSO (10 mL) was added 3-cyclopentyl-1*H*-pyrazol-5-aminetriethylamine (1) (500 mg, 3.3 mmol, 1.1 eq) and *N,N*-diisopropylethylamine (0.8 mL, 4.5 mmol, 1.5 eq). The solution was heated at 60°C for 24 hours. The reaction mixture was concentrated in vacuum, the residue was purified by column chromatography using CH_2Cl_2/MeOH as eluent giving 3 (650 mg) in 82% yield. ^{1}H NMR (400 MHz, DMSO-*d6*): δ 12.17 (s, 1H), 10.27 (s, 1H), 8.15 (s, 1H), 3.01 (m, 1H), 1.99 (m, 2H), 1.51-1.72 (m, 6H).

Synthesis of 2-(4-((4-((3-cyclopentyl-1*H*-pyrazol-5-yl)amino)pyrimidin-2-yl)amino)phenyl)acetonitrile (ASC67):

To a solution of 3 (200 mg, 0.76 mmol, 1.0 eq) in *n*-BuOH (8 mL) was added 2-(4-aminophenyl)acetonitrile (102 mg, 0.76 mmol, 1.0 eq) and concentrated hydrochloric acid (0.1 mL) in a sealed tube. The solution was heated at 100°C for 24 hours. The reaction mixture was concentrated in vacuum, the residue was purified by column chromatography using CH_2Cl_2/MeOH as eluent giving ASC67 (200 mg) in 73% yield. ^{1}H NMR (400 MHz, DMSO-*d6*): δ 11.98 (s, 1H), 9.50 (s, 1H), 9.12 (s, 1H), 7.98 (s, 1H), 7.77 (dd, J = 8 Hz, 2H), 7.22 (dd, J = 8 Hz, 2H), 6.47 (br, 1H), 6.31 (br, 1H), 3.93 (s, 2H), 3.02 (m, 1H), 2.01 (m, 2H), 1.54-1.72 (m, 6H).

Synthesis of 1-(4-((4-((3-cyclopentyl-1H-pyrazol-5-yl)amino)pyrimidin-2-yl)amino)phenyl)ethan-1-one (C3I-22, aka HZ1):

To a solution of 3 (100 mg, 0.38 mmol, 1.0 eq) in *n*-BuOH (4 mL) was added 1-(4-aminophenyl)ethan-1-one (52 mg, 0.38 mmol, 1.0 eq) and concentrated hydrochloric acid (0.05 mL) in a sealed tube. The solution was heated at 100°C for 24 hours. The reaction mixture was concentrated in vacuum, the residue was purified by column chromatography using CH_2Cl_2/MeOH as eluent giving C3I-22 (HZ1, 95 mg) in 70% yield. ^{1}H NMR (400 MHz, CD_3OD): δ 8.01 (br, 1H), 7.95 (dd, J = 8 Hz, 2H), 7.83 (dd, J = 8 Hz, 2H), 6.44 (br, 1H), 6.28 (br, 1H), 3.10 (m, 1H), 2.56 (s, 3H), 2.09 (m, 2H), 1.62-1.80 (m, 6H).

Synthesis of 4-((4-((3-cyclopentyl-1*H*-pyrazol-5-yl)amino)pyrimidin-2-yl)amino)benzonitrile (C3I-42):

To a solution of 3 (100 mg, 0.38 mmol, 1.0 eq) in *n*-BuOH (4 mL) was added 4-aminobenzonitrile (45 mg, 0.38 mmol, 1.0 eq) and concentrated hydrochloric acid (0.05 mL) in a sealed tube. The solution was heated at 100°C for 24 hours. The reaction mixture was concentrated in vacuum, and the residue was purified by column chromatography using CH_2Cl_2/MeOH as eluent giving C3I-42 (45 mg) in 35% yield. ^{1}H NMR (400 MHz, CD_3OD): δ 8.02 (br, 1H), 7.89 (dd, J = 8 Hz, 2H), 7.59 (dd, J = 8 Hz, 2H), 6.46 (br, 1H), 6.22 (br, 1H), 3.10 (m, 1H), 2.10 (m, 2H), 1.62-1.81 (m, 6H).

Synthesis of 2-(4-((4-((3-cyclopentyl-1H-pyrazol-5-yl)amino)pyrimidin-2-yl)amino)phenyl)ethan-1-ol (C3I-50):

To a solution of 3 (100 mg, 0.38 mmol, 1.0 eq) in *n*-BuOH (4 mL) was added 2-(4-aminophenyl)ethan-1-ol (50 mg, 0.38 mmol, 1.0 eq) and concentrated hydrochloric acid (0.05 mL) in a sealed tube. The solution was heated at 100°C for 24 hours. The

reaction mixture was concentrated in vacuum, and the residue was purified by column chromatography using CH_2Cl_2/MeOH as eluent giving C3I-50 (110 mg) in 80% yield. ^{1}H NMR (400 MHz, DMSO-*d6*): δ 11.94 (s, 1H), 9.46 (s, 1H), 8.93 (s, 1H), 7.94 (s, 1H), 7.60 (dd, *J* = 8 Hz, 2H), 7.08 (dd, *J* = 8 Hz, 2H), 6.42 (br, 1H), 6.34 (br, 1H), 4.60 (t, *J* = 8 Hz, 1H), 3.59 (q, *J* = 8 Hz, 2H), 3.01 (m, 1H), 2.66 (t, *J* = 8 Hz, 2H), 1.99 (m, 2H), 1.61-1.72 (m, 6H).

Synthesis of 4-((4-((3-cyclopentyl-1H-pyrazol-5-yl)amino)pyrimidin-2-yl)amino)benzoic acid (C3I-22-1):

To a solution of 3 (100 mg, 0.38 mmol, 1.0 eq) in 1,4-dioxane (4 mL) was added 4-aminobenzoic acid (53 mg, 0.38 mmol, 1.0 eq) and concentrated hydrochloric acid (0.05 mL) in a sealed tube. The solution was heated at 100°C for 24 hours. The reaction mixture was concentrated in vacuum, and the residue was purified by column chromatography using CH_2Cl_2/MeOH as eluent giving C3I-22-1 (98 mg) in 72% yield. ^{1}H NMR (400 MHz, DMSO-*d6*): δ 12.40 (br, 1H), 10.29 (br, 1H), 8.01 (s, 1H), 7.94 (dd, *J* = 8 Hz, 2H), 7.76 (dd, *J* = 8 Hz, 2H), 6.54 (br, 1H), 6.20 (br, 1H), 2.97 (m, 1H), 1.99 (m, 2H), 1.61-1.72 (m, 6H).

Synthesis of N^2-(4-(2-aminoethyl)phenyl)-N^4-(3-cyclopentyl-1*H*-pyrazol-5-yl)pyrimidine-2,4-diamine (C3I-22-2):

To a solution of 3 (100 mg, 0.38 mmol, 1.0 eq) in *n*-BuOH (4 mL) was added *tert*-butyl (4-aminophenethyl)carbamate (90 mg, 0.38 mmol, 1.0 eq) and concentrated hydrochloric acid (0.05 mL) in a sealed tube. The solution was heated at 100°C for 24 hours. The reaction mixture was concentrated in vacuum, and the residue was washed with diethyl ether to afford C3I-22-2 (80 mg) in 58% yield. ^{1}H NMR (400 MHz, DMSO-*d6*): δ 11.95 (s, 1H), 9.48 (s, 1H), 8.95 (s, 1H), 7.96 (d, *J* = 4 Hz, 1H), 7.63 (dd, *J* = 8 Hz, 2H), 7.08 (dd, *J* = 8 Hz, 2H), 6.38 (br, 2H), 2.99 (m, 1H), 2.74 (t, *J* = 8 Hz, 2H), 2.57 (t, *J* = 8 Hz, 2H), 1.99 (m, 2H), 1.60-1.72 (m, 6H).

Synthesis of C3I-PEG3-Biotin and streptavidin pull-down assay of cellular contents

C3I-50 and Br-PEG3-Biotin react in DMF at the presence of K_2CO_3 to obtain C3I-PEG3-Biotin. Cells were co-incubated with 100nM C3I-PEG3-Biotin or PEG3-Biotin (as negative control) for 6 hrs at 37°C, followed by cell lysis with PLB. After centrifugation, the supernatant was subjected to dialysis (12,000 Da) for 4 hrs. The remaining solution in the dialysis bag was then subjected to streptavidin-conjugated resin for incubation at 4°C for 2hrs. After three times of washing, the resins were boiled in SDS buffer. And the supernatant was subjected to SDS-PAGE and WB detection.

Growth of cells in athymic nude mouse and treatment of tumor tissues

Seventy 6-week-old female athymic nude mice (purchased from Beijing HFK Biotechnology Co., Ltd.) were divided into 7 groups randomly (10 mice per group). DLD-1 cells were trypsinized and resuspended by PBS and kept on ice. 1×10^6 DLD-1 cells were subcutaneously injected on the backs of mice. When tumors emerged subcutaneously, C3I-22 (suspended in PBS) or the same volume of PBS was given through oral gavage daily for 12-14 days. The size of tumor was measured every 2 days. Tumor volume was calculated using the formula: ½ (Length × Width^2). At the end of the experiment, mice were sacrificed and tumors were removed, weighed and

evaluated by immunohistochemistry.

Tumor tissues were lysed by using RIPA lysis buffer (Cowin Bio) containing protease inhibitor cocktail (Roche) and phosphatase inhibitor cocktail (Cowin Bio), and then total protein concentration was measured by using the BCA Kit (Thermo).

Immunohistochemistry

Human patient and mouse tissue samples for immunohistochemistry were fixed in 10% formalin, embedded in paraffin, sectioned, deparaffinized, submerged in citric acid (pH 6.0) and microwaved for antigen retrieval. After cooling to RT, according to the manufacturer's instruction, the sections were treated with 3% hydrogen peroxide, 10% goat serum, and corresponding primary antibodies (1:400 or 1:600). HRP Envision Systems (DAB Kit, MXB Biotechnologies) was used to stain. After sealing with neutral balsam, sections were analyzed with dissecting microscope (Leica DM4000). Hematoxylin and Eosin (Sangon Biotech) staining was done according to the manufacturer's instructions.

A semi-quantitative scoring method was used to assess the expression of targeted protein. The staining intensity was divided into 0 (no staining), 1 (weak staining), 2 (moderate) and 3 (strong). The percentage of cells stained was categorized as 0 (0-5%), 1 (6-25%), 2 (26-50%), 3 (51-75%), and 4 (76%-100%). The final scores were generated by multiplying the staining intensity by percentage of cells.

Organoids immunofluorescence analysis

Organoids sample embedded in Matrigel gel were fixed with 4% PFA (30 minutes, RT). Fixed organoids were collected and centrifuged (800 rpm, 5 minutes) to remove PFA and washed with ultrapure water. Following resuspension in ultrapure water, organoids were spread evenly over adherent slides. Subsequently, organoids spread on adherent slides were permeabilized with 0.2% Triton X-100 in PBS (1 hour, RT) and blocked for 3 hours using 10% goat serum containing 0.01% Triton X-100. Primary antibodies Ki67 (1:50; CST) was subsequently incubated overnight at 4°C. Following at least three washes with PBS the following day, samples were incubated with secondary antibodies (1:1,000; Invitrogen) for 2 hours at 4°C. Following at least three PBS washes, the stained organoids were imaged in confocal microscope (DM6000CS, Leica).

Surface plasmon resonance analysis

Surface plasmon resonance (SPR) assays were performed using a Biacore T200 (Becton Dickinson and Company). In SPR experiments, purified CDKL3 were coupled to CM5 chips by a standard amine-coupling procedure in 10 mM sodium acetate (pH 4.5). Compounds were serially diluted and injected onto a sensor chip at a flow rate of 15μL/min for 90 seconds (contact phase), followed by 180 seconds of buffer flow (dissociation phase). The Kd value was derived using Biacore T200 Evaluation software Version 1.0 (Cytiva) and steady state analysis of data at equilibrium.

Supplemental Figure 1

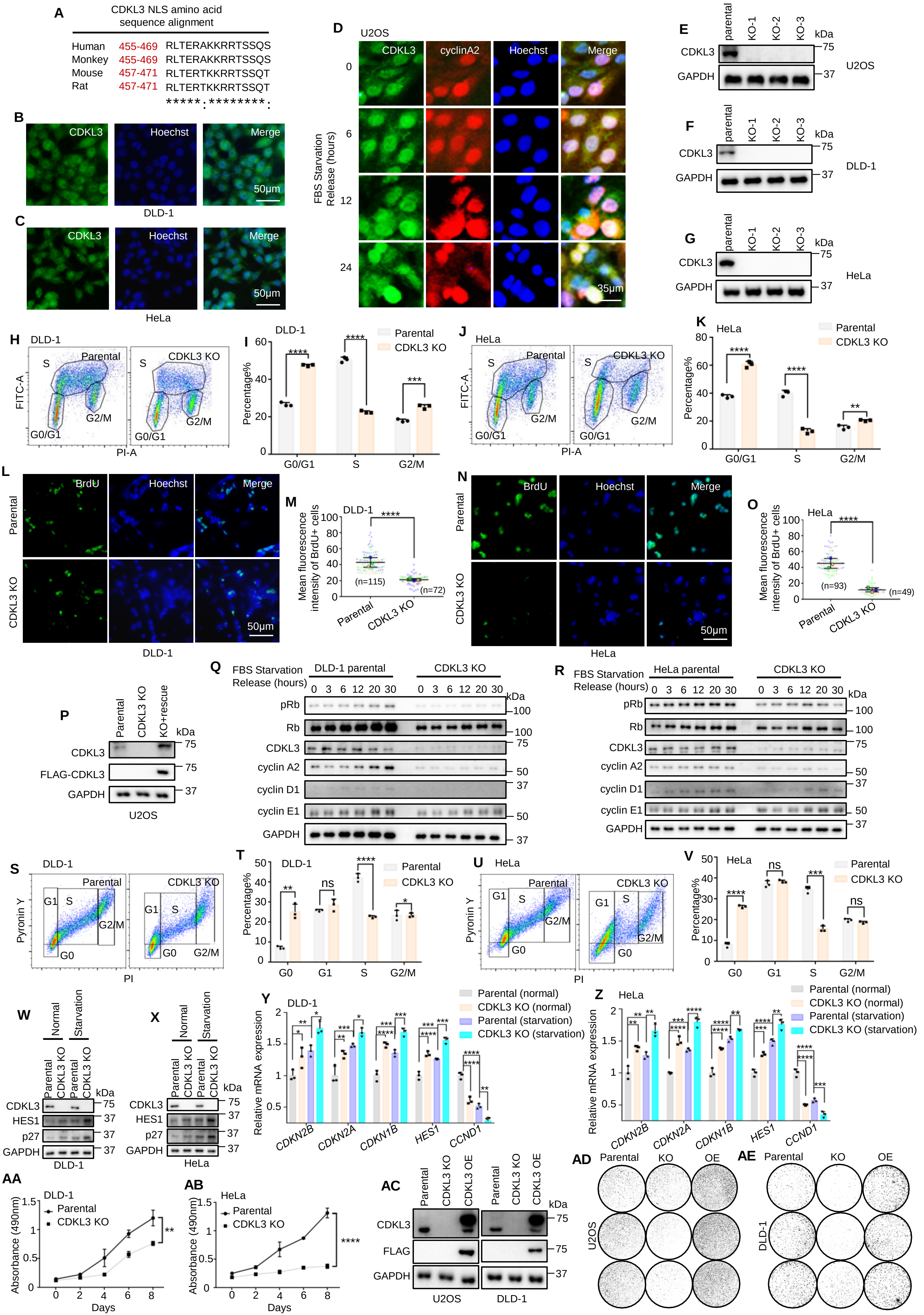

Supplemental Figure 1. Supporting information showing CDKL3-loss abrogates cancer cell growth by impeding G0-to-G1 transition.
(**A**) Sequence alignment showed the putative nucleus-localization sequence (NLS) in CDKL3 was conserved in mammalian (CDKL3 only has direct homolog within mammalians). (**B, C**) Immunostaining showing the nuclear-localization of endogenous CDKL3 in DLD-1 (**B**) and HeLa cells (**C**). (**D**) Immunostaining showing the nuclear-localization of endogenous CDKL3 was independent of cell cycle phases in U2OS cells. Cyclin A2 was used as the marker for cell cycle progression.
(**E-G**) Validation of CDKL3 knockout by multiple sgRNAs by immunoblotting assay in U2OS (**E**), DLD-1 (**F**) and HeLa (**G**). CDKL3 KO-1 in these cells were used and abbreviated as CDKL3 KO in sequel experiments. (**H, I**) Representative flow cytometry results (**H**) and statistical analysis (**I**) of DLD-1 cells with BrdU-FITC/PI dual staining. Error bar means ± SD, n=3, by two-tailed Student's t-test. (**J, K**) Representative flow cytometry results (**J**) and statistical analysis (**K**) of HeLa cells with BrdU-FITC/PI dual staining. Error bar means ± SD, n=3, by two-tailed Student's t-test. (**L, M**) Representative images of immunofluorescence of BrdU in DLD-1 (**L**) and superplot analysis of BrdU-positive cells and mean BrdU intensity per cell (**M**). Green: GFP; Blue: Hoechst. Error bar means ± SD, triplicated, by two-tailed Student's t-test. n value in the panel represents the total number of cells. (**N, O**) Representative images of immunofluorescence of BrdU in HeLa (**N**) and superplot analysis of BrdU-positive cells and mean BrdU intensity per cell (**O**). Green: GFP; Blue: Hoechst. Error bar means ± SD, triplicated, by two-tailed Student's t-test. n value in the panel represents the total number of cells. (**P**) Validation of CDKL3 KO and rescue by immunoblotting. (**Q, R**) Immunoblotting of multiple cell cycle related proteins after serum starvation and release in DLD-1 (**Q**) and HeLa cells (**R**). (**S, T**) Representative flow cytometry results (**S**) of DLD-1 cells with Pyronin Y/PI dual staining and statistical analysis (**T**). Error bar means ± SD, n=3, by two-tailed Student's t-test. (**U, V**) Representative flow cytometry results (**U**) of HeLa cells with Pyronin Y/PI dual staining and statistical analysis (**V**). Error bar means ± SD, n=3, by two-tailed Student's t-test. (**W, X**) Immunoblotting showing CDKL3 ablation increased the protein levels of G0 markers Hes1 and p27 in DLD-1 (**W**) and HeLa (**X**) cells. (**Y, Z**) RT-pPCR assay showing the transcription of G0 markers genes (*CDKN2B*, *CDKN2A*, *CDKN1B* and *HES1*) increased in the absence of CDKL3 in DLD-1 (**Y**) and HeLa (**Z**) cells. *CCND1* was used as the marker of G1 phase. Error bar means ± SD, n=3, by one-way ANOVA. (**AA, AB**) MTT assay showing the growth of DLD-1 (**AA**) and HeLa cells (**AB**). Error bars mean ± SD, n=3, by two-way ANOVA. (**AC**) Validation of CDKL3 KO and overexpression in U2OS and DLD-1 cells by immunoblotting. (**AD, AE**) The gross images of colony formation of U2OS (**AD**) and DLD-1 (**AE**) cells under three-dimensional culturing condition. The quantifications were shown in Figure 2H and 2I. All images in the same panel are under the same amplification scales. ns, not significant; *, p<0.05; **, p<0.01; ***, p<0.001; ****, p<0.0001.

Supplemental Figure 2

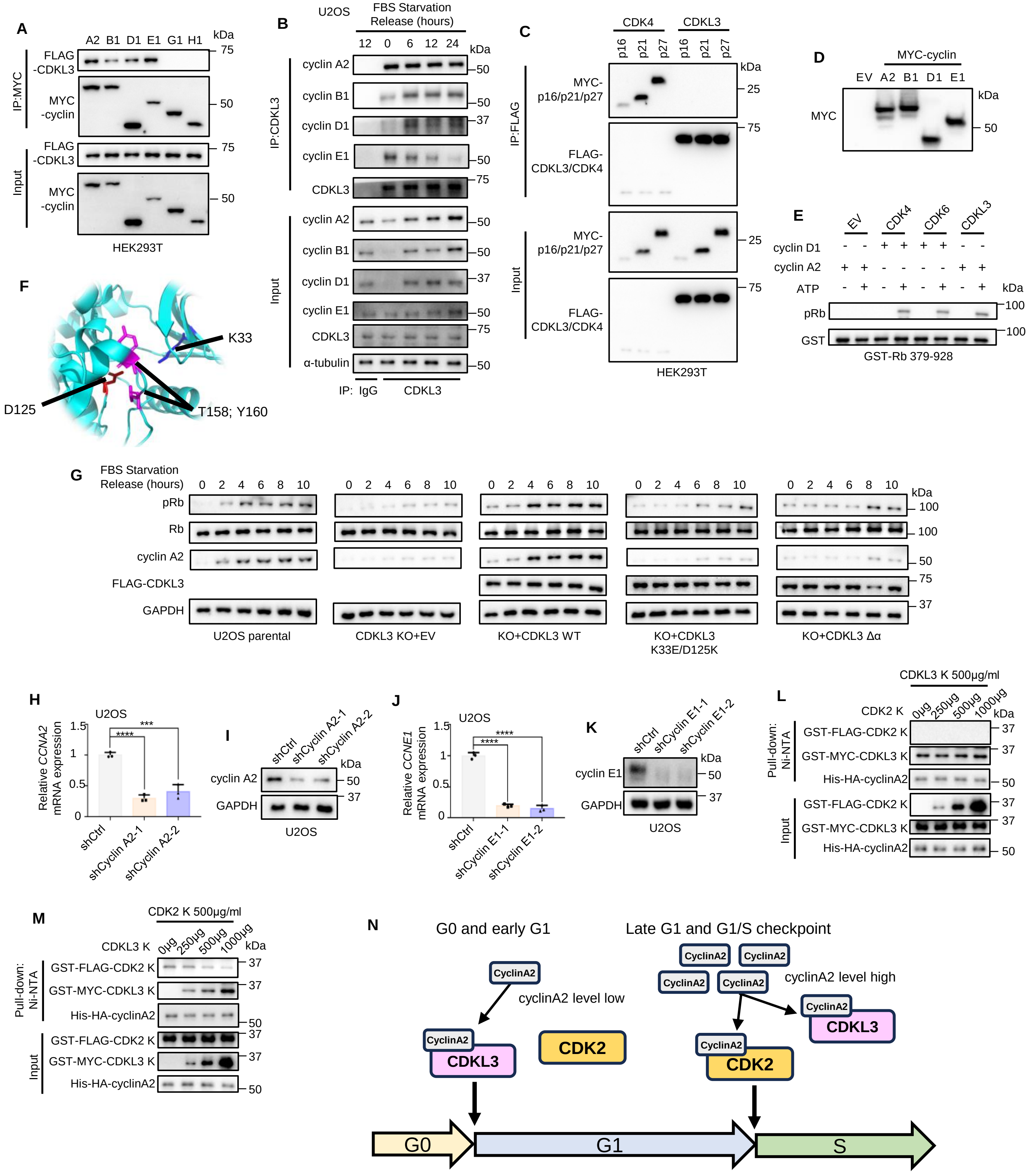

Supplemental Figure 2. Supporting information showing CDKL3 phosphorylates Rb for cell cycle entry when coupling with cyclin A2.

(**A**) Co-IP assay showing exogenous cyclin A2, B1, D1 and E1 binds to CDKL3. (**B**) Co-IP assay at different cell cycle phases showing endogenous CDKL3-cyclin A2 binding was largely unaltered. Starting from G0, CDKL3-cyclin B1/D1 binding gradually increased as CDKL3-cyclin E1 binding decreased. (**C**) Co-IP assay showing exogenous CDKL3 does not bind to p16, p21 or p27. (**D**) Validation of purified cyclins by immunoblotting. (**E**) In vitro kinase assay showing CDKL3 phosphorylates Rb (379-928) to the similar extent of CDK4/6. (**F**) Schematic diagram of K33 (ATP binding site), D125 (conserved substrate-binding Asp), T158/Y160 (TxY motif) sites on CDKL3 kinase domain. PDBID: 3ZDU. (**G**) Immunoblotting assay showing CDKL3 K33E/D125K and Δα mutants lost the capacity of promoting the initial Rb phosphorylation after serum starvation and release in U2OS cells. (**H, I**) Validation of cyclin A2 knockdown by RT-qPCR (**H**) and immunoblotting (**I**) in U2OS cells. shCyclin A2-1 was abbreviated as shCyclin A2 in the sequel experiments. Error bar means ± SD, n=3, by one-way ANOVA. (**J, K**) Validation of cyclin E1 knockdown by RT-qPCR (**J**) and immunoblotting (**K**) in U2OS cells. shCyclin E1-1 was abbreviated as shCyclin E1 in the sequel experiments. Error bar means ± SD, n=3, by one-way ANOVA. (**L, M**) In vitro competition assay showing the pre-formed CDKL3 kinase domain-cyclin A2 complex cannot be effectively disrupted by the addition of CDK2 kinase domain (**L**), whereas titration of CDKL3 kinase domain can disrupt the binding between CDK2 kinase domain and cyclin A2 (**M**). This demonstrates that cyclin A2 binds to CDKL3 with higher affinity than CDK2. K: Kinase domain. (**N**) Working model of CDKL3 and CDK2 associating with cyclin A2. Cyclin A2 has low abundance during cell quiescence, so that cyclin A2 preferably binds to CDKL3 for G0 exit. In late G1 phase, cyclin A2 becomes abundant, which enables the activation of CDK2, potentially leading to the G1-to-S transition. ***, p<0.001; ****, p<0.0001.

Supplemental Figure 3

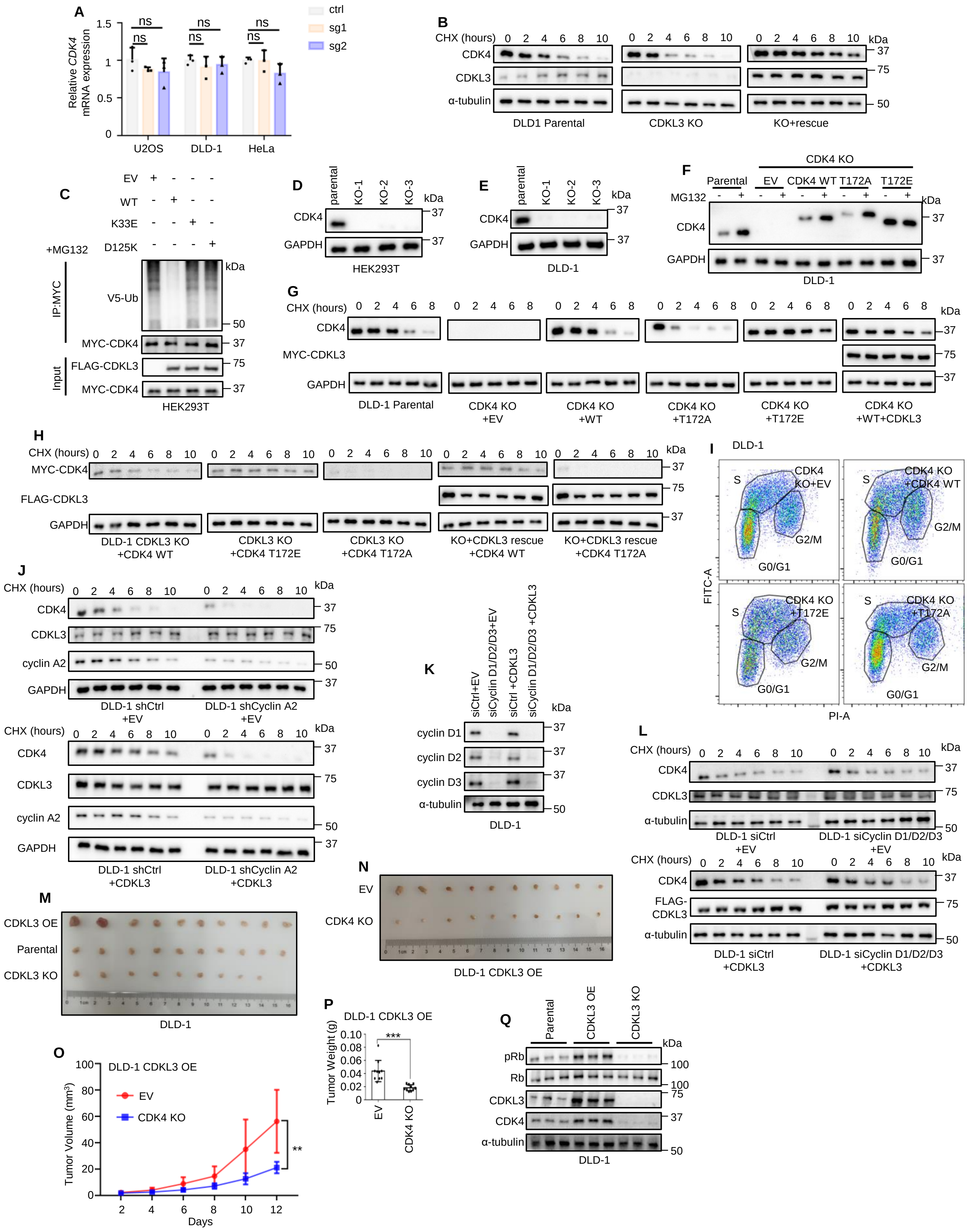

Supplemental Figure 3. Supporting information showing CDKL3 phosphorylates CDK4 on T172 to promote CDK4 stability via K48-linked poly-ubiquitination prevention.

(**A**) RT-qPCR showing CDKL3 knockout does not alter the transcription level of *CDK4* in U2OS, DLD-1 or HeLa cells. Error bar means ± SD, n=3, by one-way ANOVA. (**B**) The CHX-blocking assay showing CDKL3-loss reduced CDK4 protein stability. The quantification was shown in Figure 4B. (**C**) Ubiquitination assay of exogenous CDK4 showing the presence of WT CDKL3 reduced CDK4 ubiquitination instead of K33E/D125K mutant. MG132 was pretreated to maintain the same protein level. (**D, E**) Validation of CDK4 knockout by multiple sgRNAs by immunoblotting in HEK293T (**D**) and DLD-1 (**E**). CDK4 KO-1 in these cells were used and abbreviated as CDK4 KO in sequel experiments. (**F**) Immunoblotting assay showing MG132 treatment stabilizes CDK4 T172A in CDK4 KO and rescued DLD-1 cells. CDK4 T172E was insensitive to MG132 treatment. The rescue of CDK4 WT, T172A and T172E was approximately at the same level with the endogenous CDK4 in DLD-1 cells. (**G, H**) The CHX-blocking assay showing the protein stability of CDK4 WT and mutants under different circumstances. The quantifications were shown in Figure 5C and 5D, respectively. (**I**) Representative flow cytometry results of DLD-1 cells with BrdU-FITC/PI dual staining. The quantification was shown in Figure 5F. (**J**) The CHX-blocking assay showing the stabilization of CDK4 by CDKL3 was dependent on cyclin A2. The quantification was shown in Figure 5G. (**K**) Immunoblotting validation of cyclin D1/D2/D3 triple knockdown by siRNA. (**L**) The CHX-blocking assay showing the stabilization of CDK4 by CDKL3 was independent of cyclin D. The quantification was shown in Figure 5H. (**M**) Images of the tumors formed by subcutaneously transplanted DLD-1 cells after abscission. (**N**) Images of the tumors formed by subcutaneously transplanted CDKL3 OE DLD-1 cells after abscission. (**O, P**) Quantitative analyses of the tumor volume (**O**) and weight (**P**) of tumors formed by the subcutaneously transplanted CDKL3 OE DLD-1 cells. (**O**): Error bars mean ± SD, n=10, two-way ANOVA. (**P**): Error bar means ± SD, n=10, two-tailed Student's t-test. (**Q**) Immunoblotting of the key cell cycle related proteins (and phosphorylation) in the transplanted DLD-1 tumor tissues (n=3). Each lane represents an individual tissue. ns, not significant; **, $p<0.01$; ***, $p<0.001$.

Supplemental Figure 4

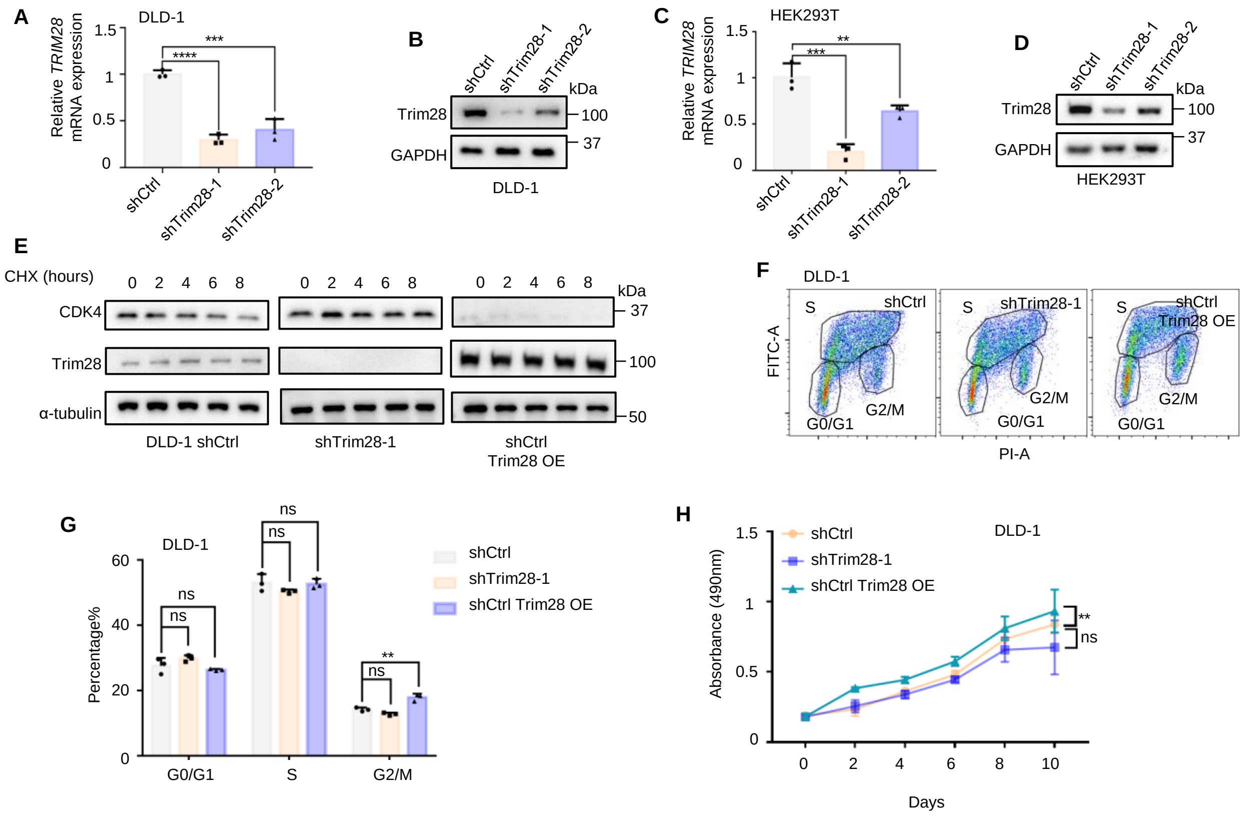

Supplemental Figure 4. Supporting information showing Trim28 ubiquitinates CDK4 for protein degradation in the absence of T172 phosphorylation.

(**A, B**) Validation of Trim28 knockdown by RT-qPCR (**A**) and immunoblotting (**B**) in DLD-1 cells. Error bar means ± SD, n=3, by one-way ANOVA. shTrim28-1 was used and abbreviated as shTrim28 in sequel experiments. (**C, D**) Validation of Trim28 knockdown by RT-qPCR (**C**) and immunoblotting (**D**) in HEK293T cells. Error bar means ± SD, n=3, by one-way ANOVA. shTrim28-1 was used and abbreviated as shTrim28 in sequel experiments. (**E**) CHX-blocking assay of endogenous CDK4 under Trim28 overexpression or knockdown conditions in DLD-1. The quantification was shown in Figure 6G. (**F**) Representative flow cytometry results of DLD-1 cells with BrdU-FITC/PI dual staining. (**G**) Statistical analysis of **F**. Error bar means ± SD, n=3, by one-way ANOVA. (**H**) MTT assay showing the growth of DLD-1 cells under Trim28 overexpression or depletion conditions. Error bars mean ± SD, n=3, by two-way ANOVA. ns, not significant; **, p<0.01; ***, p<0.001; ****, p<0.0001.

Supplemental Figure 5

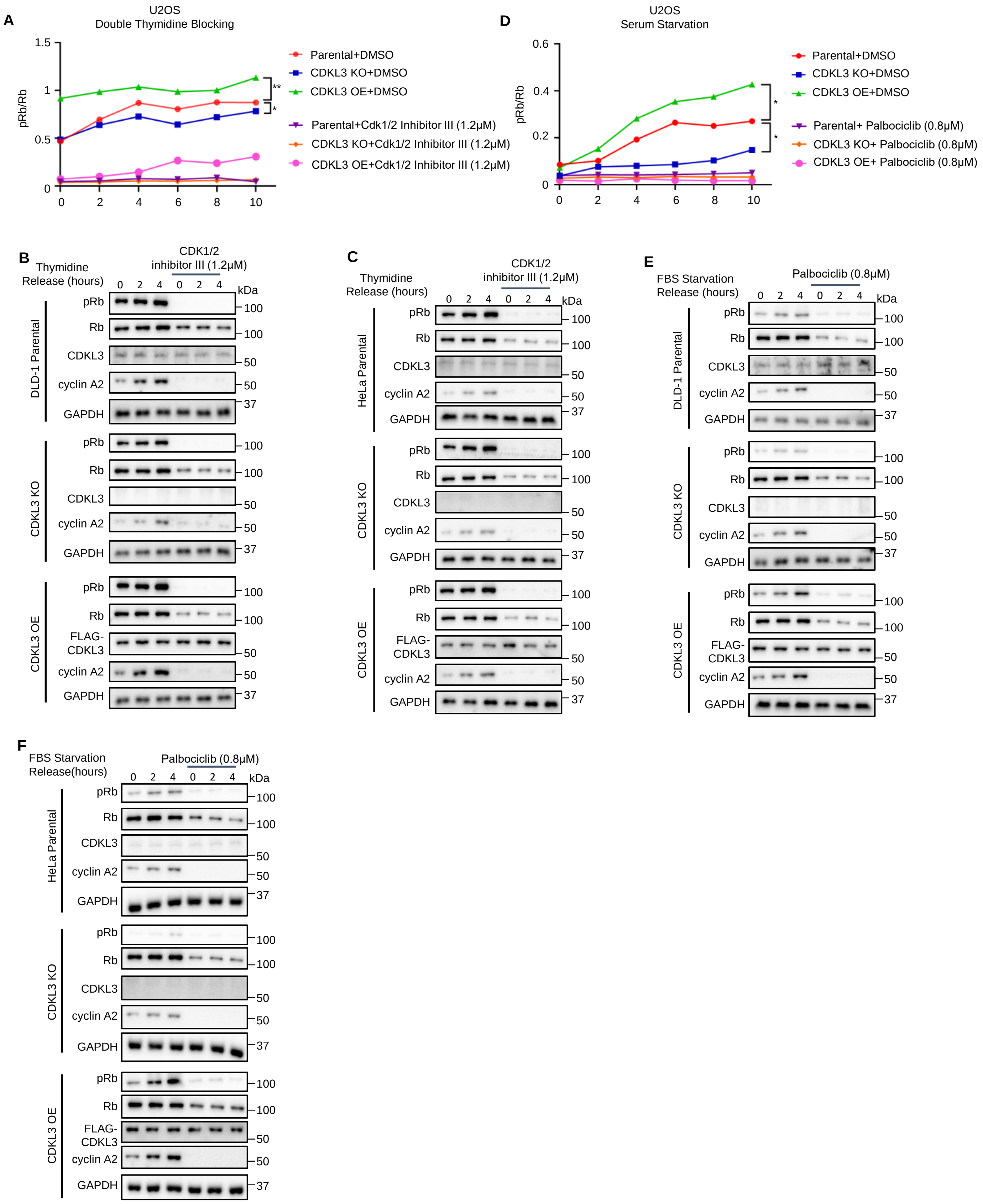

Supplemental Figure 5. Supporting information showing CDK inhibitors do not affect CDKL3 kinase activity.

(**A**) Statistical analysis of Rb phosphorylation under CDKL3 knockout/overexpression and CDK1/2 inhibitors conditions in U2OS cells with double thymidine blocking treatment in Figure 7C. By one-way ANOVA. (**B**) Immunoblotting of multiple cell cycle related proteins with double thymidine blocking and release and CDK1/2 inhibitor III treatment (1.2μM) under parental, CDKL3 knockout or overexpression conditions in DLD-1 cells. (**C**) Immunoblotting of multiple cell cycle related proteins with double thymidine blocking and release and CDK1/2 inhibitor III treatment (1.2μM) under parental, CDKL3 knockout or overexpression conditions in HeLa cells. (**D**) Statistical analysis of Rb phosphorylation under CDKL3 knockout/overexpression and Palbociclib conditions in U2OS cells with serum starvation treatment in Figure 7E. By one-way ANOVA. (**E**) Immunoblotting of multiple cell cycle related proteins after serum starvation and release and Palbociclib treatment (0.8μM) under parental, CDKL3 knockout or overexpression conditions in DLD-1 cells. (**F**) Immunoblotting of multiple cell cycle related proteins after serum starvation and release and Palbociclib treatment (0.8μM) under parental, CDKL3 knockout or overexpression conditions in HeLa cells. *, $p<0.05$; **, $p<0.01$.

Supplemental Figure 6

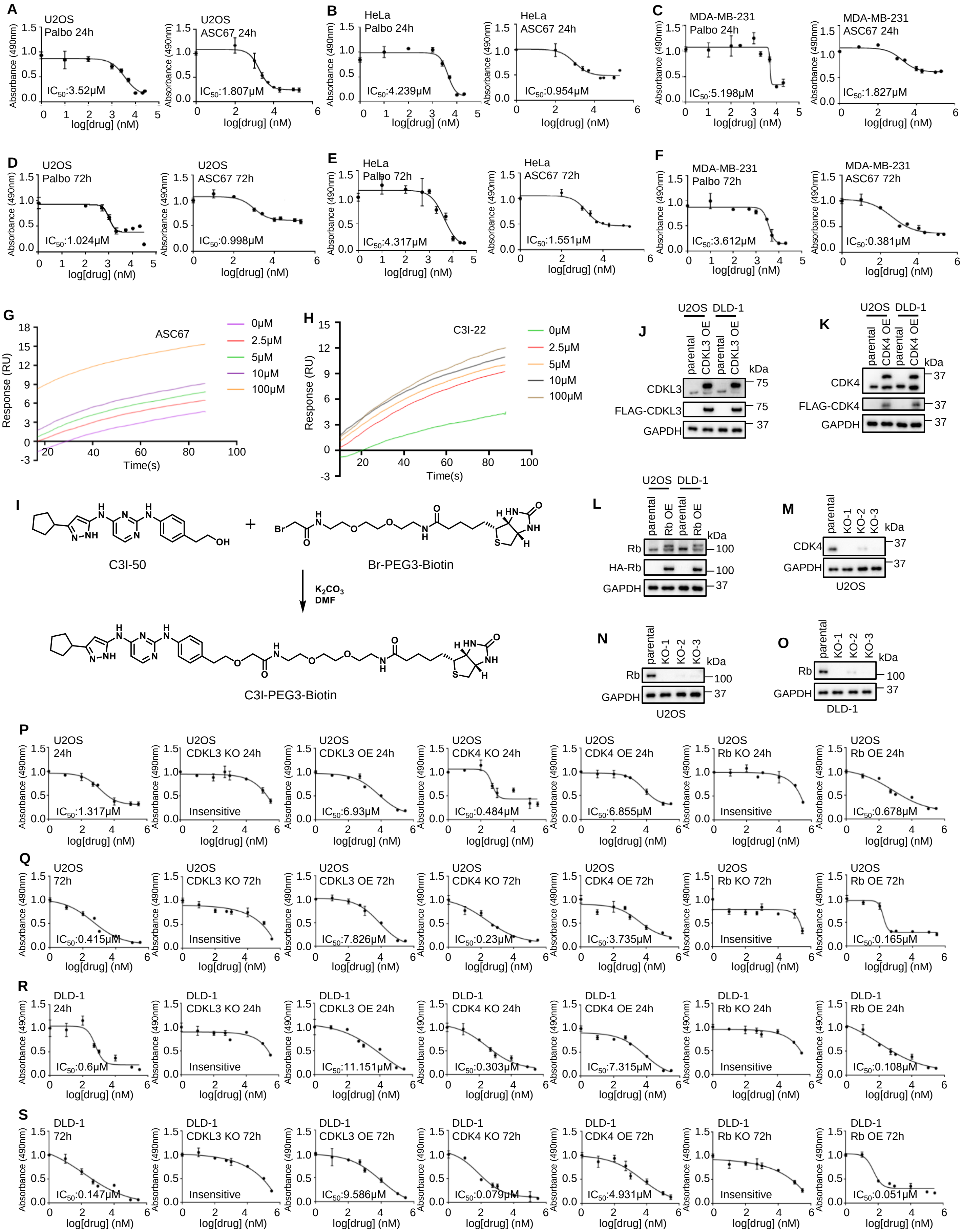

Supplemental Figure 6. Supporting information of design and characterization of CDKL3 inhibitor.

(**A-F**) Tumor-suppressing effects of Palbociclib and ASC67 under various conditions. A, U2OS, 24h; B, HeLa, 24h; C, MDA-MB-231, 24h; D, U2OS, 72h; E, HeLa, 72h; F, MDA-MB-231, 72h. (**G, H**) SPR sensorgrams of ASC67 (**G**) and C3I-22 (**H**) binding to CDKL3. (**I**) Synthesis route of the orthogonal PEG- and biotin-linked ASC67 derivative (C3I-PEG3-Biotin). (**J-L**) Immunoblotting validation of the overexpressed protein levels of CDKL3 (**J**), CDK4 (**K**) and Rb (**L**) in U2OS and DLD-1 cells. (**M-O**) Immunoblotting validation of CDK4 knockout in U2OS (**M**), Rb knockout in U2OS (**N**) and DLD-1 (**O**) by multiple sgRNAs. CDK4 or Rb KO-1 in these cells were used and abbreviated as CDK4 or Rb KO in sequel experiments, respectively. CDKL3 KO validation was shown in Supplemental Figure 1, E and F, for U2OS and DLD-1, respectively. CDK4 KO validation in DLD-1 was shown in Supplemental Figure 3E. (**P-S**) IC_{50} curves of C3I-22 in parental, CDKL3/CDK4/Rb overexpressed, CDKL3/CDK4/Rb KO cells, respectively. **P**: 24h treatment in U2OS; **Q**: 72h treatment in U2OS; **R**: 24h treatment in DLD-1; **S**: 72h treatment in DLD-1. These data are summarized in Table 1. All IC_{50} analysis was triplicated.

Supplemental Figure 7

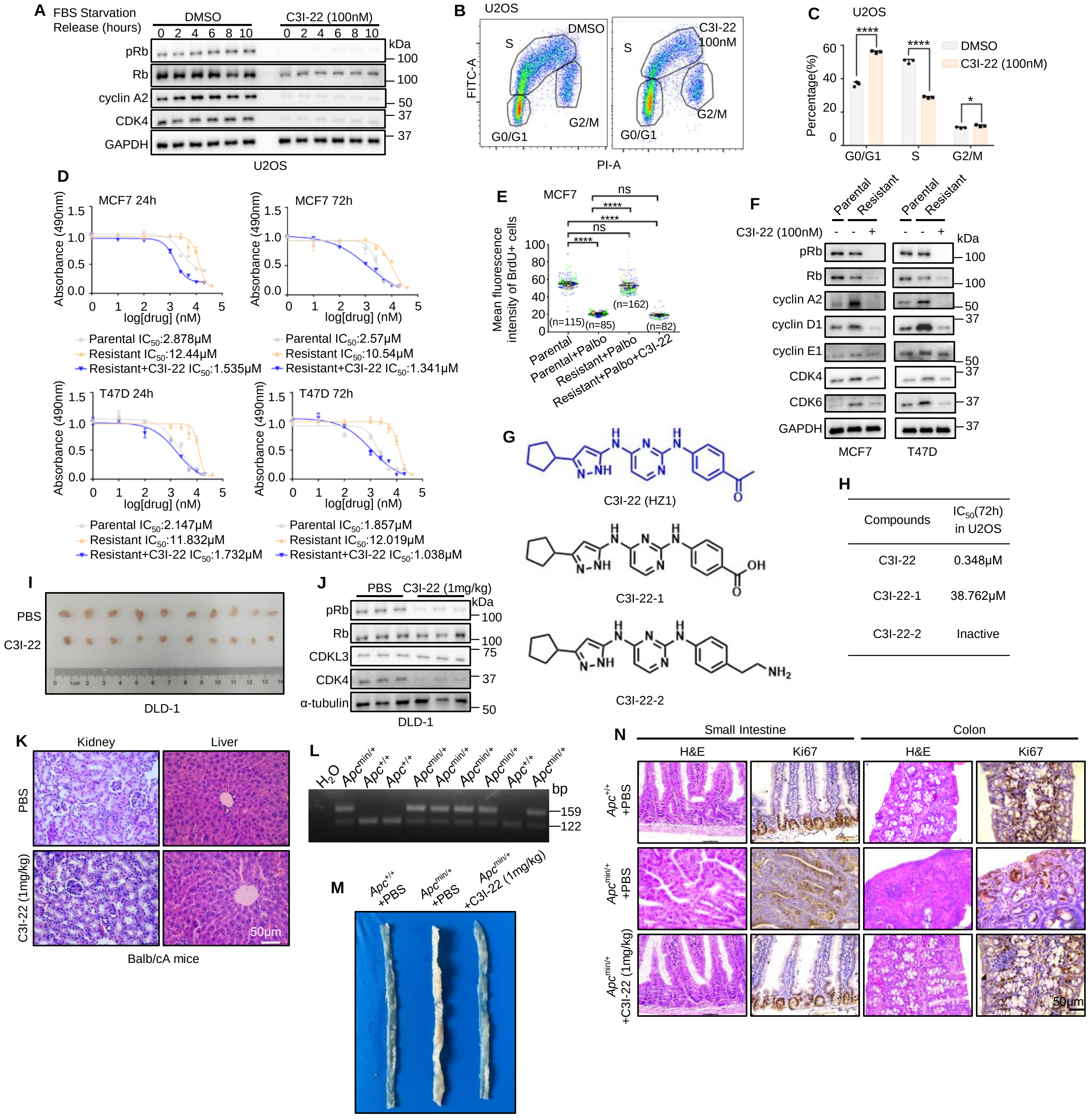

Supplemental Figure 7. Supporting assays showing C3I-22 (HZ1) antagonizes cancer growth via cell cycle arrest in multiple models.

(**A**) Immunoblotting of multiple cell cycle related proteins after serum starvation and release treat with C3I-22 (100nM) in U2OS cells. (**B**) Representative flow cytometry results of C3I-22 treatment (100nM) in U2OS cells with BrdU-FITC/PI dual staining. (**C**) Statistical analysis of **B**. Error bar means ± SD, n=3, by two-tailed Student's t-test. (**D**) IC_{50} curves of Palbociclib in parental/resistant MCF7 and T47D cells in Table 2. C3I-22 was treated at 100nM. (**E**) Superblot analysis of the number of BrdU-positive cells and mean BrdU intensity per cell in parental/resistant MCF7 cells under different conditions. Palbociclib: 800nM; C3I-22: 100nM. Error bar means ± SD, triplicated, by one-way ANOVA. n value in the panel represents the total number of cells. (**F**) Western blotting showing treatment of C3I-22 effectively reduced the endogenous pRb, cyclin A2, cyclin D1 and CDK4/6 levels. (**G**) Chemical structures of C3I-22, C3I-22-1 and C3I-22-2. (**H**) List of IC_{50} value of C3I-22-derived compounds in U2OS at 72h. (**I**) Images of the tumors formed by subcutaneously transplanted DLD-1 cells treat with CDKL3 inhibitor C3I-22 after abscission. (**J**) Immunoblotting of the key cell cycle related proteins (and phosphorylation) in the transplanted DLD-1 tumor tissues in **I**. Each lane represents an individual tumor tissue. (**K**) Representative HE staining images of kidney and liver of the C3I-22 treated nude mice. (**L**) Representative genotyping of *Apc*$^{+/+}$, *Apc*$^{min/+}$ and *Apc*$^{min/min}$ mice. (**M**) Representative intestine images of *Apc*$^{min/+}$ mice treat with PBS or C3I-22. (**N**) Representative HE and IHC staining images of small intestine and colon tumors treated with PBS or C3I-22 in *Apc*$^{min/+}$ mice. All images in the same panel are under the same amplification scales. All IC_{50} analysis was triplicated. ns, not significant; *, $p<0.05$; ****, $p<0.0001$.

Supplemental Figure 8

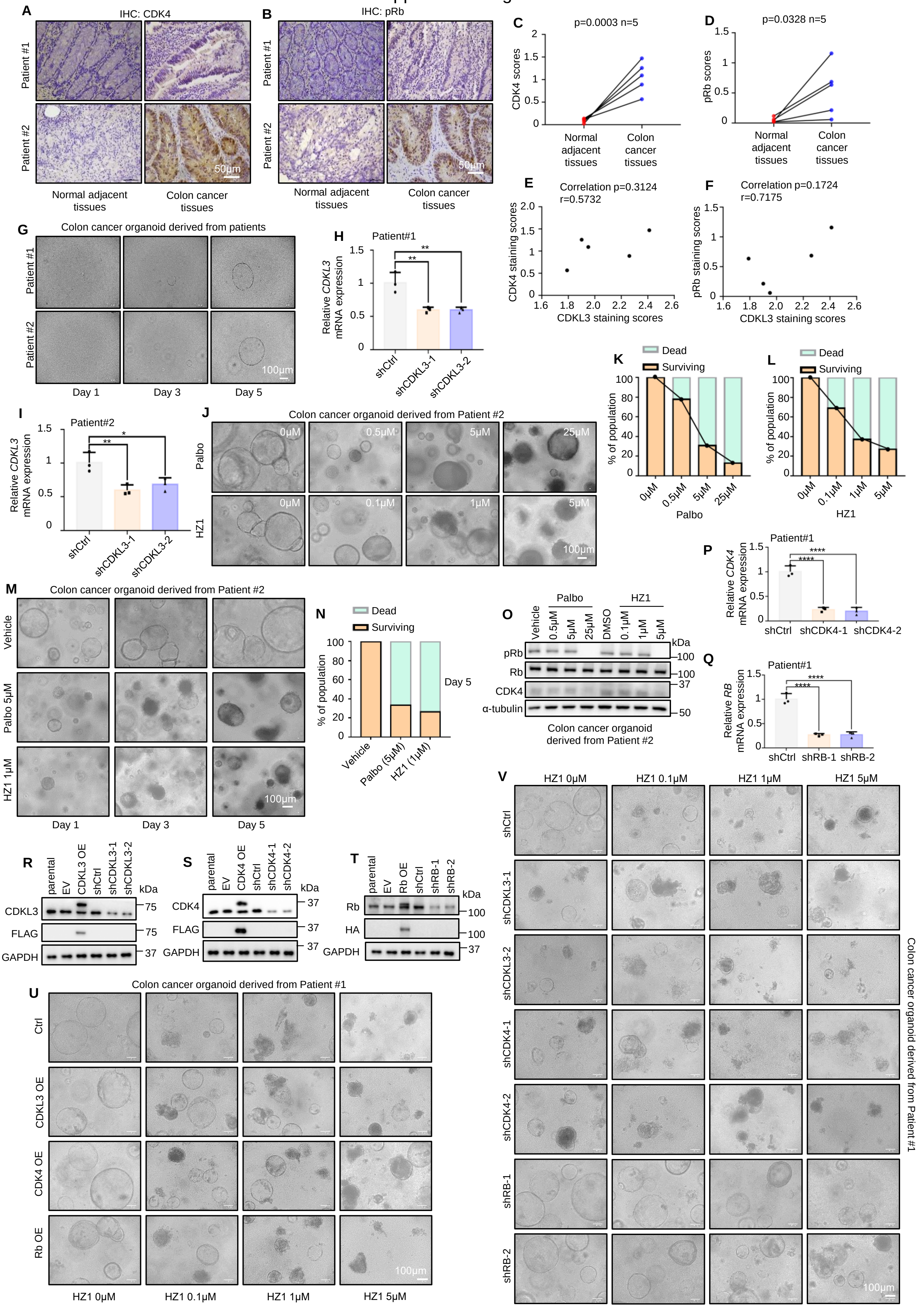

Supplemental Figure 8. Supporting information showing CDKL3 inhibitor has strong clinical implication in colon cancer treatment.

(**A, B**) Representative IHC staining images of CDK4 (**A**) and pRb (**B**) in colon cancer patients. (**C, D**) CDK4 (**C**) and pRb (**D**) immunohistochemical scores in colon cancer tissues and normal adjacent tissues. Error bar means ± SD, n=5, by two-tailed Student's t-test. (**E, F**) The correlations of CDKL3 with CDK4 (**E**) and pRb (**F**) in colon cancer patients. By two-tailed Spearman correlation analysis. (**G**) Representative images showing the establishment of patient-derived colon cancer organoids. (**H, I**) Validation of CDKL3 knockdown in the patient-derived organoids by RT-qPCR. Error bar means ± SD, n=3, by one-way ANOVA. (**J**) Representative images of patient-derived colon cancer organoids#2 under Palbociclib and HZ1 treatments. (**K, L**) Quantitative analysis of the surviving/dead organoids percentage under Palbociclib (**K**) and HZ1 (**L**) treatments in **J**. (**M**) Representative images of patient-derived colon cancer organoids#2 under Palbociclib and HZ1 treatments at different time points. (**N**) Quantitative analysis of the surviving/dead organoids percentage under Palbociclib and HZ1 treatment at Day 5. (**O**) Immunoblotting assay showing HZ1 decreased the levels of pRb and CDK4 in patient-derived colon cancer organoids#2. (**P, Q**) Validation of CDK4 (**P**) and Rb (**Q**) knockdown in the patient-derived organoid by RT-qPCR. Error bar means ± SD, n=3, by one-way ANOVA. (**R-T**) Immunoblotting validation of overexpression and knockdown of CDKL3 (**R**), CDK4 (**S**) and Rb (**T**) in the patient-derived organoid, respectively. (**U, V**) Representative images of patient-derived colon cancer organoids#1 under overexpression (**U**) or knockdown (**V**) condition of CDKL3/CDK4/Rb, respectively. All images in the same panel are under the same amplification scales. *, $p<0.05$; **, $p<0.01$; ****, $p<0.0001$.

Supplemental Table 1. General information of the colon cancer patients.

PATIENT	GENDER	AGE	PATHOLOGY	GRADE	USED IN ASSAYS
**#1**	Male	63	Adenocarcinoma	Moderately differentiated, MS-S	Organoid culture and IHC
**#2**	Male	66	Adenocarcinoma	Moderately differentiated, MS-S	Organoid culture and IHC
**#3**	Male	62	Adenocarcinoma	Moderately differentiated, MS-S	Organoid culture and IHC
**#4**	Female	64	Adenocarcinoma	Moderately-well differentiated, MS-S	IHC
**#5**	Female	62	Adenocarcinoma	Moderately differentiated, MS-S	IHC

Supplemental Table 2. Key resources table

**REAGENT or RESOURCE**	**Source**	**Identifier**
**Antibodies**		
CDKL3 Rabbit pAb	ABclonal	E16212 (customized)
Anti-mouse IgG, HRP-linked Antibody	Cell Signaling Technology	#7076
Anti-rabbit IgG, HRP-linked Antibody	Cell Signaling Technology	#7074
BrdU (Bu20a) Mouse mAb	Cell Signaling Technology	#5292
CDK4 (D9G3E) Rabbit mAb	Cell Signaling Technology	#12790
CDK4 Mouse mAb	Beyotime Biotechnology	AC251
CDK2 (78B2) Rabbit mAb	Cell Signaling Technology	#2546
CDK6 (D4S8S) Rabbit mAb	Cell Signaling Technology	#13331
CDK1 Rabbit mAb	Med Chem Express	HY-P80611
CDK3 Antibody (4B6)	Santa Cruz Biotechnology	sc-81836
p27 Kip1 Antibody (F-8)	Santa Cruz Biotechnology	sc-1641
HES1 Rabbit pAb	ABclonal	A11718
Cyclin A2 (BF683) Mouse mAb	Cell Signaling Technology	#4656T
Cyclin B1 Antibody	Cell Signaling Technology	#4138T
Cyclin D1 (92G2) Rabbit mAb	Cell Signaling Technology	#2978T
Cyclin D2 Polyclonal antibody	Proteintech	10934-1-AP
Cyclin D3 Rabbit pAb	ABclonal	A0746
Cyclin E1 (HE12) Mouse mAb	Cell Signaling Technology	#4129T
Cyclin G1 Polyclonal Antibody	Invitrogen	PA5-36050
Cyclin H Antibody	Cell Signaling Technology	#2927
DYKDDDDK Tag (D6W5B) Rabbit mAb	Cell Signaling Technology	#14793
HA-Tag (C29F4) Rabbit mAb	Cell Signaling Technology	#3724
KAP-1 (C42G12) Rabbit mAb	Cell Signaling Technology	#4124
Ki-67 (8D5) Mouse mAb	Cell Signaling Technology	#9449
MYC-Tag (9B11) Mouse mAb	Cell Signaling Technology	#2276
Phospho-Rb (Ser807/811) Antibody	Cell Signaling Technology	#9308
Rb (4H1) Mouse mAb	Cell Signaling Technology	#9309
Ubiquitin Antibody	Cell Signaling Technology	#3933
V5-Tag (D3H8Q) Rabbit mAb	Cell Signaling Technology	#13202
Goat anti-mouse IgG (H+L) Highly Cross-Adsorbed Secondary Antibody, Alexa Fluor™ 488	Invitrogen	A11029
Phospho-CDK4-T172 Rabbit pAb	ABclonal	AP0593

Alpha Tubulin Polyclonal antibody	Proteintech	11224-1-AP
GAPDH Polyclonal antibody	Proteintech	10494-1-AP
GST(B-14) Mouse mAb	Santa Cruz Biotechnology	sc-138
**Bacterial strain**		
E. coli NEB® 5-alpha	New England Biolabs (NEB)	C2987H
E. coli BL21	New England Biolabs (NEB)	C2530H
**Commercial chemicals and biologics/Chemicals**		
4% paraformaldehyde	Beyotime Biotechnology	P0099
MG132	Beyotime Biotechnology	S1748
Isopropyl-β-D-thiogalactoside (IPTG)	Beyotime Biotechnology	ST098
Penicillin-Streptomycin-Glutamine (100X)	Gibco	10378016
Puromycin	Invivogen	ant-pr-1
Neofect™ DNA transfection reagent	Neofect	ME201901
Ampicillin	Sangon Biotech	A100339
Crystal violet	Sangon Biotech	A100528
Glycerol	Sangon Biotech	A100854
Glycine	Sangon Biotech	A110167
Phosphate buffer	Sangon Biotech	A610100-0001
Sodium dodecyl sulfate	Sangon Biotech	A600485-0500
Polybrene	Santa Cruz Biotechnology	SC-134220
Palbociclib	Selleckchem	S4482
Cycloheximide	Sigma-Aldrich	239763-M
Cdk1/2 Inhibitor III	Sigma-Aldrich	217714
IGEPAL® CA-630	Sigma-Aldrich	I3021
Pyronin Y	Sigma-Aldrich	P9172
Thiazolyl Blue Tetrazolium Bromide	Sigma-Aldrich	M5655
Thymidine	Sigma-Aldrich	T1895
Tris[(1-benzyl-1H-1,2,3-triazol-4-yl) methyl] amine	Sigma-Aldrich	678937
Triton™ X-100	Sigma-Aldrich	X100
Hoechst	Thermo	1990363
ATP	Solarbio	A8270
Advanced DMEM/F-12	Gibco	12634028
B-27™ Supplement (50X), minus vitamin A	Gibco	12587010

N-2 Supplement (100X)	Gibco	17502048
EGF Recombinant Mouse Protein	Gibco	PMG8041
N-Acetyl-L-cysteine	Sigma-Aldrich	A9165
Nicotinamide	Sigma-Aldrich	N0636
Y-27632	Sigma-Aldrich	Y0503
HEPES	Beyotime	ST092
Recombinant Murine Noggin	Peprotech	250-38
Corning® Matrigel® Growth Factor Reduced (GFR) Basement Membrane Matrix, Phenol Red-free, *LDEV-free	Corning	356231
2,4-dichloropyrimidine	Sigma-Aldrich	143847
DIPEA	Sigma-Aldrich	800894
3-cyclopentyl-1*H*-pyrazol-5-aminetriethylamine	Aladdin	C175937
CH_2Cl_2	Sigma-Aldrich	40042
**Critical commercial assays/kits**		
BCA Protein Assay Kit	Thermo	23227
Endo-free Plasmid Mini Kit	Omega	D6950
UNIQ-10 Column Trizol Total RNA Isolation Kit	Sangon Biotech	B511321
MonScript™ RTIII All-in-One Mix with dsDNase	Monad	MR05101M
MonAmp™ ChemoHS qPCR Mix	Monad	MQ00401S
GeneJET Gel Extraction Kit	Thermo	K0692
GeneJET PCR Purification Kit	Thermo	K0702
UltraSensitiveTM SP (Mouse/Rabbit) IHC Kit	MXB Biotechnologles	KIT-9720
DAB Kit (20×)	MXB Biotechnologles	DAB-0031
Hematoxylin-Eosin (HE) staining Kit	BBI LIFE	E607318
ChemistarTM High-sig ECL Western Blotting Substrate	Tanon	180-5001
RIPA buffer	Cowin Bio	CW2333
GST Fusion Protein Purification Kit	Genscript	L00206
Ubiquitination Kit	Enzo Life Science	BML-UW9920-0001
CALNP™ RNAi in vitro	D-Nano Therapeutics	DN001- 01
**Experimental models: Cell**		

**lines**		
HEK293T	ATCC	CRL-11268 RRID:CVCL_1926
DLD-1	NCACC, China	TCHu134
U2OS	ATCC	HTB-96
HeLa	ATCC	CCL-2
MCF-7	ATCC	HTB-22
MDA-MB-231	ATCC	HTB-26
T47D	ATCC	HTB-133
**Experimental models: Animals**		
BALB/cA-nude mice	Beijing HFK Bioscience CO.,LTD	13001A
C57BL/6J-*Apc*$^{min/+}$ mice	GemPharmatech (Nanjing, China)	T001457
C57BL/6J mice	GemPharmatech (Nanjing, China)	11001A
**Oligonucleotides**		
Control-shRNA sequence	This study	AAAAAAAAAAAAAAAAAAAAA
#1-shCyclin A2 sequence (Human)	This study	ATACTTGAGGTATGGGTCAGC
#2-shCyclin A2 sequence (Human)	This study	TTAACCTCCATTTCCCTAAGG
#1-shCyclin E1 sequence (Human)	This study	ATGACGAGAAATGATACAAGG
#2-shCyclin E1 sequence (Human)	This study	TAACCAATCCAGAAGAATTGC
#1-shTrim28 sequence (Human)	This study	CCTGGCTCTGTTCTCTGTCCT
#2-shTrim28 sequence (Human)	This study	CTGAGACCAAACCTGTGCTTA
#1-shCDKL3 sequence (Human)	This study	TTGGTTCTGAGATATCTCCTC
#2-shCDKL3 sequence (Human)	This study	GTTAGTTAGATTGATGGGTGG
#1-shRB sequence (Human)	This study	TTTGGACTAGAAATAATGTGG
#2-shRB sequence (Human)	This study	TTGCAGTAGAATTTACACGCG
#1-RB sgRNA sequence (Human)	This study	GTTCTTTGAGCAACATGGG
#2-RB sgRNA sequence	This study	GAACTACTTACGA

(Human)		ACTGCT
#3-RB sgRNA sequence (Human)	This study	ACATCTAATGGACTTCCAG
#1-CDKL3 sgRNA sequence (Human)	This study	GGGCTGTATGATCATTGAGA
#2-CDKL3 sgRNA sequence (Human)	This study	CAAAATCAAGAAAAACAAG
#3-CDKL3 sgRNA sequence (Human)	This study	TCAATTTGTAAACAAGCCT
#1-CDK4 sgRNA sequence (Human)	This study	GTCCACATATGCAACACCTG
#2-CDK4 sgRNA sequence (Human)	This study	TCTACATGCTCAAACACCA
#3-CDK4 sgRNA sequence (Human)	This study	CAGTGGCTGAAATTGGTGT
Human *GAPDH*-RT-qPCR primers	This study	F:GGAGCGAGATCCCTCCAAAT R:GGCTGTTGTCATACTTCTCAG
Human *CDK4*-RT-qPCR primers	This study	F:ATGGCTACCTCTCGATATGAGC R:CATTGGGGACTCTCACACTCT
Human *CCNA2*-RT-qPCR primers	This study	F:CGCTGGCGGTACTGAAGTC R:GAGGAACGGTGACATGCTCAT
Human *CCNE1*-RT-qPCR primers	This study	F:GCCAGCCTTGGGACAATAATG R:CTTGCACGTTGAGTTTGGGT
Human *TRIM28*-RT-qPCR primers	This study	F:TTTCATGCGTGATAGTGGCAG R:GCCTCTACACAGGTCTCACAC
Human *CDKN2B*-RT-qPCR primers	This study	F:TGGGGTGGGAAAGTGGATTGCA R:CCCAGTGCAGAGGTGTTCAGGTCT
Human *CDKN2A*-RT-qPCR primers	This study	F:GCTGCTCACCTCTGGTGCCAAA R:ACCTGCGCACCATGTTCTCG

Human *CDKN1B*-RT-qPCR primers	This study	F:GGTTAGCGGAGCAATGCGCA R:AACCGGCATTTGGGGAACCGTC
Human *HES1*-RT-qPCR primers	This study	F:CCCAGCCAGTGTCAACACGACA R:GGTACCGCCCTTACCTTTCTGTGC
Human *CCND1*-RT-qPCR primers	This study	F:GCTGCGAAGTGGAAACCATC R:CCTCCTTCTGCACACATTTGAA
Mouse Genotyping *Apc*-PCR primers	This study	F:ATACTACGGTATTGCCCAGC R:TGTTGTTGGATGGTAAGCAC
Control-siRNA	GENERAL BIOL	F:UUCUCCGAACGUGUCACGUTT R:ACGUGACACGUUCGGAGAATT
CCND1 (human) siRNA	GENERAL BIOL	F:AGGAAGAGGAGGAGGAGGATT R:UCCUCCUCCUCCUCUUCCUTT
CCND2 (human) siRNA	GENERAL BIOL	F:CCUACAGACGUGCGGGAUATT R:UAUCCCGCACGUCUGUAGGTT
CCND3 (human) siRNA	GENERAL BIOL	F:CUACAGAUGUCACAGCCAUTT R:AUGGCUGUGACAUCUGUAGTT
**Recombinant DNA**		
Lenti-EF1α-puro V5-Ub	This study	
Lenti-EF1α-puro FLAG-CDKL3	This study	
Lenti-EF1α-puro MYC-CDKL3	This study	
Lenti-EF1α-puro GFP-CDKL3	This study	
Lenti-EF1α-puro tandem GFPs	This study	
Lenti-EF1α-puro MYC-cyclin A2	This study	
Lenti-EF1α-puro MYC-cyclin B1	This study	
Lenti-EF1α-puro MYC-cyclin D1	This study	
Lenti-EF1α-puro MYC-cyclin E1	This study	
Lenti-EF1α-puro MYC-cyclin G1	This study	

Lenti-EF1α-puro MYC-cyclin H1	This study	
pGEX4T-1-Rb 379-928	This study	
pGEX4T-1-Rb 792-928	This study	
Lenti-EF1α-puro FLAG-CDK4	This study	
Lenti-EF1α-puro MYC-CDK4	This study	
Lenti-EF1α-puro FLAG-CDK6	This study	
Lenti-EF1α-puro FLAG-CDK7	This study	
pGEX4T-1-CDK4	This study	
Lenti-EF1α-puro HA-UBE3A	This study	
Lenti-EF1α-puro HA-Stub1	This study	
Lenti-EF1α-puro HA-Trim28	This study	
pET28a-Trim28	This study	
pGEX4T-1-CDKL3	This study	
Lenti-EF1α-puro FLAG-CDK2	This study	
Lenti-EF1α-puro MYC-p16	This study	
Lenti-EF1α-puro MYC-p21	This study	
Lenti-EF1α-puro MYC-p27	This study	
Lenti-EF1α-puro FLAG-CDK1	This study	
Lenti-EF1α-puro FLAG-CDK3	This study	
Lenti-EF1α-puro MYC-cyclin C	This study	
Lenti-EF1α-Blast FLAG-CDKL3	This study	
Lenti-EF1α-Blast FLAG-CDK4	This study	
Lenti-EF1α-Blast MYC-CDK4	This study	
pGEX4T-1-CDK4-D158N	This study	
pGEX4T-1-CDKL3 Kinase domain (K)	This study	
pGEX4T-1-CDK2 Kinase domain (K)	This study	
pET28a-CyclinA2	This study	
psPAX2 vector	Addgene	#12260
pCMV-VSV-G	Addgene	#8454
Lenti-Cas9-puro	Addgene	#108100
Lenti-Cas9-puro sgCDKL3#1	This study	
Lenti-Cas9-puro sgCDKL3#2	This study	
Lenti-Cas9-puro sgCDKL3#3	This study	
Lenti-Cas9-puro sgCDK4#1	This study	
Lenti-Cas9-puro sgCDK4#2	This study	
Lenti-Cas9-puro sgCDK4#3	This study	
pLKO-puro shCyclin A2#1	This study	
pLKO-puro shCyclin A2#2	This study	
pLKO-puro shCyclin E1#1	This study	
pLKO-puro shCyclin E1#2	This study	
pLKO-puro shTrim28#1	This study	

pLKO-puro shTrim28#2	This study	
Lenti-Cas9-puro sgRB#1	This study	
Lenti-Cas9-puro sgRB#2	This study	
Lenti-Cas9-puro sgRB#3	This study	
pLKO-puro shRB#1	This study	
pLKO-puro shRB#2	This study	
**Software and algorithms**		
Graphpad Prism 8	Graphpad software	https://www.graphpad.com
ImageJ		https://imagej.nih.gov/ij/
FlowJo version 10.0.7r2	BD Biosciences	https://www.flowjo.com
**Other**		
PVDF	Millipore	IPVH00010
Protein G Plus-Agarose	Santa Cruz Biotechnology	SC-2002
α-MYC Agarose Affinity Gel	Sigma-Aldrich	7470
α-FLAG Agarose Affinity Gel	Sigma-Aldrich	4596
α-HA Agarose Affinity Gel	Sigma-Aldrich	A2095
EQKLISEEDL (MYC-tag) peptide	Sangon Biotech	Custom synthesis
DYKDDDDK (FLAG-tag) peptide	Sangon Biotech	T510060-0005
High Affinity Ni-NTA Resin	Genscript	L00250
Glutathione Resin	Genscript	L00206
Streptavidin Resin	Pierce	20349

References

1. Geng Y, Yu Q, Sicinska E, Das M, Schneider JE, Bhattacharya S, et al. Cyclin E ablation in the mouse. *Cell.* 2003;114(4):431-43.
2. Kalaszczynska I, Geng Y, Iino T, Mizuno SI, Choi Y, Kondratiuk I, et al. Cyclin A Is Redundant in Fibroblasts but Essential in Hematopoietic and Embryonic Stem Cells. *Cell.* 2009;138(2):352-65.
3. Liu L, Michowski W, Inuzuka H, Shimizu K, Nihira NT, Chick JM, et al. G1 cyclins link proliferation, pluripotency and differentiation of embryonic stem cells. *Nature cell biology.* 2017;19(3):177-88.
4. Darzynkiewicz Z, Sharpless T, Staiano-Coico L, and Melamed MR. Subcompartments of the G1 phase of cell cycle detected by flow cytometry. *Proceedings of the National Academy of Sciences of the United States of America.* 1980;77(11):6696-9.
5. Crissman HA, Darzynkiewicz Z, Tobey RA, and Steinkamp JA. Correlated measurements of DNA, RNA, and protein in individual cells by flow cytometry. *Science.* 1985;228(4705):1321-4.
6. Darzynkiewicz Z. Simultaneous analysis of cellular RNA and DNA content. *Methods Cell Biol.* 1994;41:401-20.
7. van de Wetering M, Francies HE, Francis JM, Bounova G, Iorio F, Pronk A, et al. Prospective derivation of a living organoid biobank of colorectal cancer patients. *Cell.* 2015;161(4):933-45.
