# Supplementary material for: CDKL3 is a targetable regulator of cell cycle progression in cancers
Source: J Clin Invest. 2024 Jul 4;134(16):e178428. doi: 10.1172/JCI178428 (PMC11324297; doi:10.1172/JCI178428)

Full unedited blot for Figure 2A

pRb

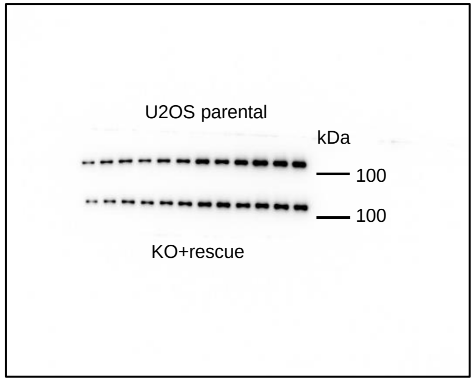

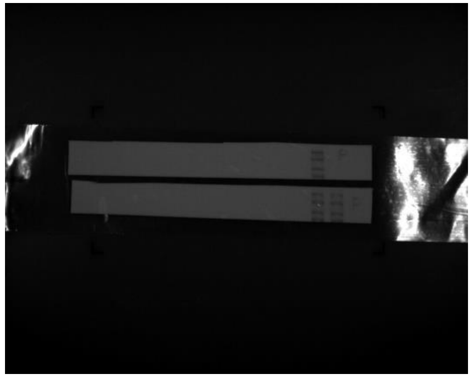

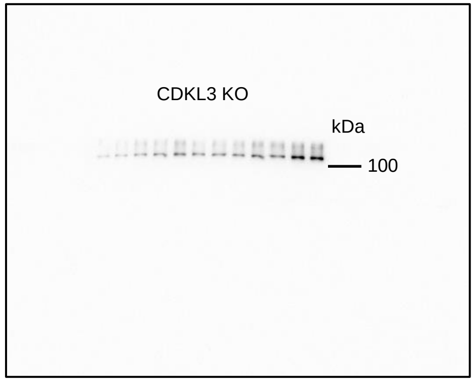

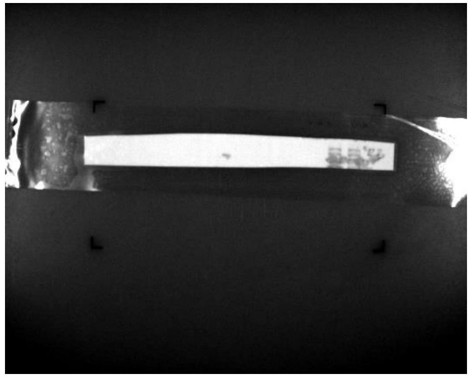

Rb

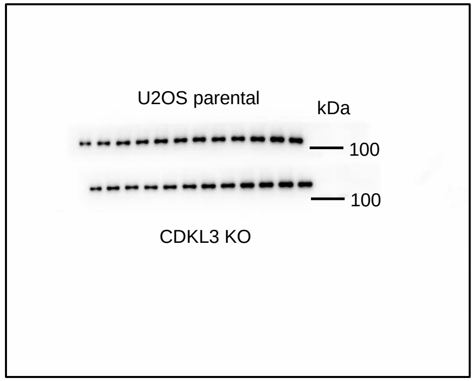

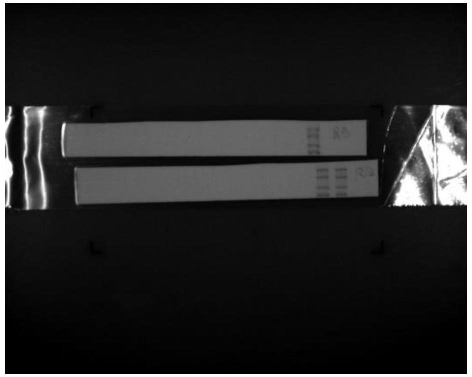

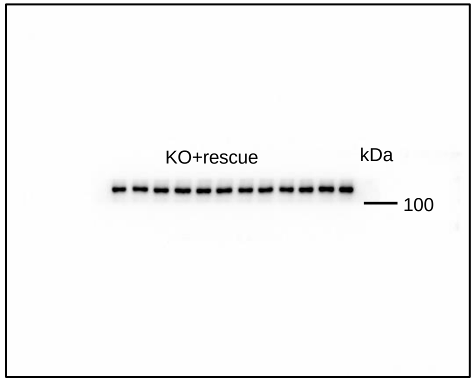

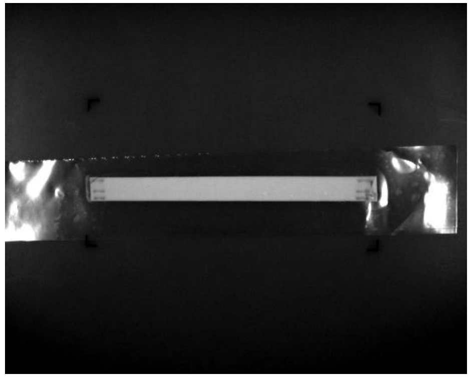

Full unedited blot for Figure 2A

cyclinA2

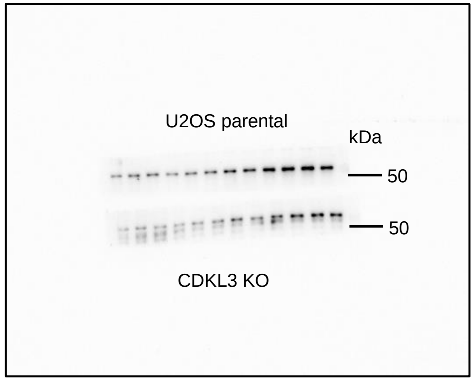

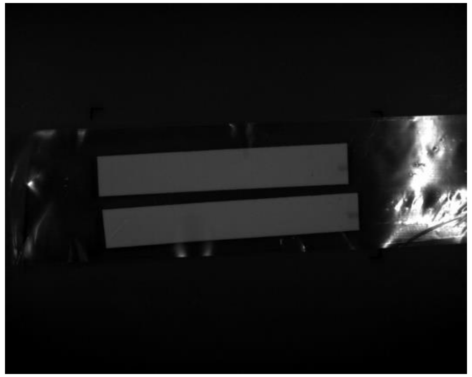

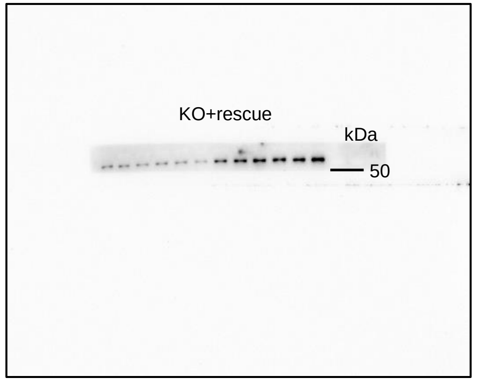

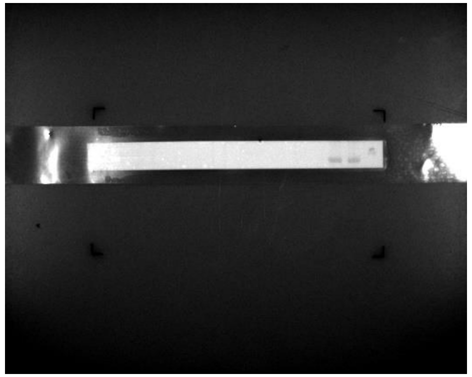

Full unedited blot for Figure 2A

cyclinD1

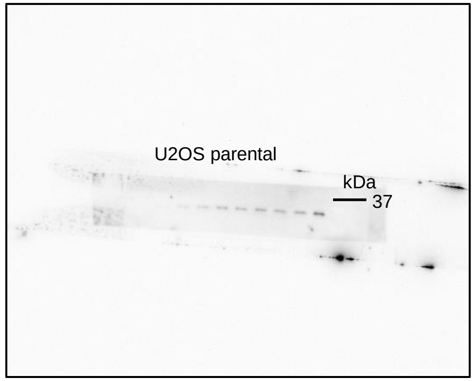

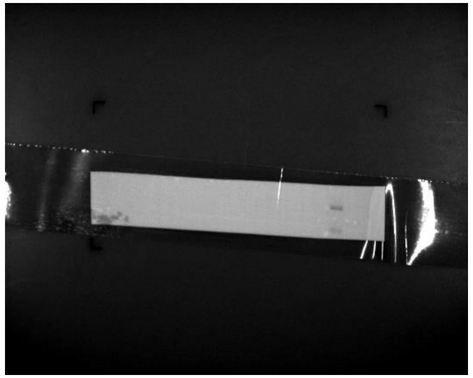

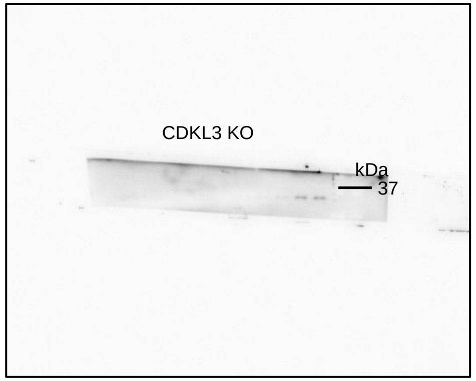

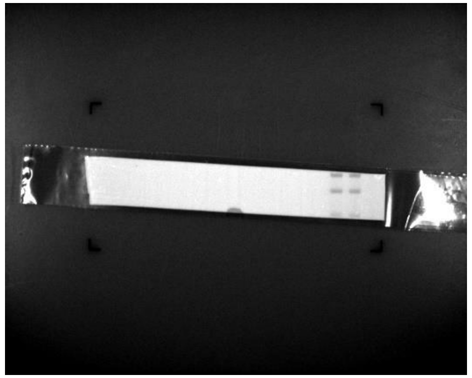

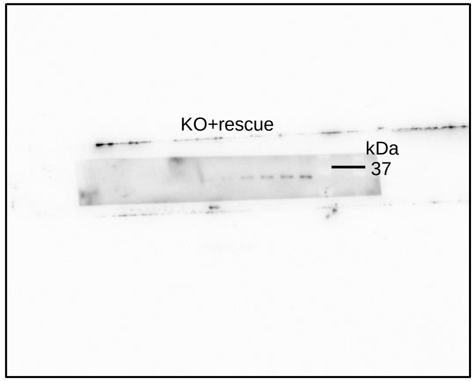

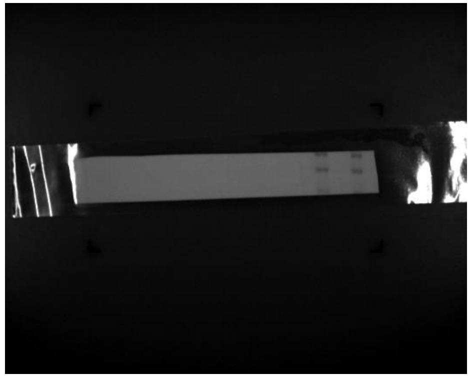

Full unedited blot for Figure 2A

cyclinE1

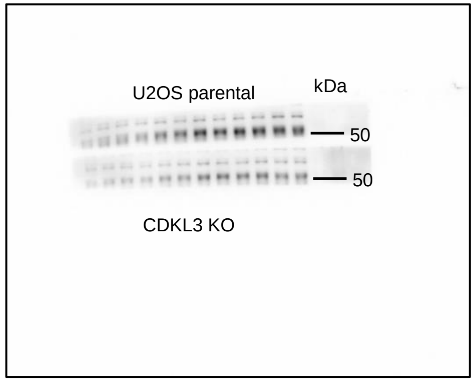

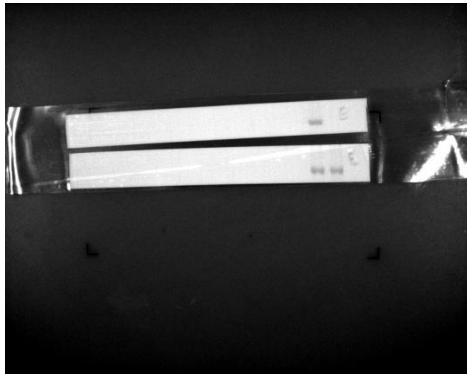

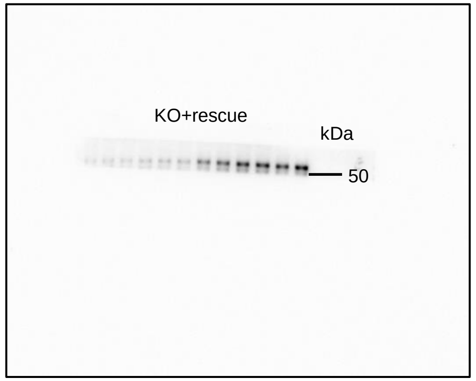

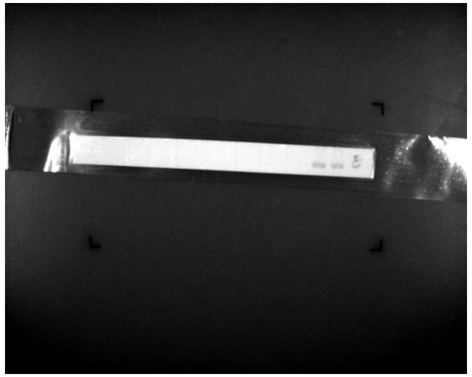

GAPDH

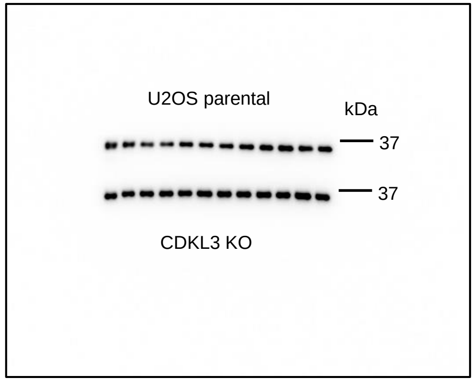

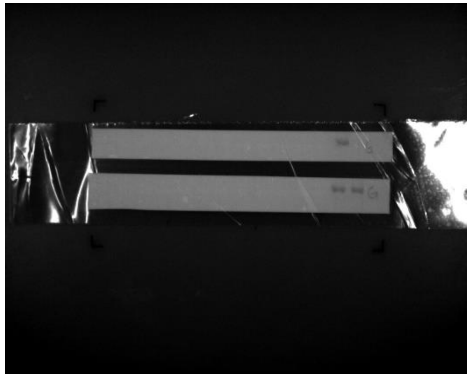

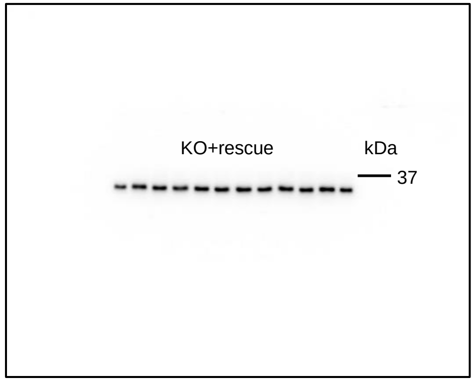

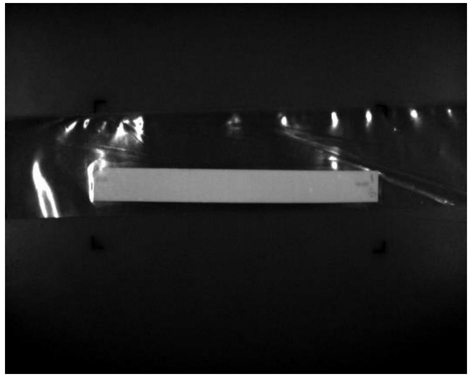

Full unedited blot for Figure 2E

CDKL3

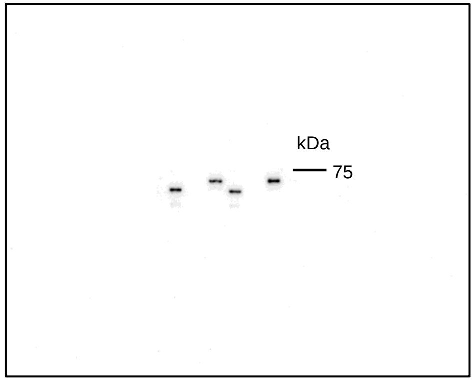

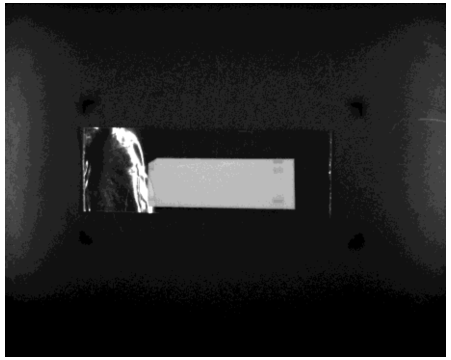

HES1

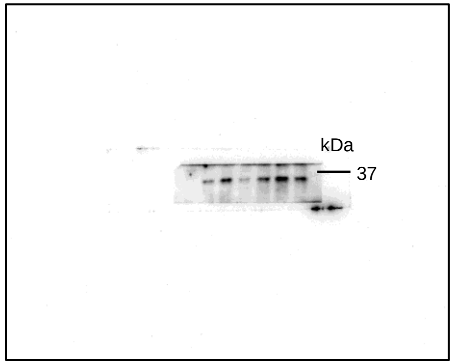

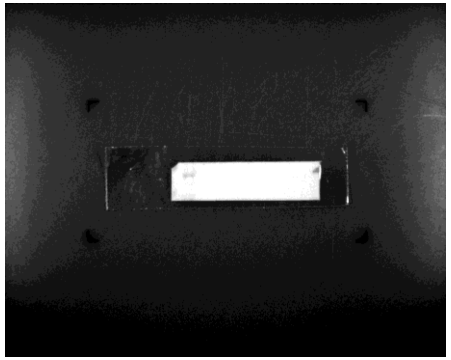

p27

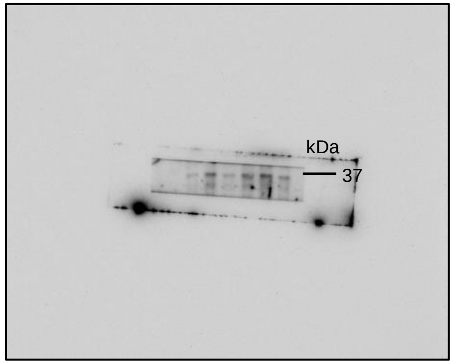

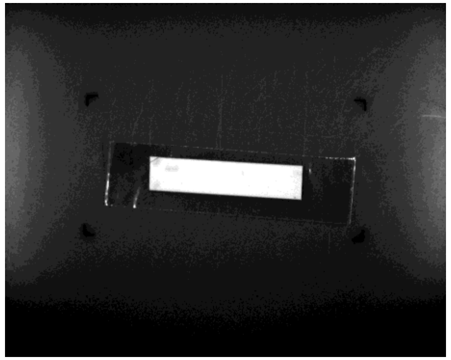

GAPDH

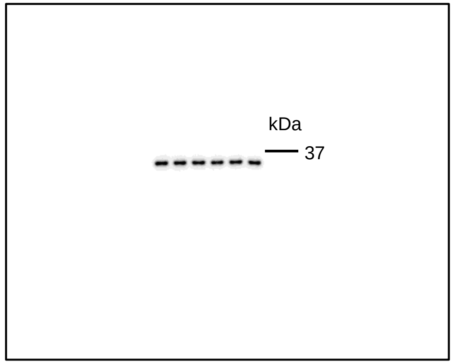

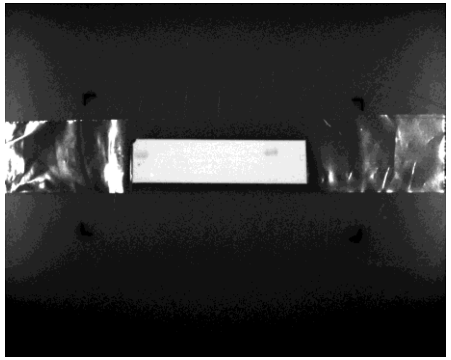

Full unedited blot for Figure 3A

FLAG-CDKL3

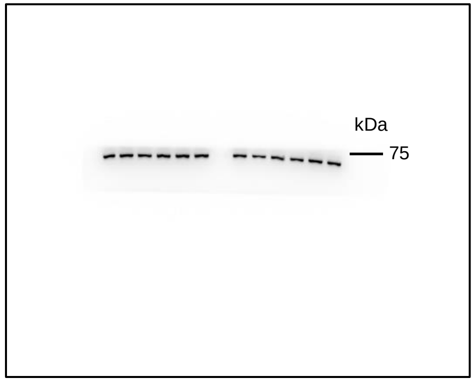

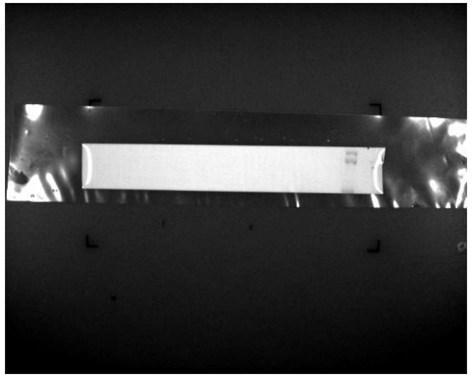

MYC-cyclin

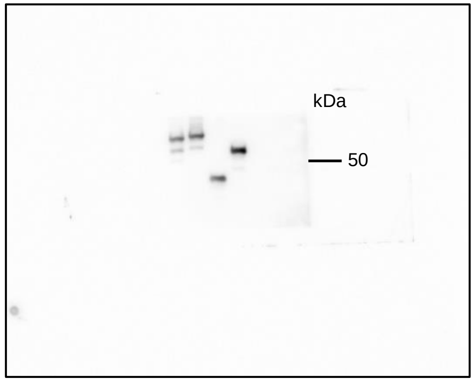

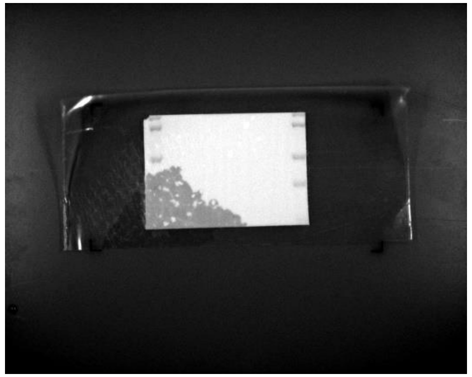

MYC-cyclin

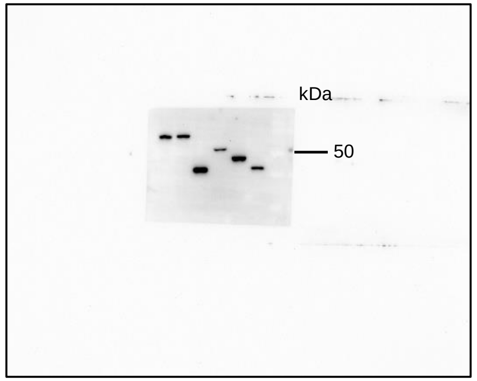

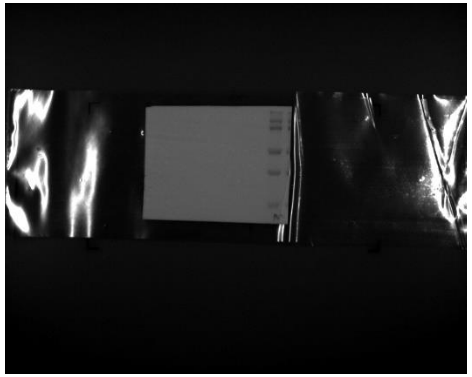

Full unedited blot for Figure 3B

Rb

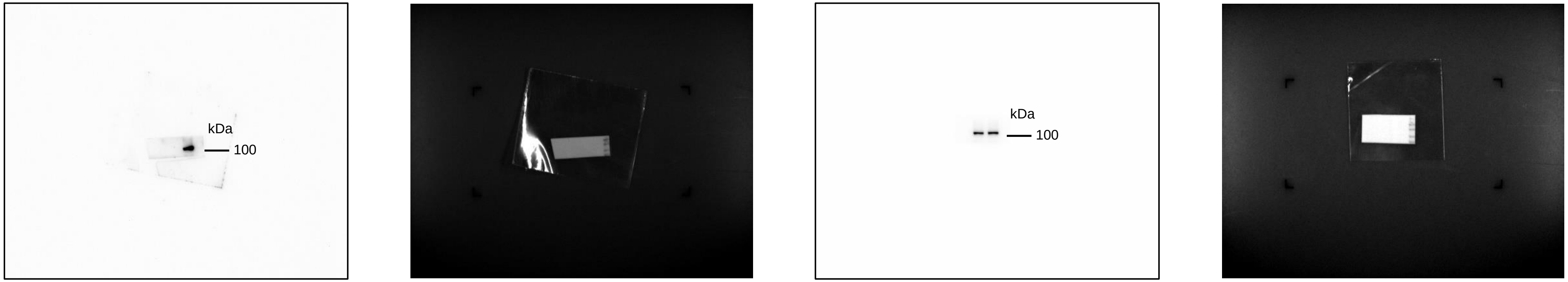

cyclinA2

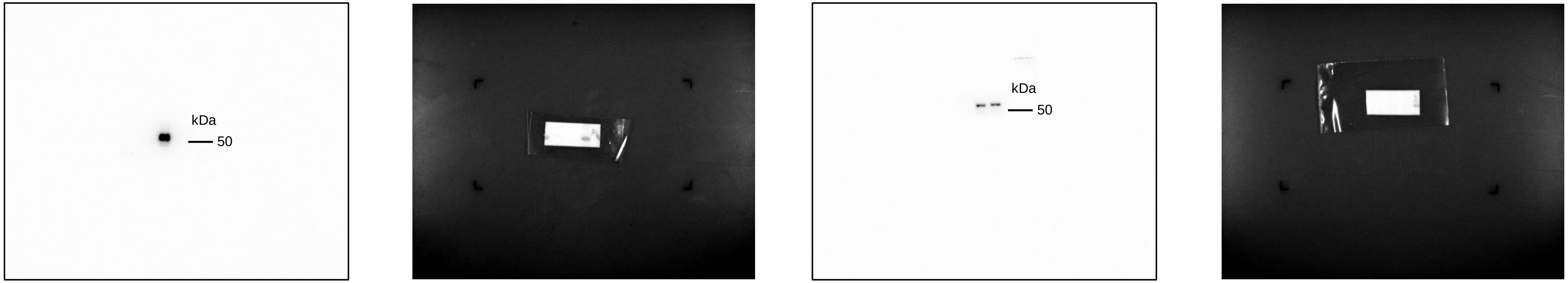

Full unedited blot for Figure 3B

cyclinB1

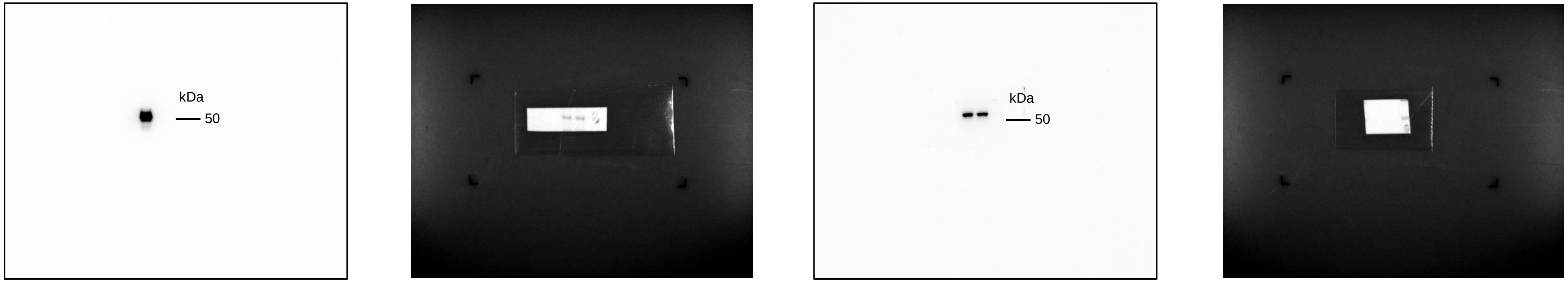

cyclinD1

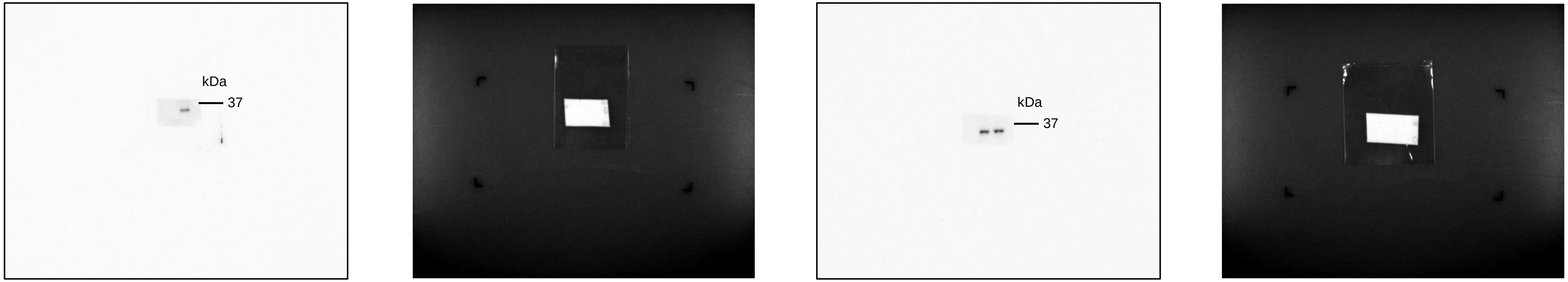

Full unedited blot for Figure 3B

cyclinE1

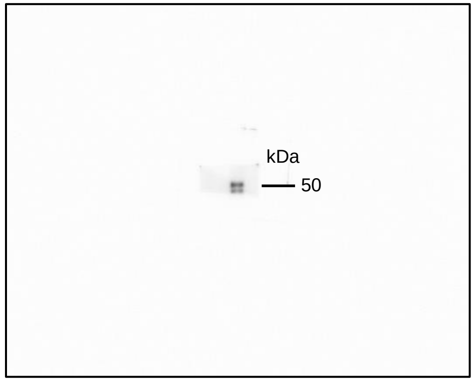

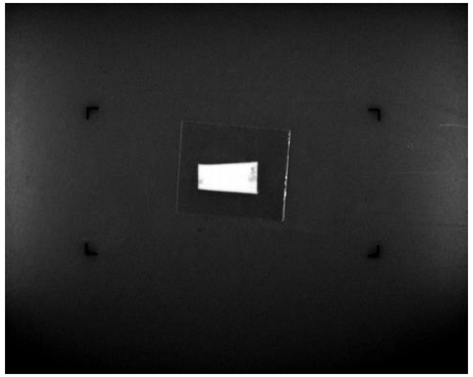

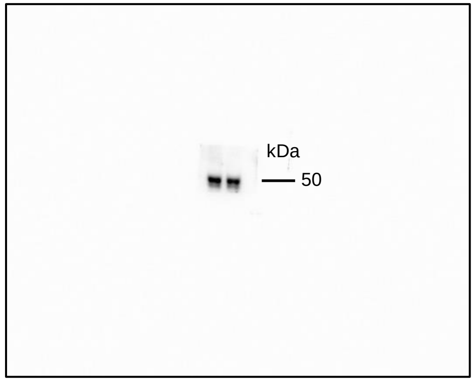

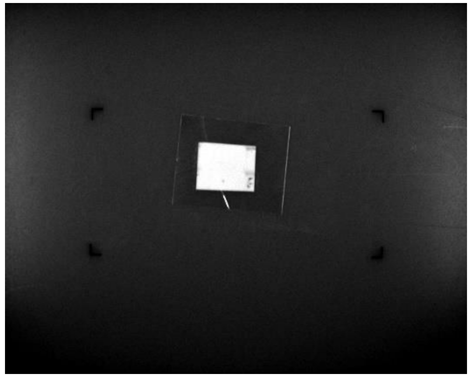

cyclinG1

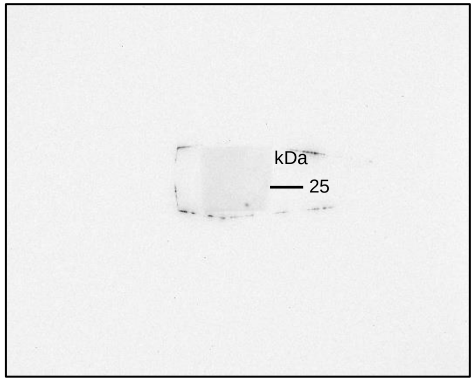

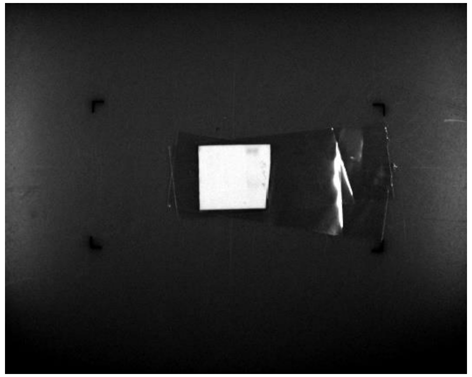

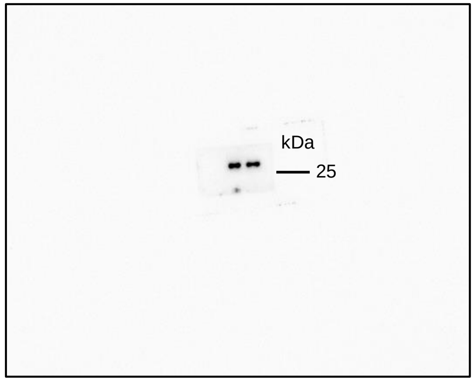

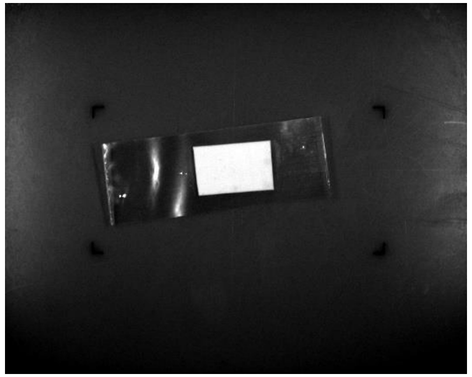

Full unedited blot for Figure 3B

cyclinH1

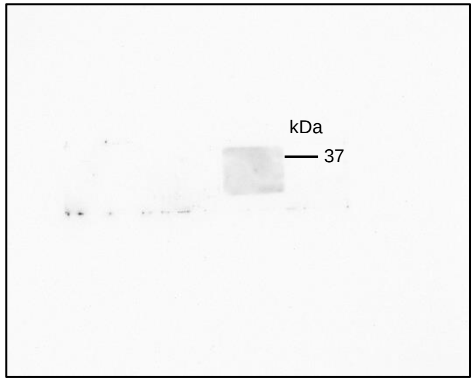

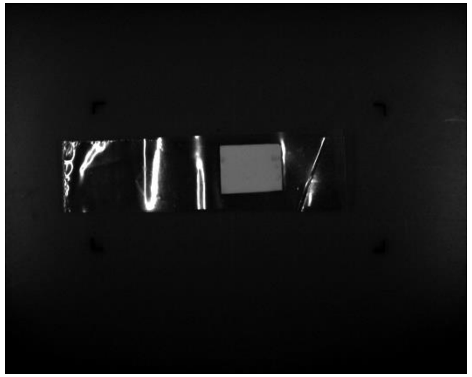

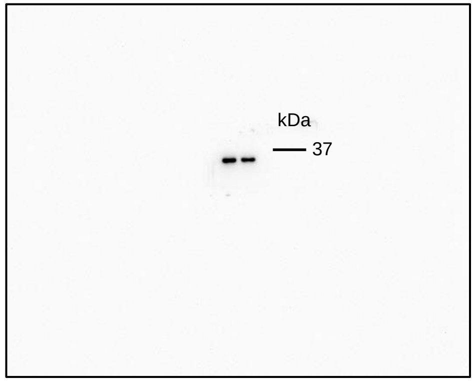

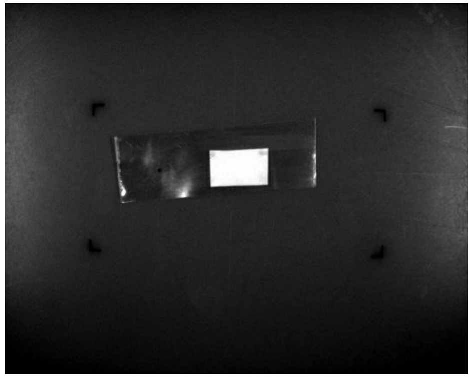

CDKL3

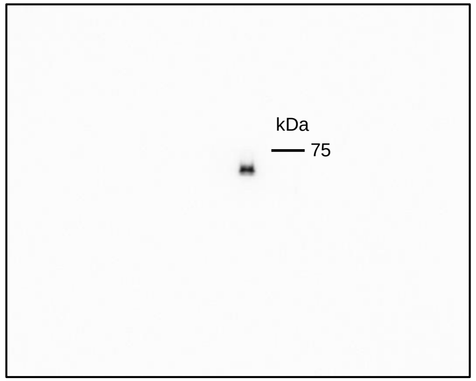

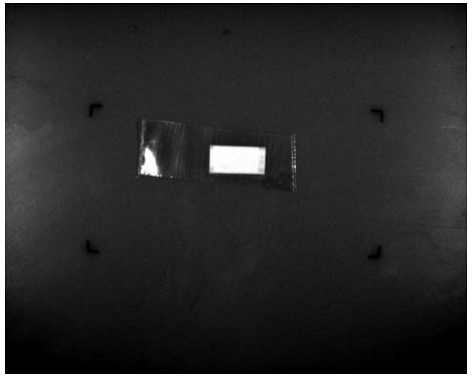

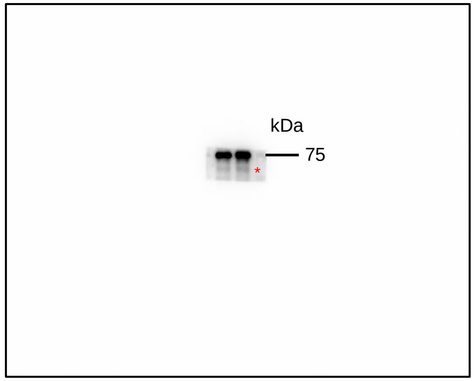

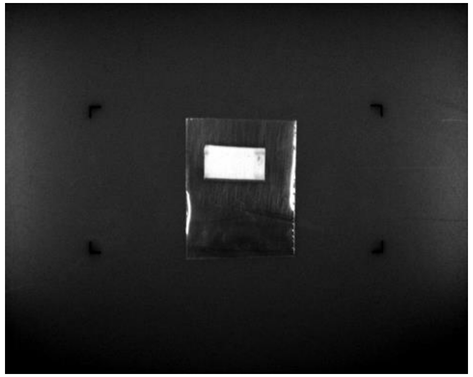

Full unedited blot for Figure 3B

CDK4

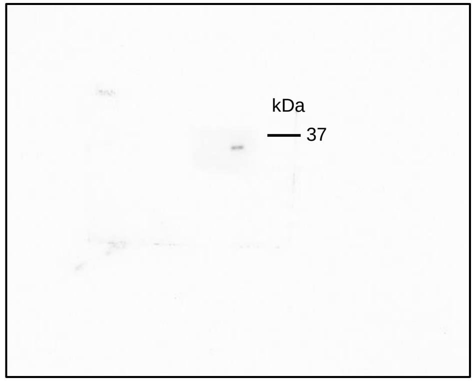

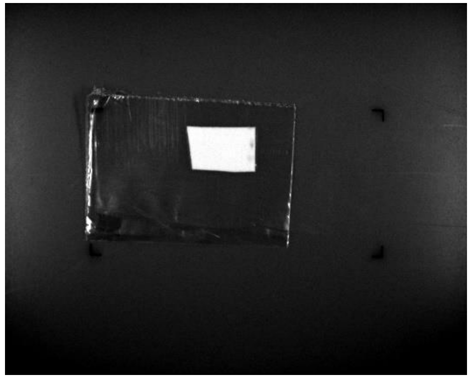

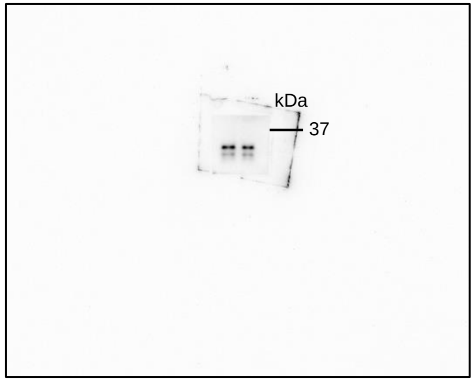

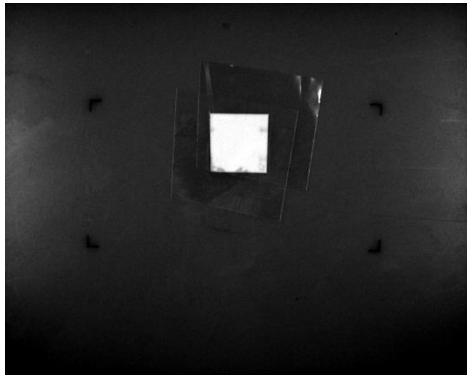

Full unedited blot for Figure 3C

MYC-cyclinA2

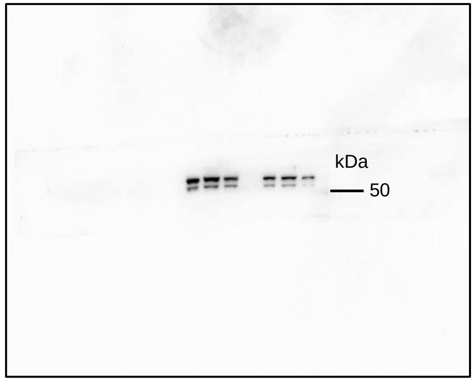

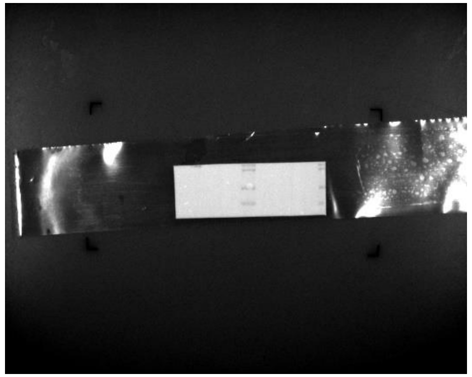

FLAG-CDKL3

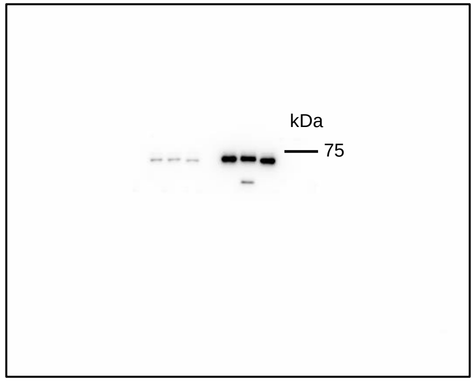

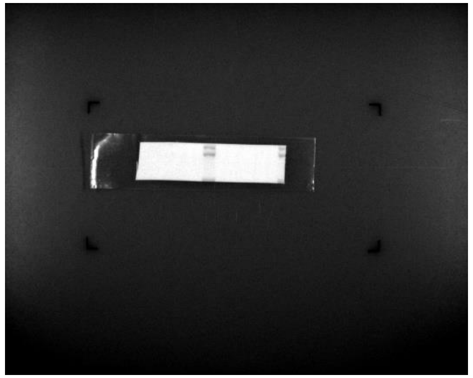

MYC-cyclinB1

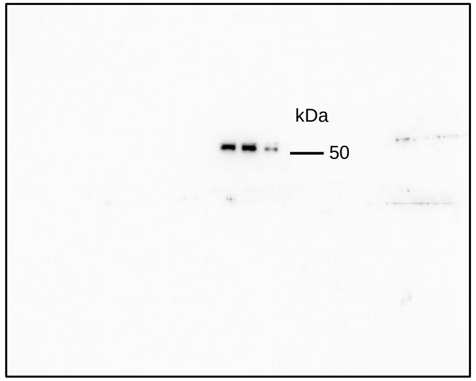

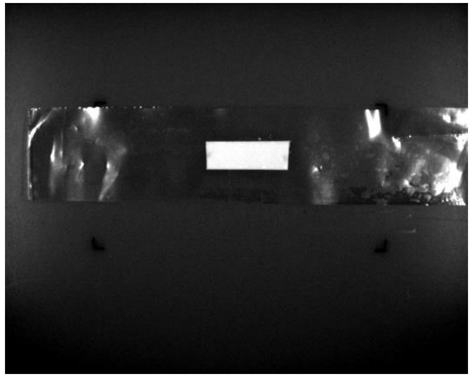

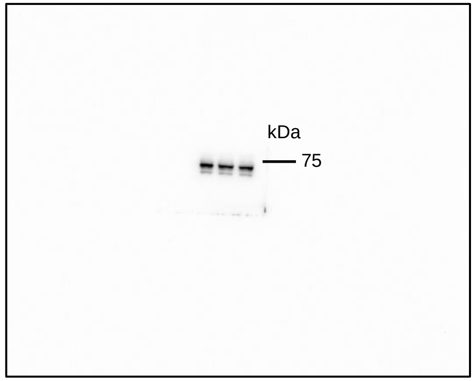

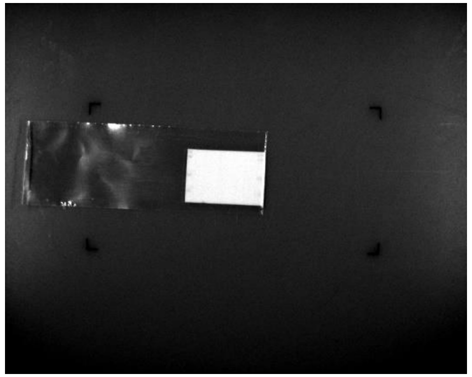

Full unedited blot for Figure 3C

FLAG-CDKL3

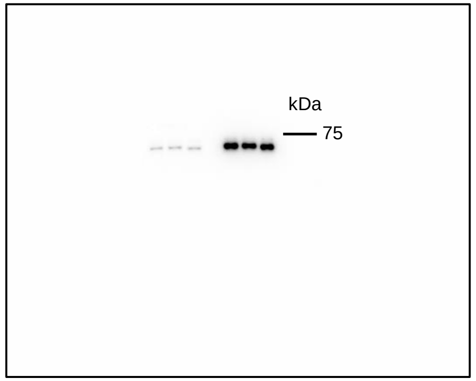

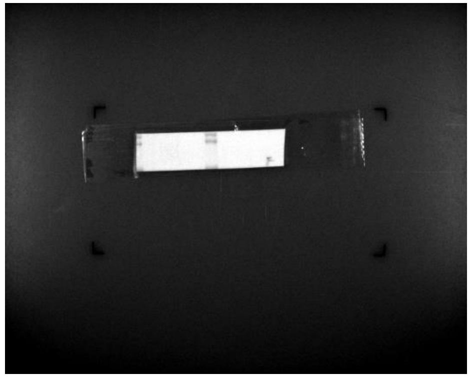

MYC-cyclinD1

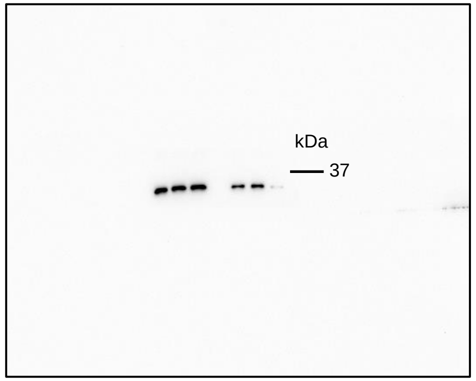

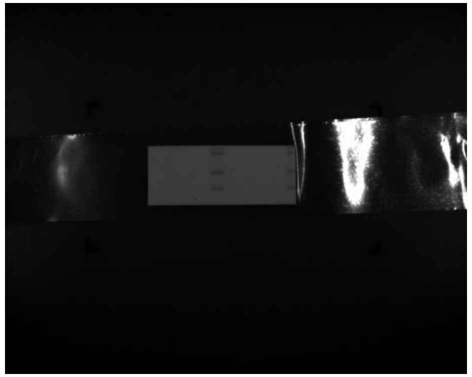

FLAG-CDKL3

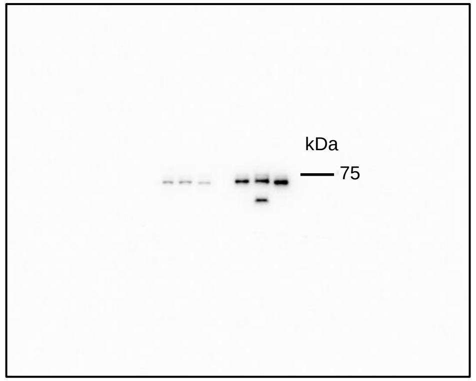

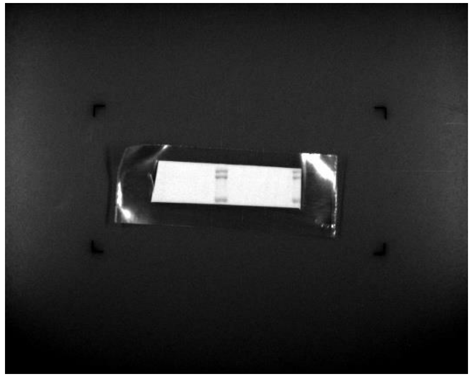

MYC-cyclinE1

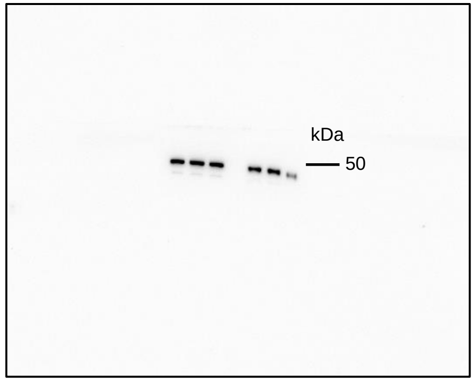

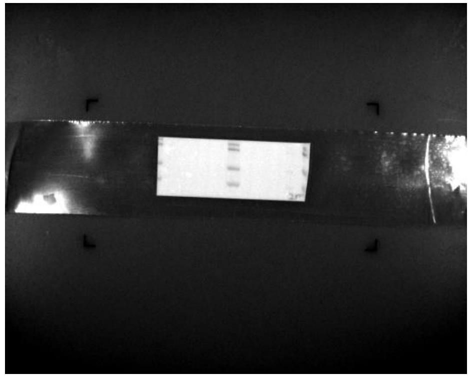

Full unedited blot for Figure 3C

FLAG-
CDKL3

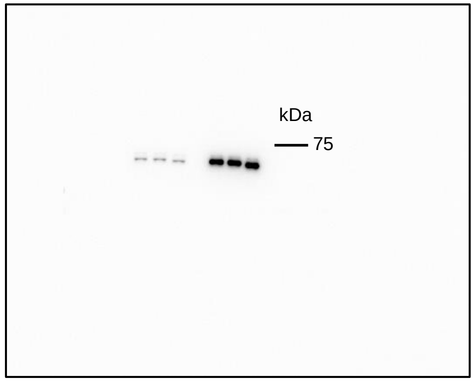

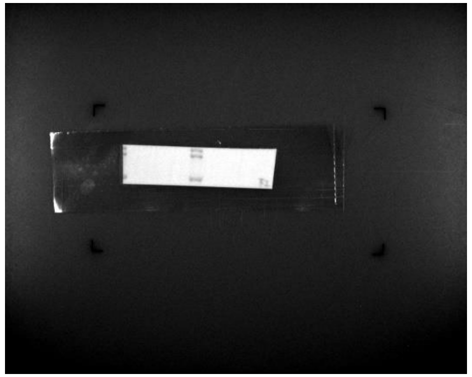

Full unedited blot for Figure 3E

Rb

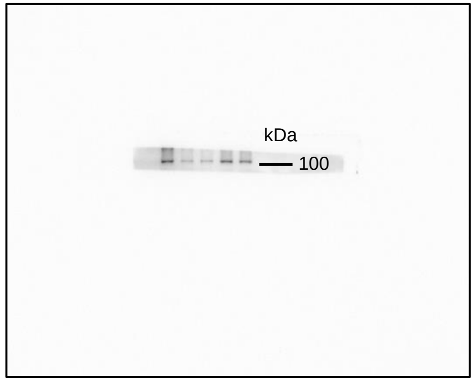

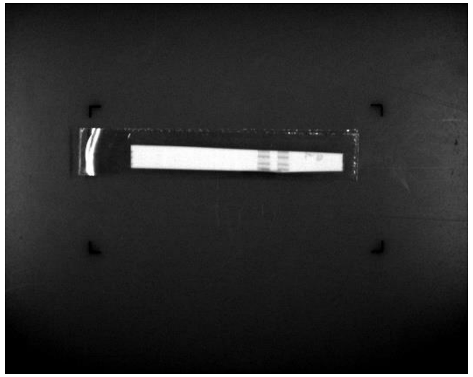

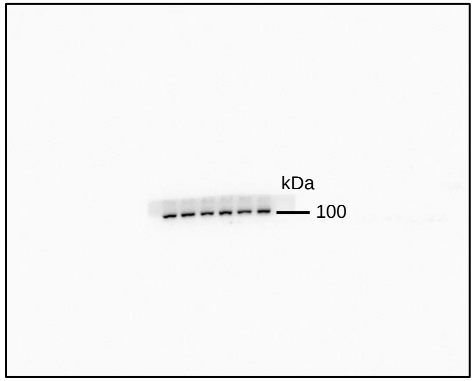

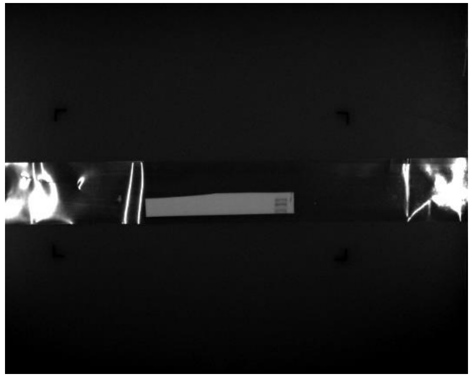

FLAG -CDKL3

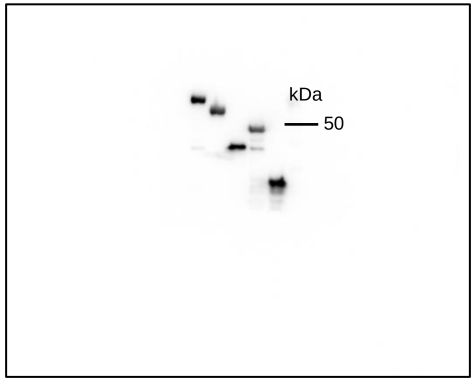

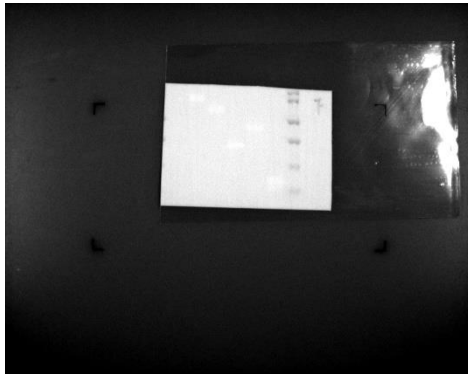

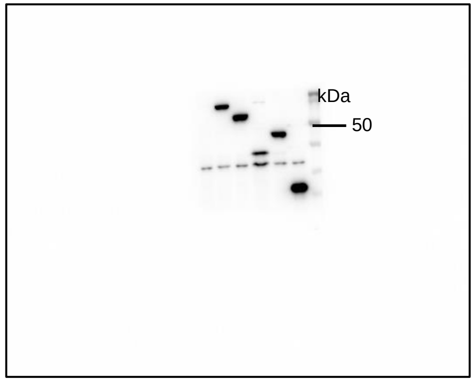

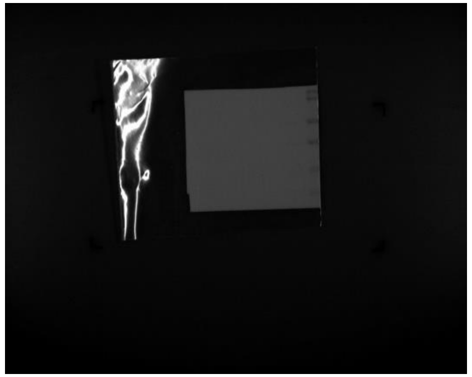

Full unedited blot for Figure 3G

pRb

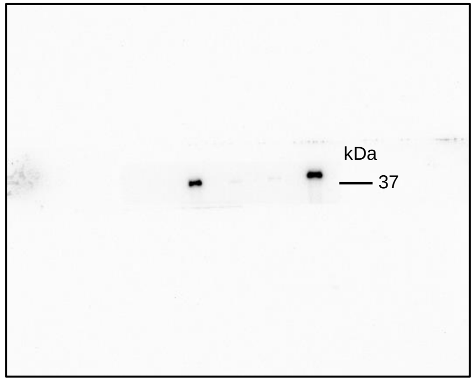

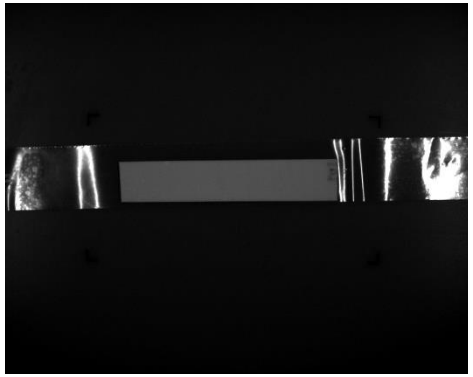

FLAG

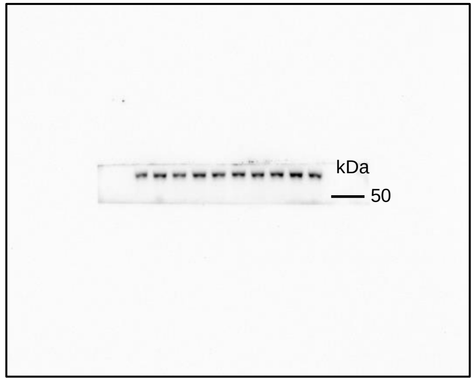

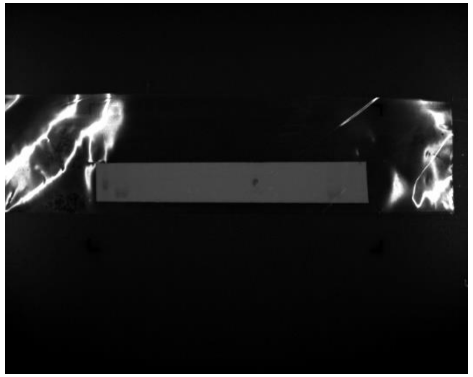

GST

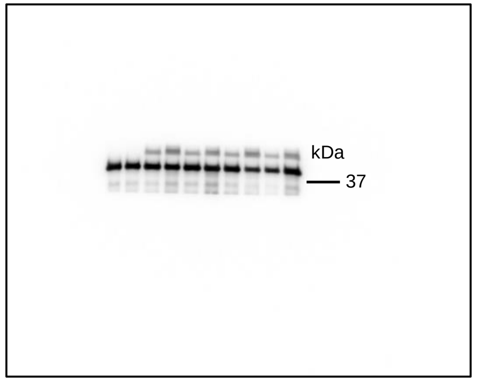

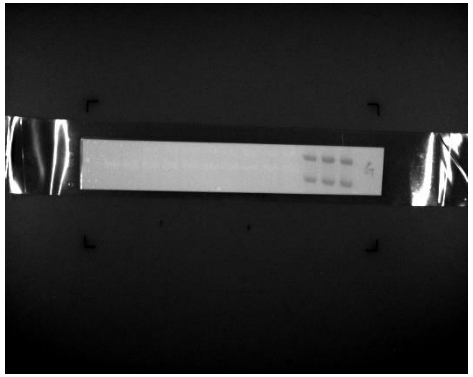

Full unedited blot for Figure 3H

pRb

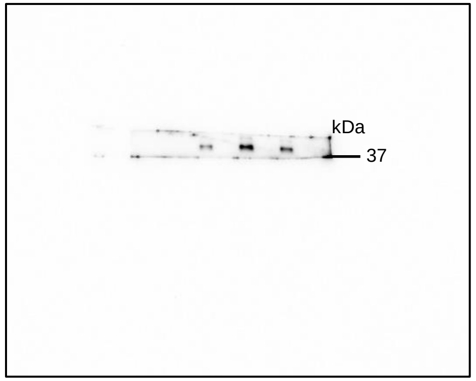

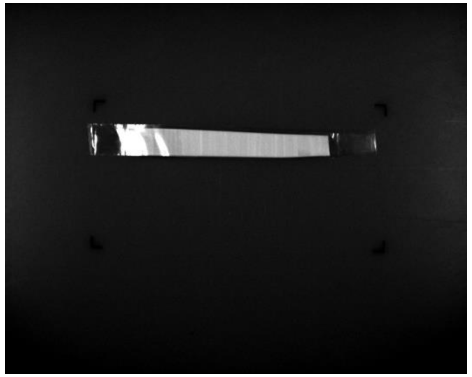

FLAG

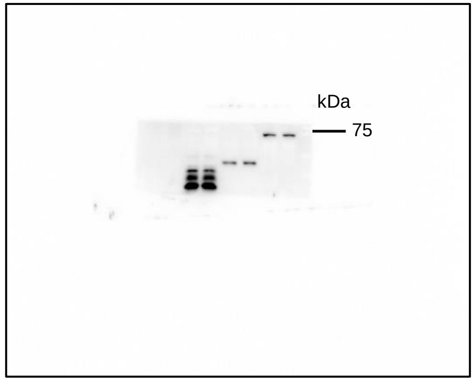

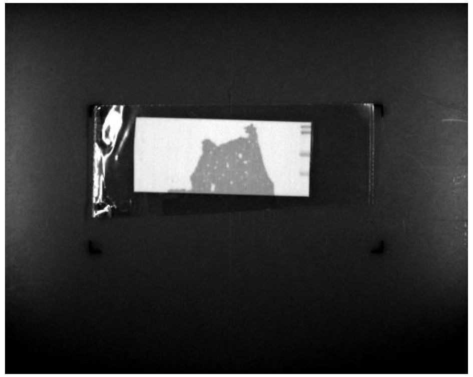

GST

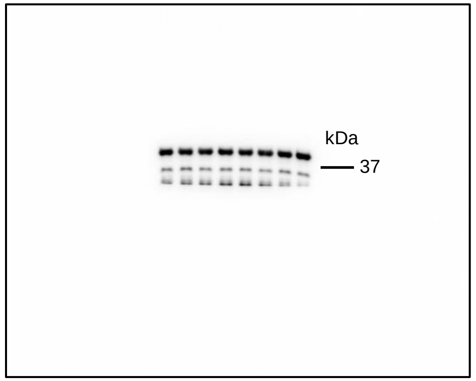

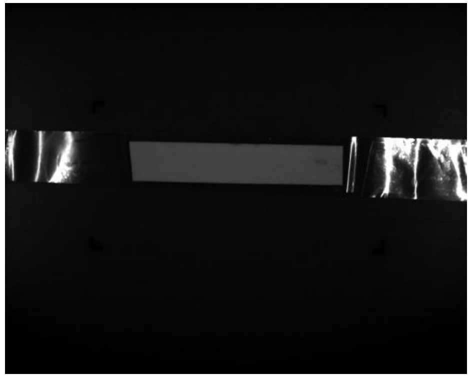

Full unedited blot for Figure 3I

pRb

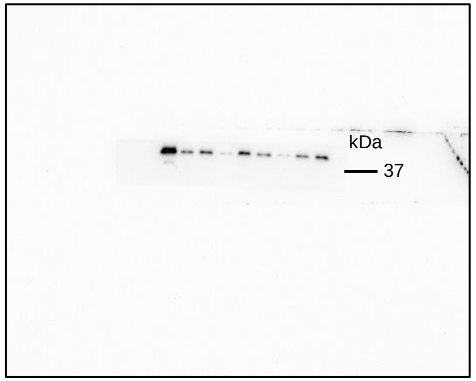

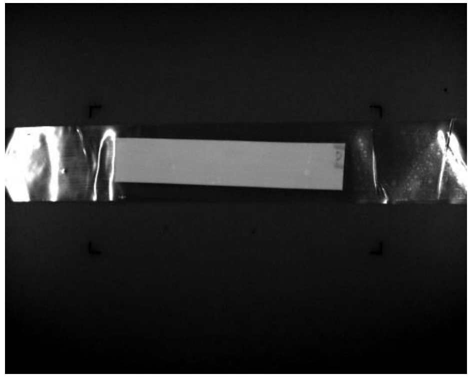

FLAG

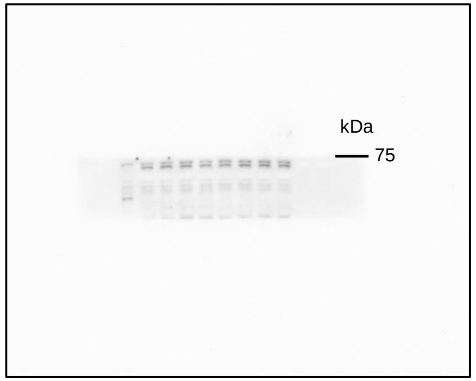

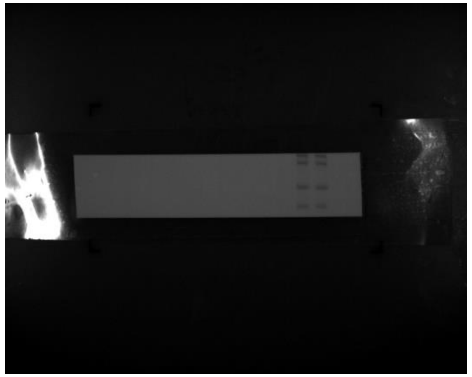

GST

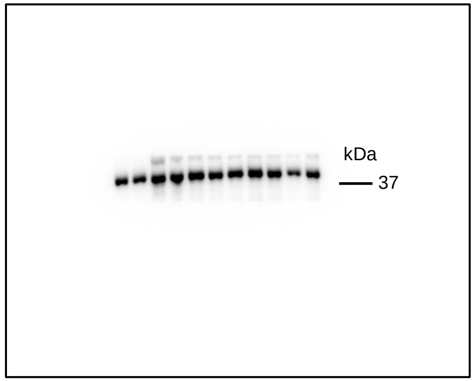

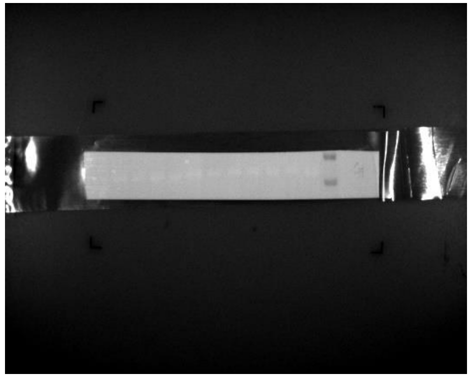

Full unedited blot for Figure 3J

pRb

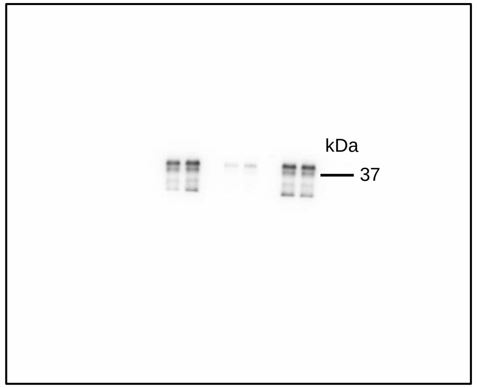

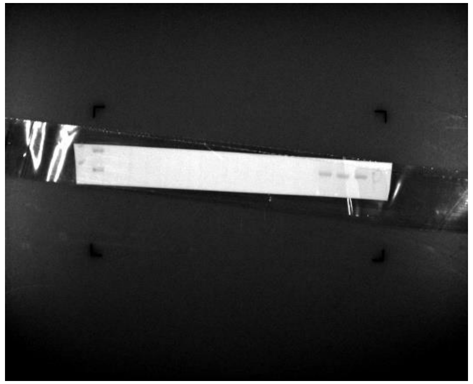

FLAG

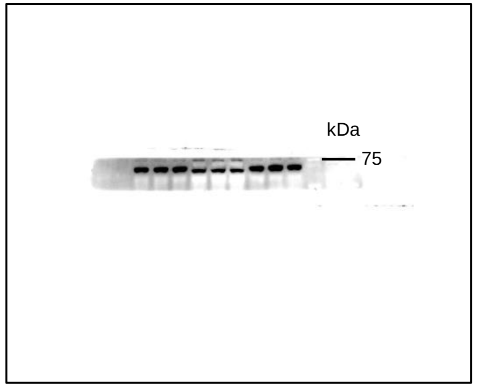

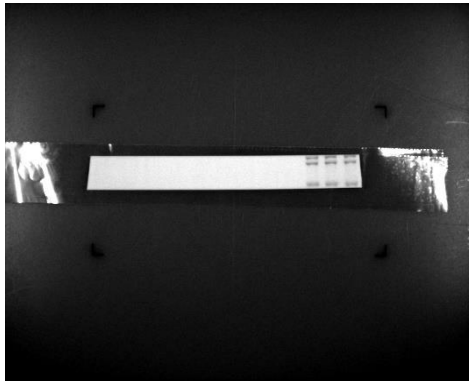

GST

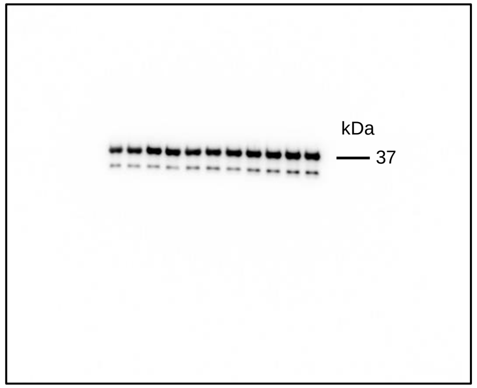

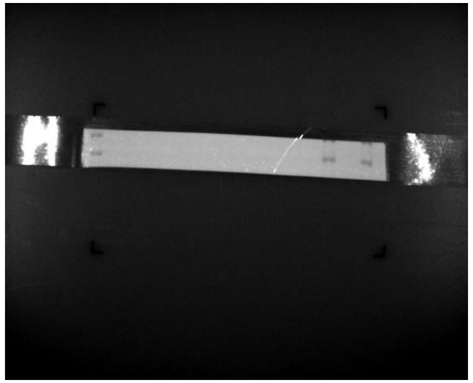

Full unedited blot for Figure 3K

pRb

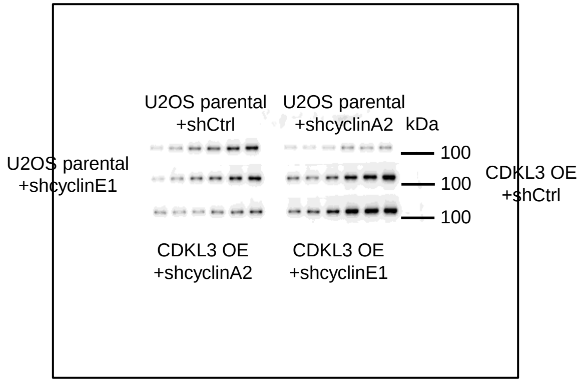

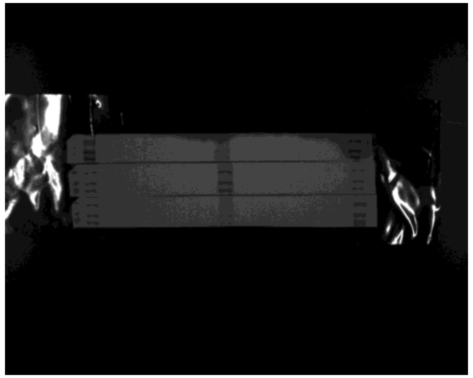

Rb

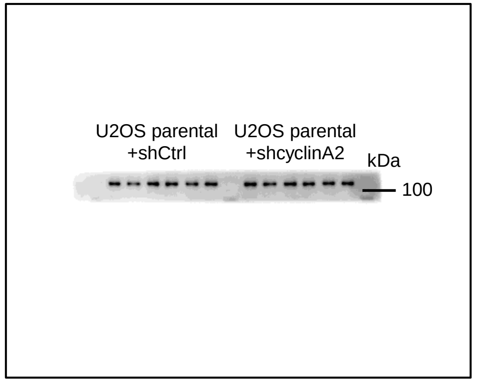

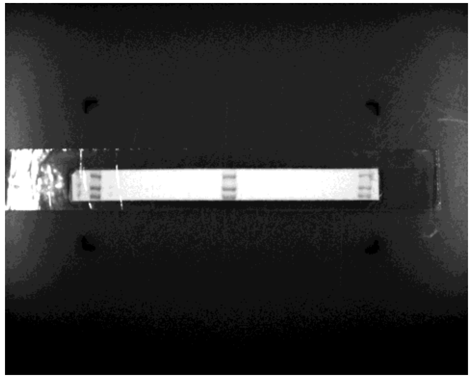

Rb

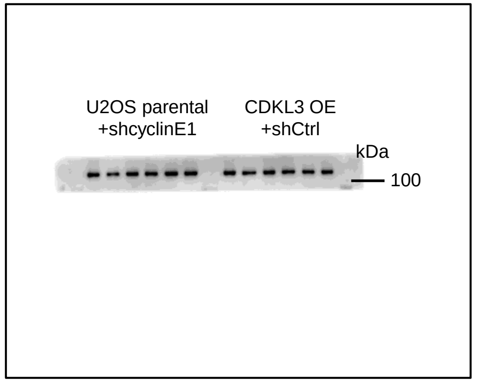

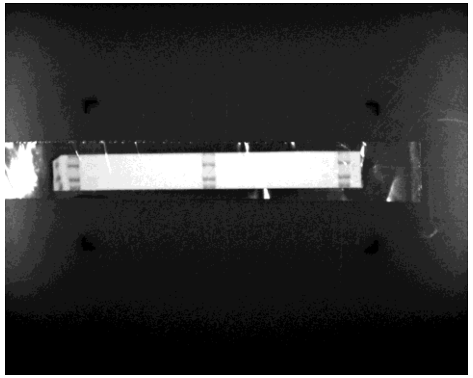

Rb

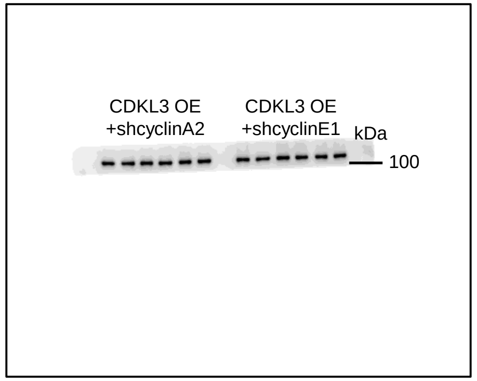

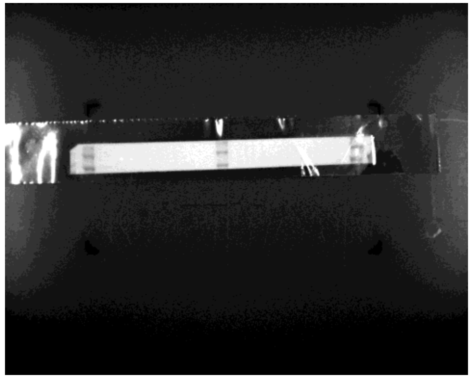

Full unedited blot for Figure 3K

FLAG

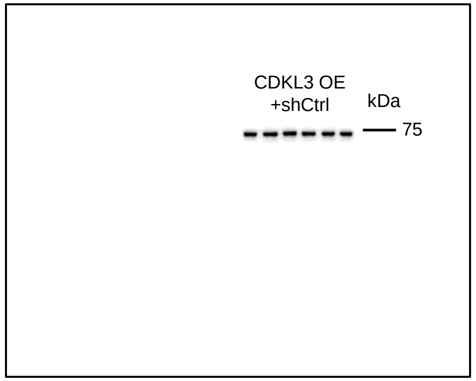

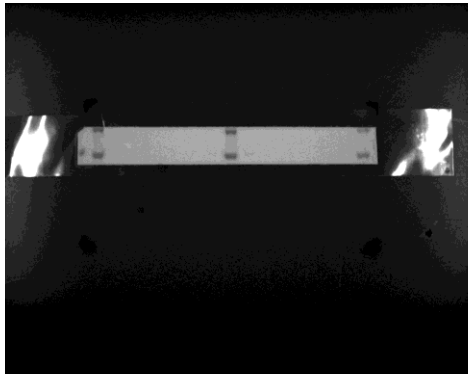

FLAG

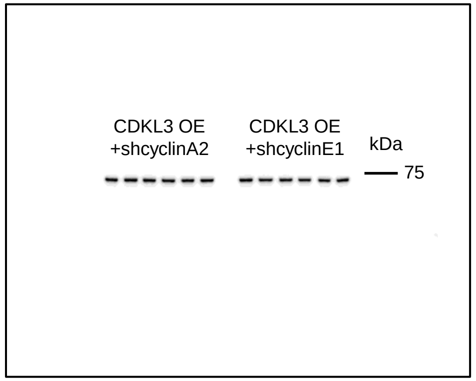

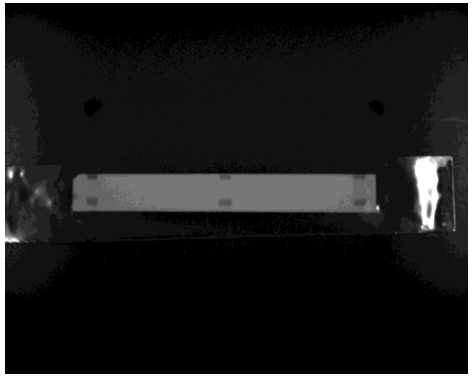

Full unedited blot for Figure 3K

GAPDH

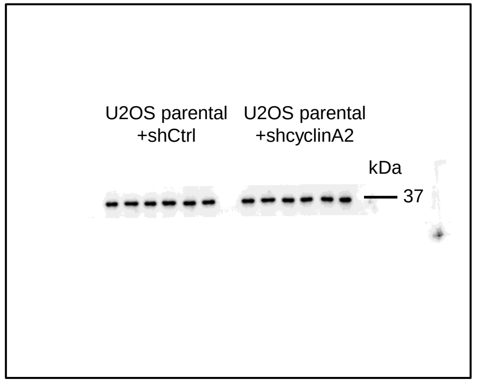

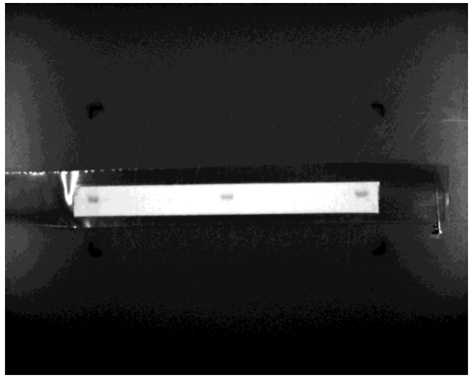

GAPDH

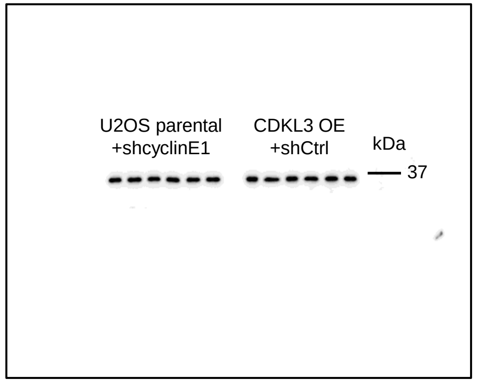

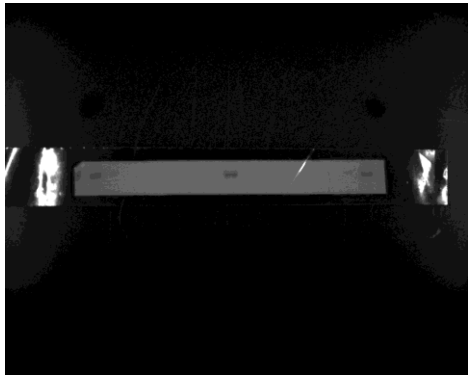

GAPDH

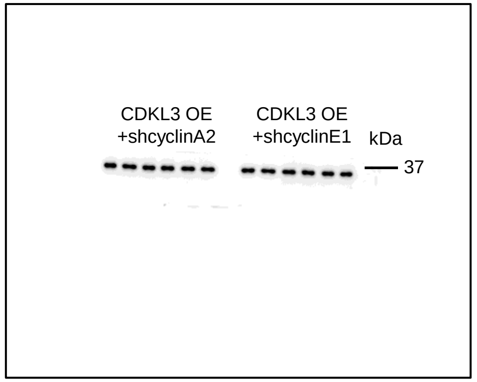

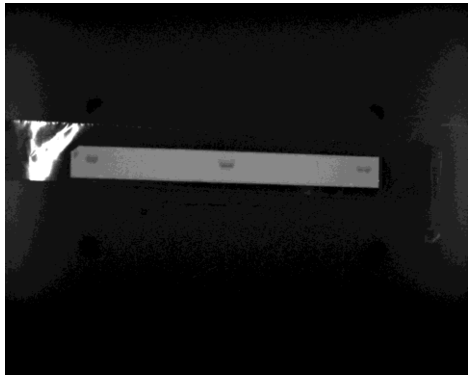

Full unedited blot for Figure 4A

pRb

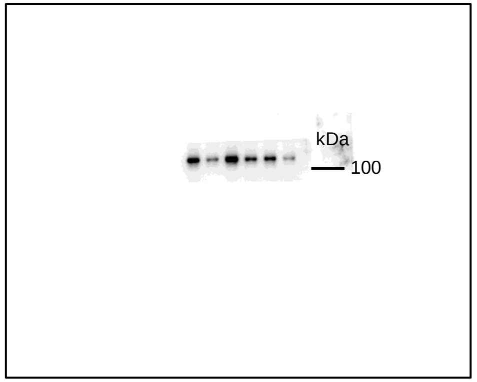

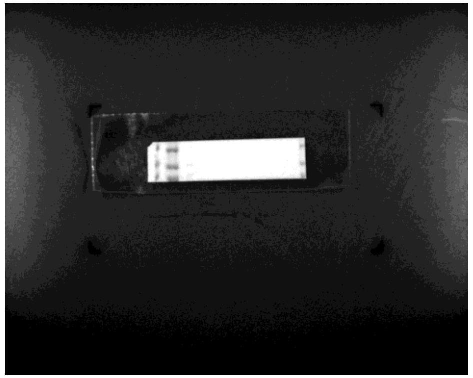

Rb

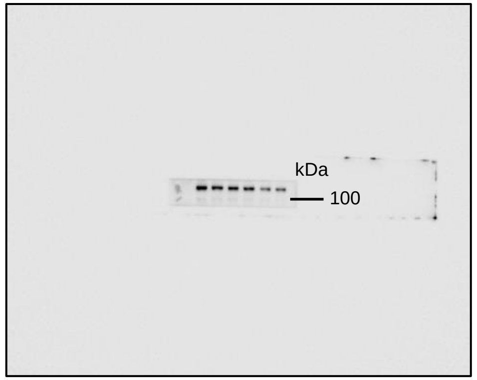

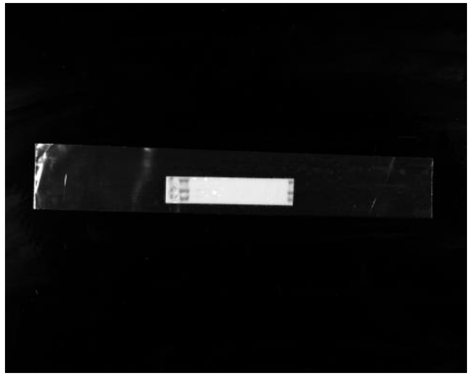

CDKL3

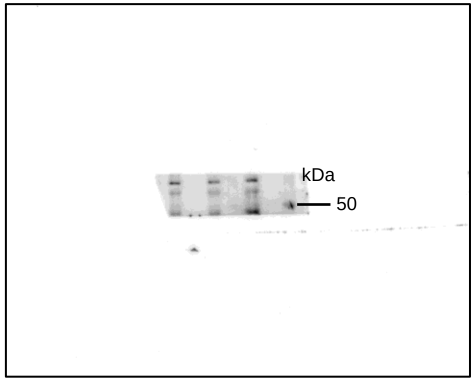

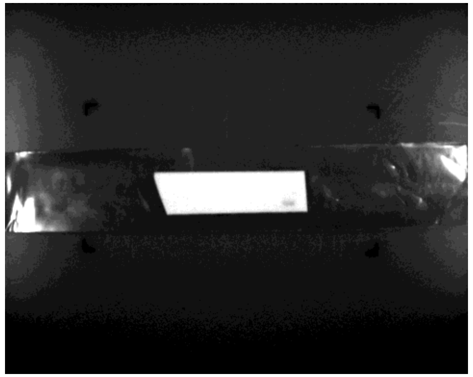

CDK1

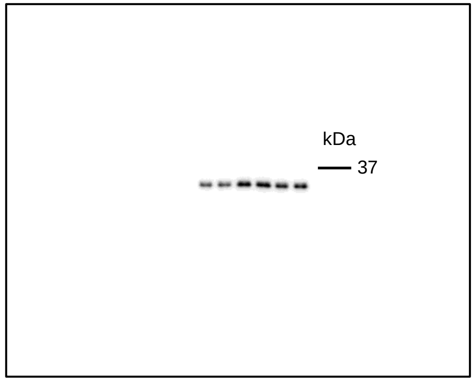

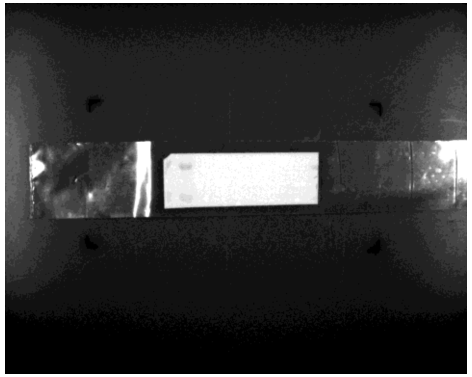

Full unedited blot for Figure 4A

CDK2

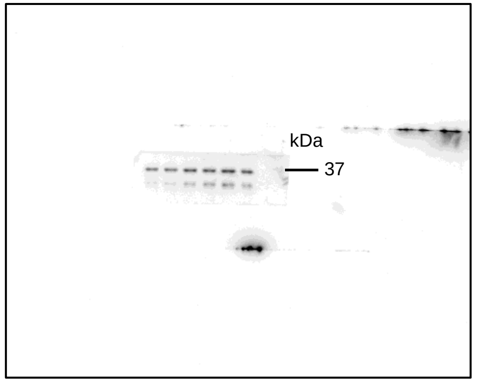

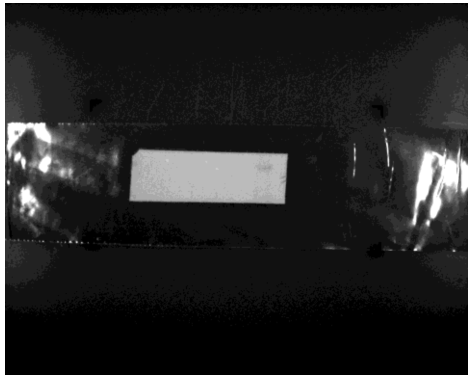

CDK3

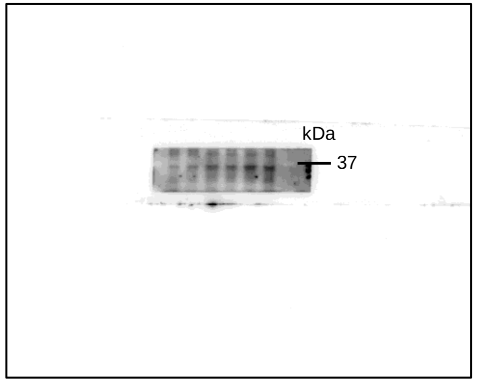

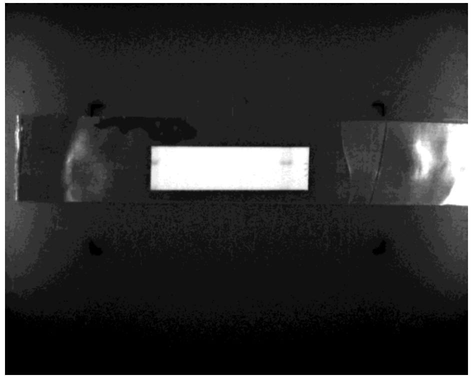

CDK4

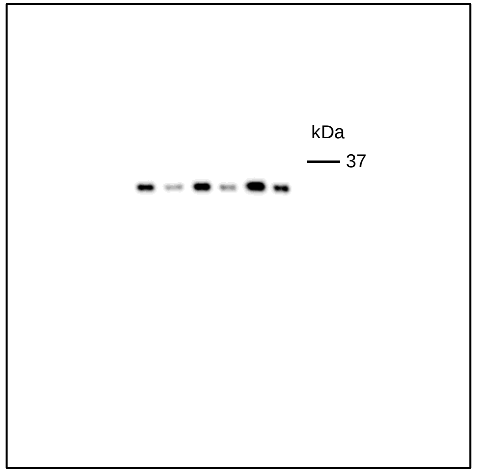

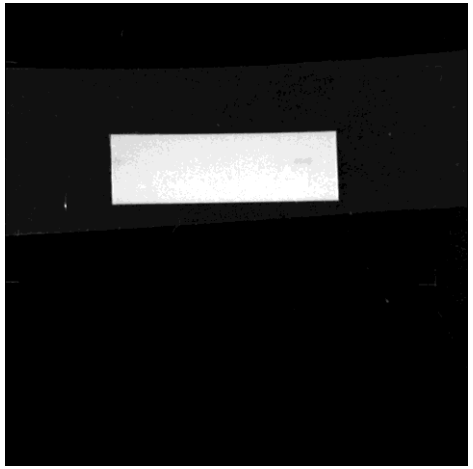

Full unedited blot for Figure 4A

CDK6

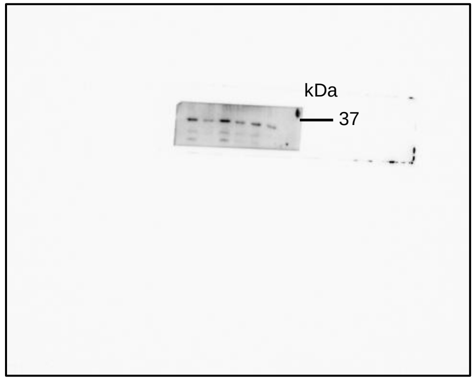

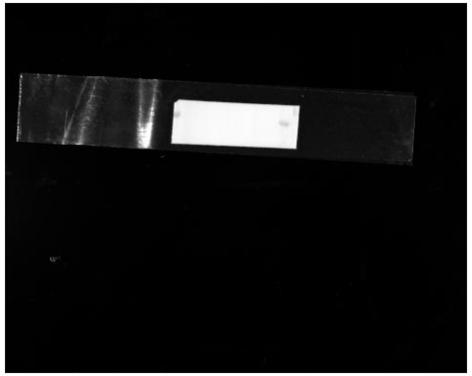

GAPDH

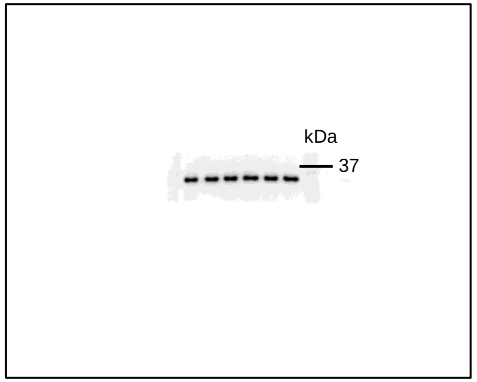

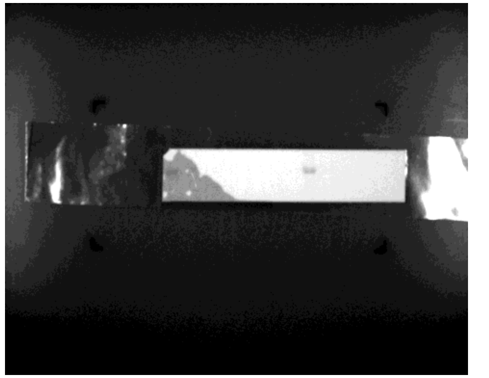

Full unedited blot for Figure 4C

V5-Ub

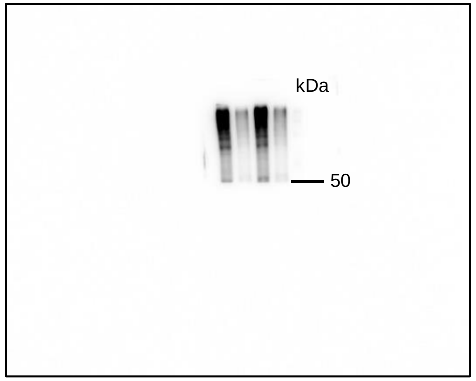

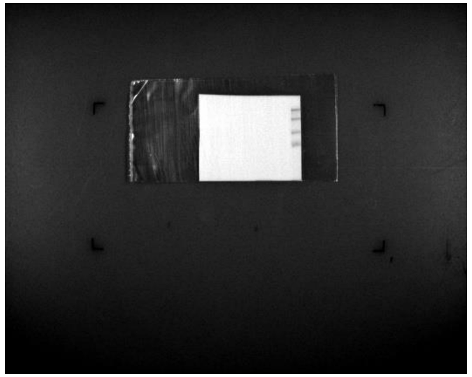

MYC
-CDK4

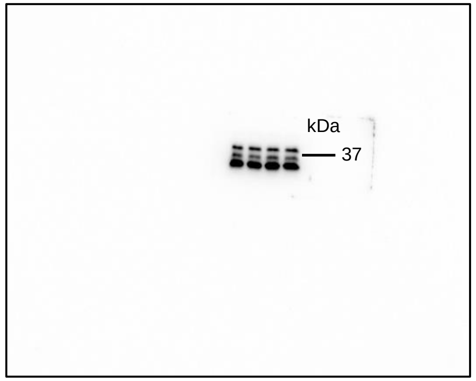

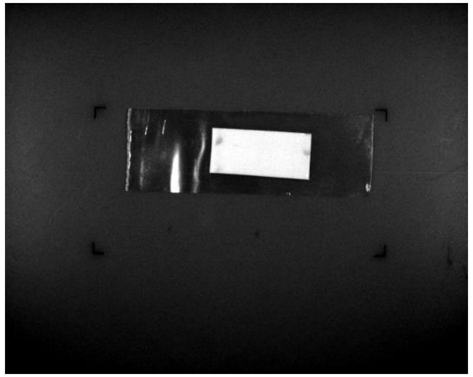

FLAG
-CDKL3

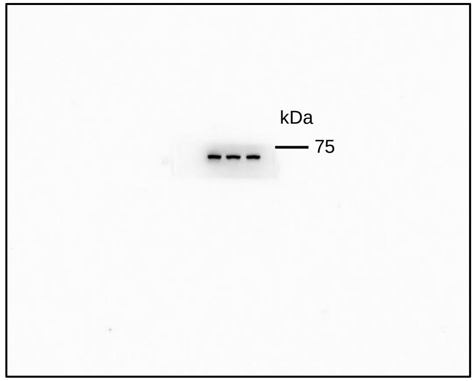

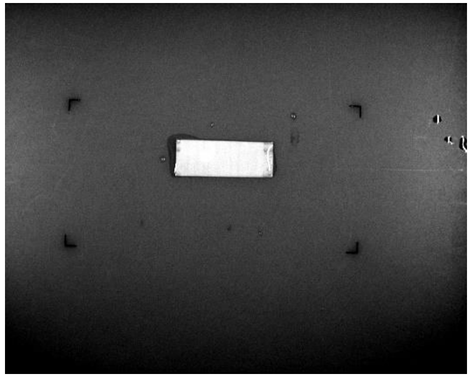

MYC
-CDK4

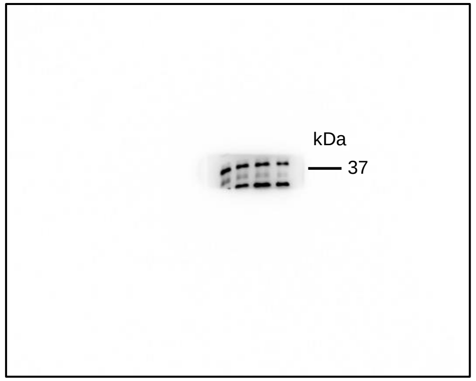

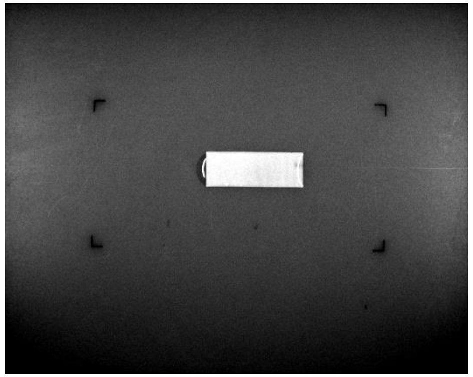

Full unedited blot for Figure 4D

CDK4 pT172

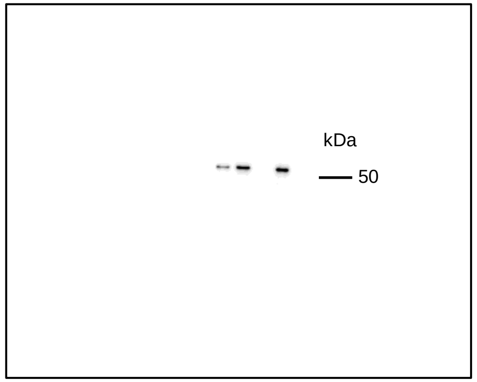

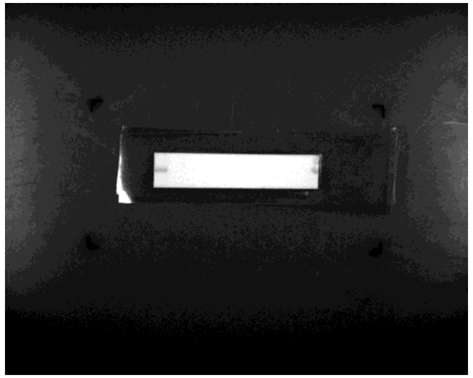

FLAG

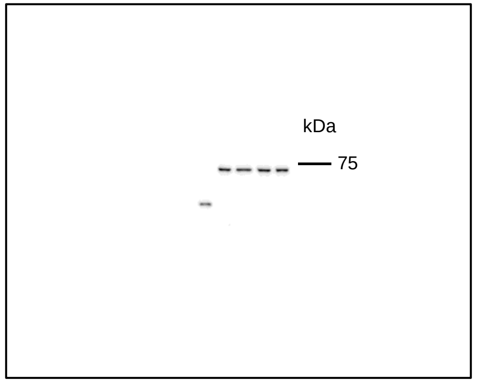

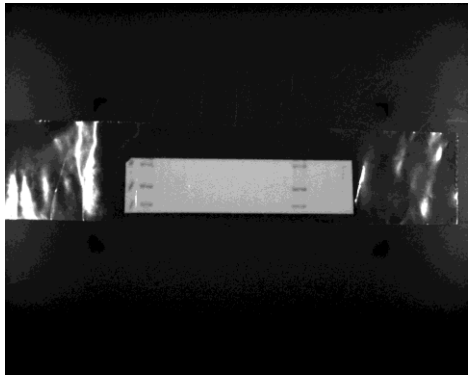

GST

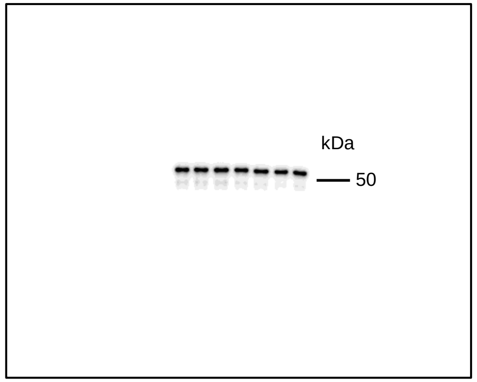

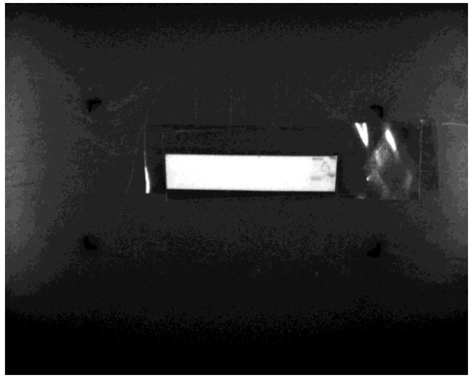

Full unedited blot for Figure 4E

CDK4 pT172

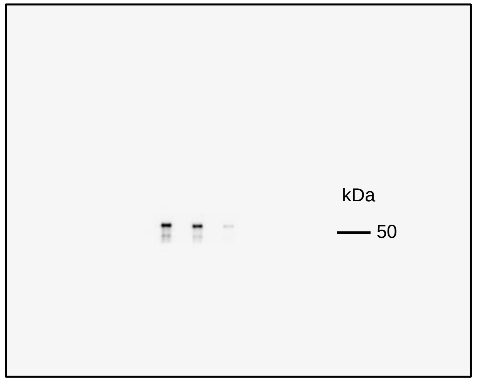

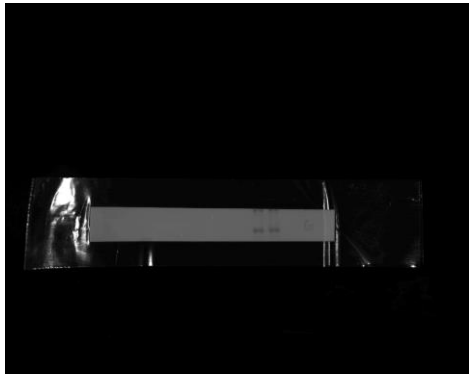

FLAG

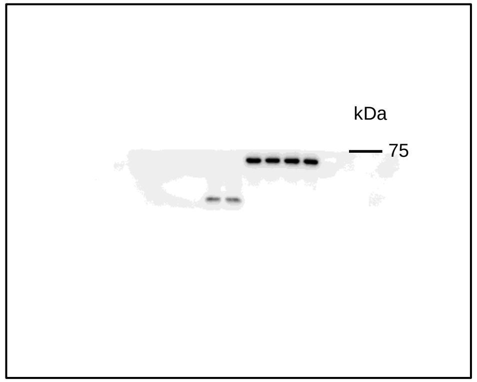

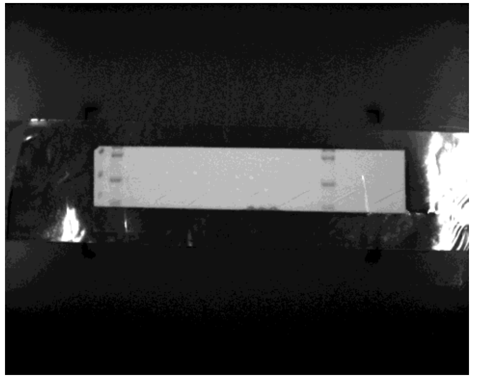

GST

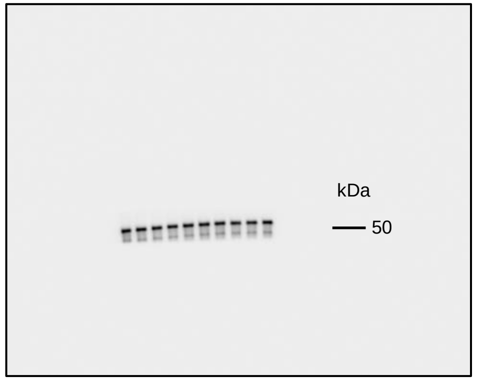

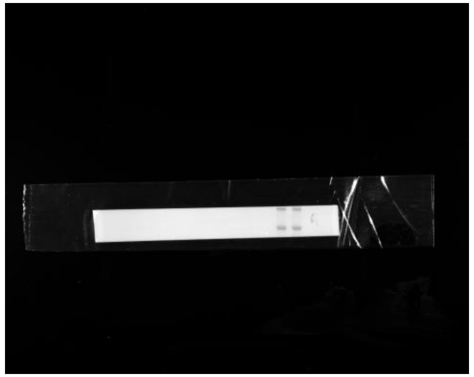

Full unedited blot for Figure 4F

CDK4

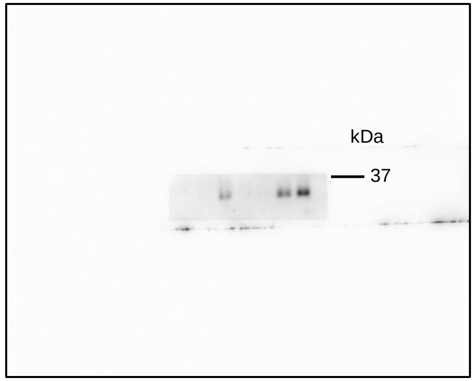

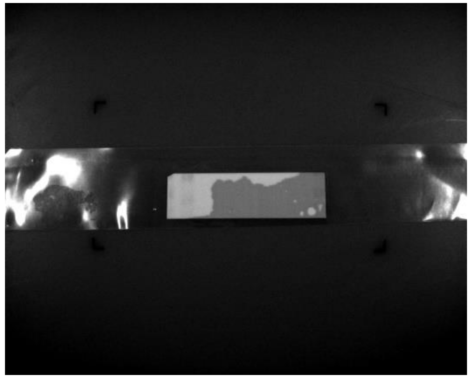

FLAG
-CDKL3

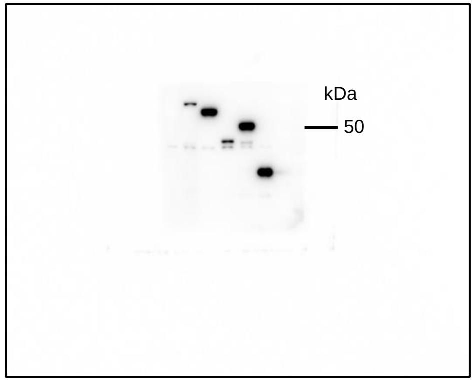

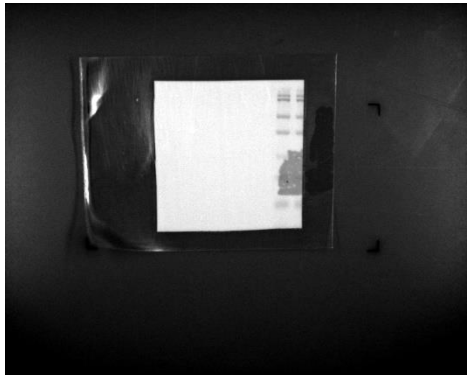

CDK4

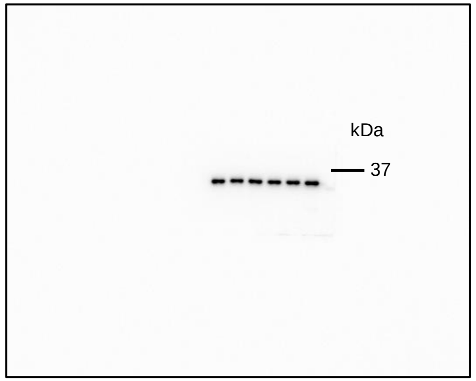

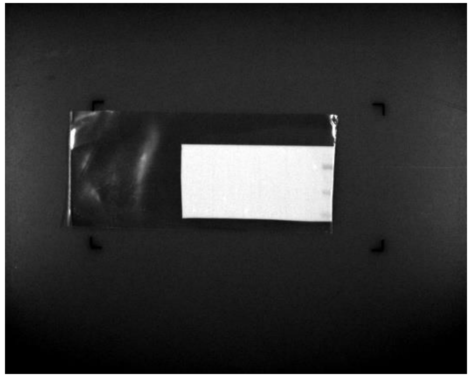

FLAG
-CDKL3

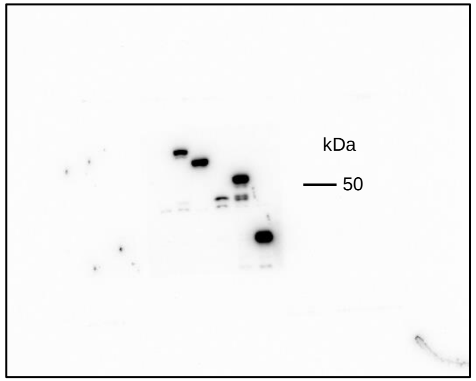

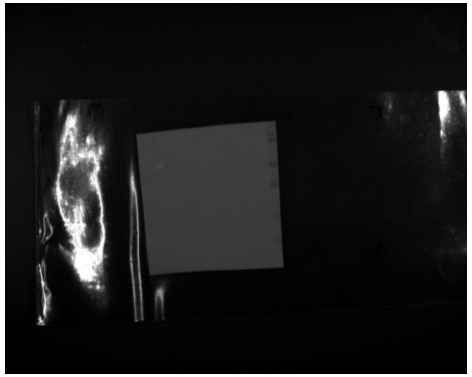

Full unedited blot for Figure 4G

Ub

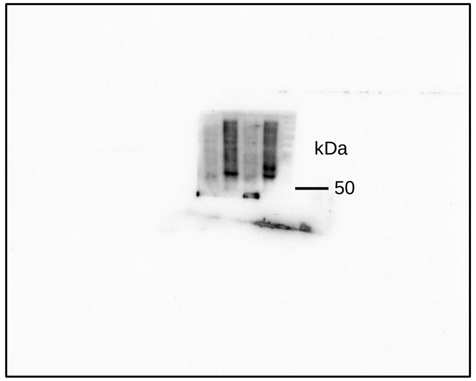

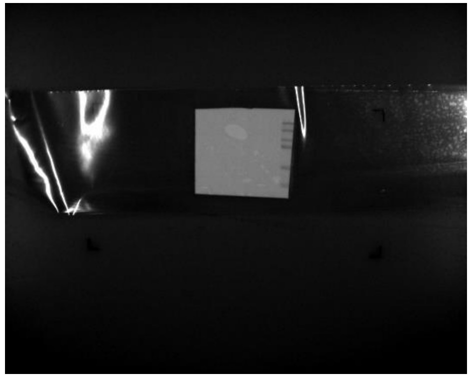

pT172

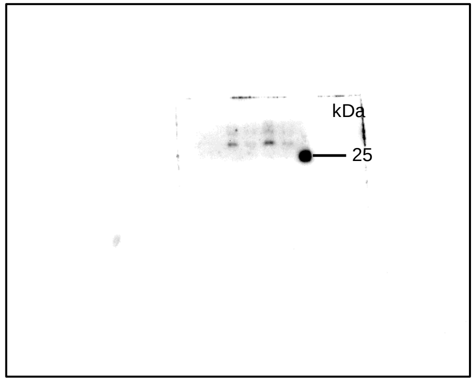

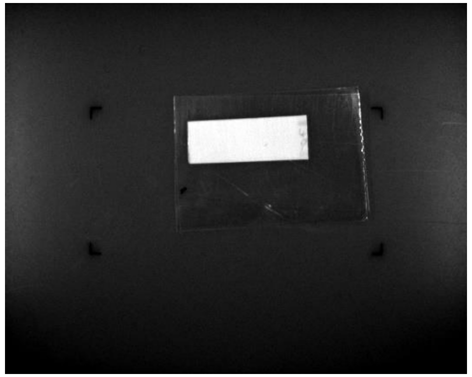

CDK4

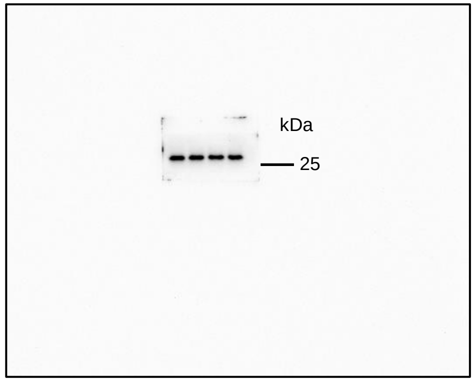

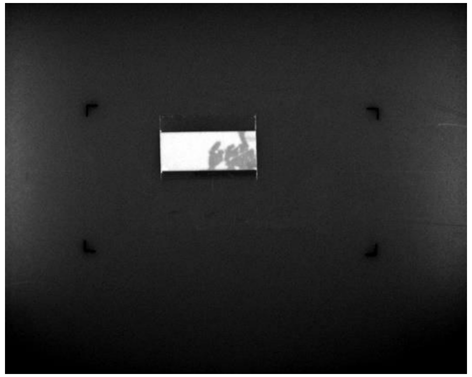

Full unedited blot for Figure 4G

CDKL3

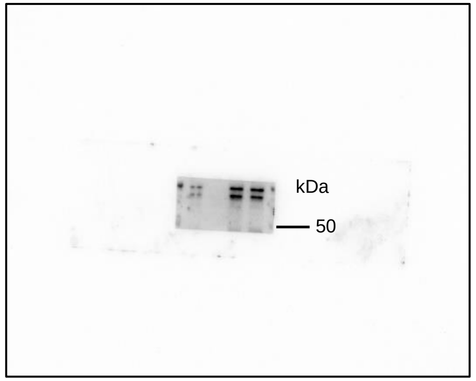

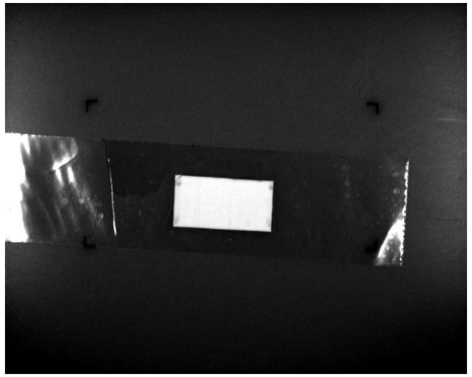

CDK4

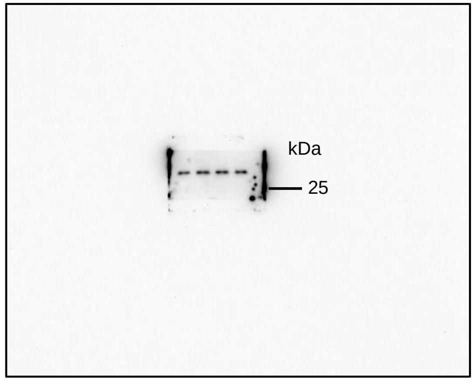

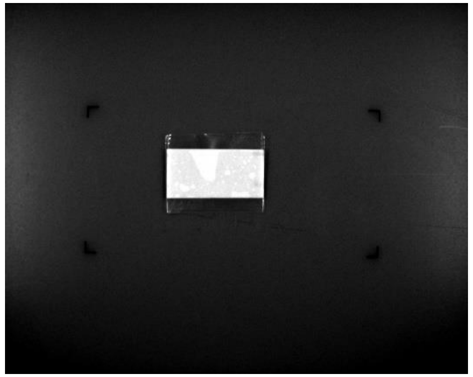

Full unedited blot for Figure 5A

V5-Ub

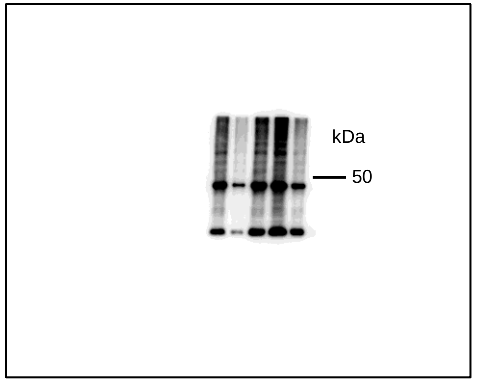

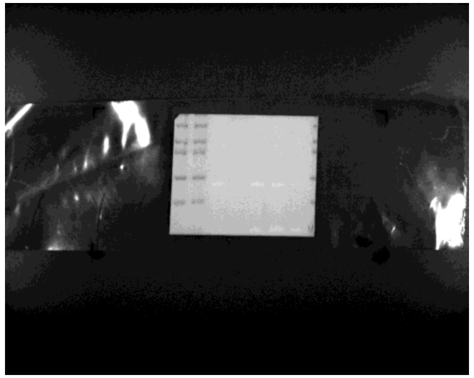

CDK4

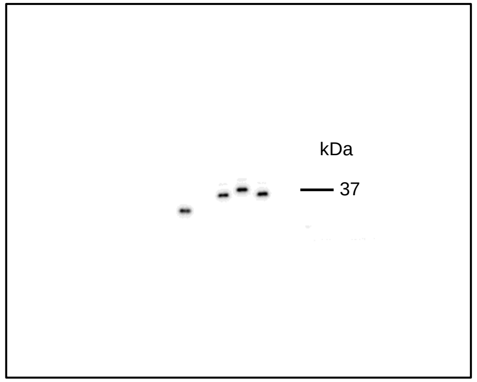

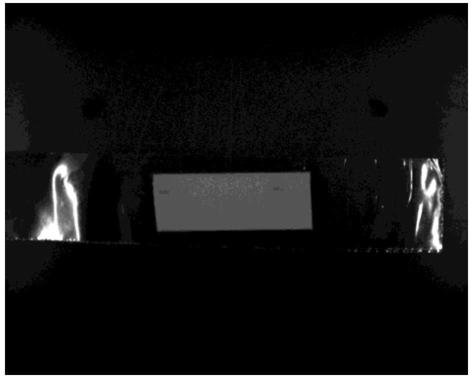

Full unedited blot for Figure 5A

CDK4

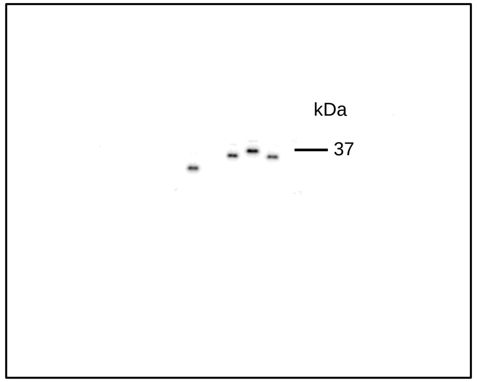

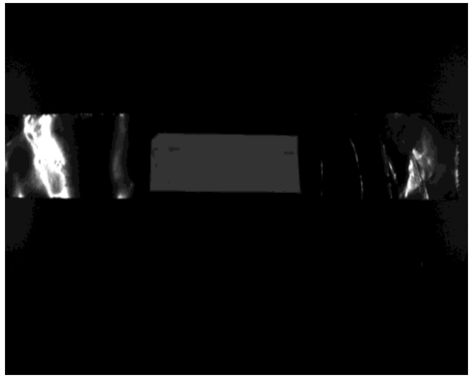

FLAG
-CDK4

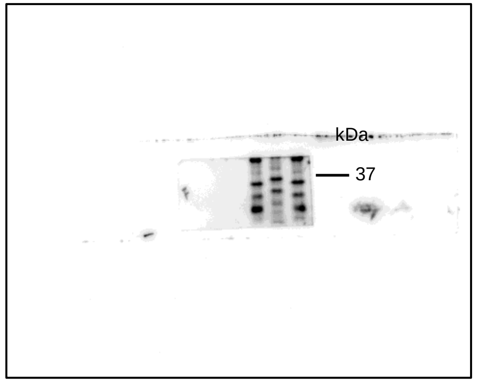

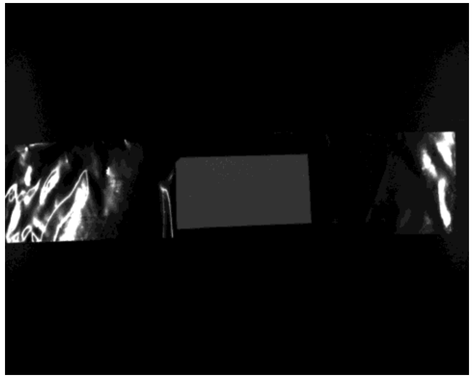

GAPDH

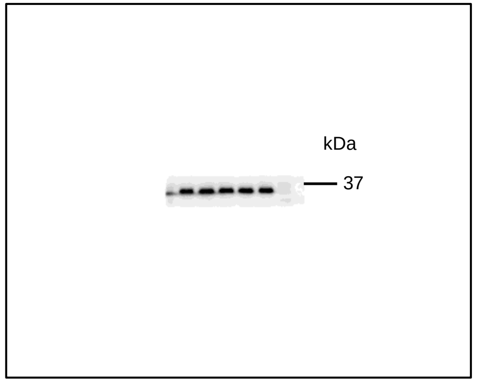

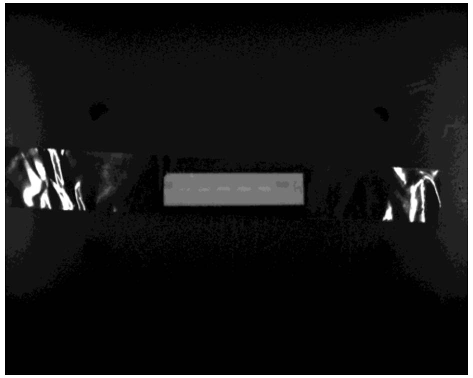

Full unedited blot for Figure 5B

CDK4

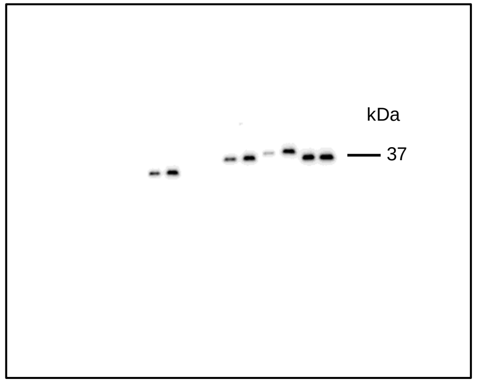

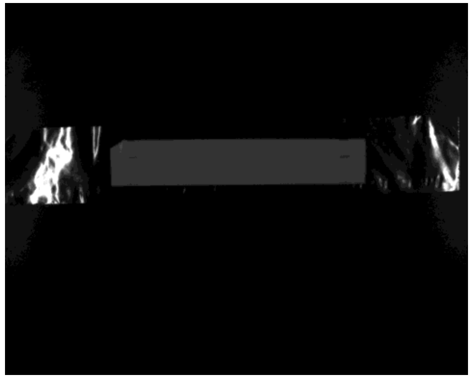

GAPDH

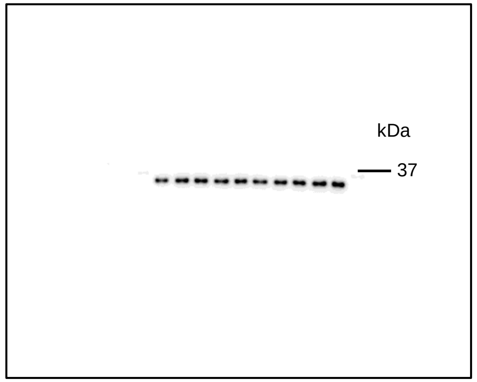

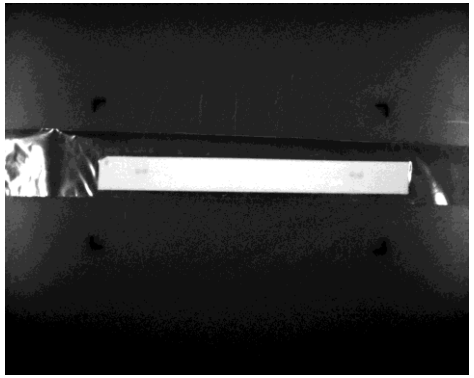

Full unedited blot for Figure 5E

pRb

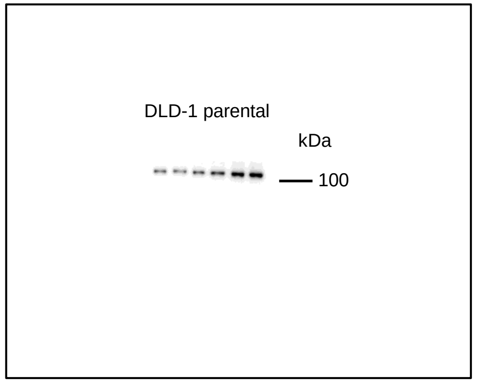

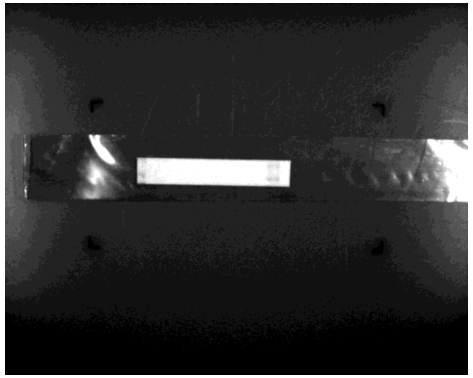

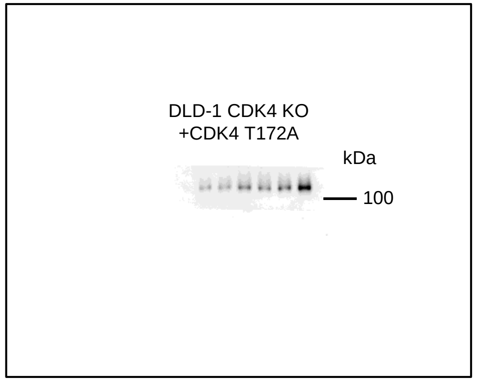

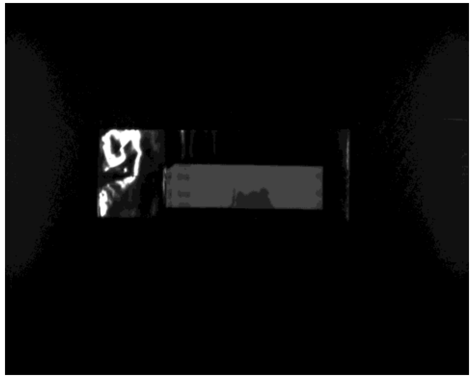

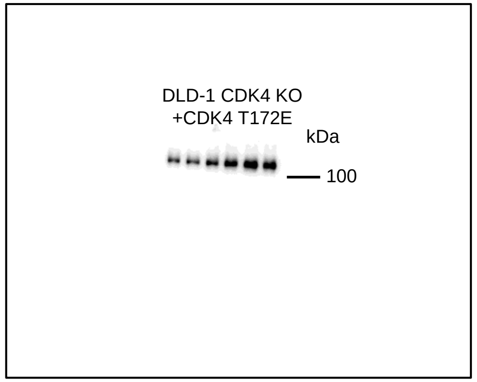

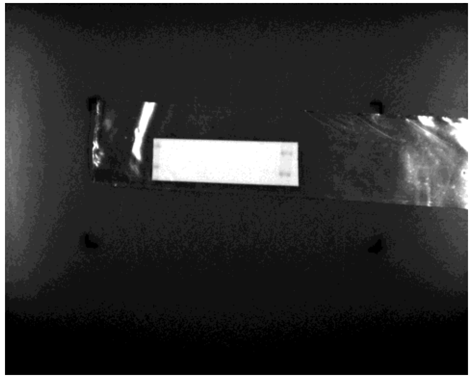

Full unedited blot for Figure 5E

pRb

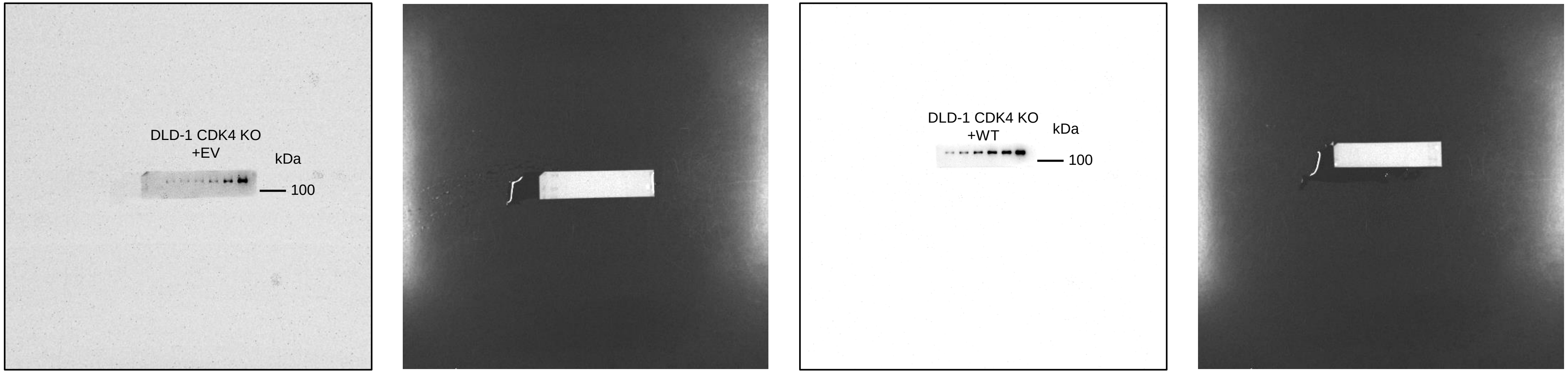

Full unedited blot for Figure 5E

Rb

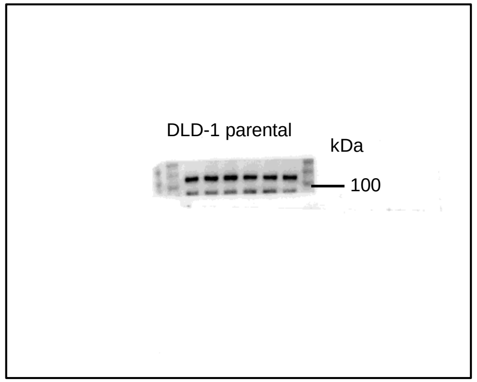

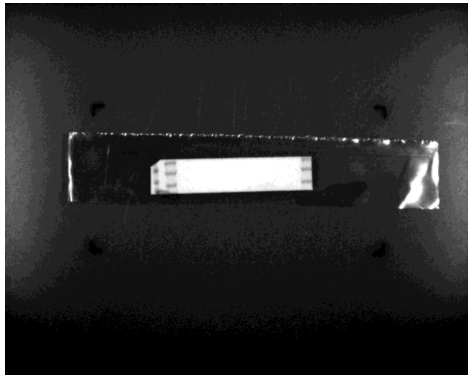

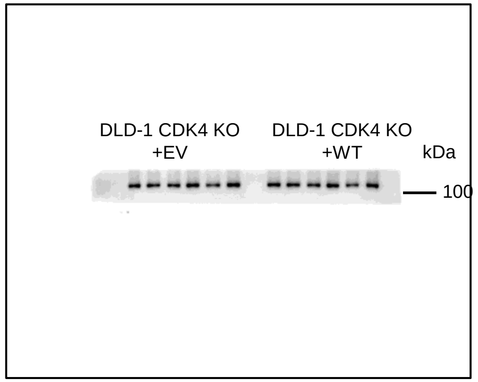

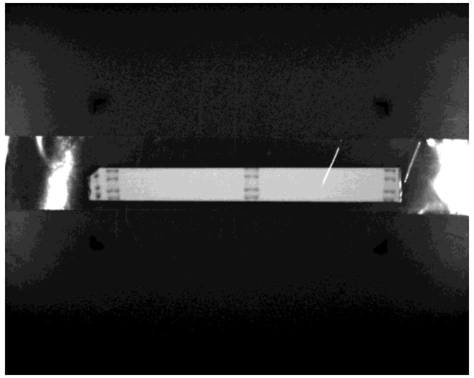

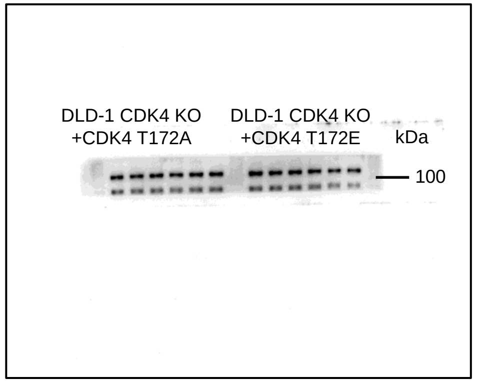

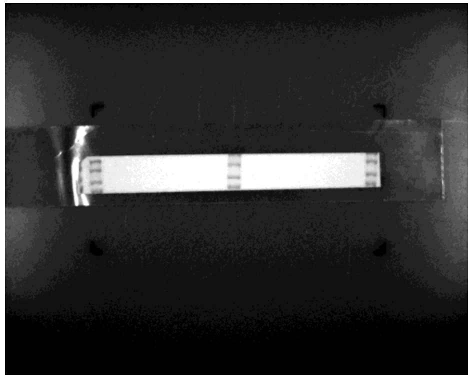

Full unedited blot for Figure 5E

cyclinA2

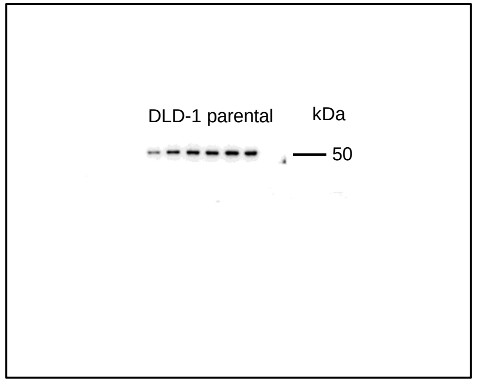

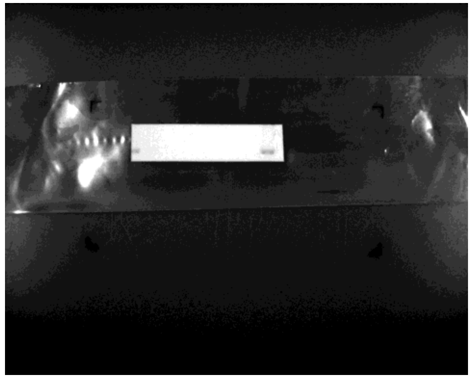

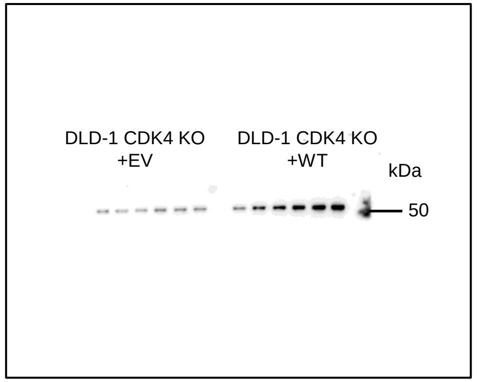

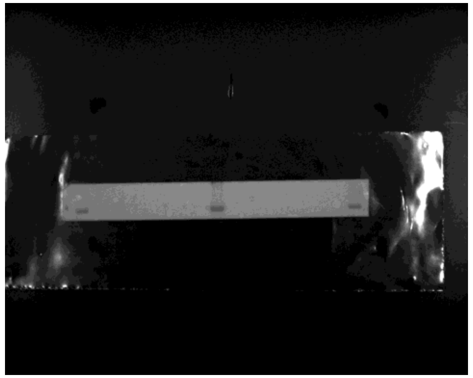

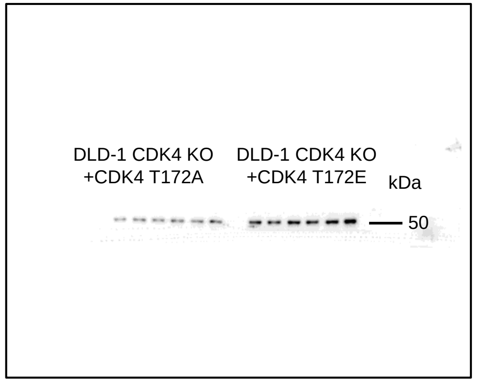

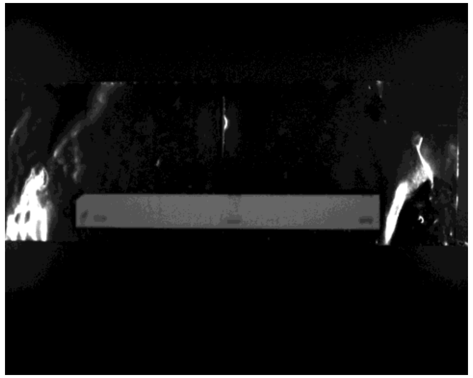

Full unedited blot for Figure 5E

CDK4

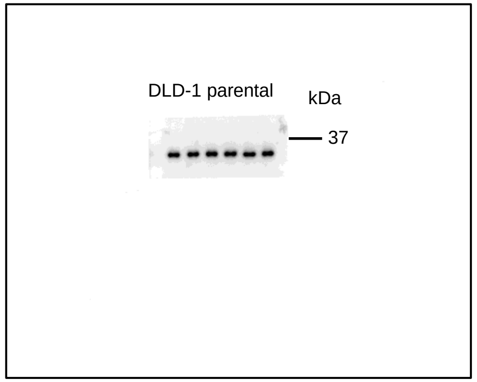

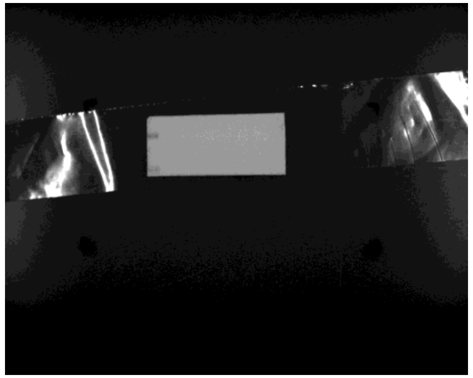

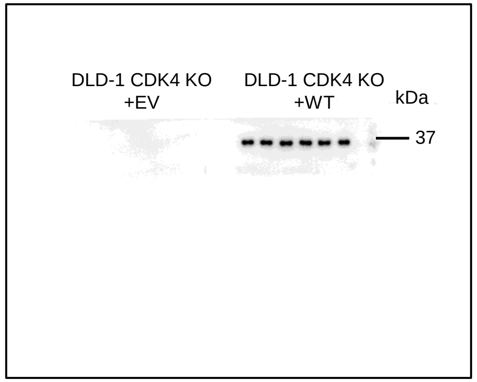

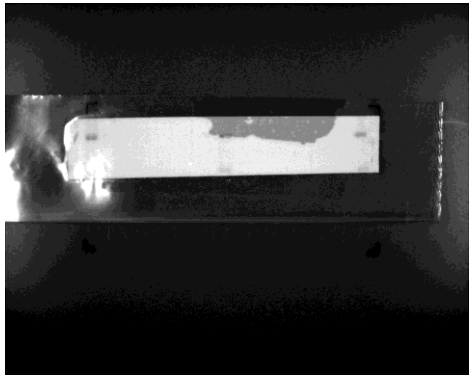

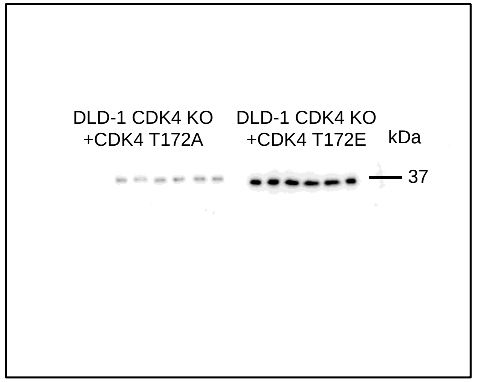

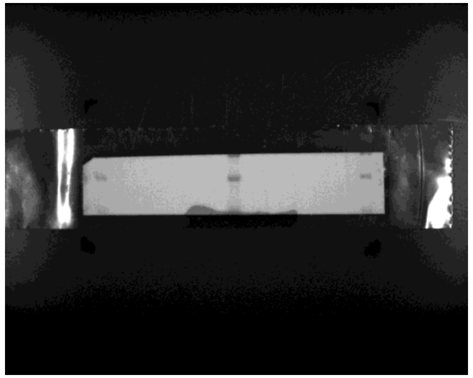

Full unedited blot for Figure 5E

GAPDH

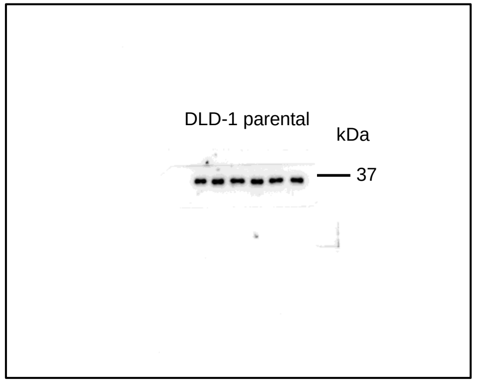

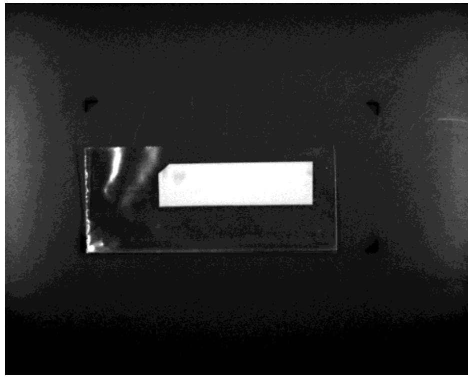

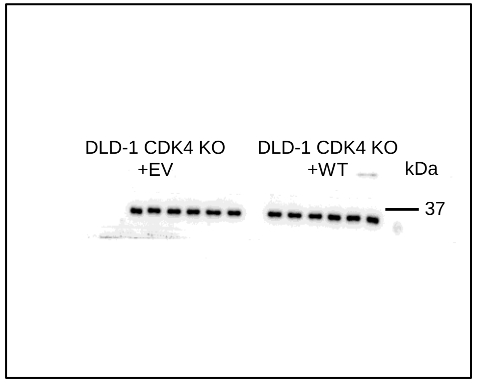

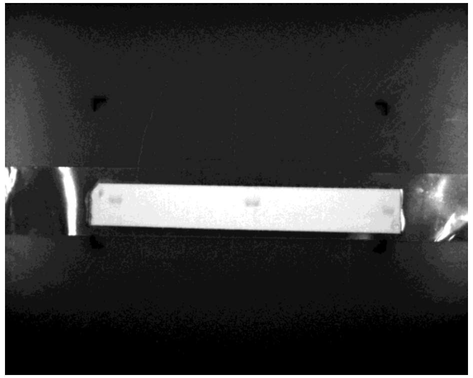

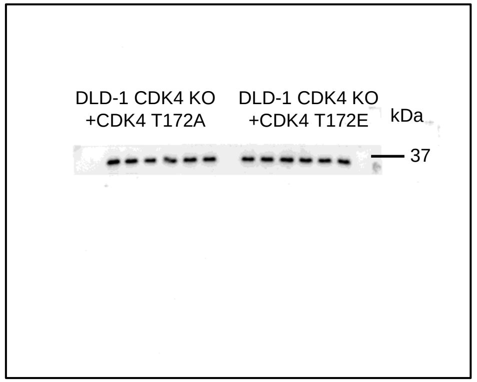

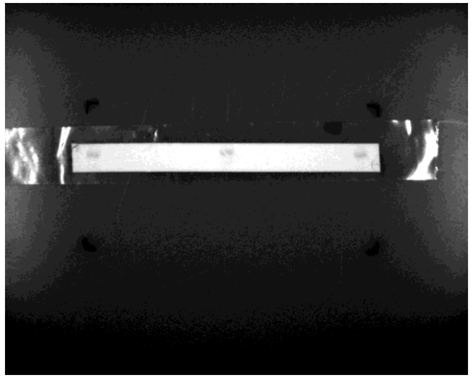

Full unedited blot for Figure 6A

V5-Ub

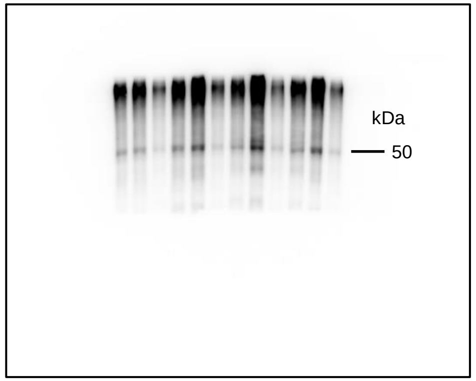

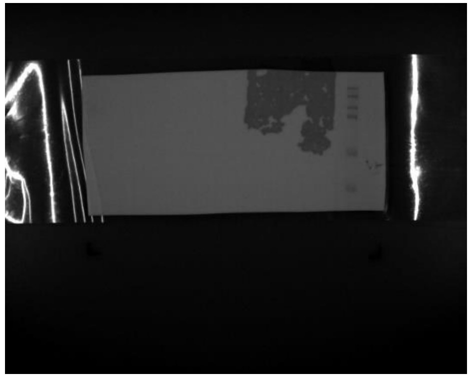

FLAG-CDK4

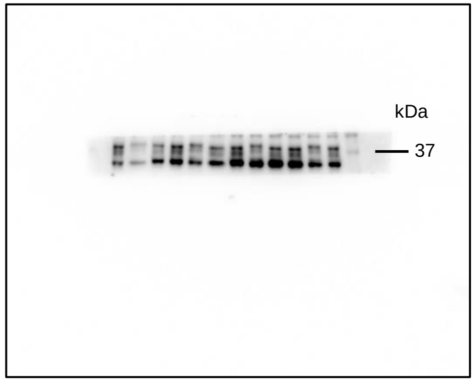

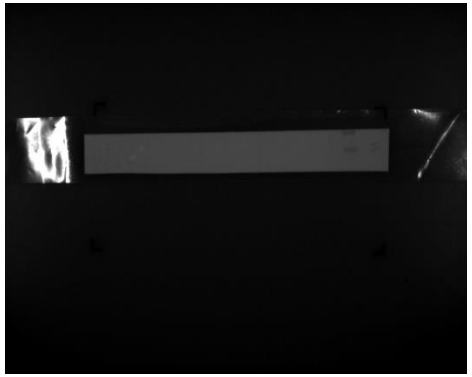

Full unedited blot for Figure 6A

HA-UBE3A/Trim28

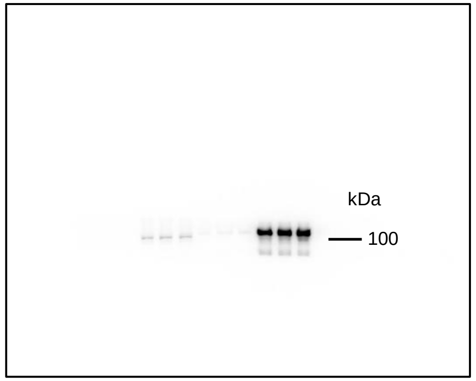

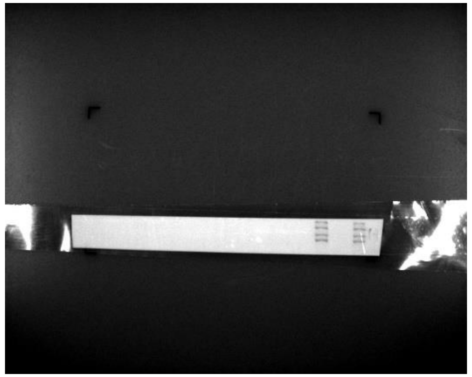

HA
-Stub1

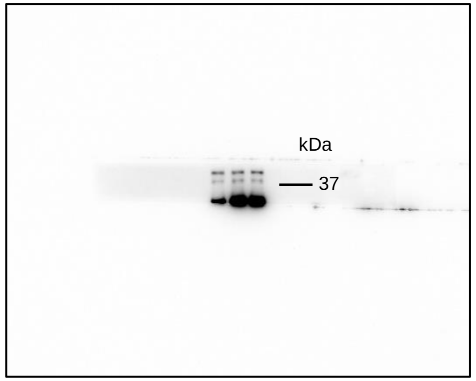

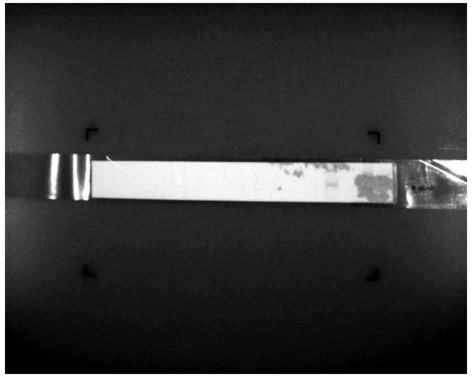

FLAG-CDK4

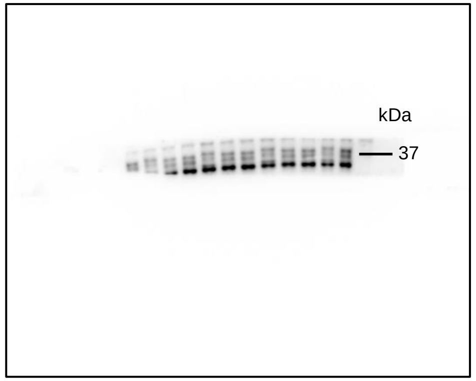

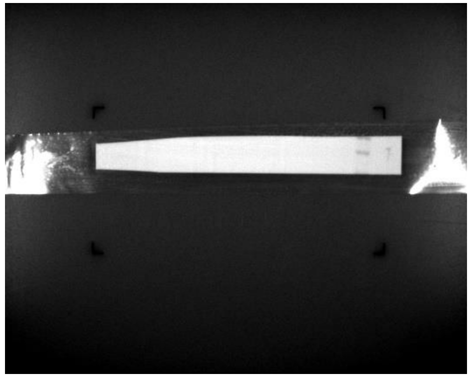

GAPDH

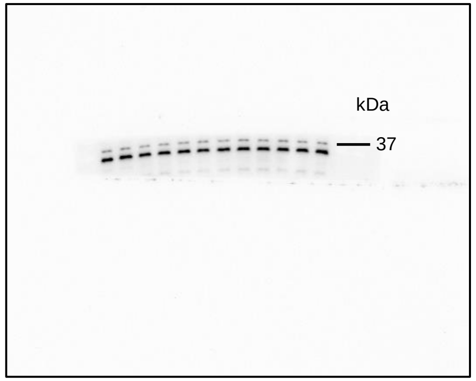

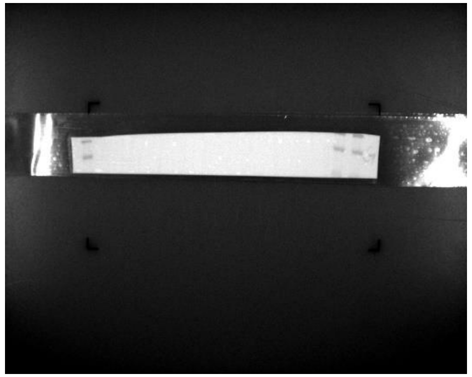

Full unedited blot for Figure 6B

HA

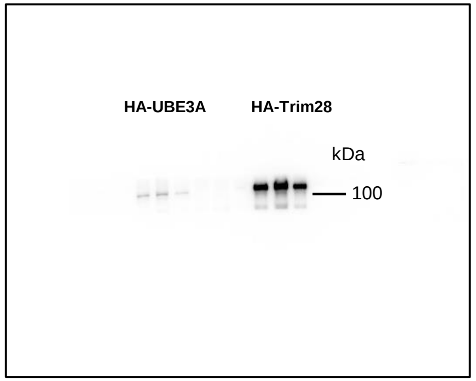

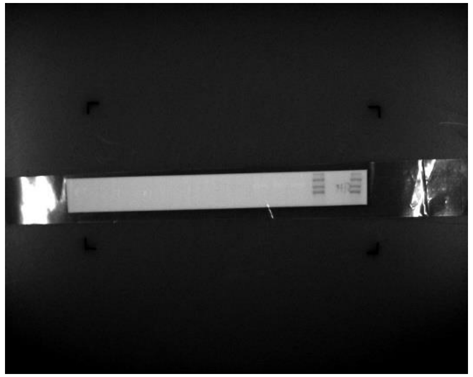

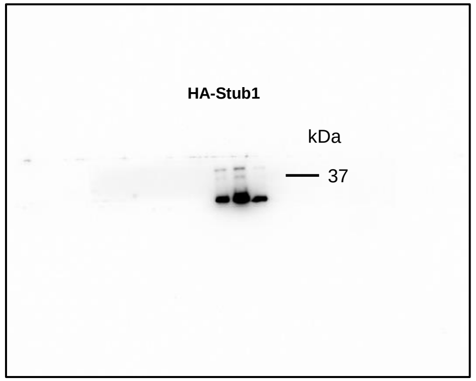

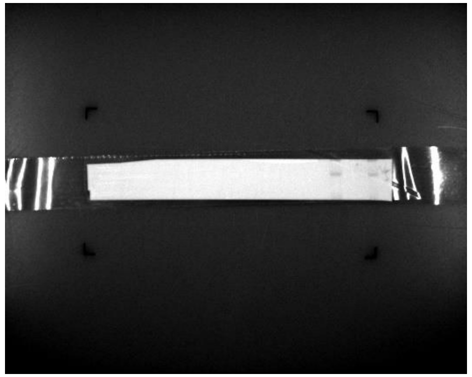

FLAG-
CDK4

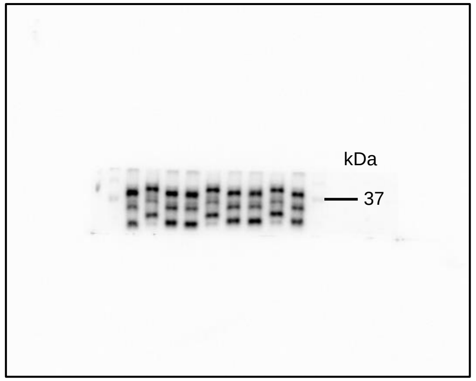

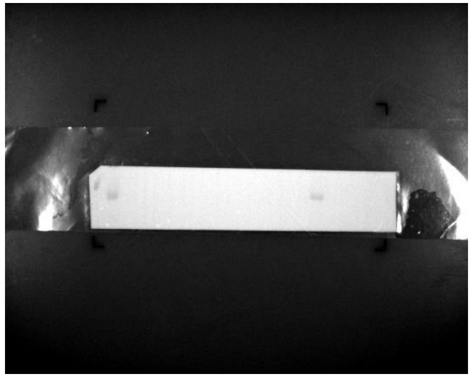

Full unedited blot for Figure 6B

HA

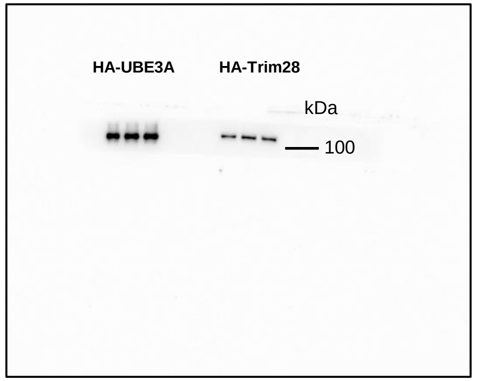

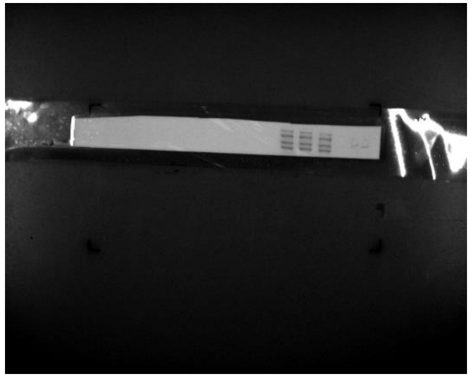

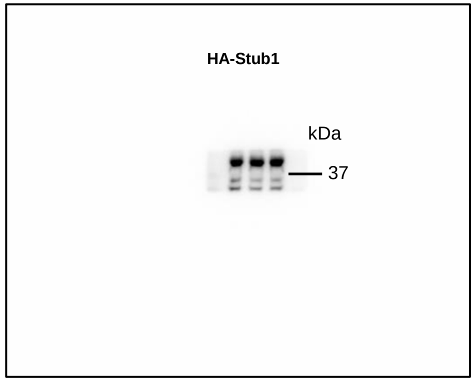

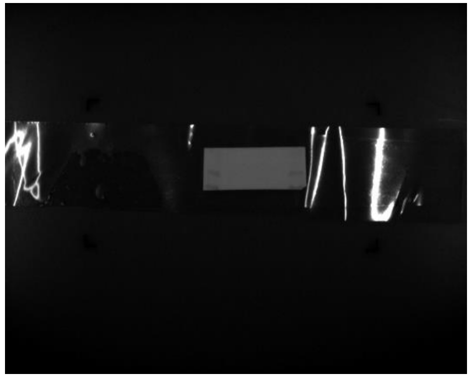

FLAG-
CDK4

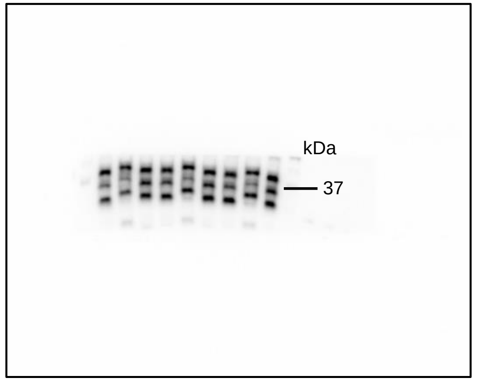

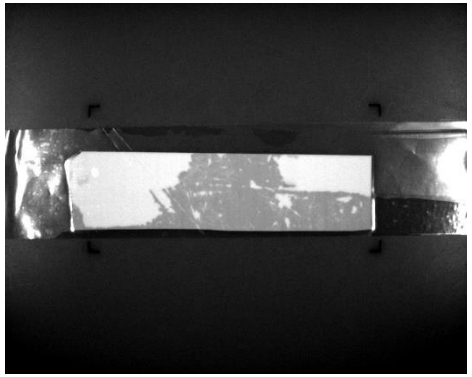

Full unedited blot for Figure 6C

Ub

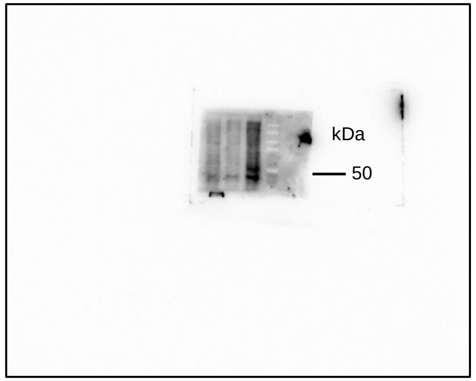

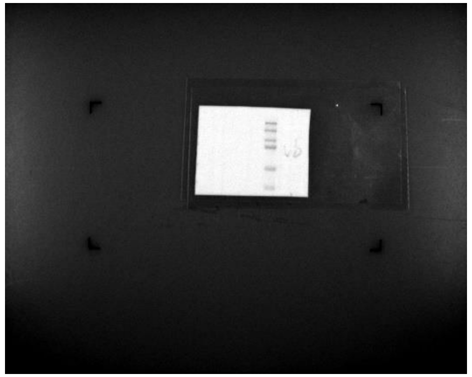

Trim28

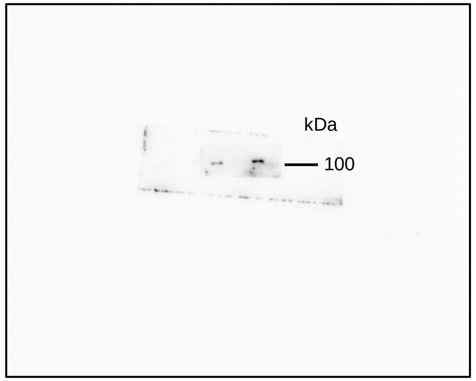

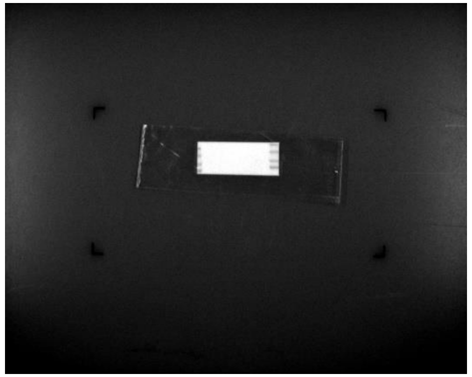

CDK4

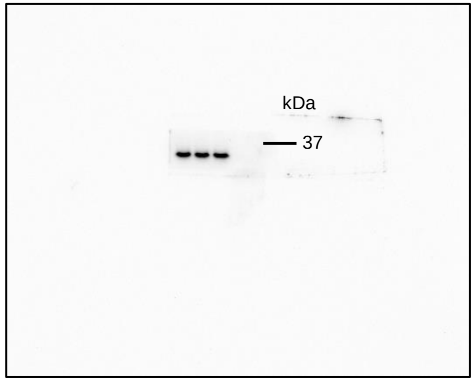

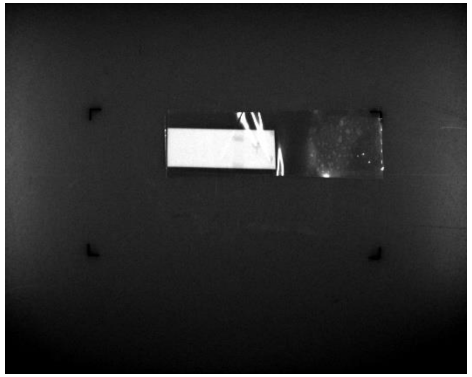

Full unedited blot for Figure 6C

Trim28

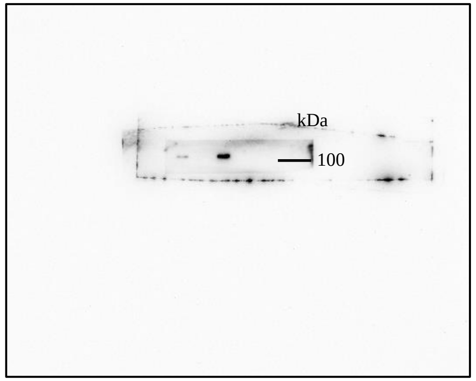

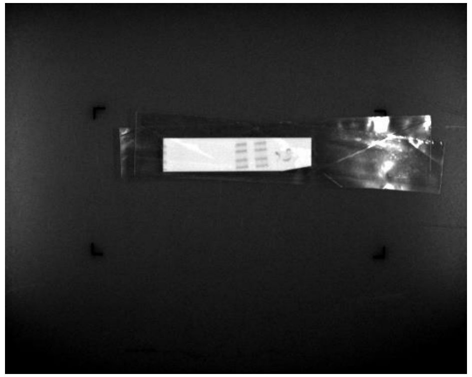

CDK4

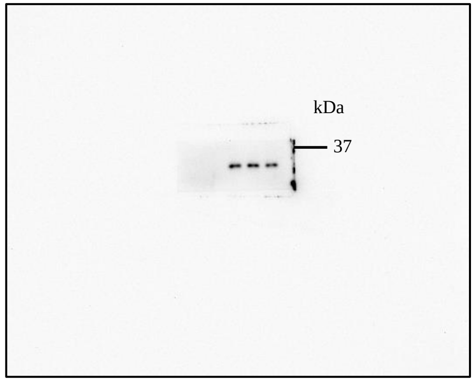

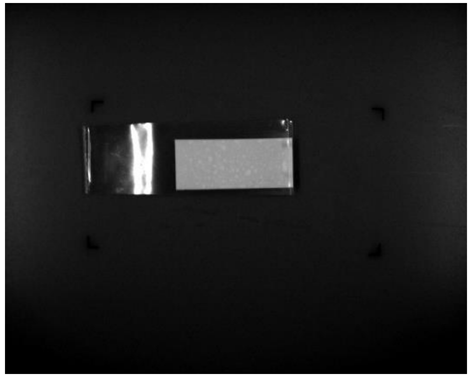

Full unedited blot for Figure 6D

V5-Ub

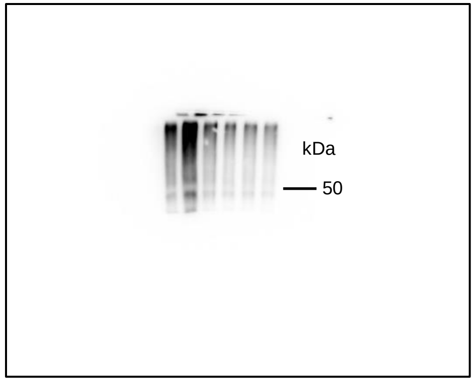

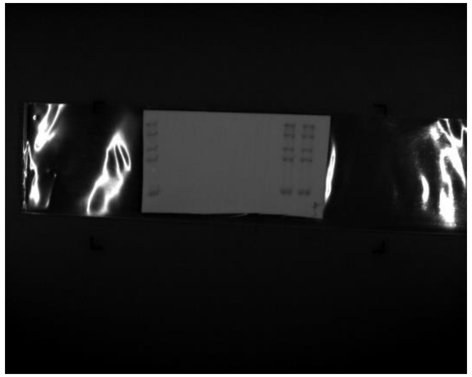

FLAG
-CDK4

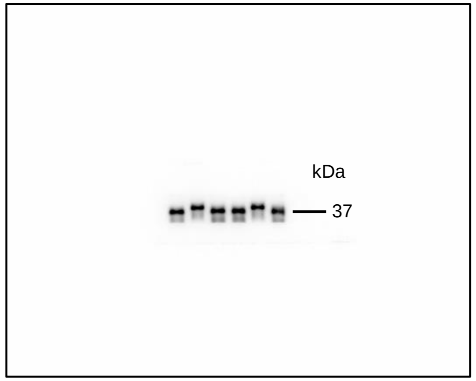

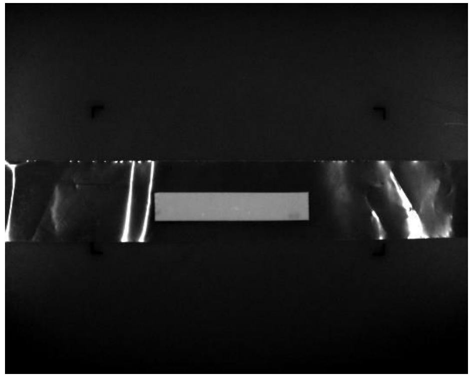

Full unedited blot for Figure 6D

Trim28

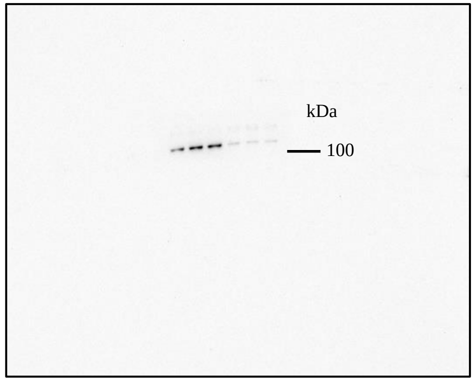

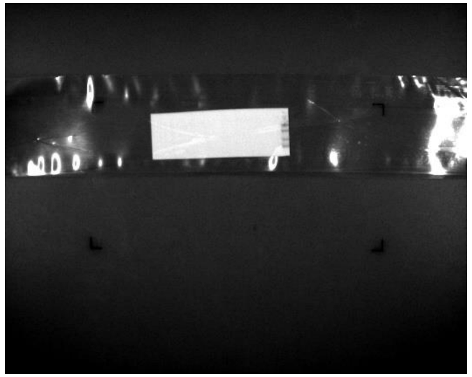

FLAG
-CDK4

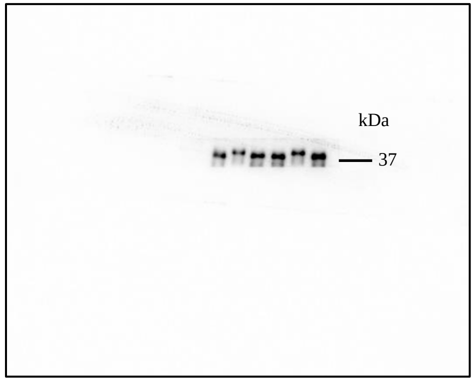

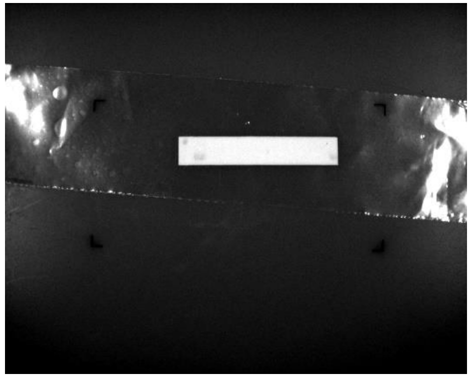

GAPDH

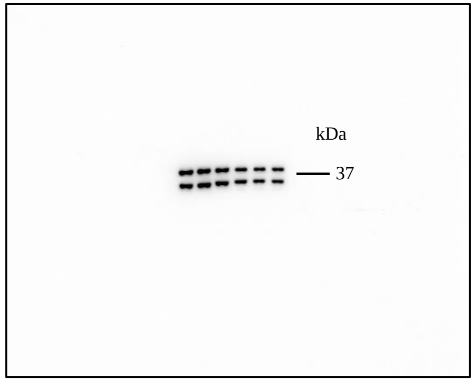

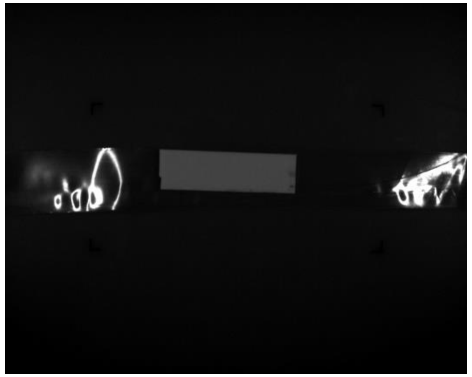

Full unedited blot for Figure 6E

V5-Ub

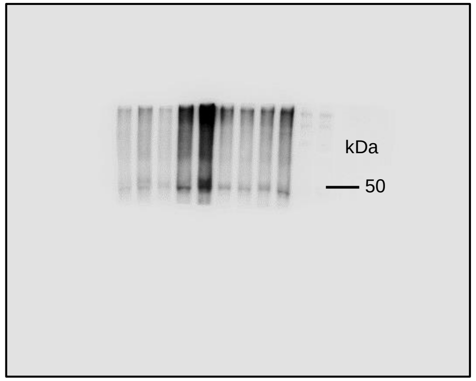

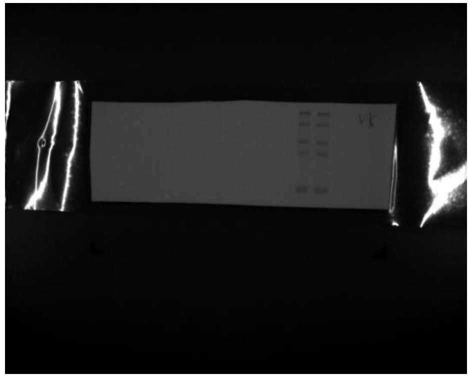

FLAG
-CDK4

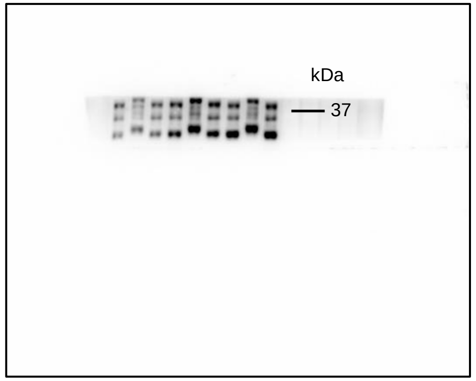

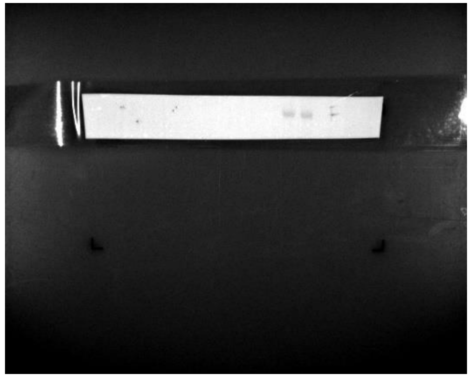

Full unedited blot for Figure 6E

HA-
Trim28

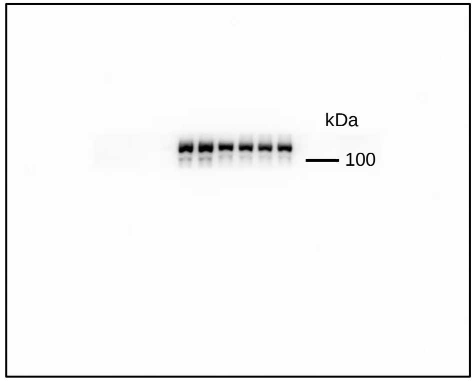

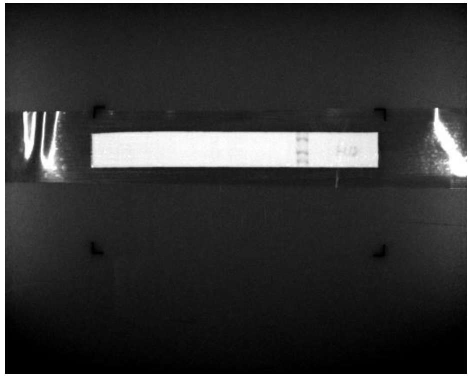

FLAG
-CDK4

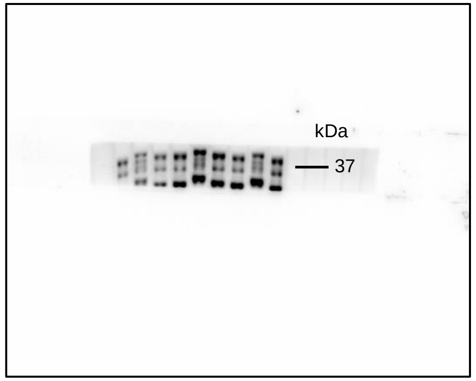

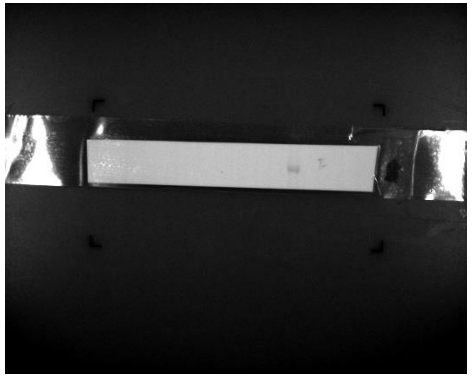

α-tubulin

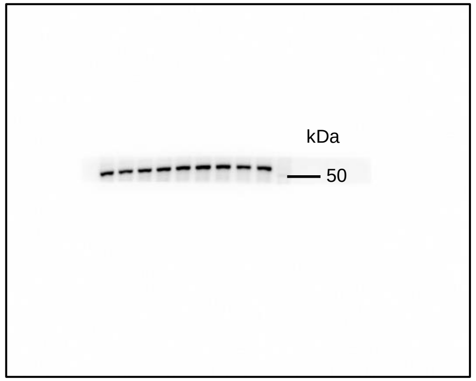

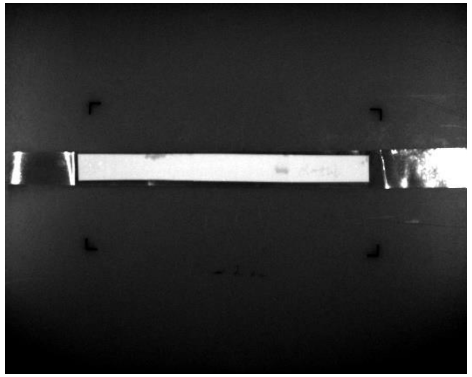

Full unedited blot for Figure 6F

Ub

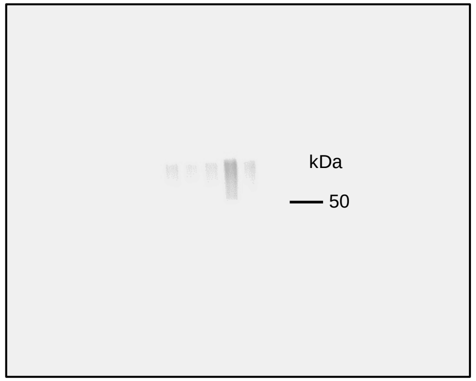

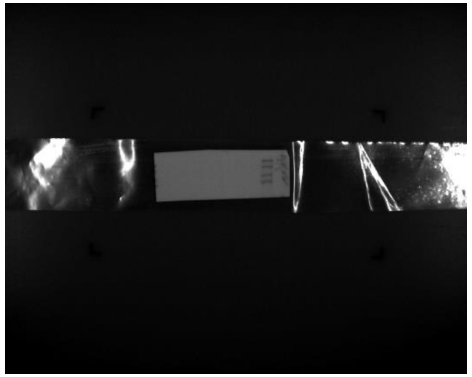

GST

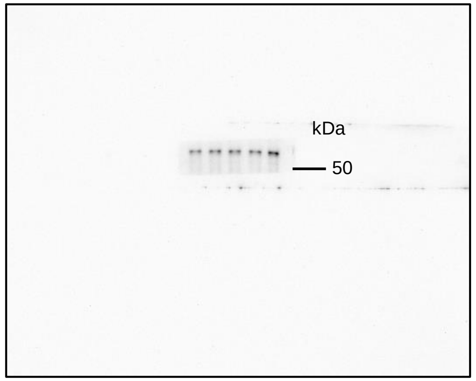

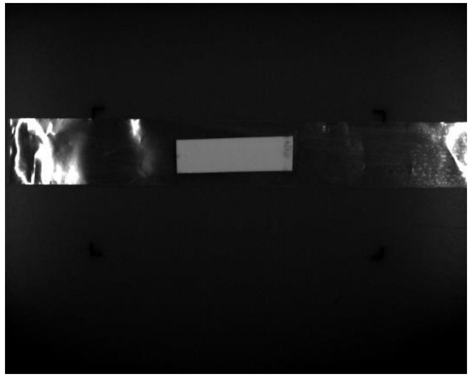

Full unedited blot for Figure 7B

pRb

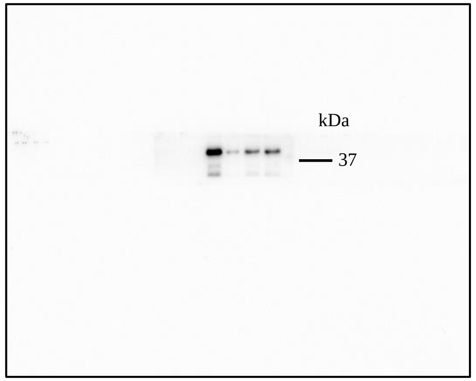

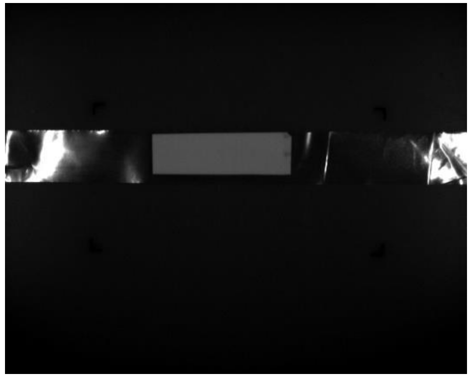

GST

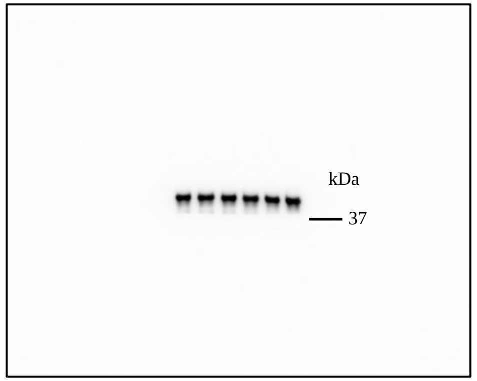

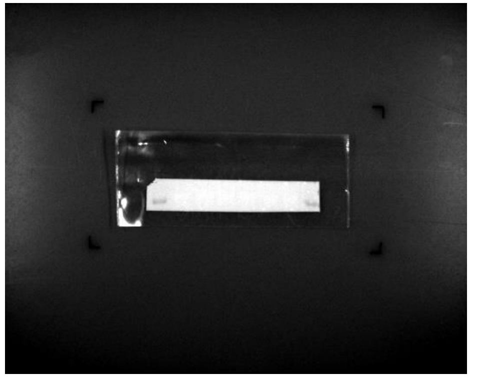

Full unedited blot for Figure 7C

pRb

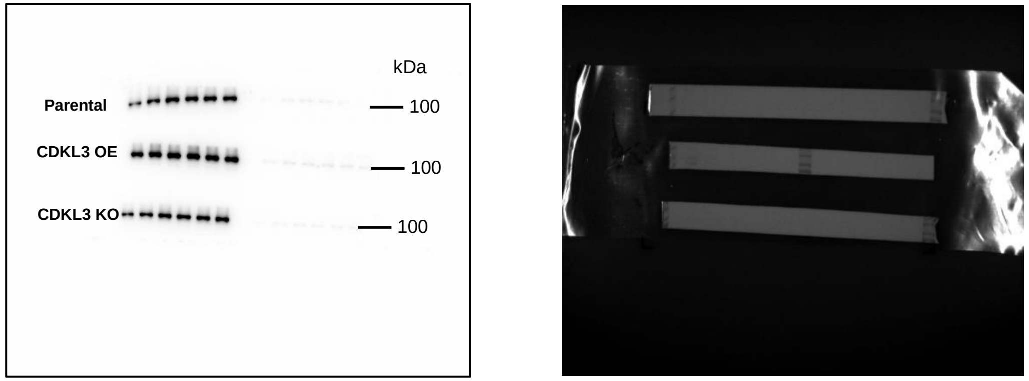

Rb

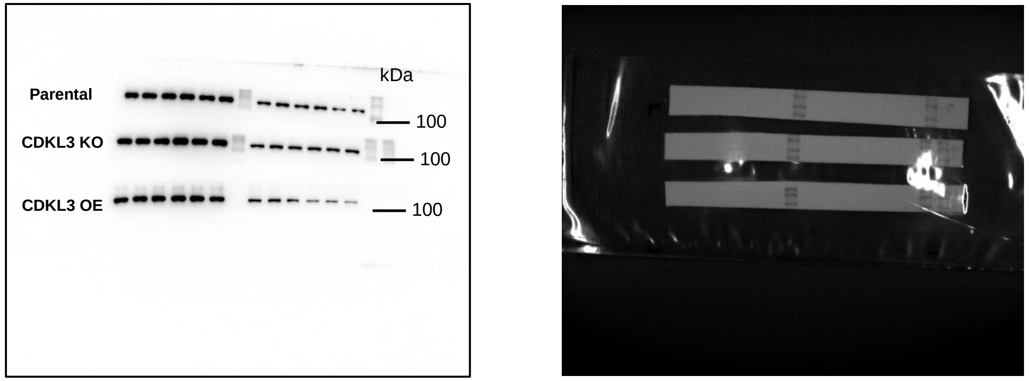

CDKL3

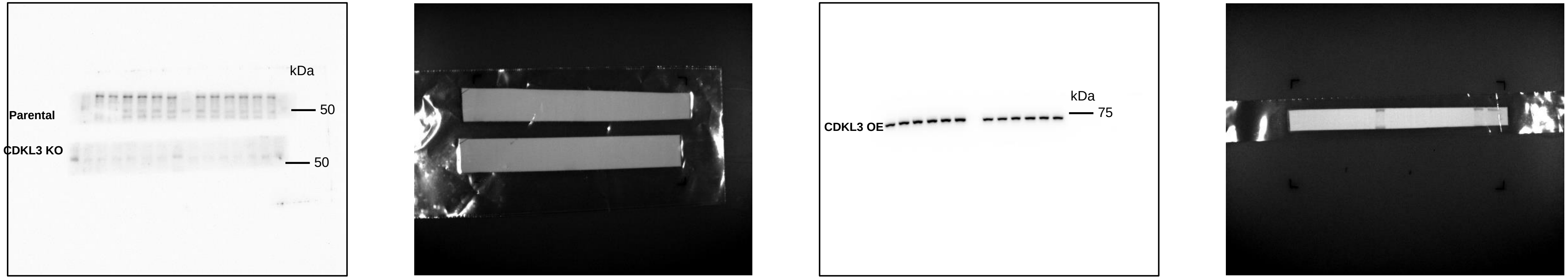

Full unedited blot for Figure 7C

cyclinA2

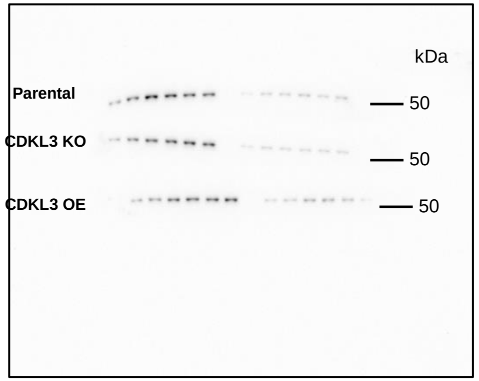

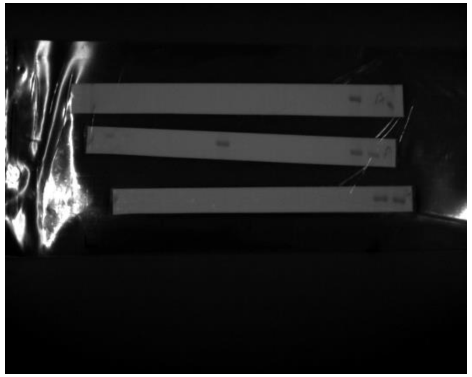

cyclinD1

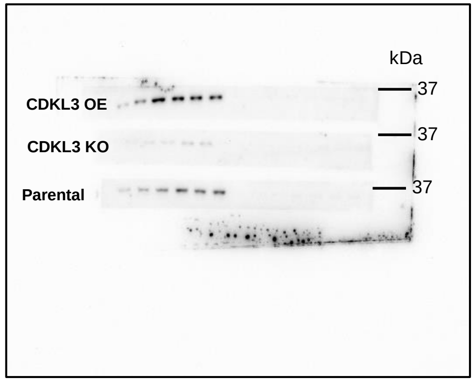

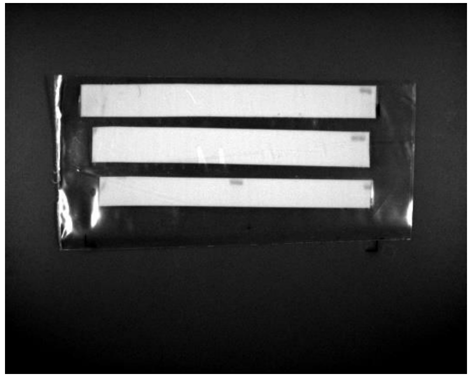

(GAPDH in Fig.7E)

Full unedited blot for Figure 7D

pRb

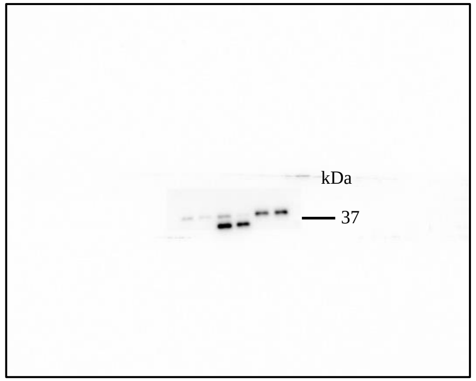

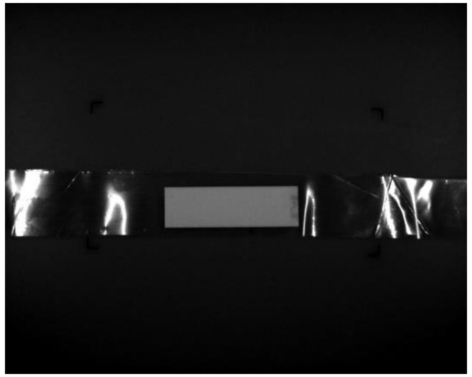

GST

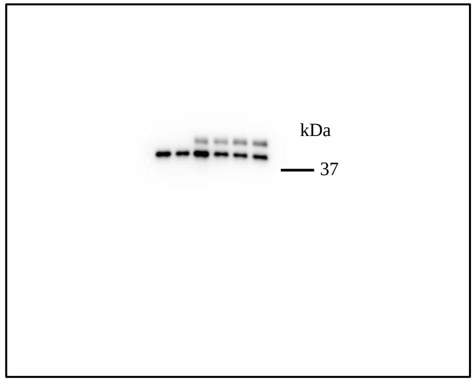

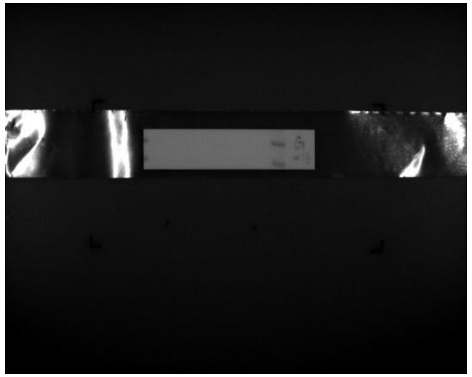

Full unedited blot for Figure 7E

pRb

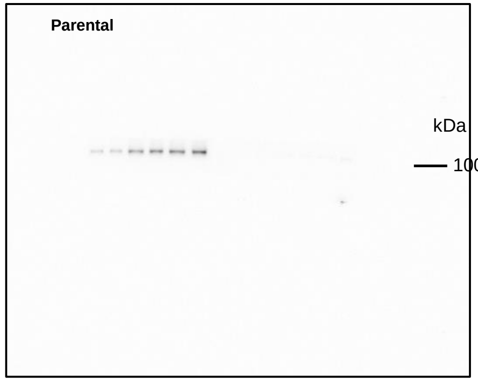

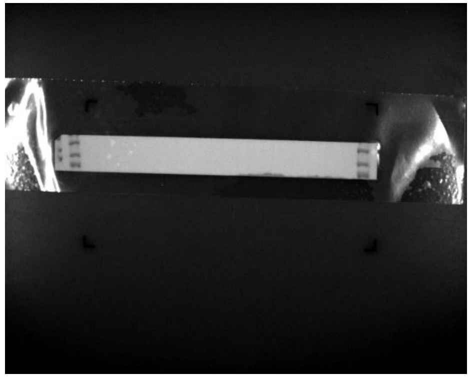

pRb

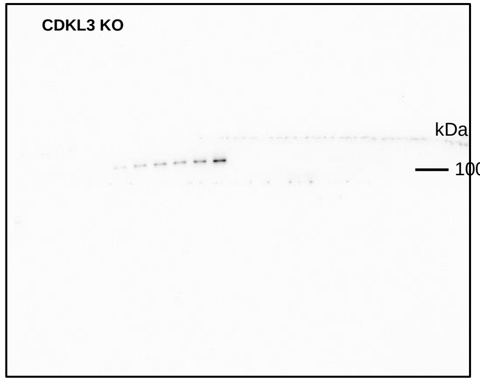

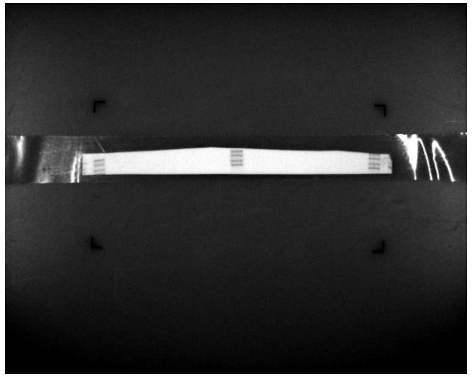

pRb

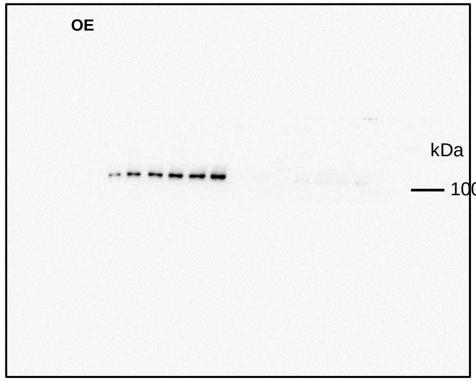

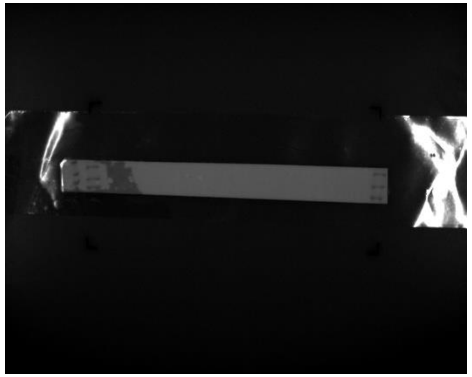

Rb

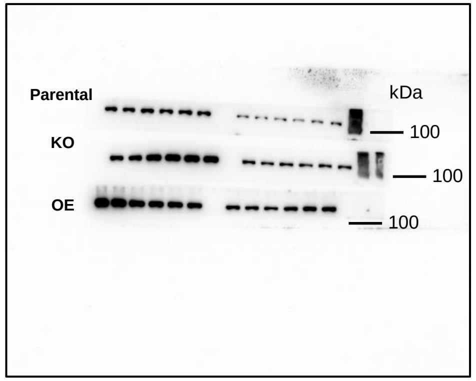

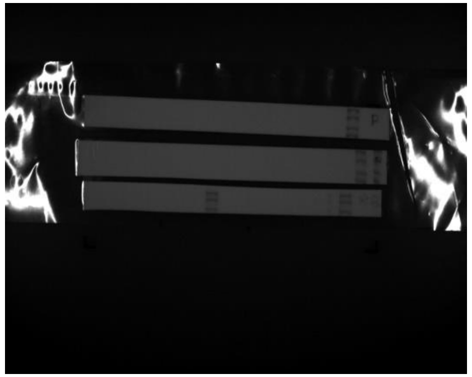

Full unedited blot for Figure 7E

CDKL3

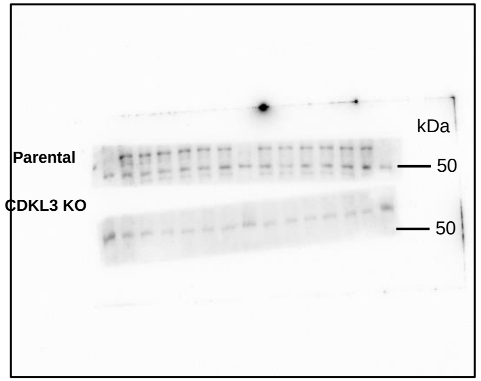

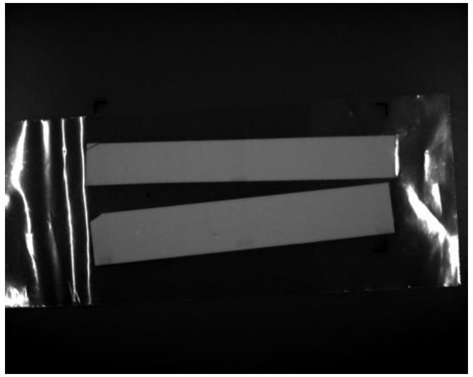

FLAG
-CDKL3

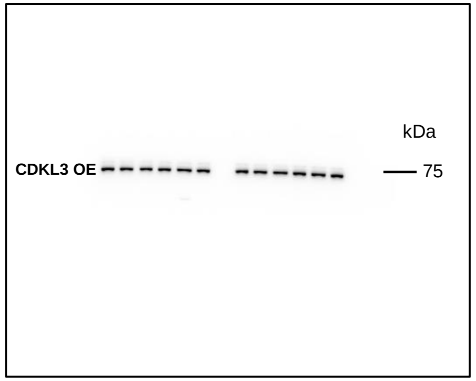

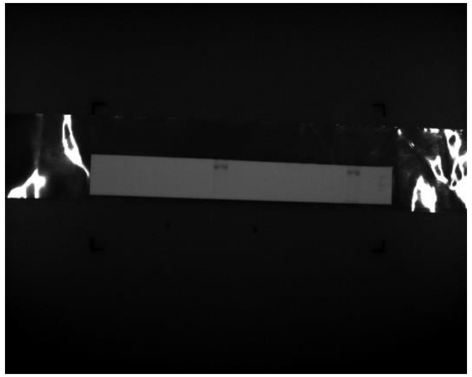

cyclinA2

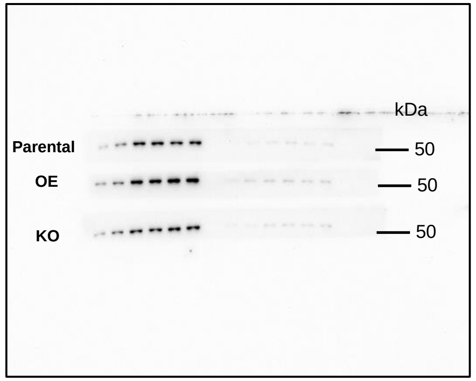

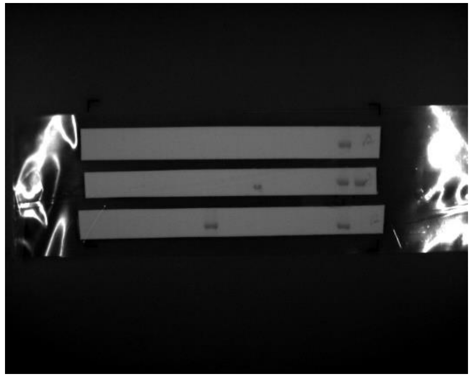

cyclinD1

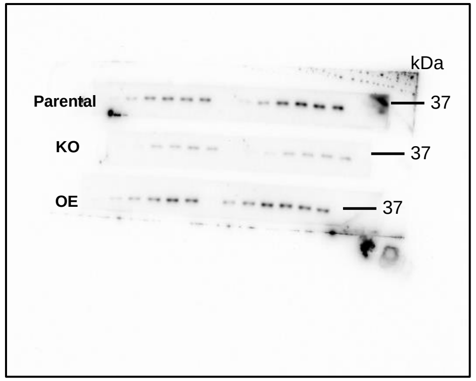

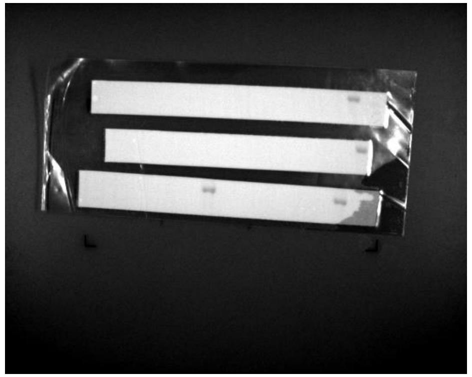

Full unedited blot for Figure 7E

CDK4

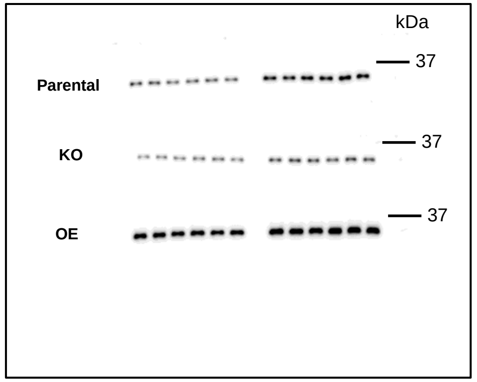

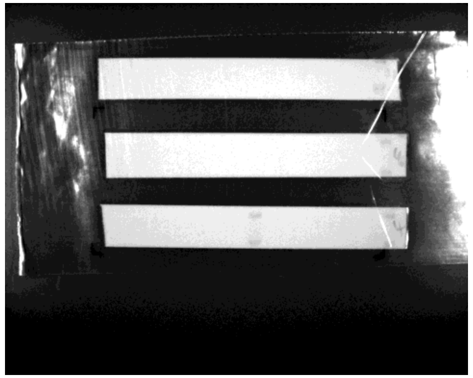

GAPDH

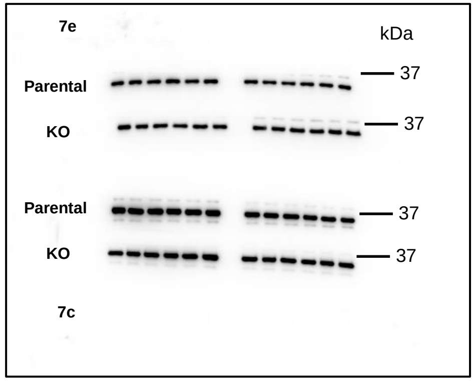

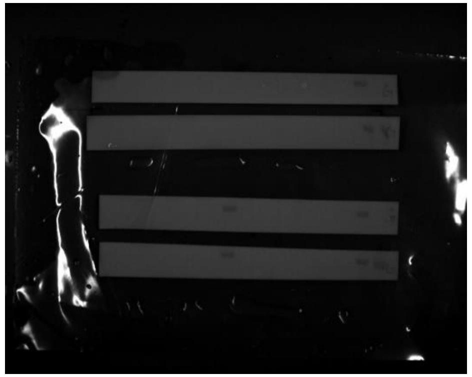

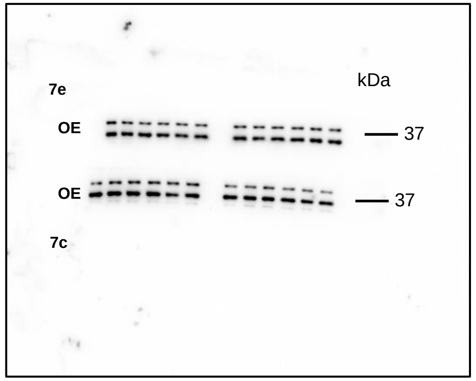

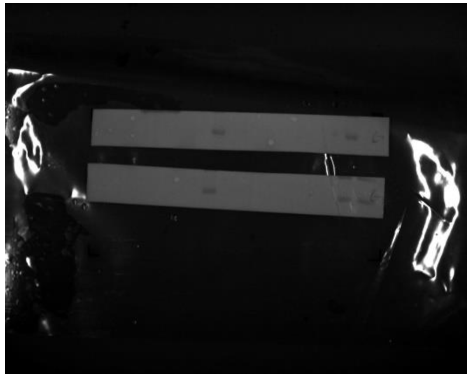

Full unedited blot for Figure 7F

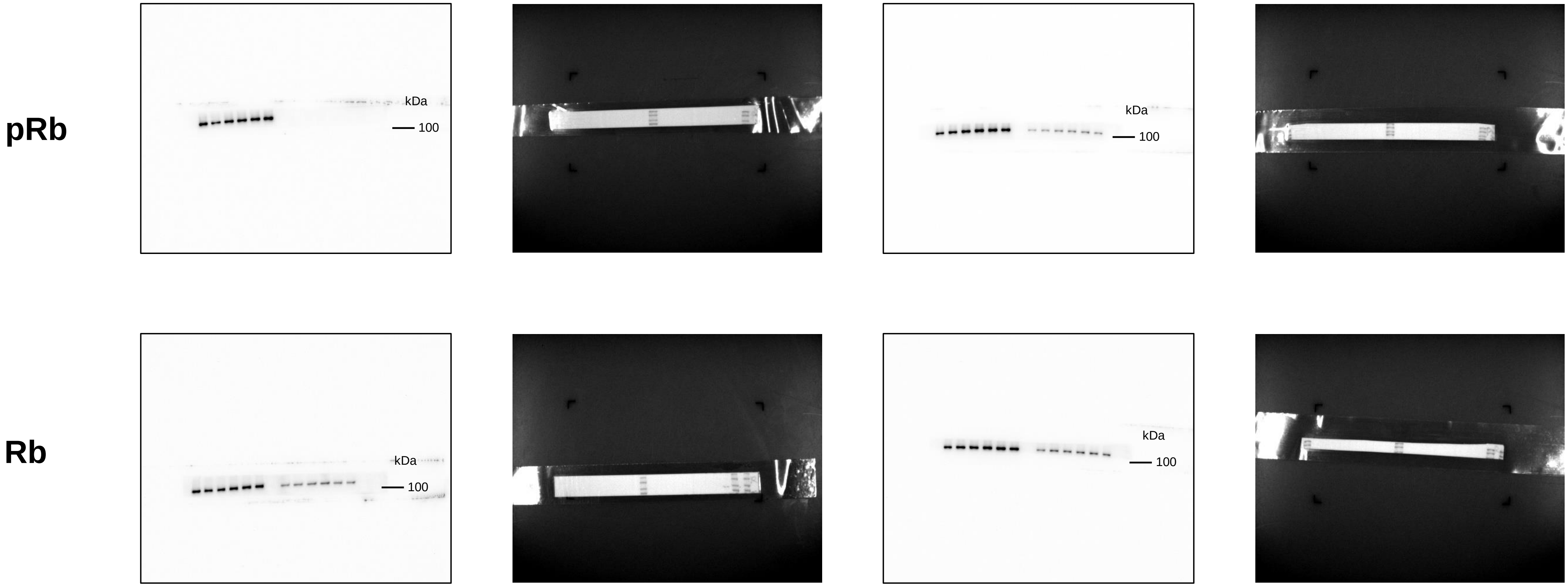

Full unedited blot for Figure 7F

FLAG
-CDKL3

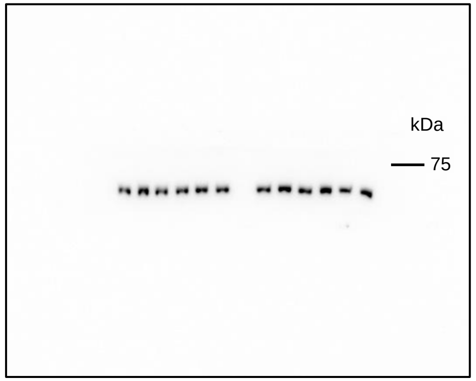

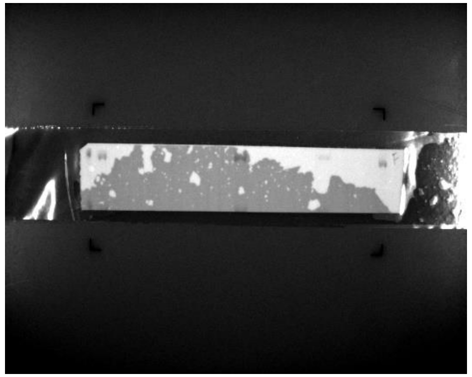

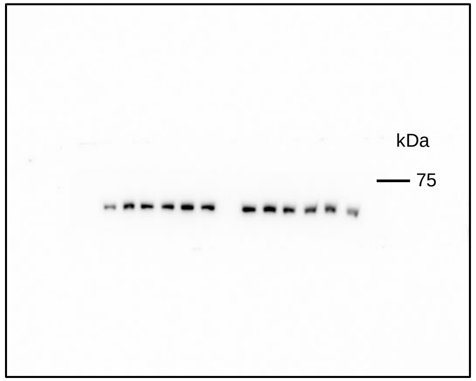

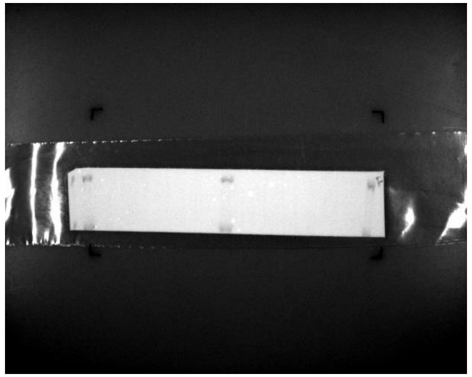

CDK4

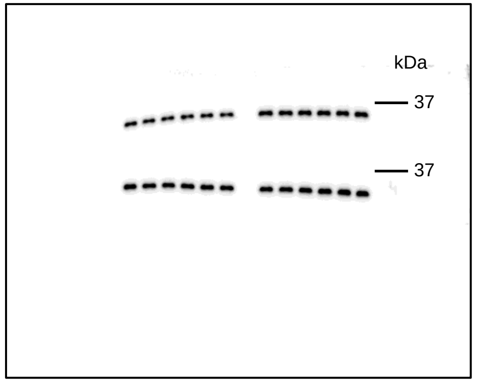

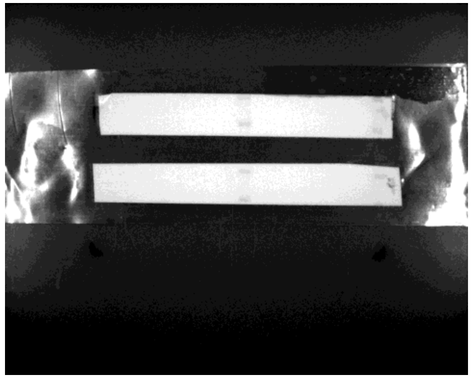

Full unedited blot for Figure 7F

cyclinA2

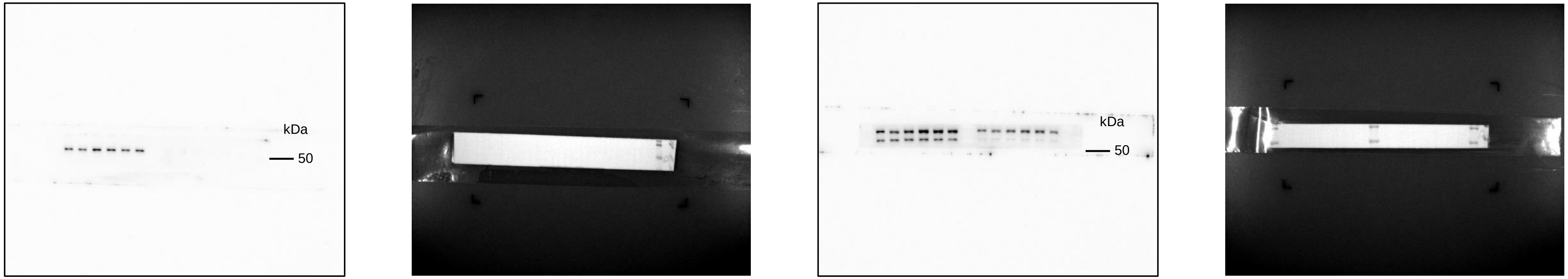

GAPDH

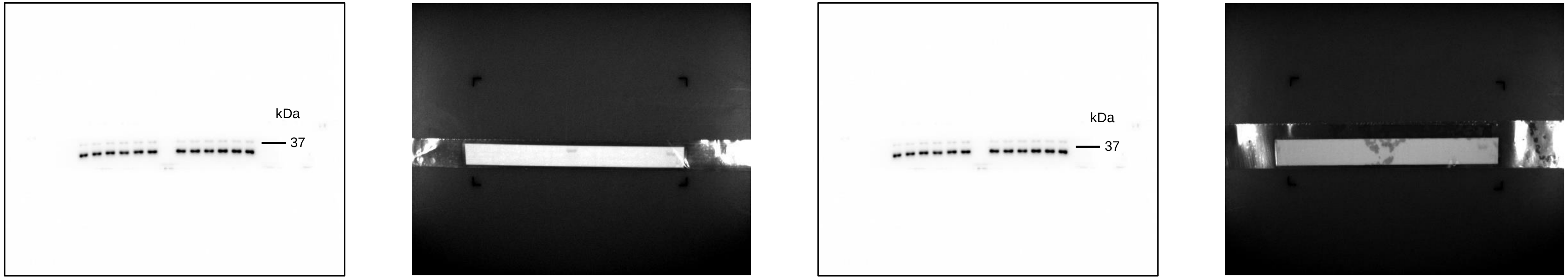

Full unedited blot for Figure 8B

pRb

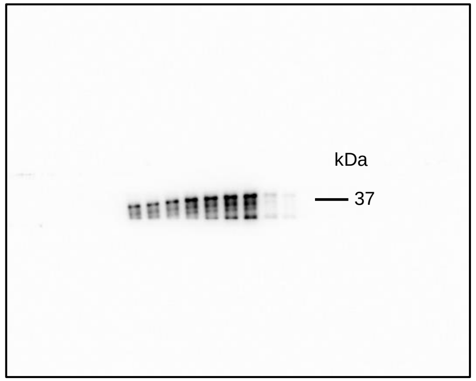

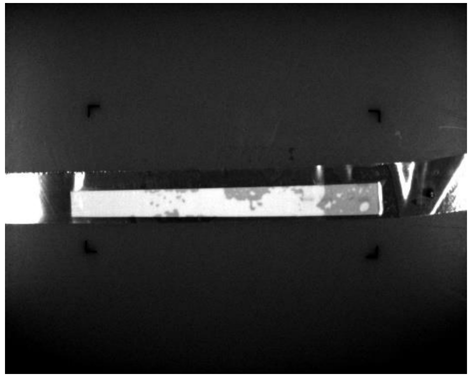

GST

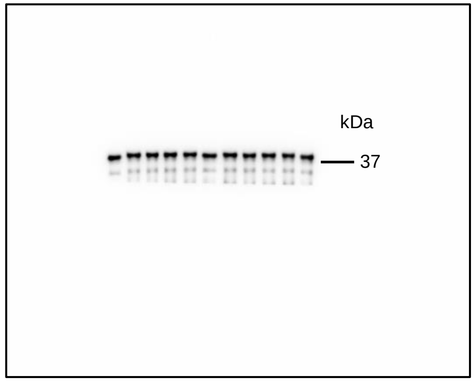

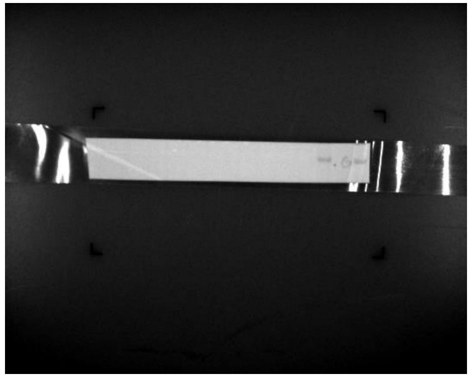

Full unedited blot for Figure 8K

pRb

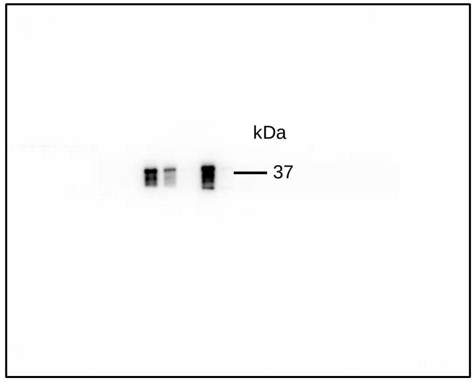

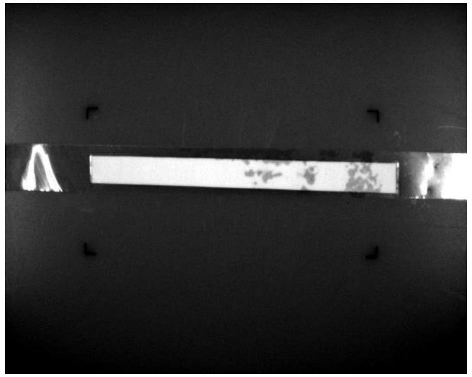

GST

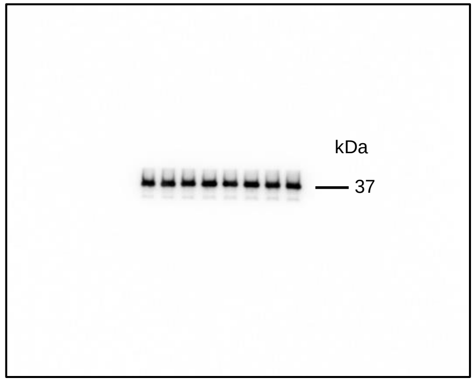

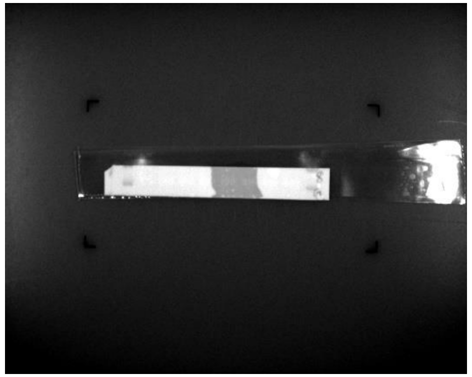

Full unedited blot for Figure 8N

CDKL3

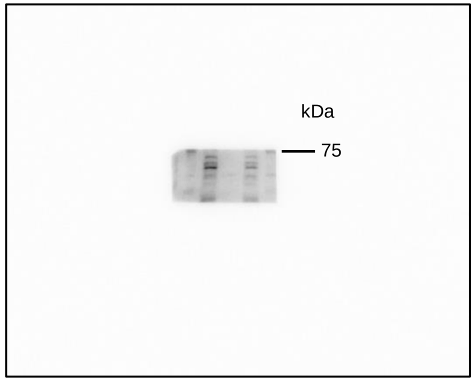

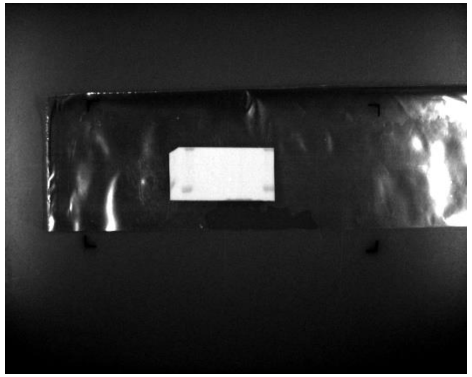

cyclinA2

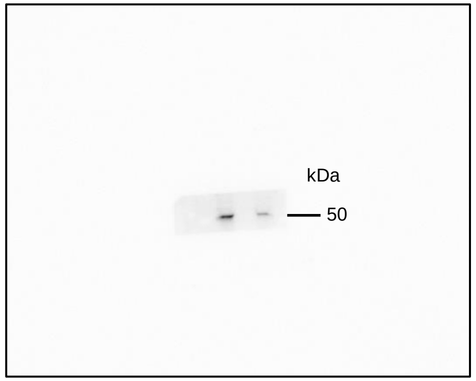

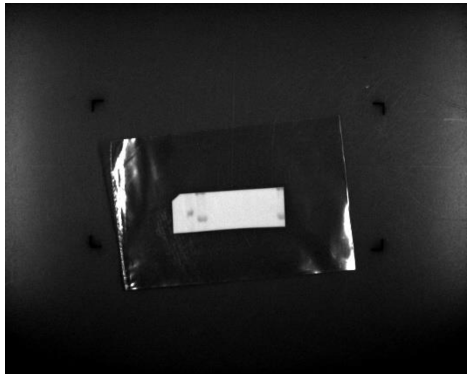

CDK6

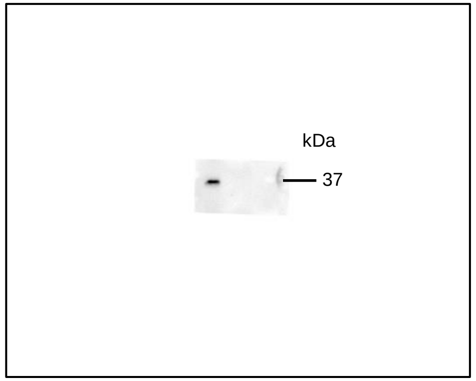

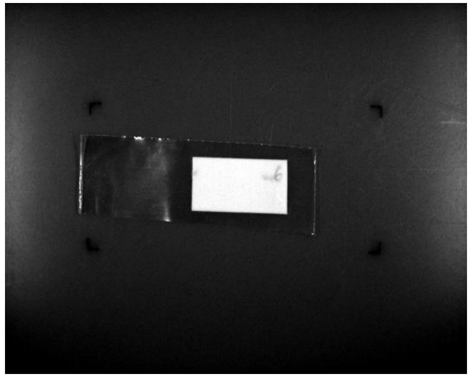

CDK2

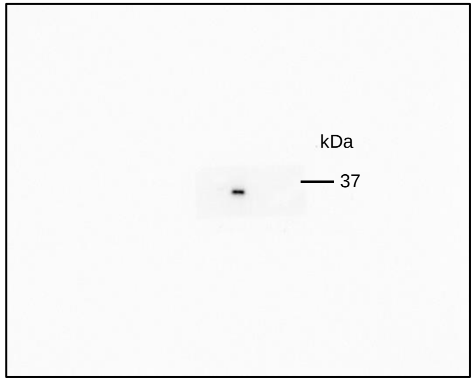

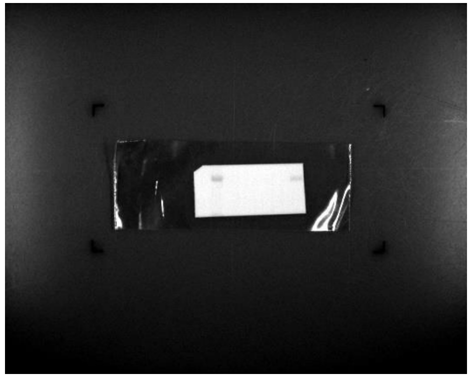

Full unedited blot for Figure 8N

CDK4

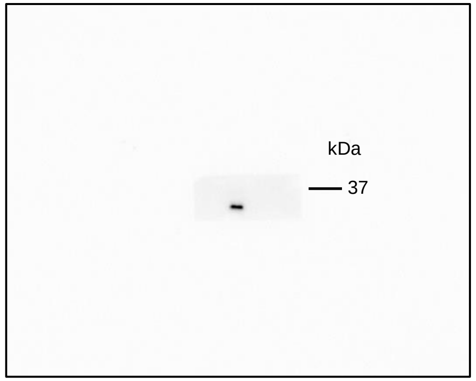

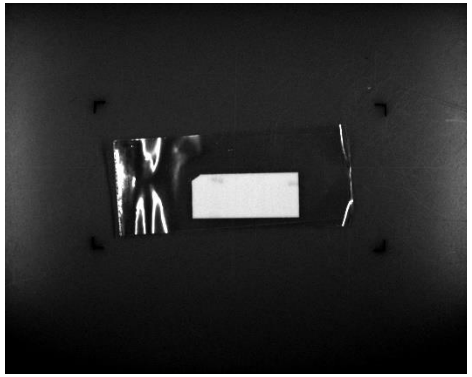

GAPDH

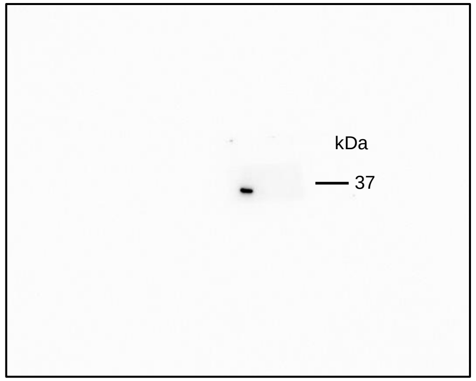

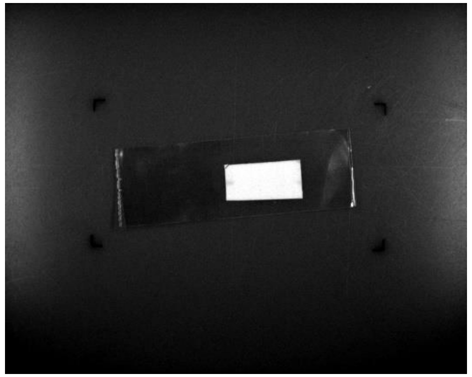

Full unedited blot for Figure 9A

pRb

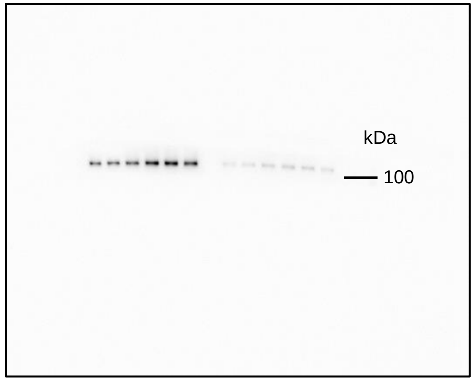

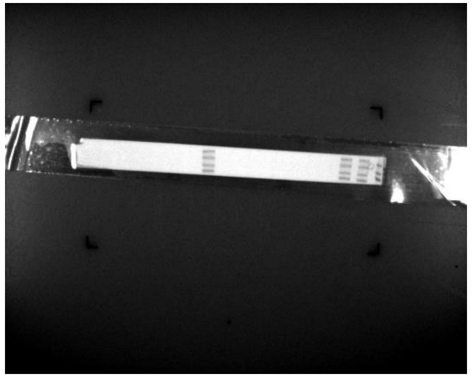

Rb

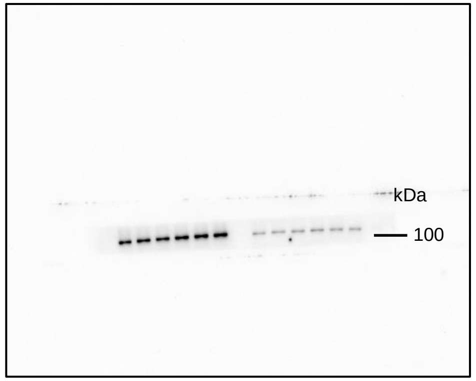

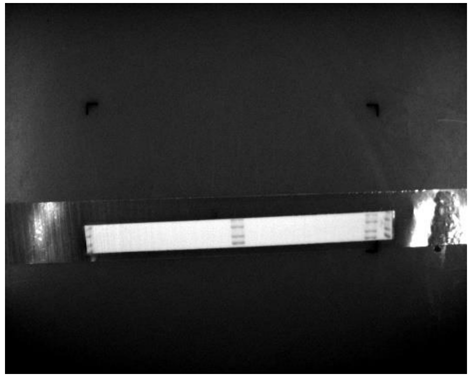

Full unedited blot for Figure 9A

cyclinA2

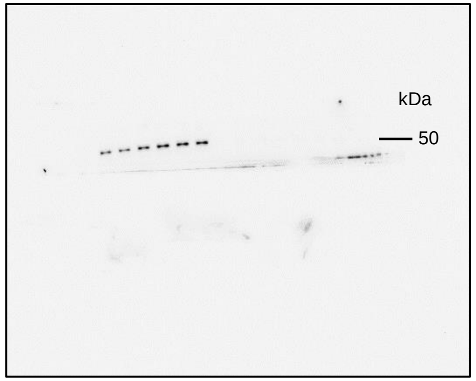

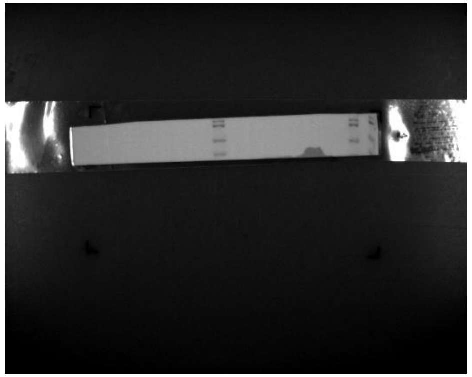

CDK4

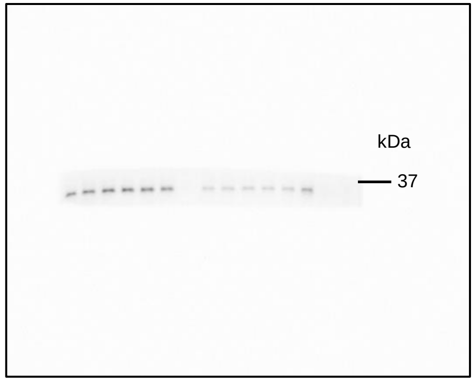

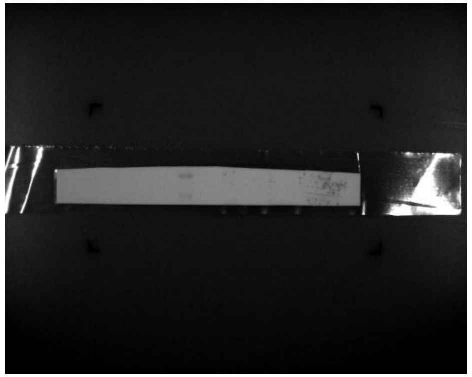

GAPDH

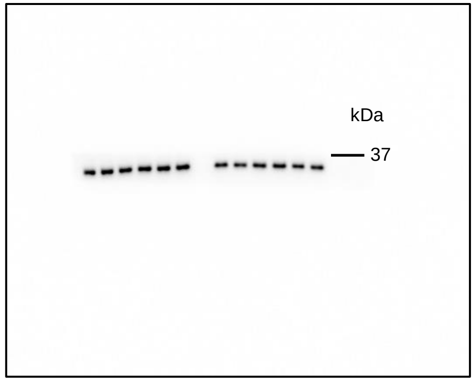

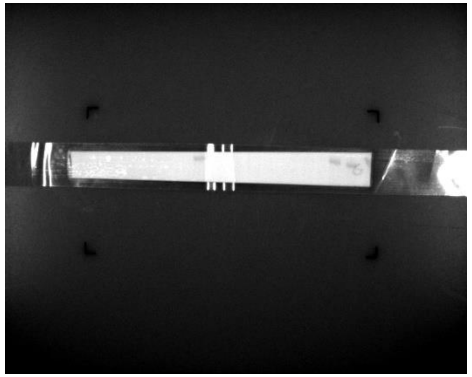

Full unedited blot for Figure 10G

pRb

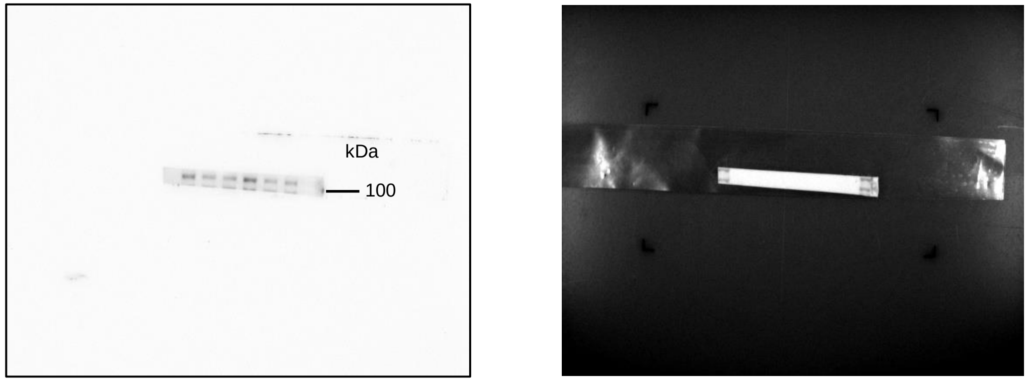

Rb

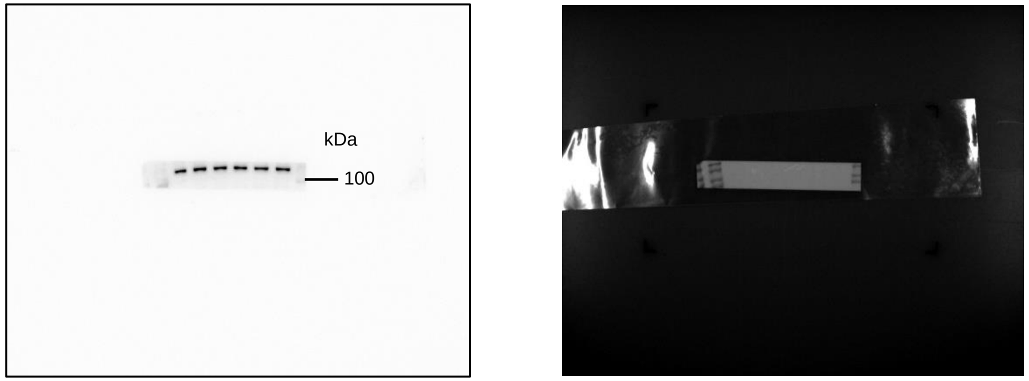

CDKL3

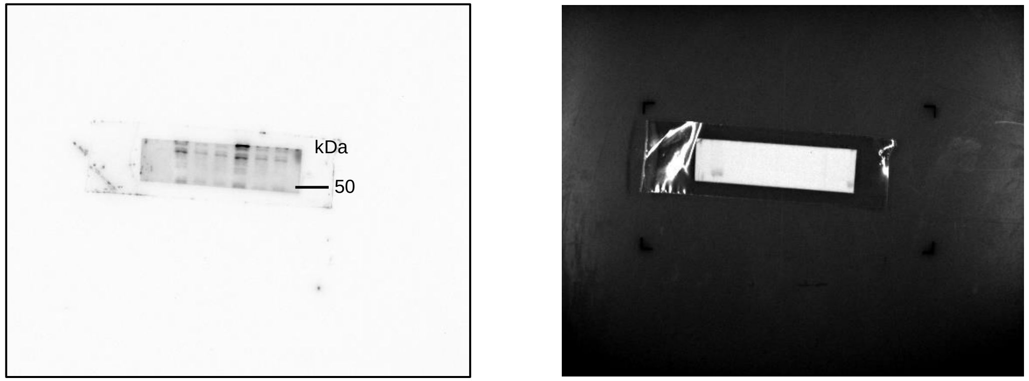

Full unedited blot for Figure 10G

CDK4

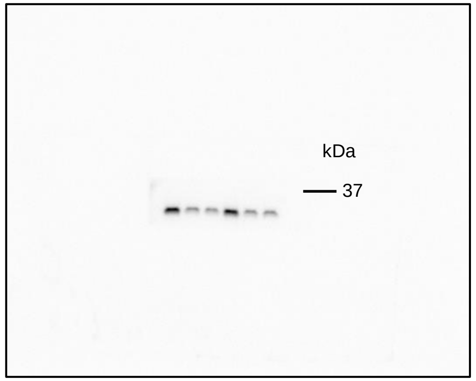

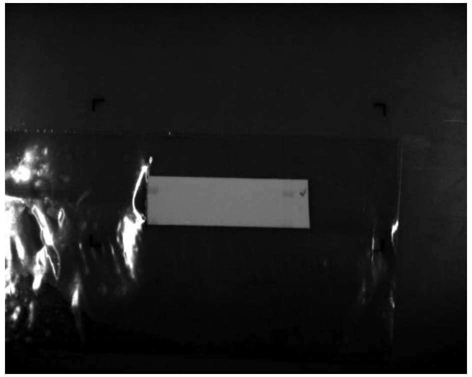

GAPDH

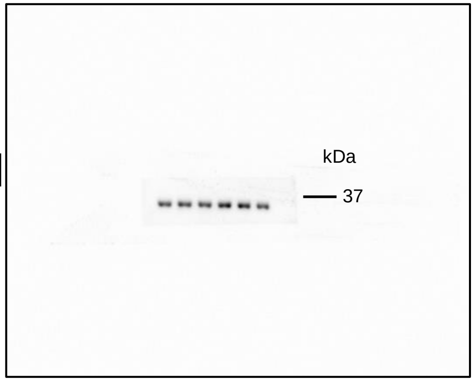

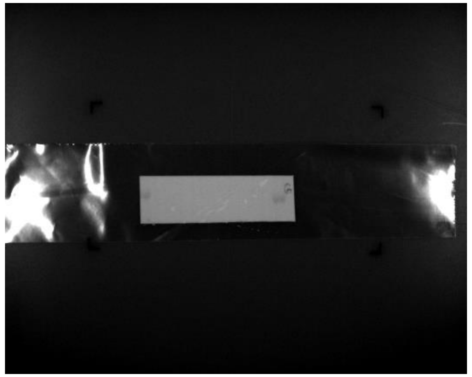

Full unedited blot for Figure 10N

pRb

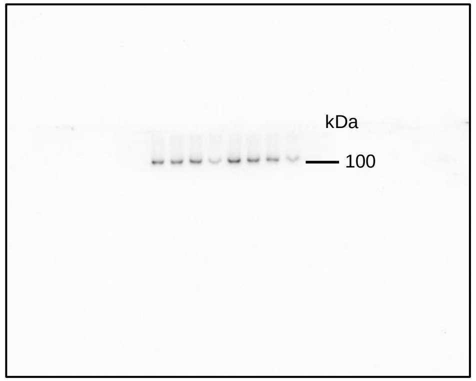

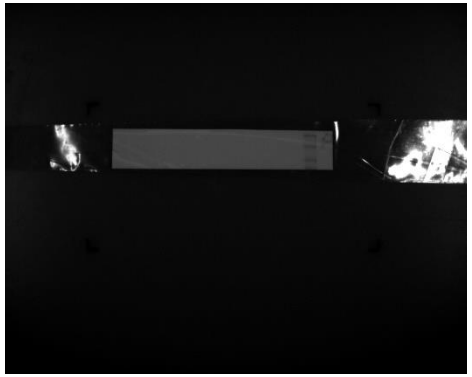

Rb

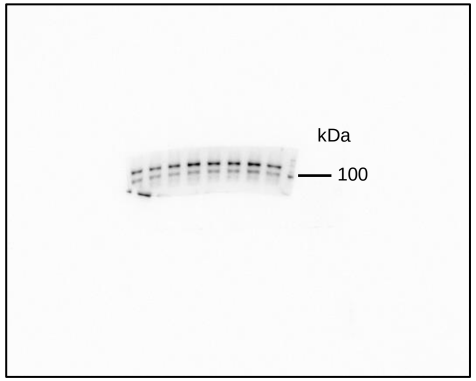

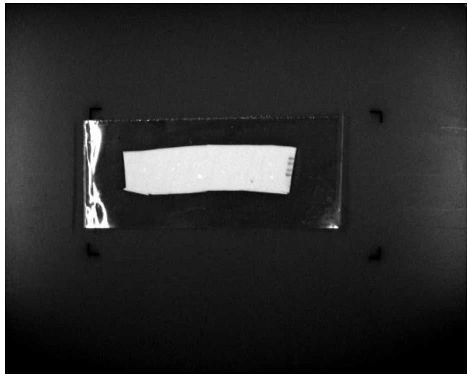

CDK4

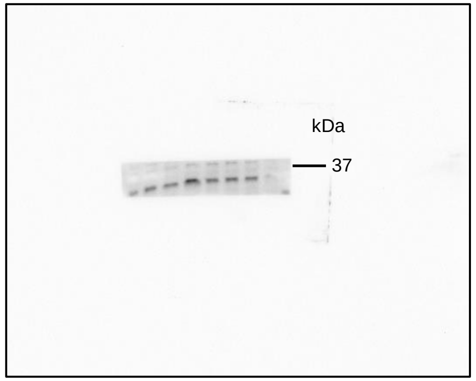

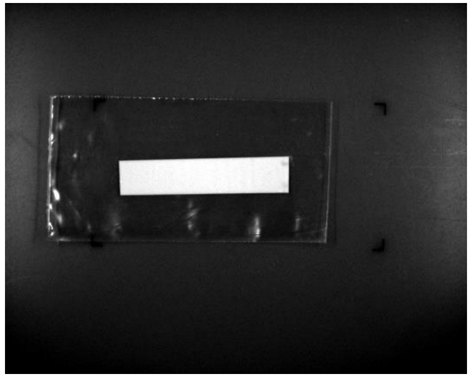

α-tubulin

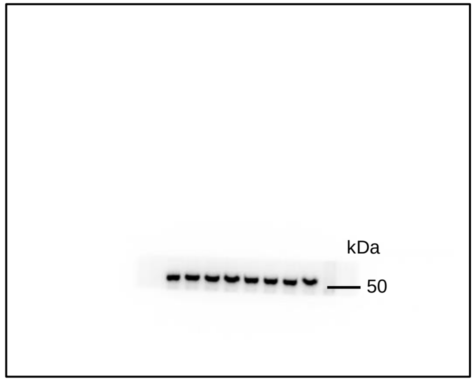

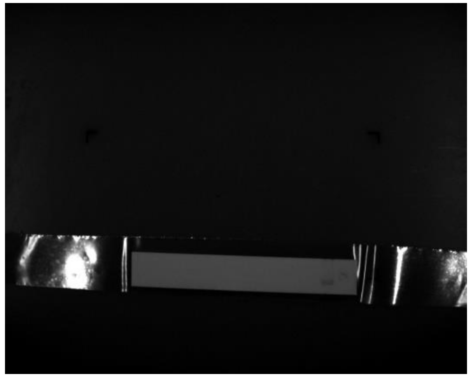

Full unedited blot for Supplemental Figure 1E

CDKL3

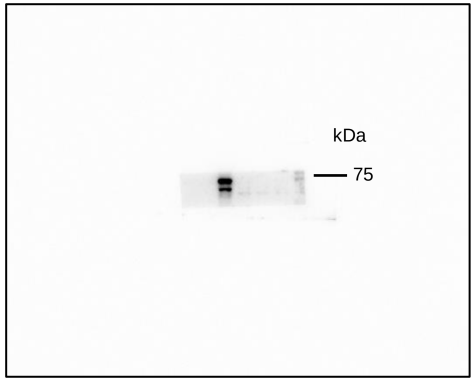

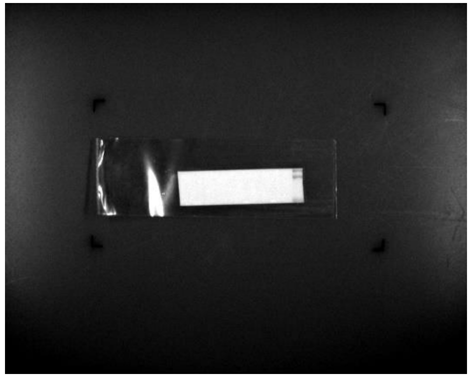

GAPDH

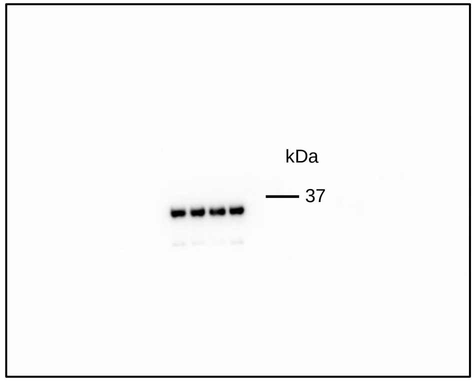

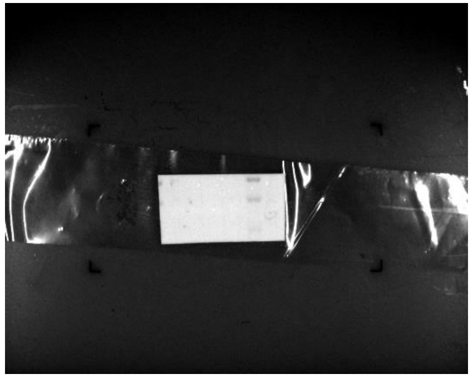

Full unedited blot for Supplemental Figure 1F

CDKL3

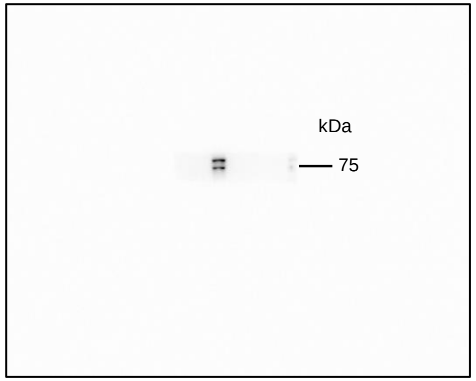

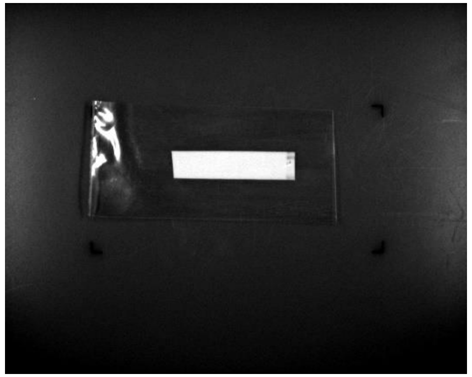

GAPDH

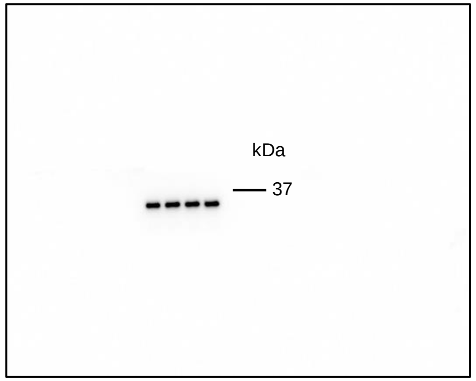

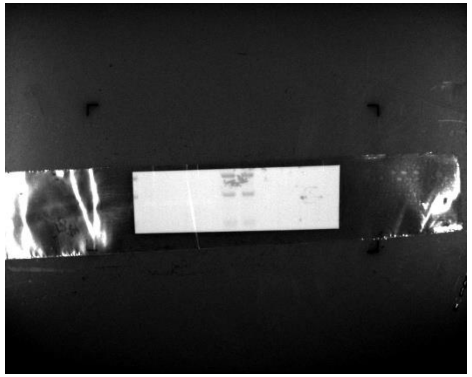

Full unedited blot for Supplemental Figure 1G

CDKL3

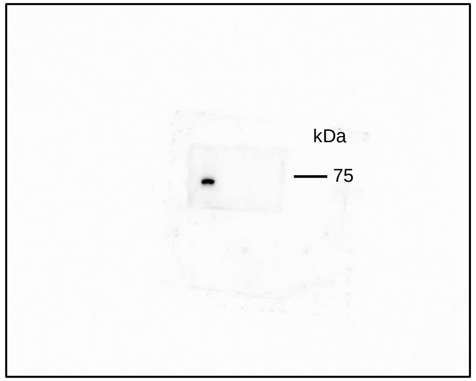

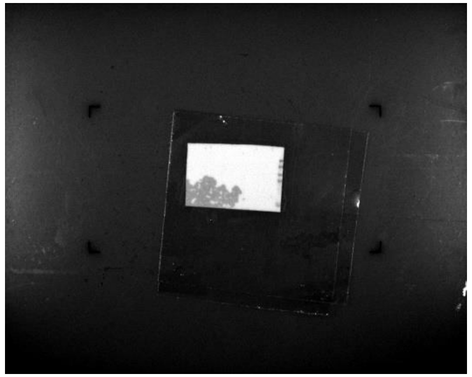

GAPDH

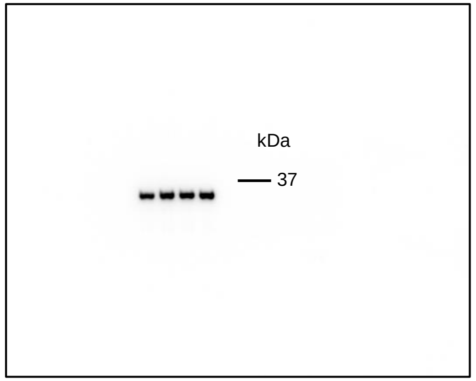

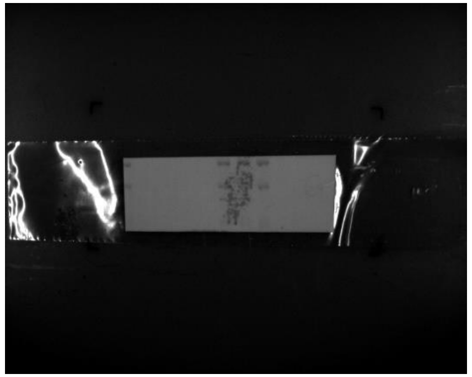

Full unedited blot for Supplemental Figure 1P

CDKL3

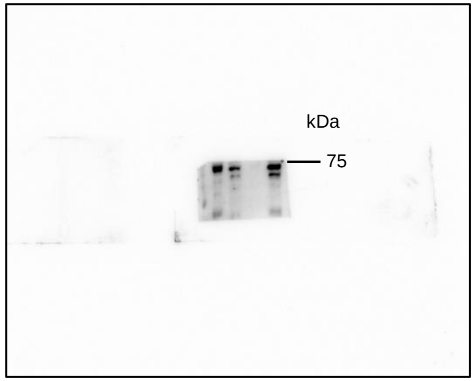

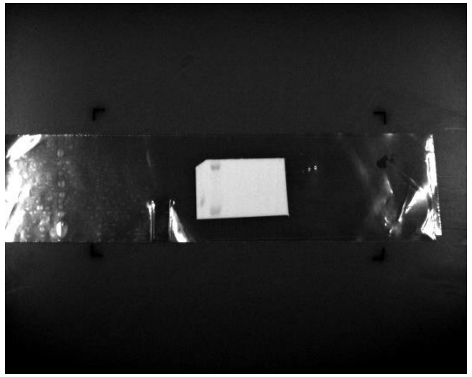

FLAG
-CDKL3

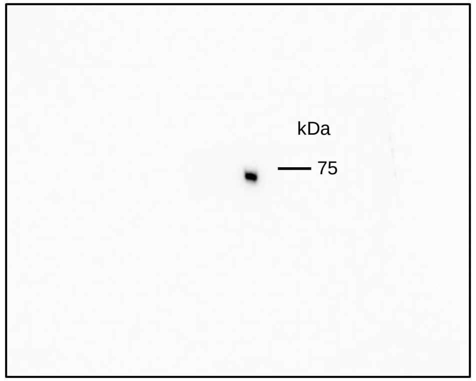

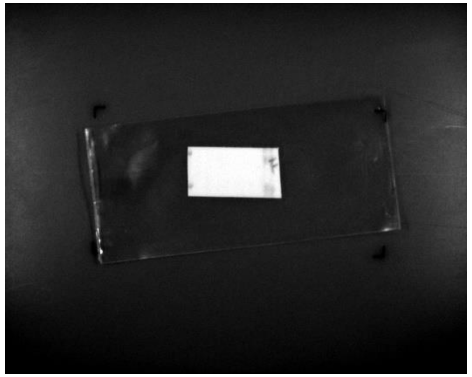

GAPDH

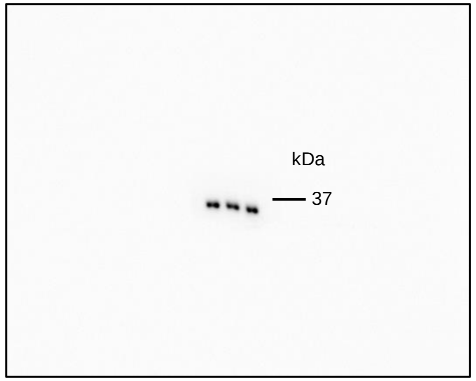

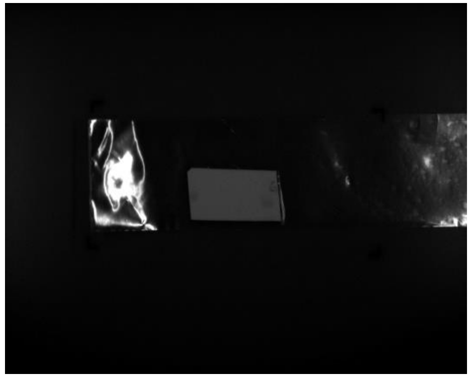

Full unedited blot for Supplemental Figure 1Q

pRb

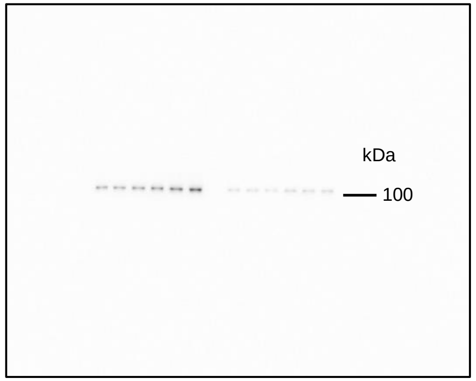

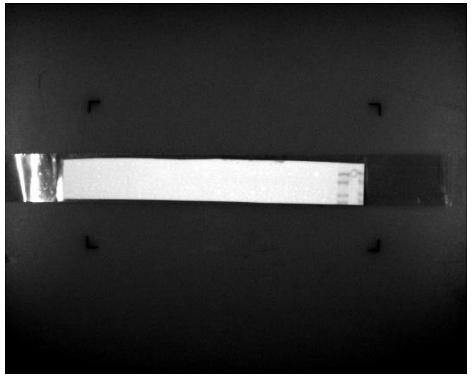

Rb

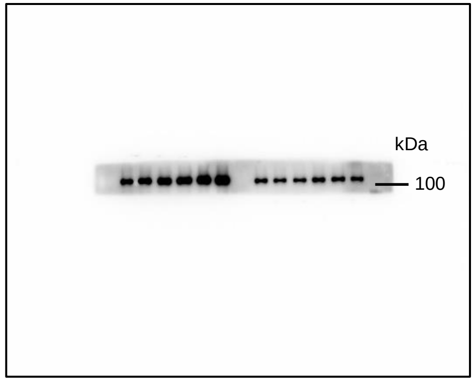

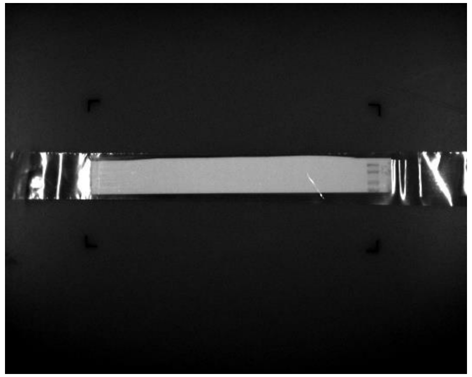

CDKL3

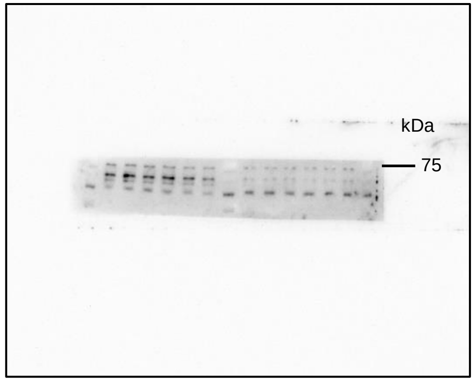

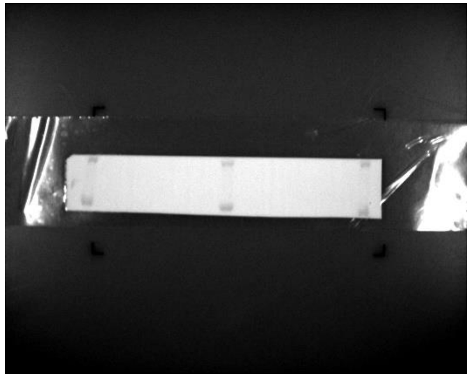

Full unedited blot for Supplemental Figure 1Q

cyclinA2

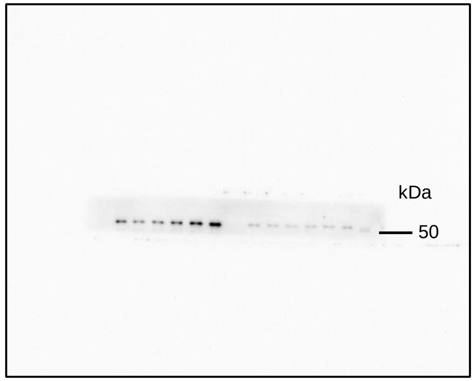

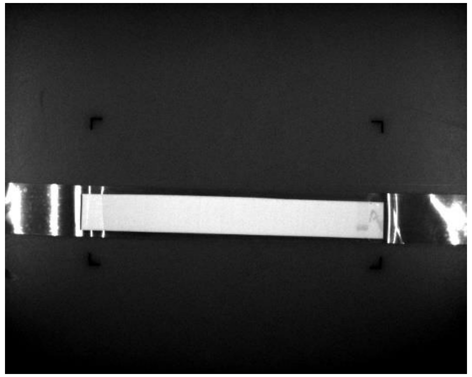

cyclinD1

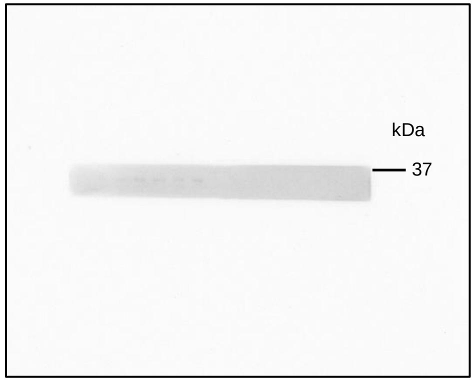

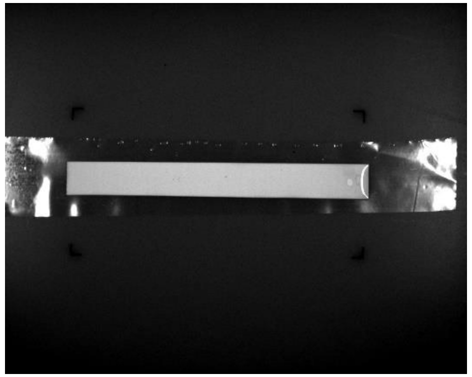

cyclinE1

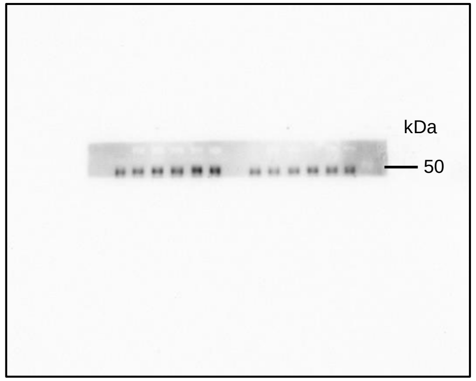

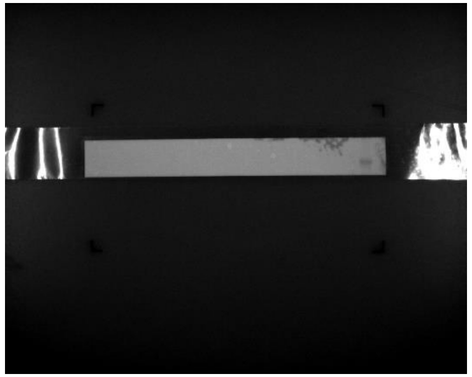

GAPDH

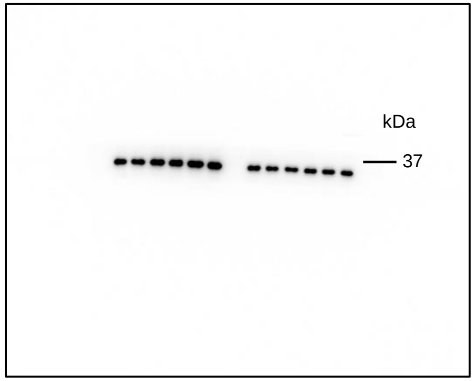

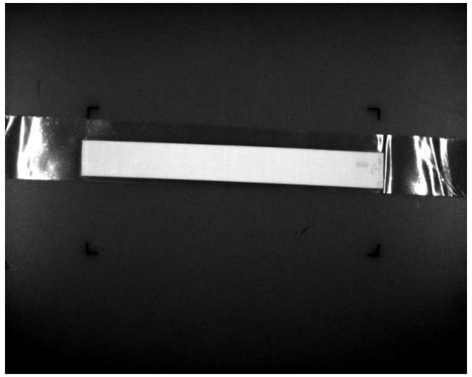

Full unedited blot for Supplemental Figure 1R

pRb

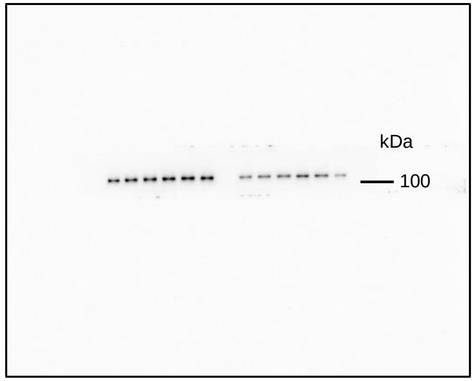

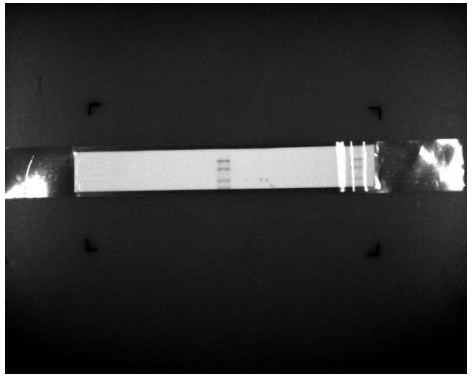

Rb

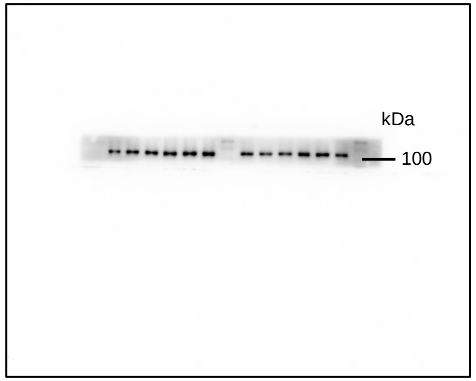

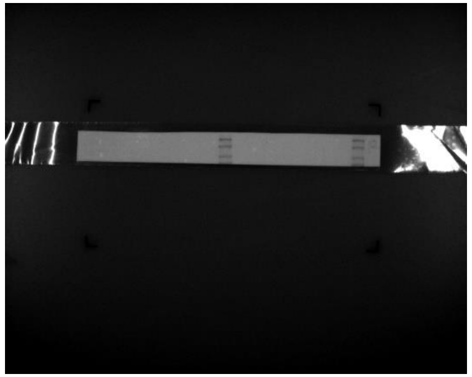

CDKL3

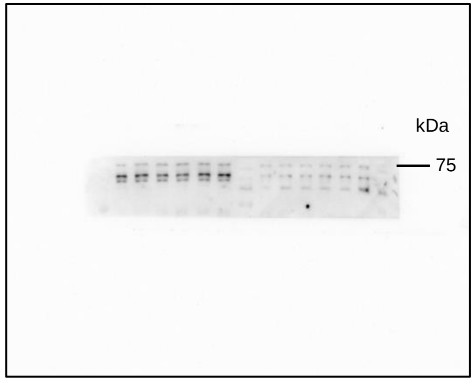

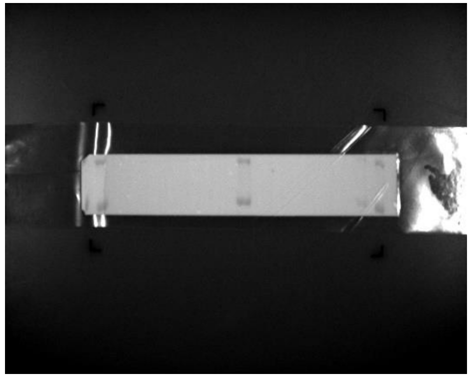

Full unedited blot for Supplemental Figure 1R

cyclinA2

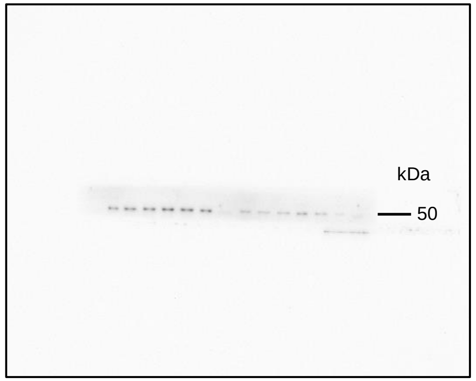

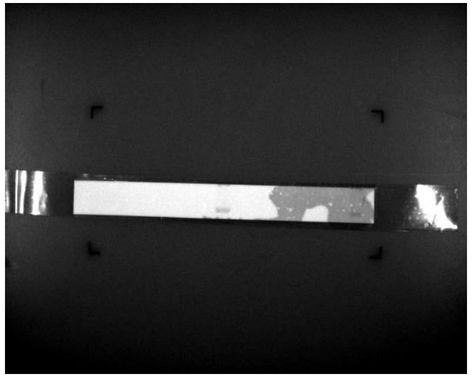

cyclinD1

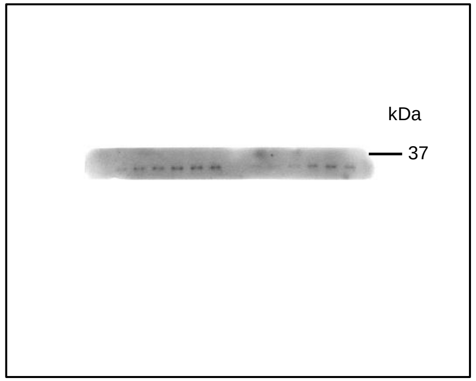

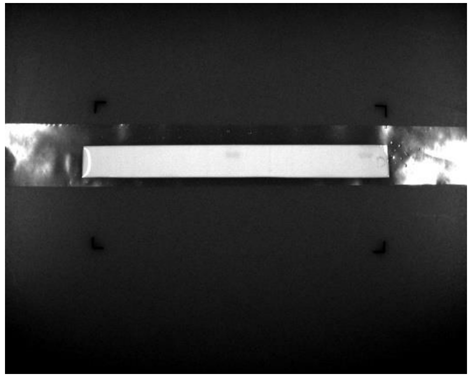

cyclinE1

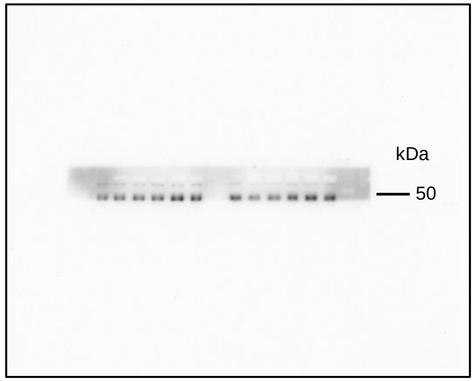

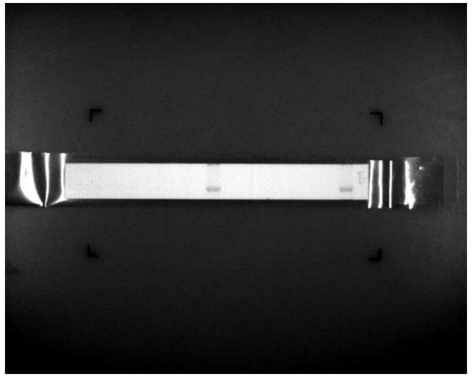

GAPDH

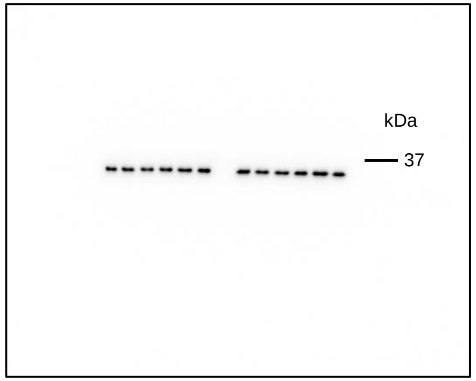

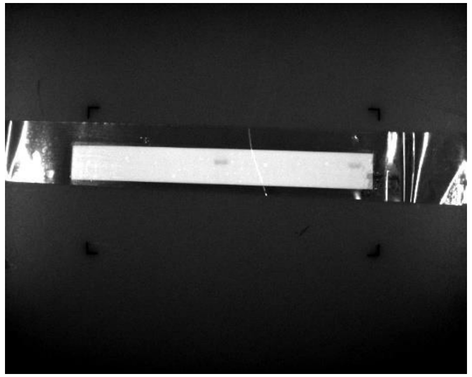

Full unedited blot for Supplemental Figure 1W

CDKL3

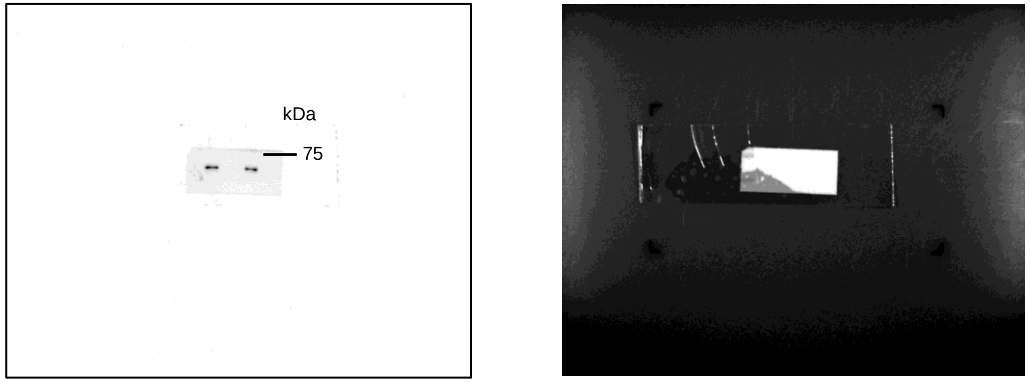

HES1

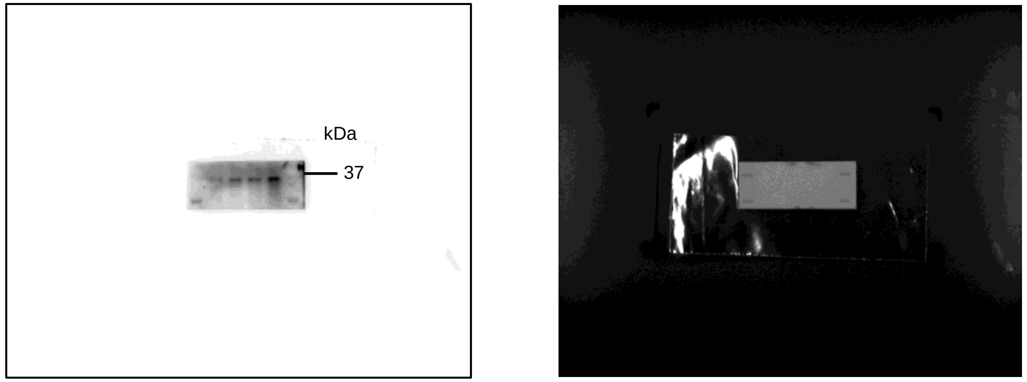

p27

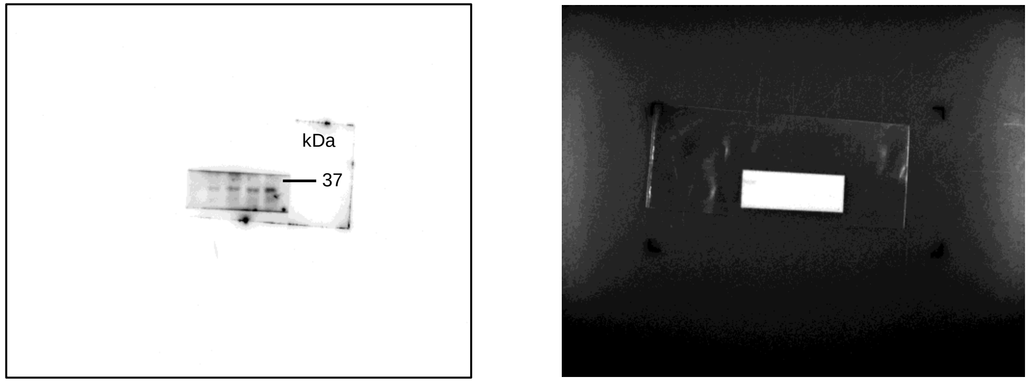

GAPDH

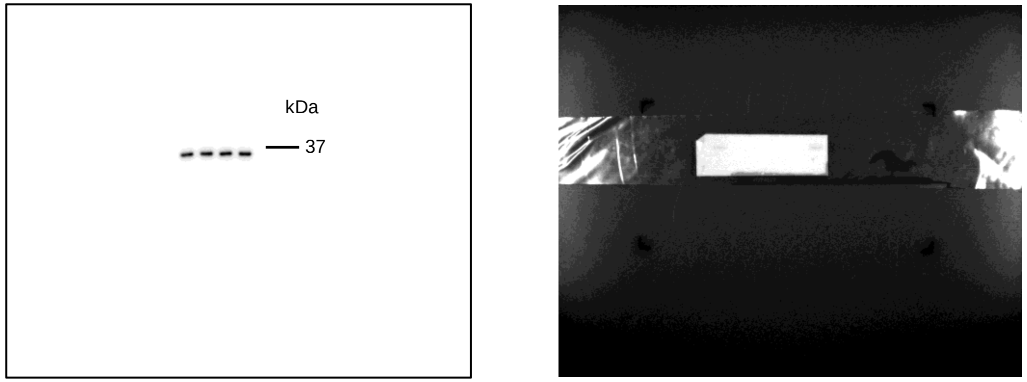

Full unedited blot for Supplemental Figure 1X

CDKL3

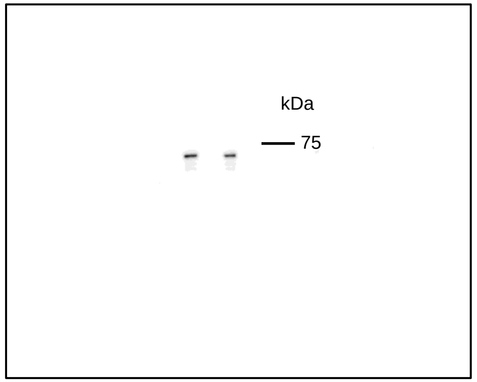

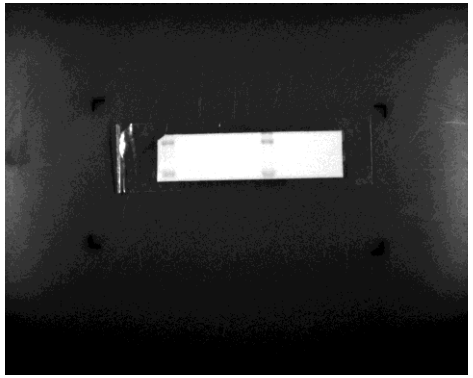

HES1

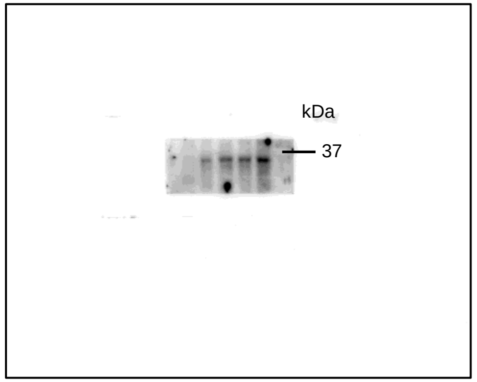

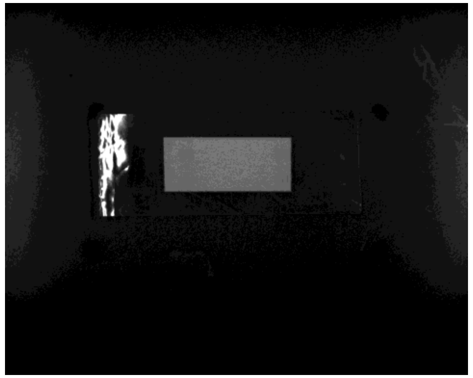

p27

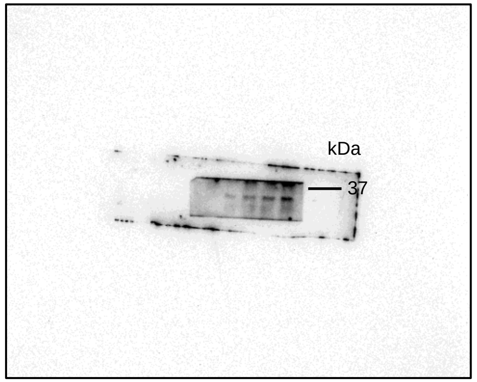

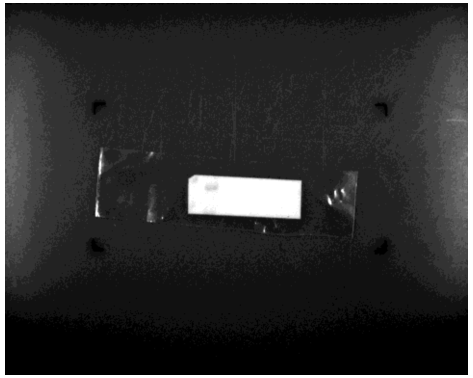

GAPDH

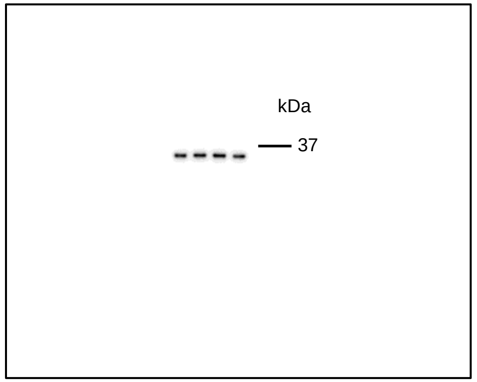

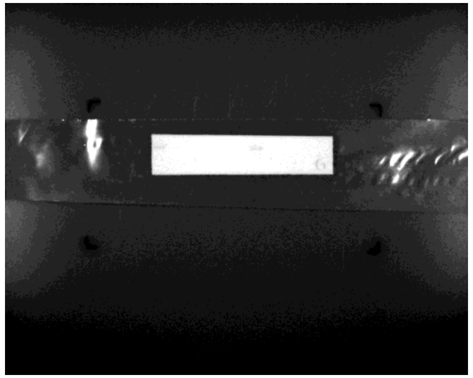

Full unedited blot for Supplemental Figure 1AC

U2OS

CDKL3

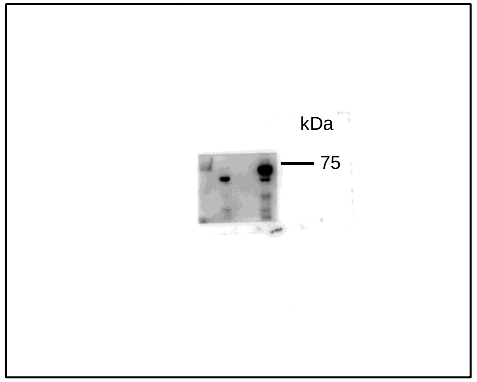

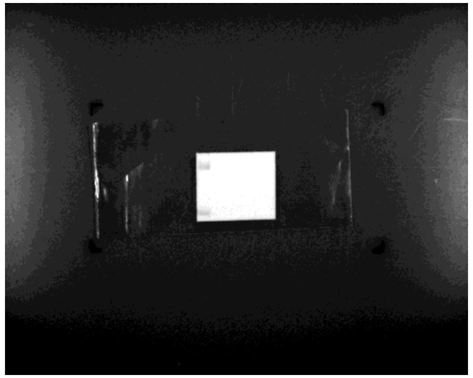

FLAG
-CDKL3

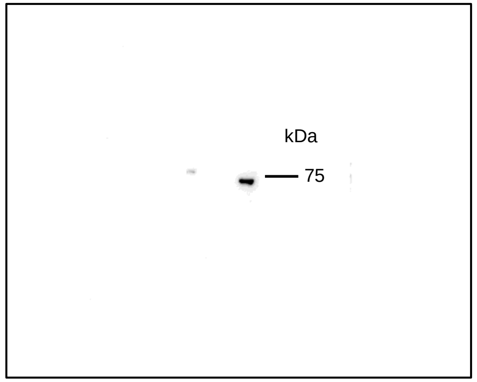

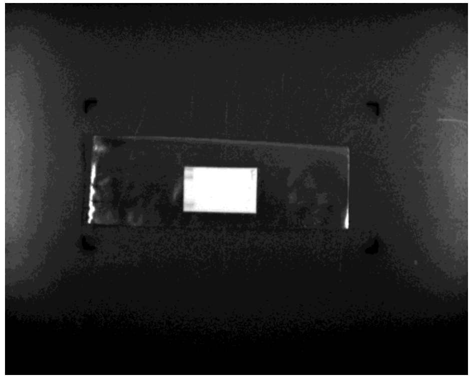

GAPDH

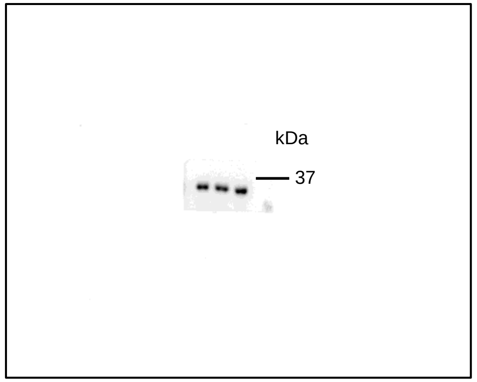

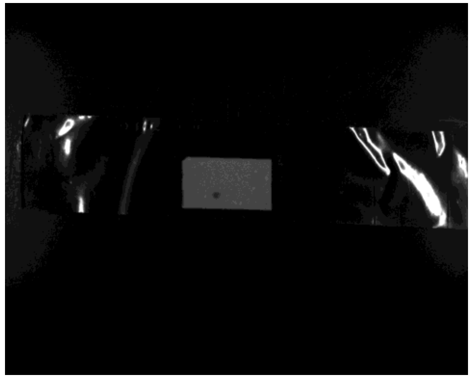

Full unedited blot for Supplemental Figure 1AC

DLD-1

CDKL3

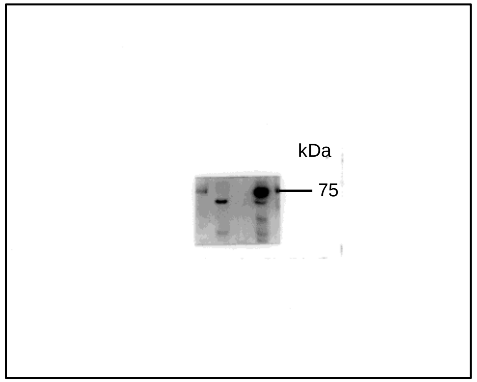

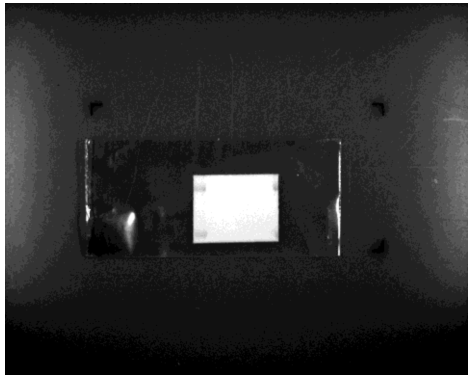

FLAG
-CDKL3

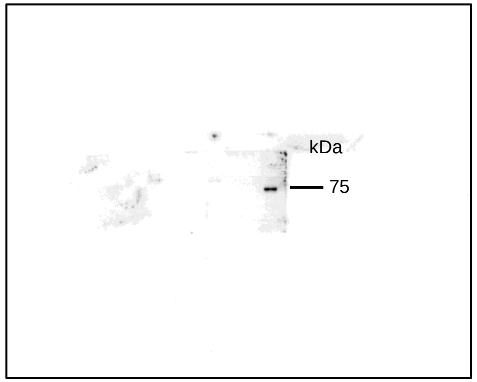

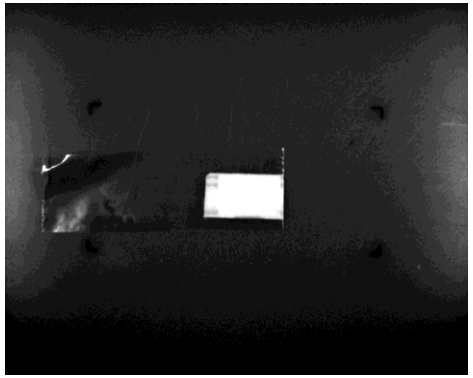

GAPDH

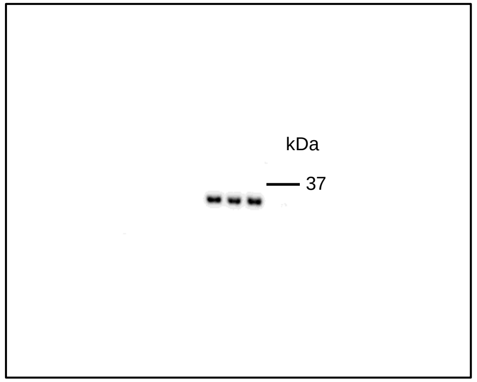

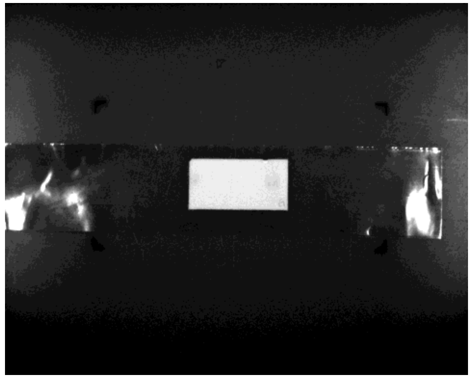

Full unedited blot for Supplemental Figure 2A

FLAG
-CDKL3

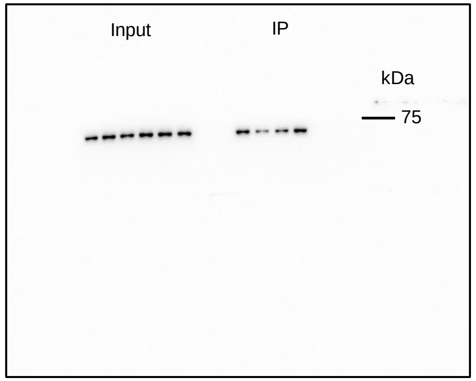

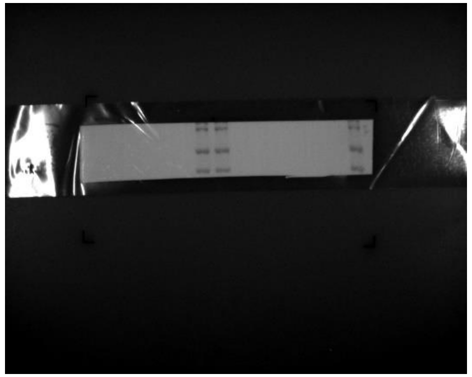

MYC
-cyclin

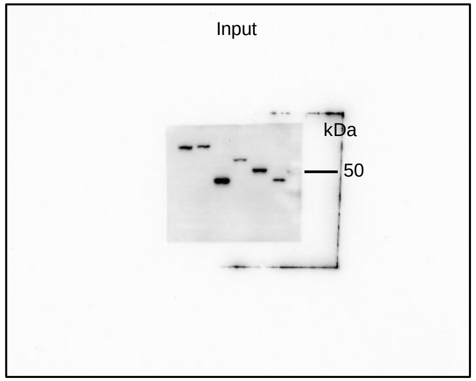

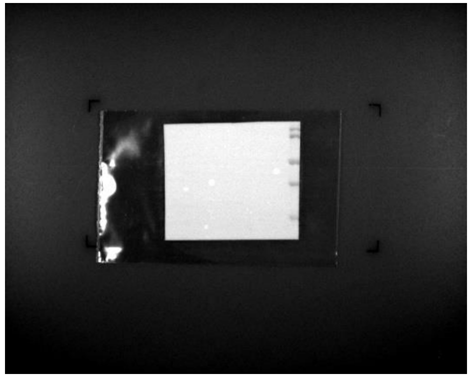

MYC
-cyclin

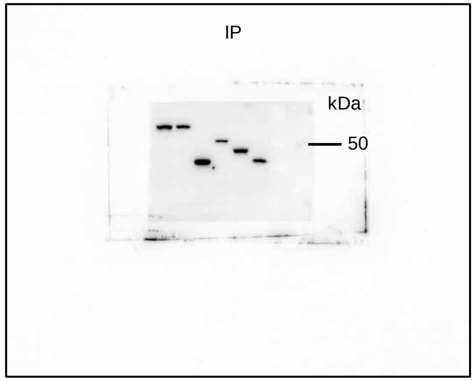

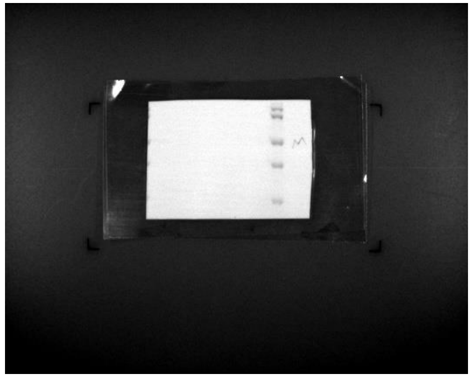

Full unedited blot for Supplemental Figure 2B

cyclinA2

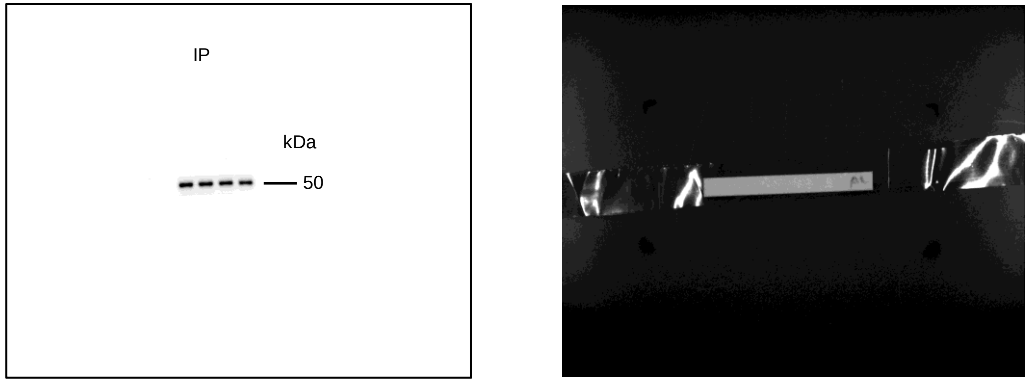

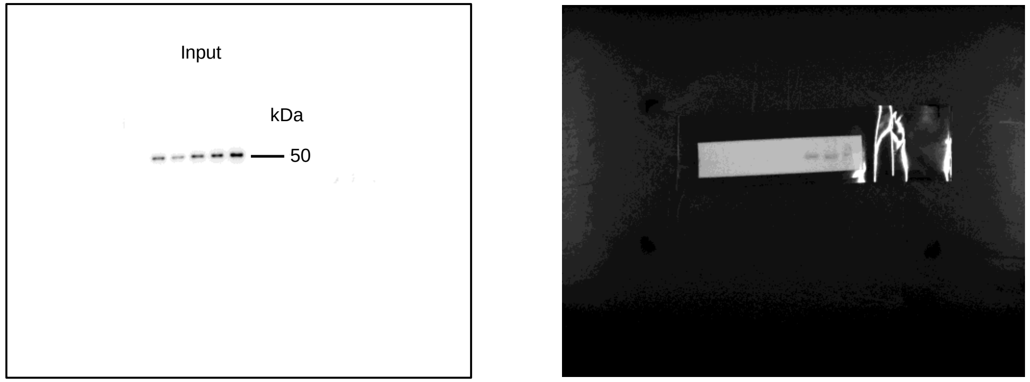

cyclinB1

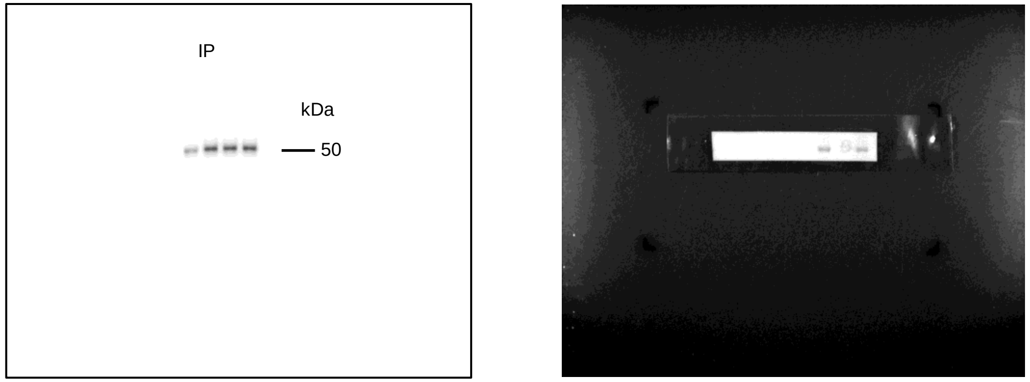

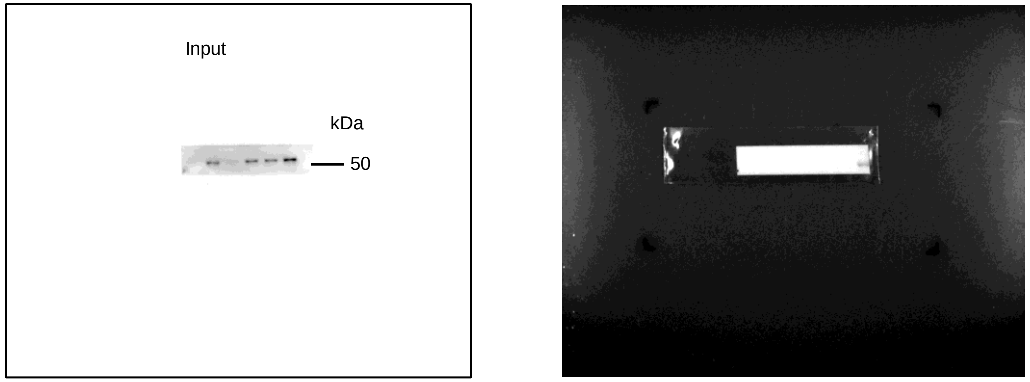

Full unedited blot for Supplemental Figure 2B

cyclinD1

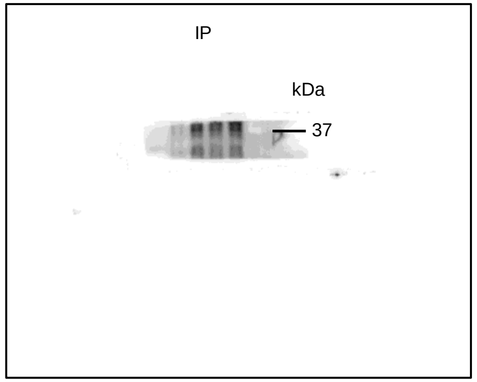

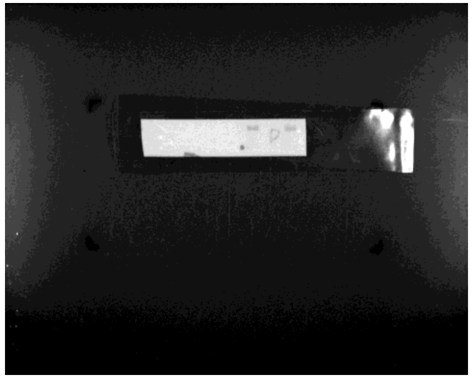

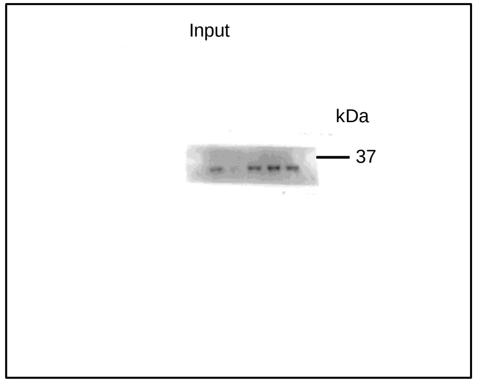

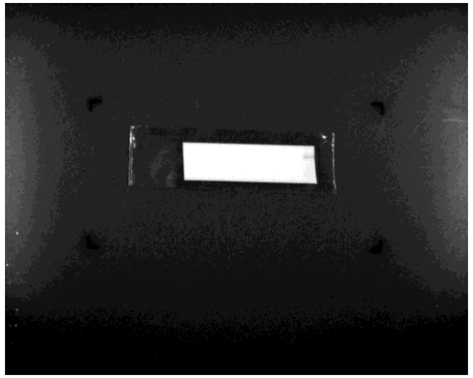

cyclinE1

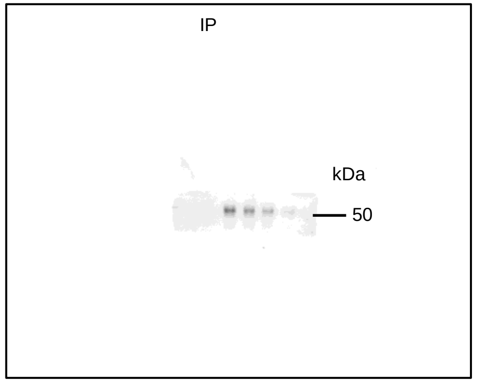

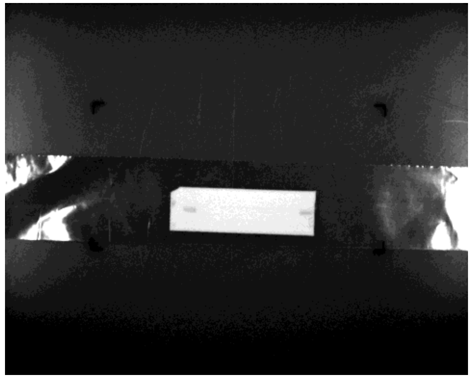

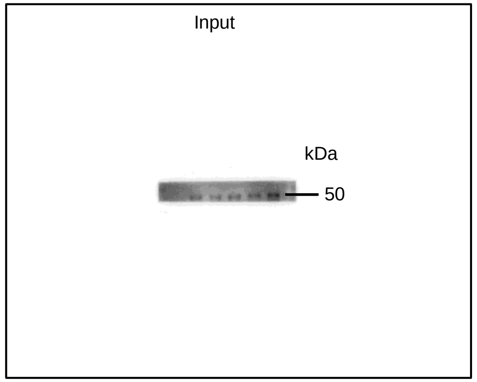

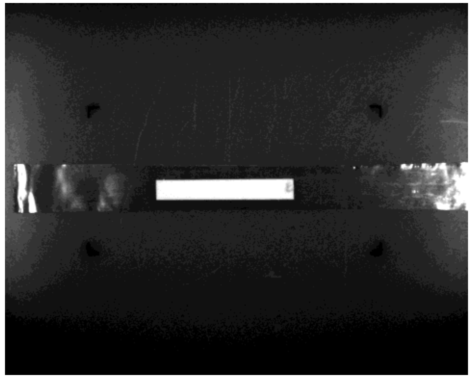

Full unedited blot for Supplemental Figure 2B

CDKL3

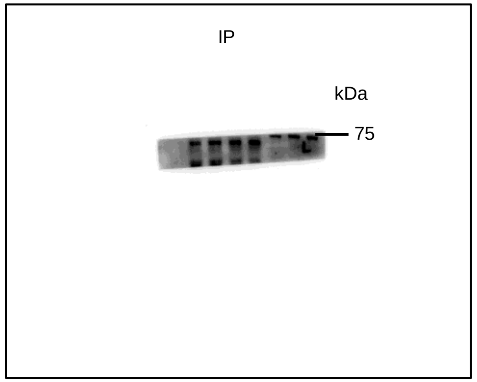

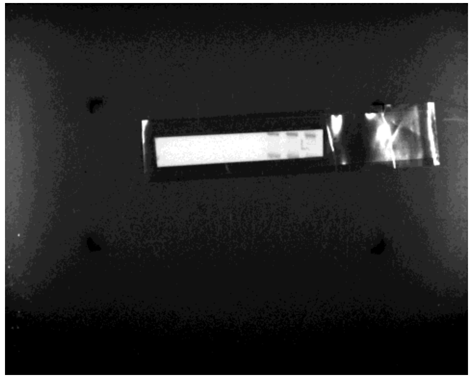

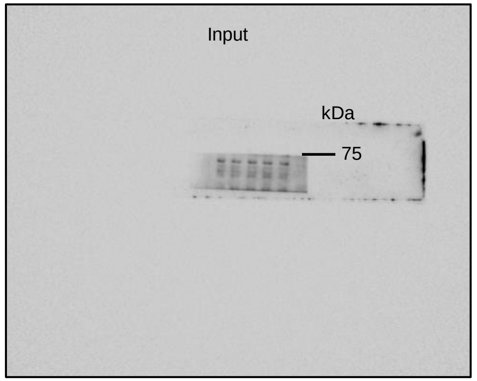

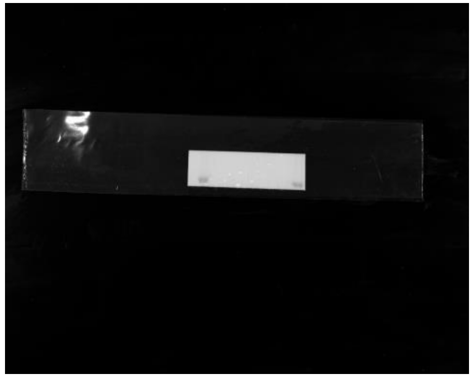

α-tubulin

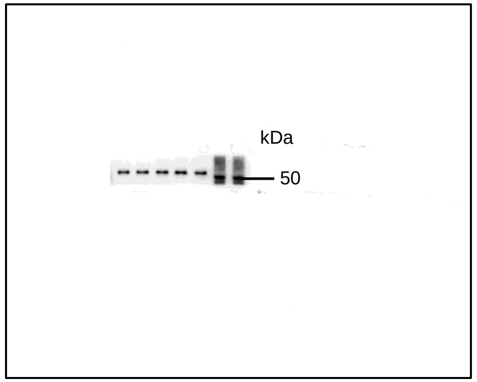

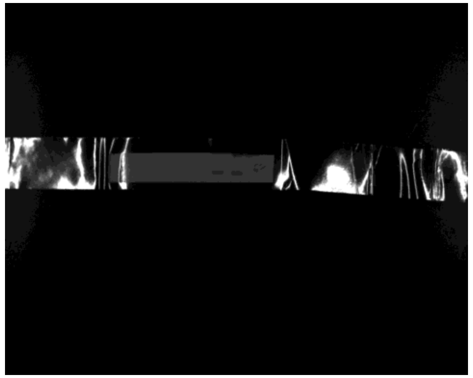

Full unedited blot for Supplemental Figure 2C

MYC-p16/p21/p27

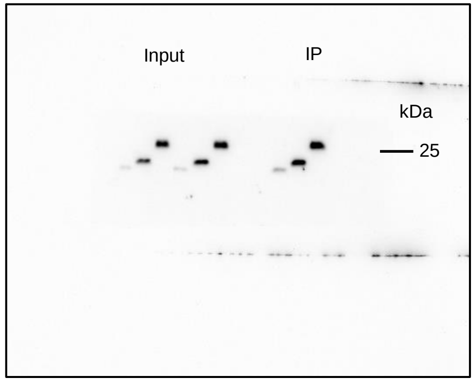

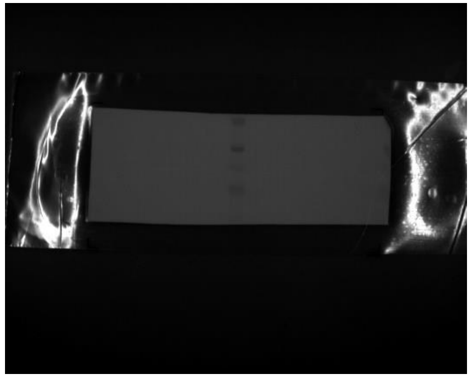

FLAG-CDKL3/CDK4

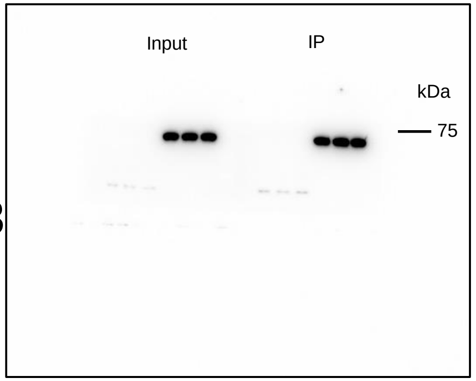

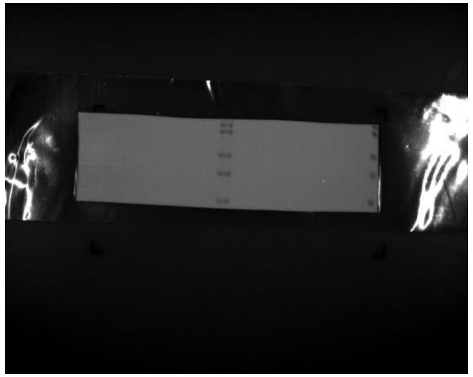

Full unedited blot for Supplemental Figure 2D

MYC-cyclin

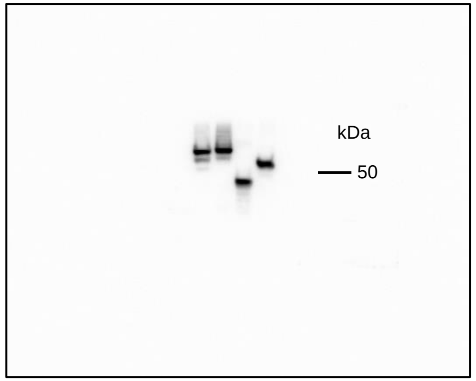

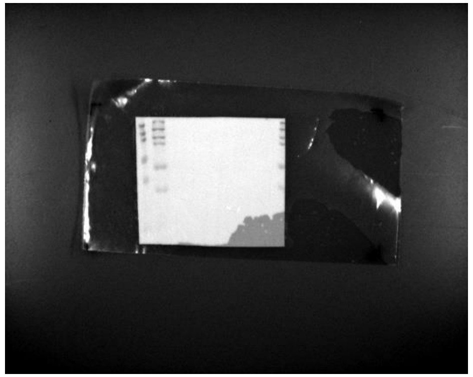

Full unedited blot for Supplemental Figure 2E

pRb

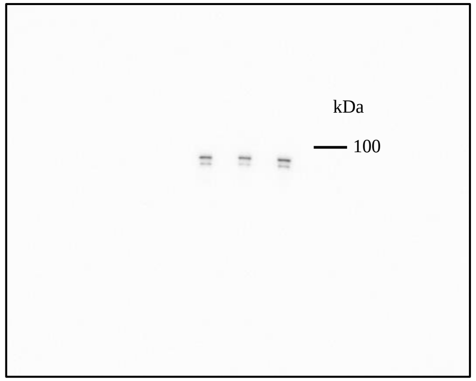

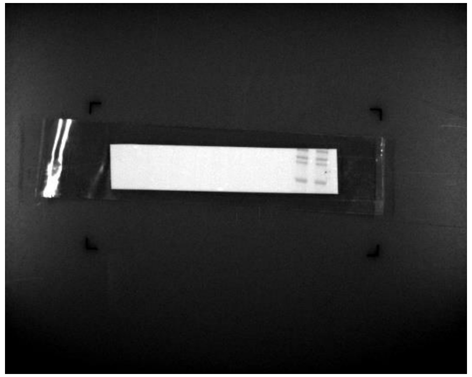

GST

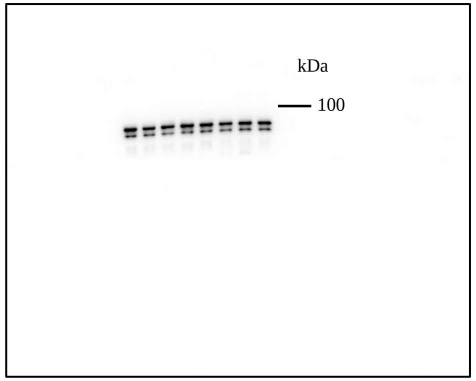

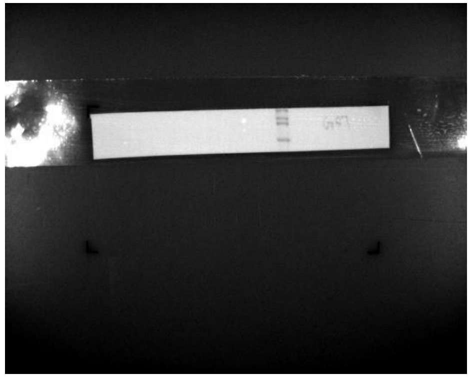

Full unedited blot for Supplemental Figure 2G

pRb

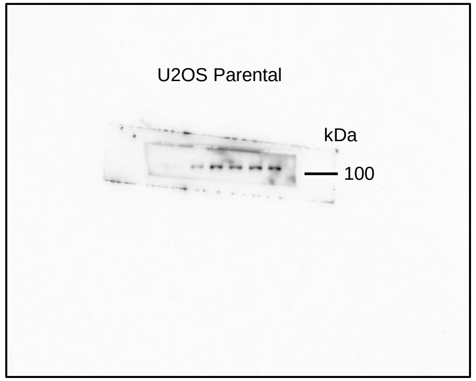

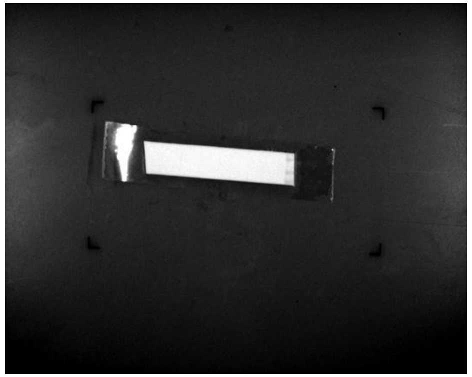

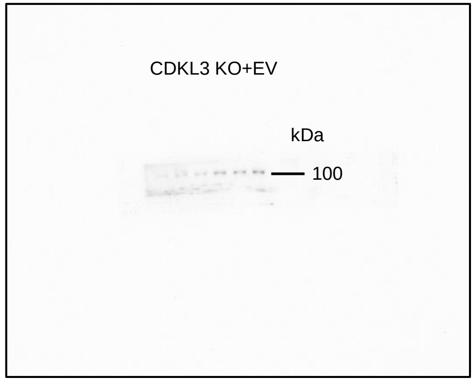

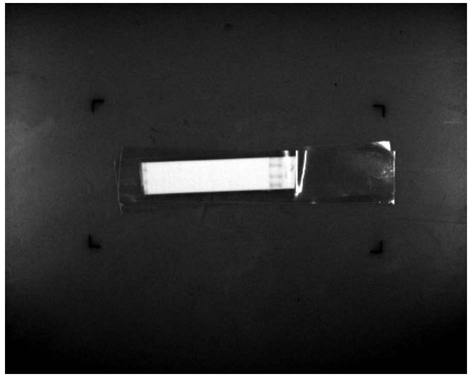

Full unedited blot for Supplemental Figure 2G

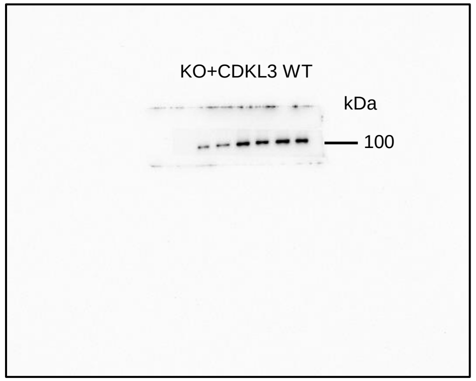

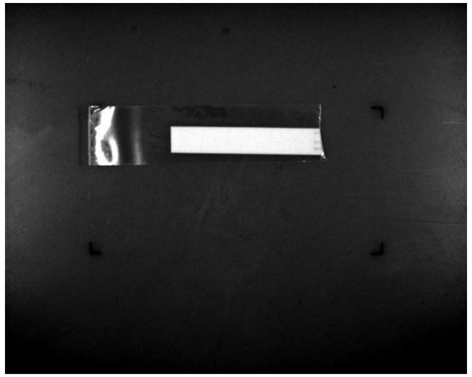

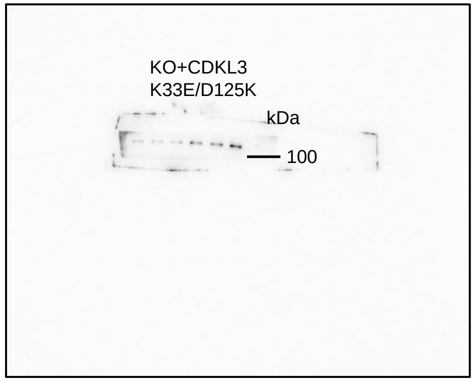

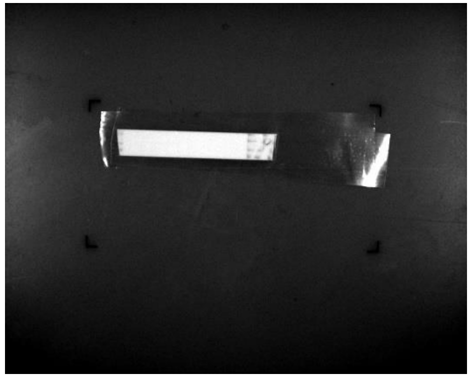

pRb

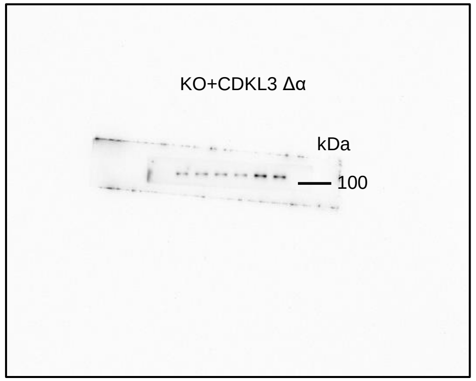

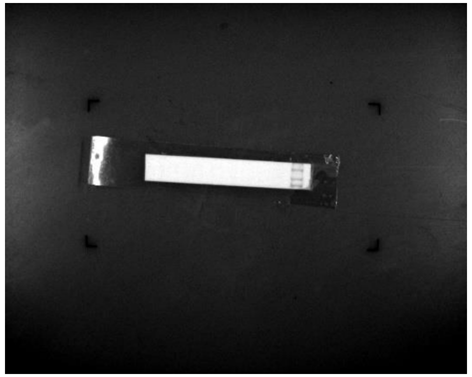

Full unedited blot for Supplemental Figure 2G

Rb

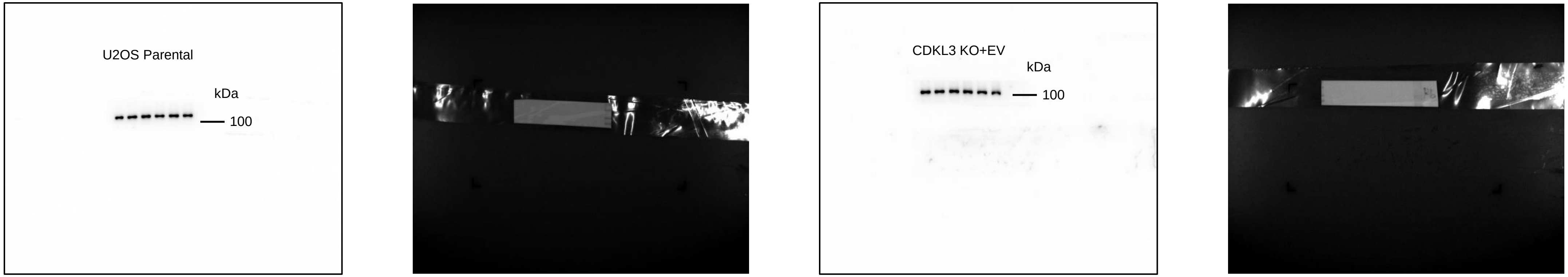

Full unedited blot for Supplemental Figure 2G

Rb

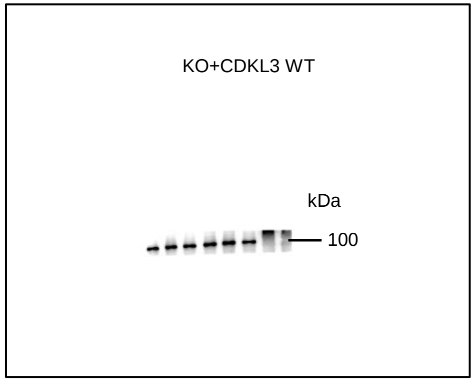

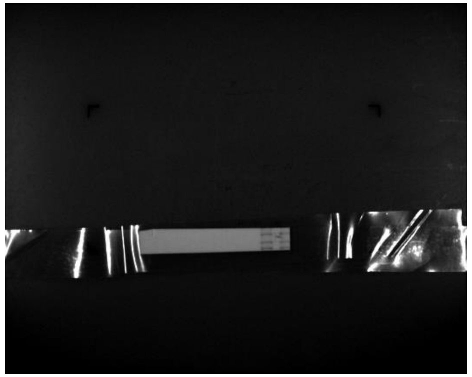

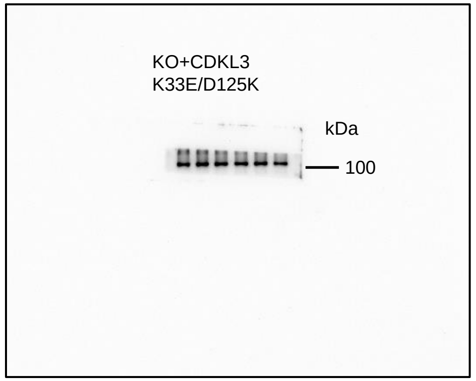

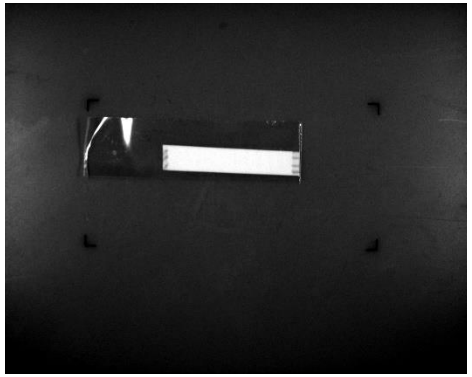

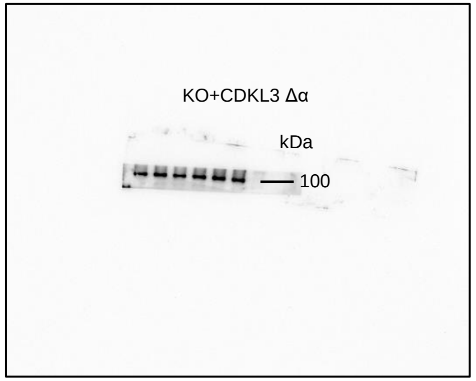

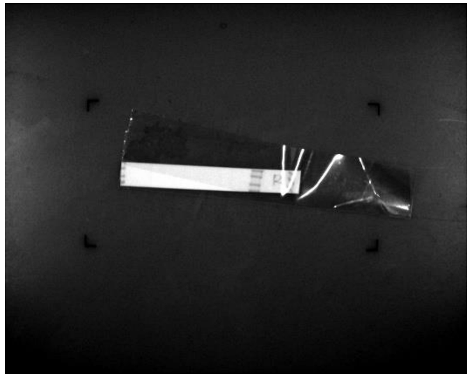

Full unedited blot for Supplemental Figure 2G

cyclinA2

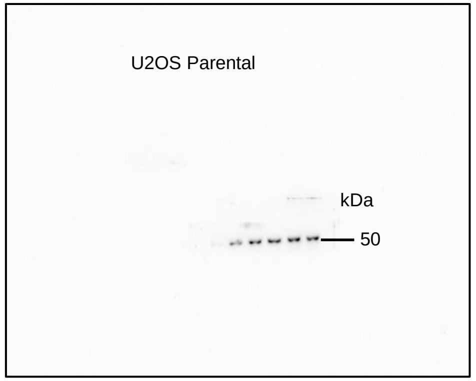

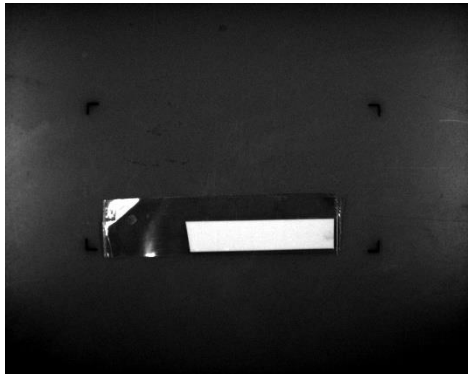

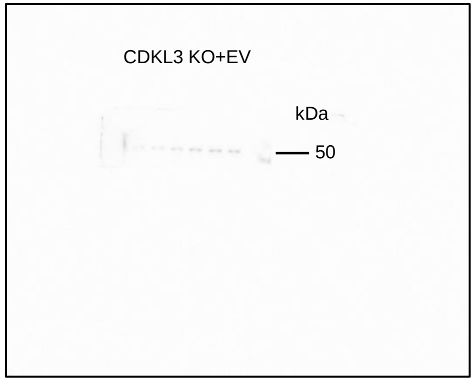

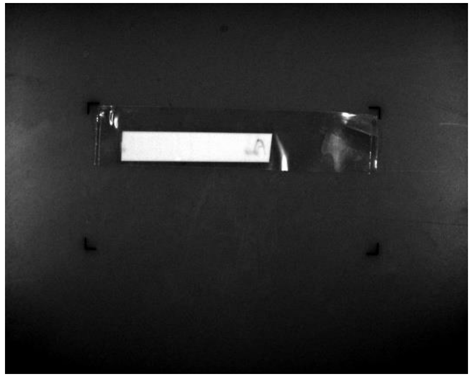

Full unedited blot for Supplemental Figure 2G

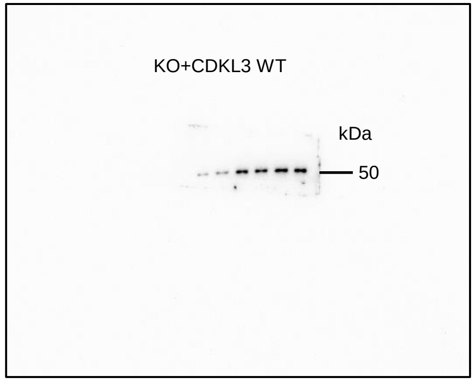

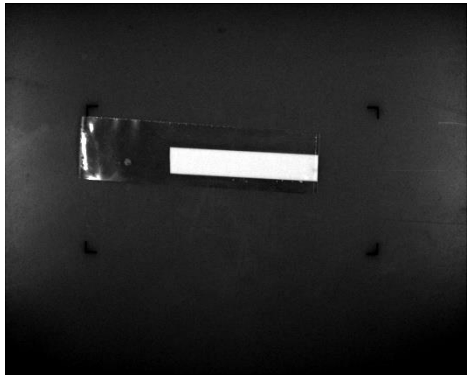

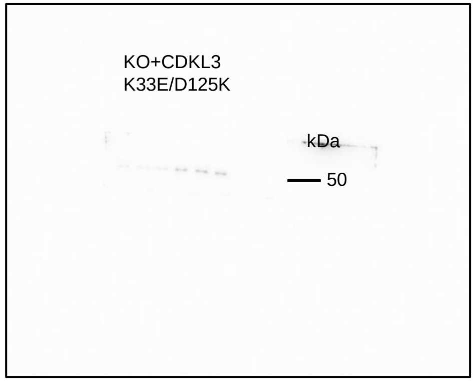

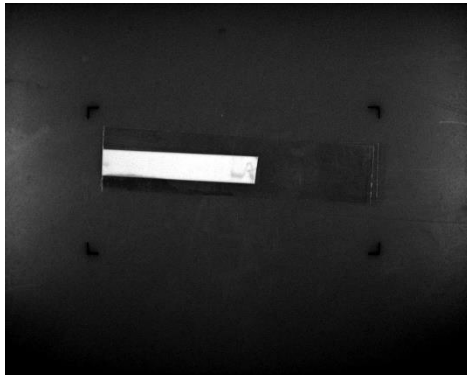

cyclinA2

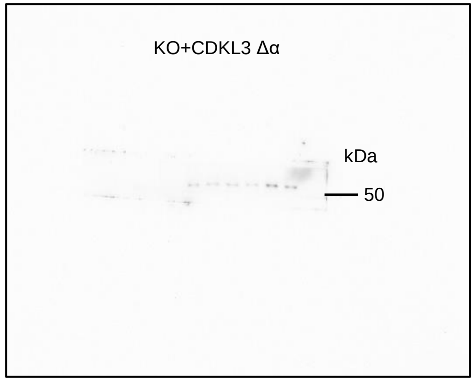

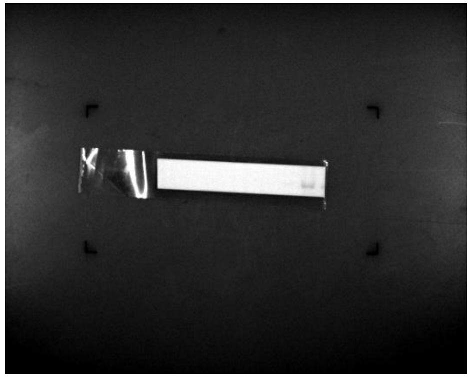

Full unedited blot for Supplemental Figure 2G

FLAG-CDKL3

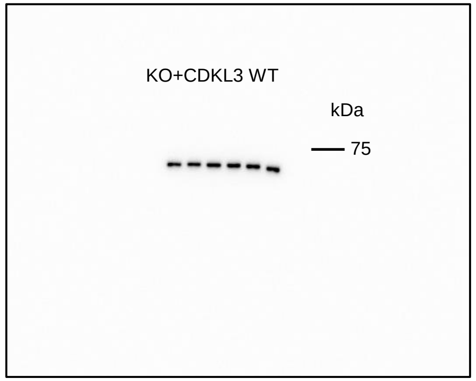

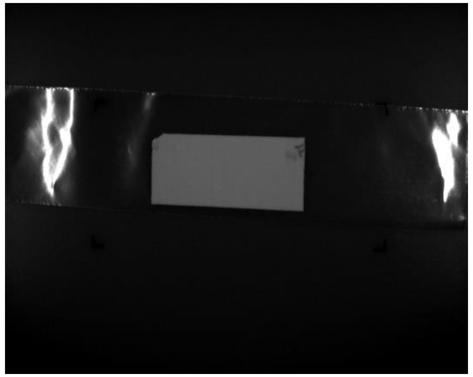

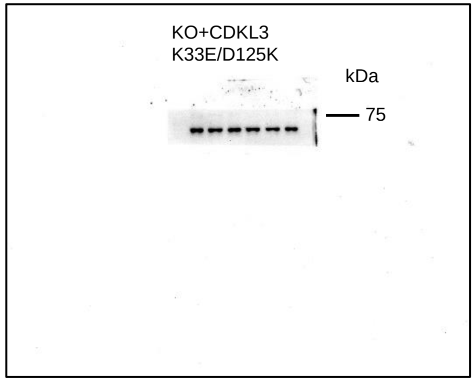

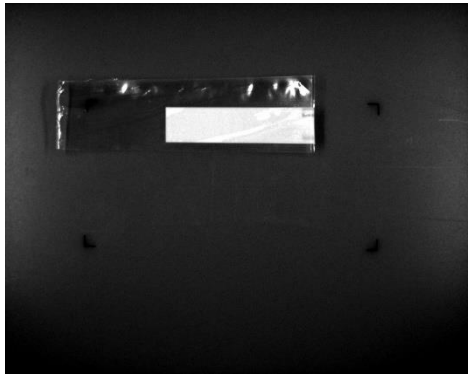

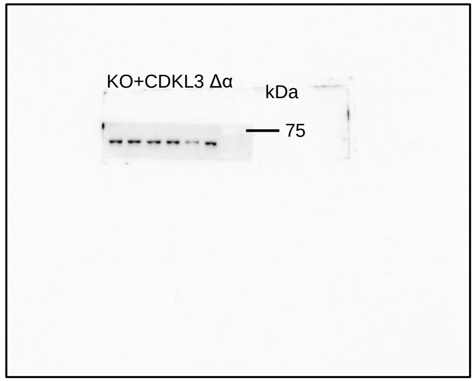

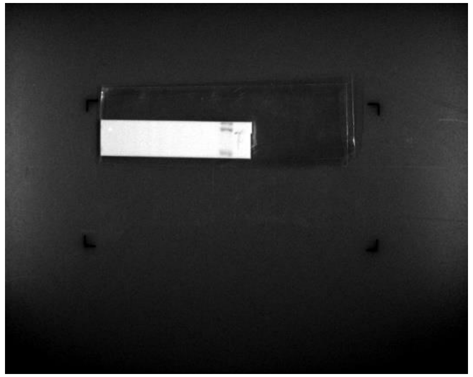

Full unedited blot for Supplemental Figure 2G

GAPDH

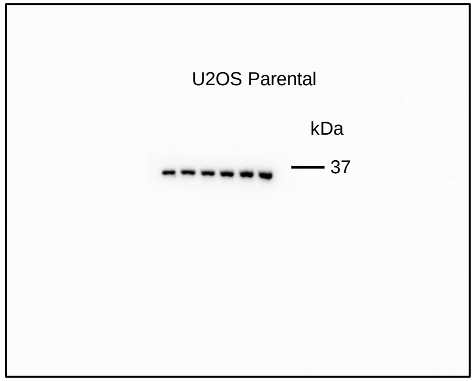

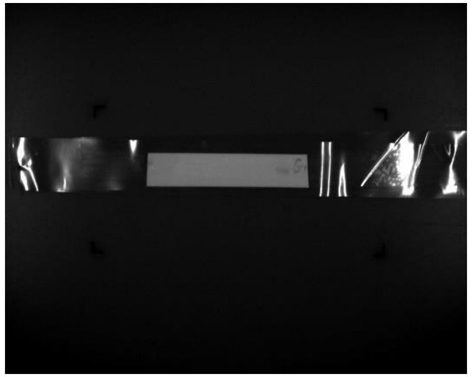

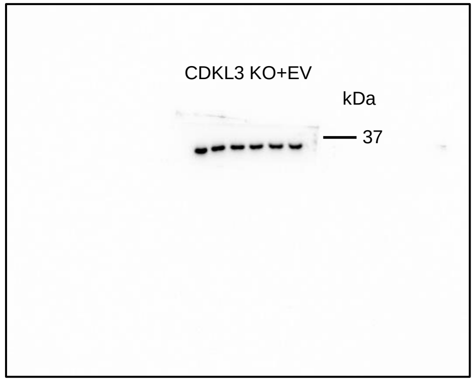

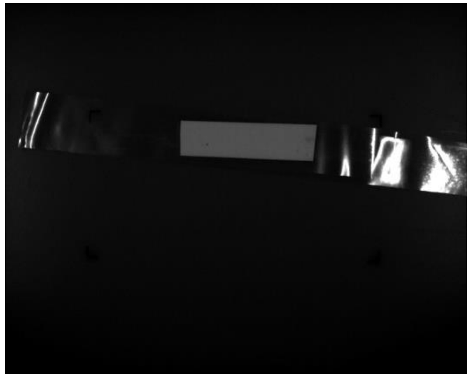

Full unedited blot for Supplemental Figure 2G

GAPDH

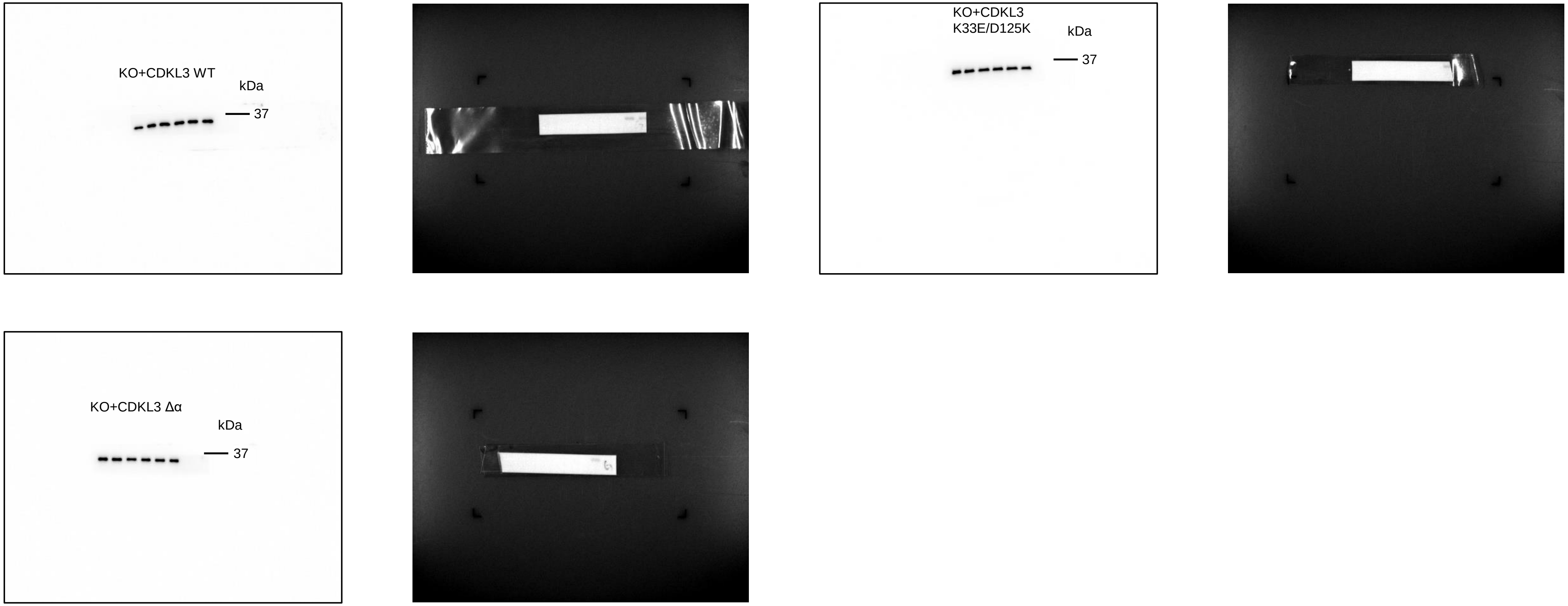

Full unedited blot for Supplemental Figure 2I

cyclinA2

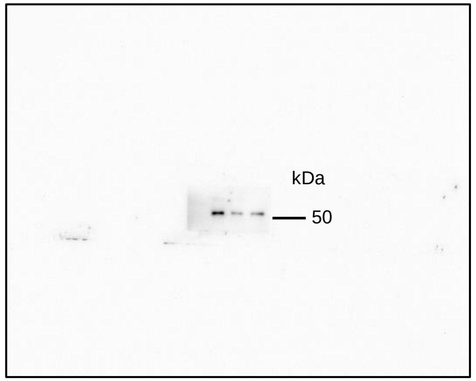

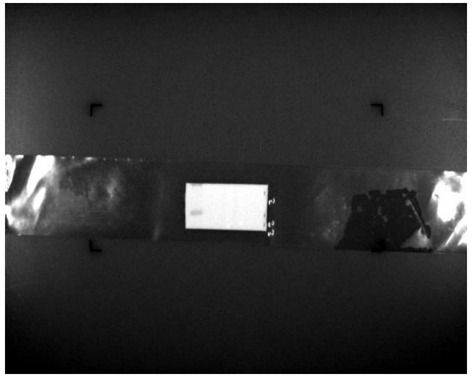

GAPDH

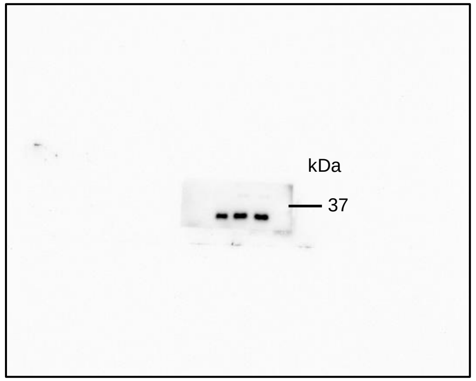

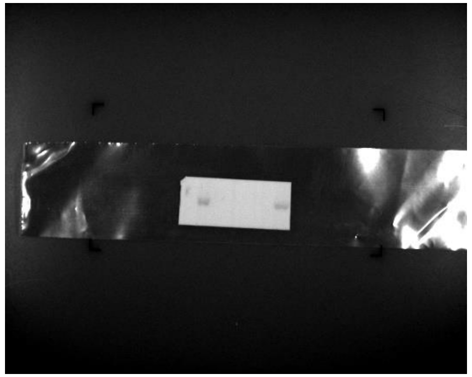

Full unedited blot for Supplemental Figure 2K

cyclinE1

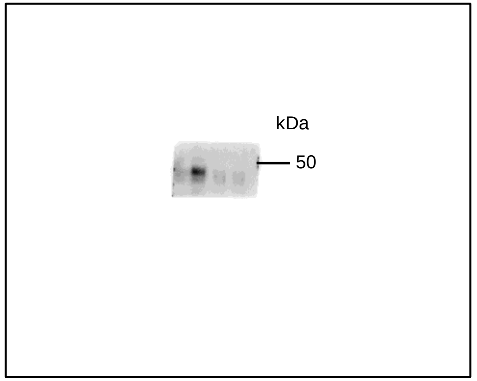

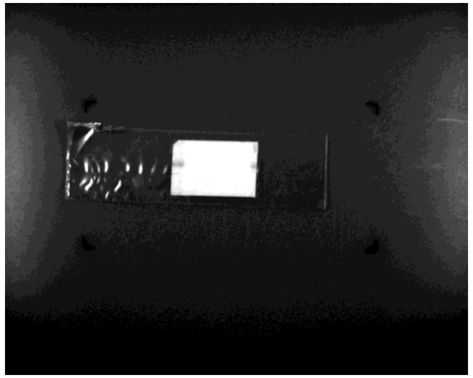

GAPDH

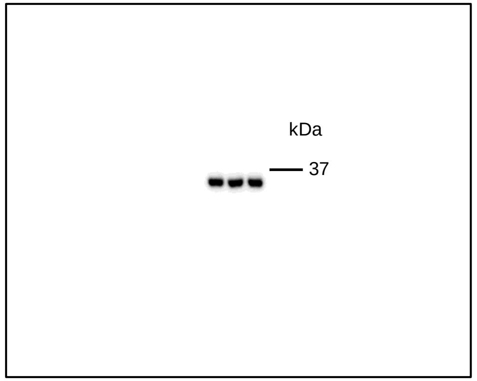

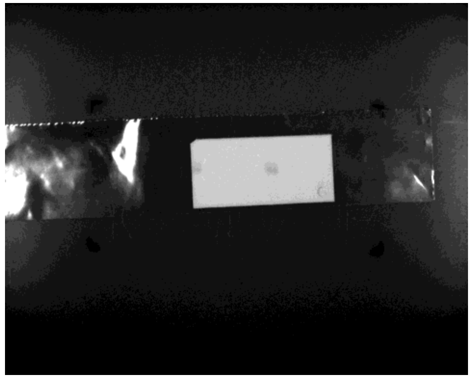

Full unedited blot for Supplemental Figure 2L

GST-FLAG-CDK2(K)

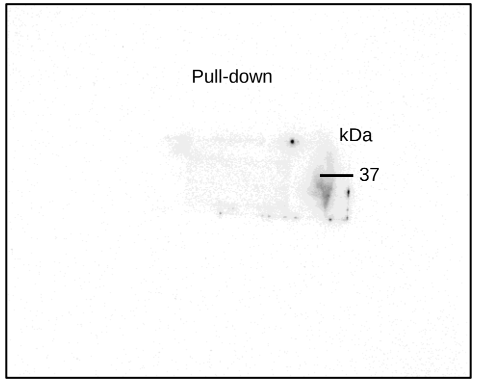

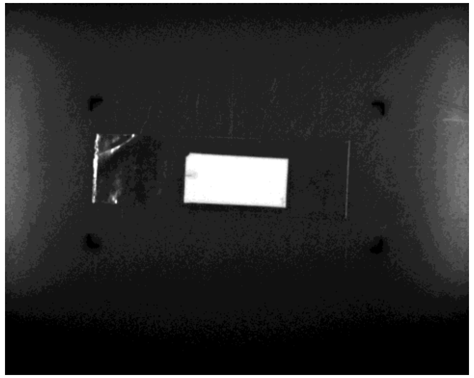

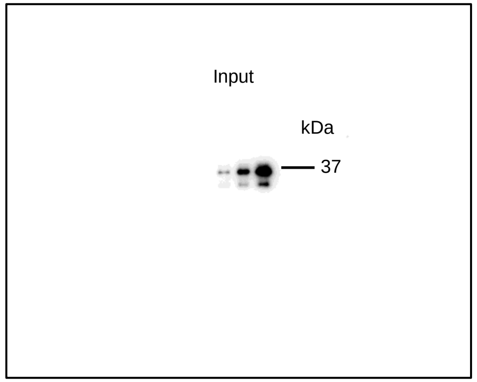

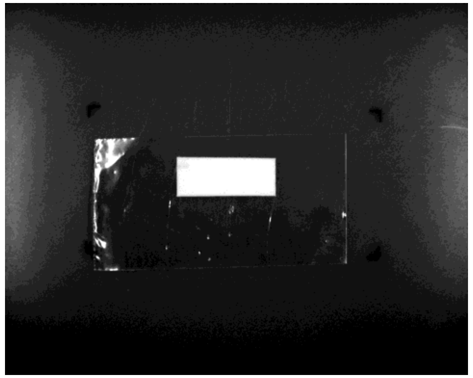

GST-MYC-CDK3(K)

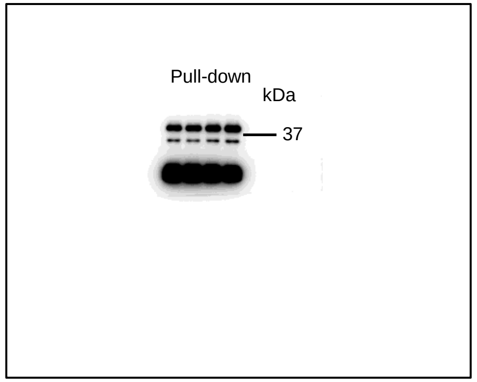

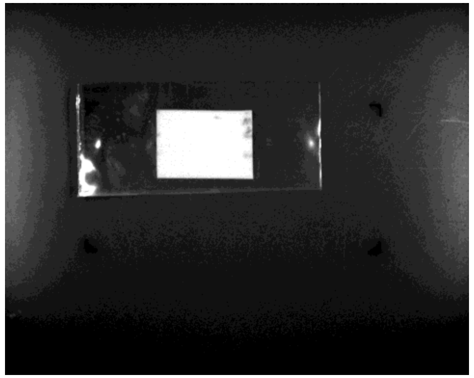

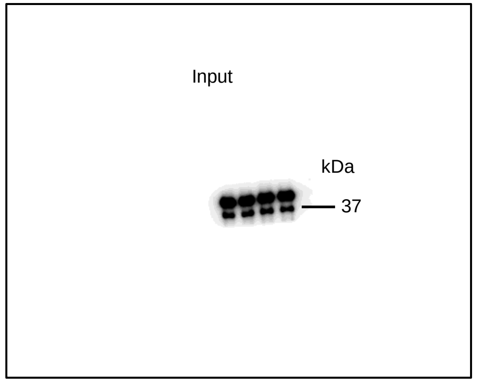

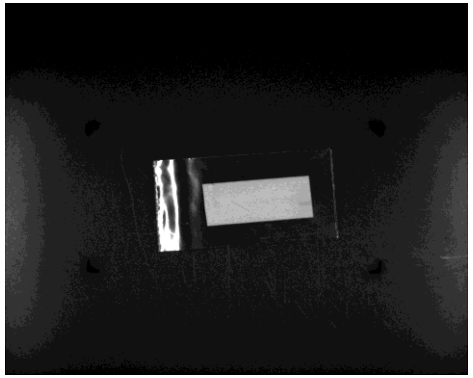

Full unedited blot for Supplemental Figure 2L

His-HA-cyclinA2

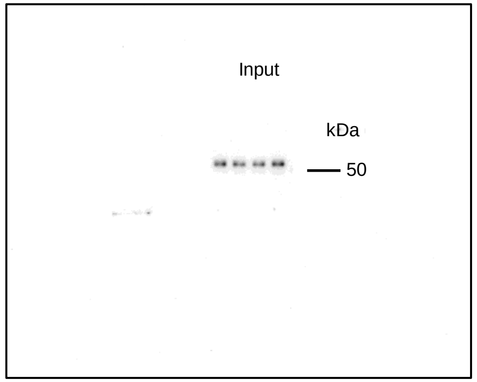

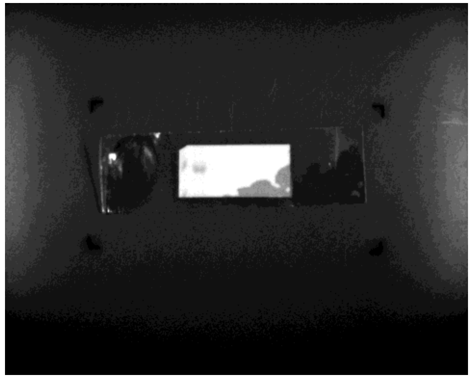

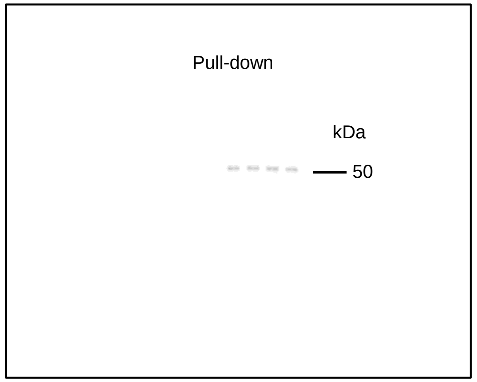

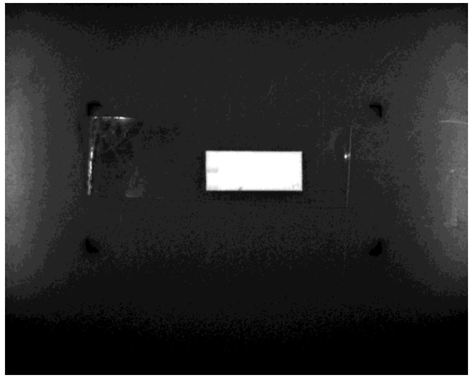

Full unedited blot for Supplemental Figure 2M

GST-FLAG-CDK2(K)

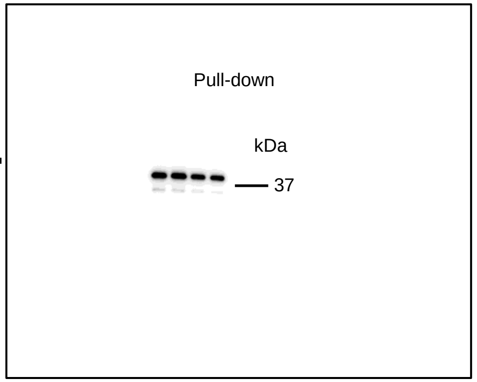

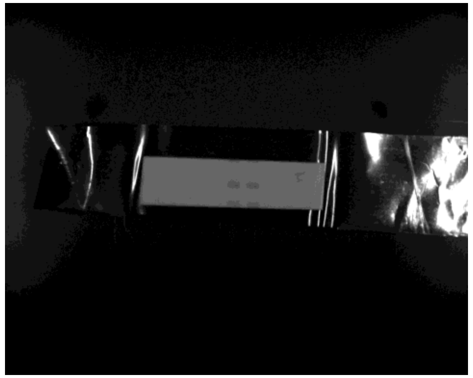

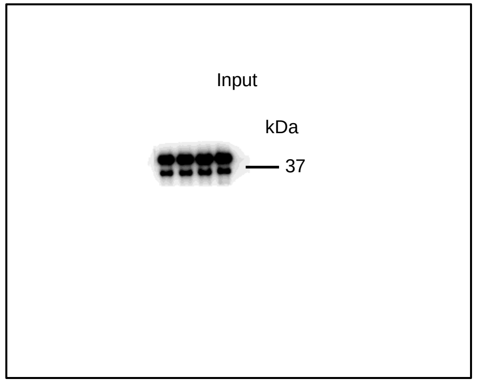

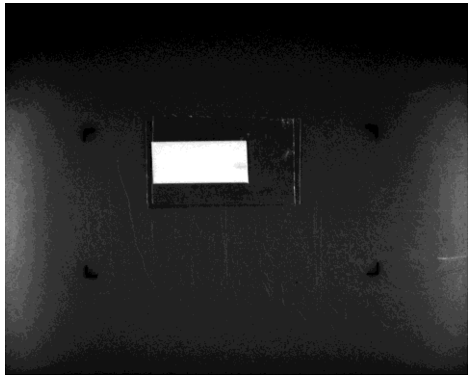

GST-MYC-CDKL3(K)

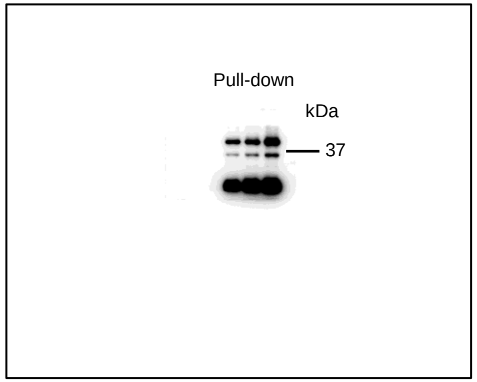

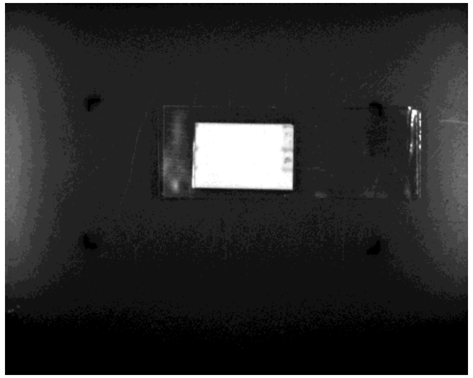

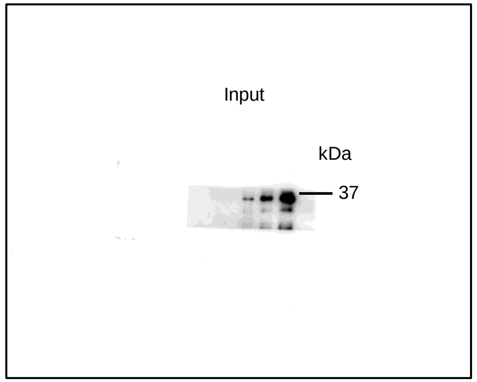

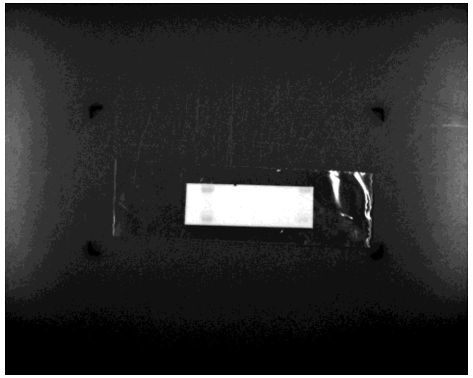

Full unedited blot for Supplemental Figure 2M

His-HA-cyclinA2

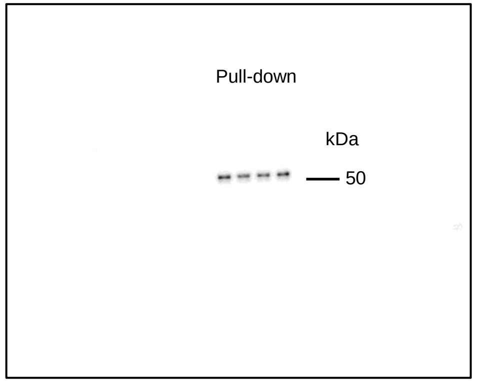

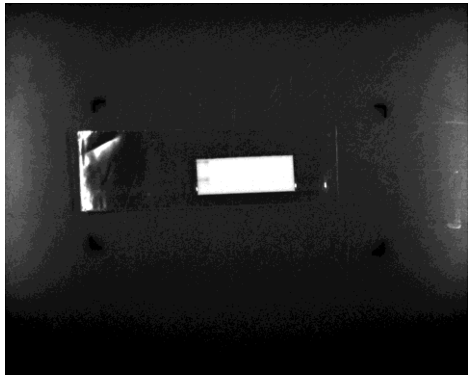

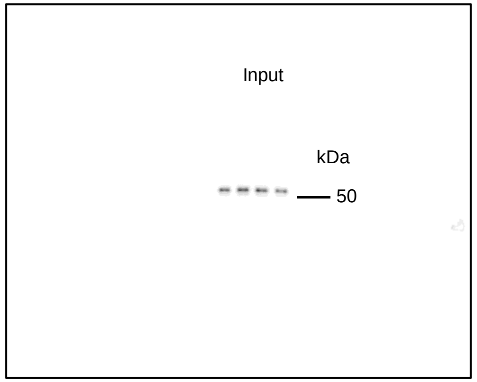

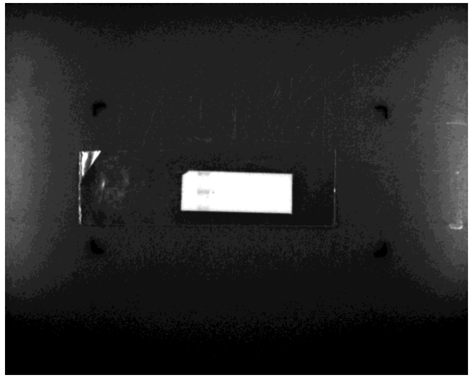

Full unedited blot for Supplemental Figure 3B

CDK4

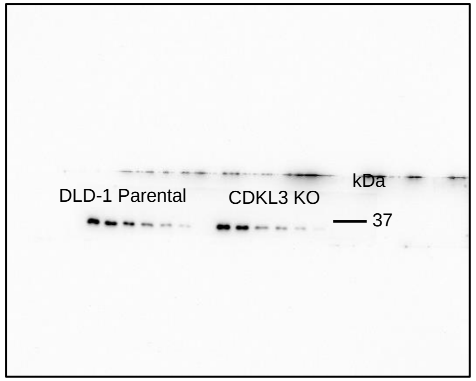

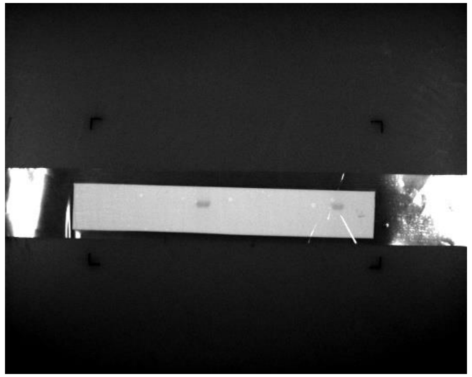

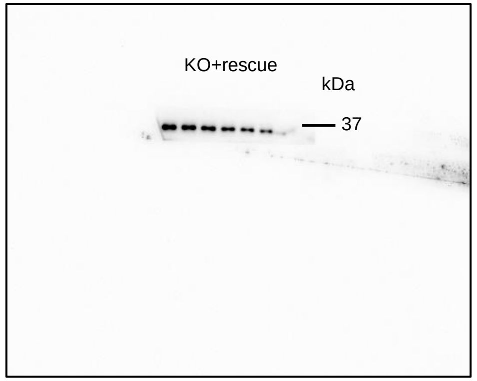

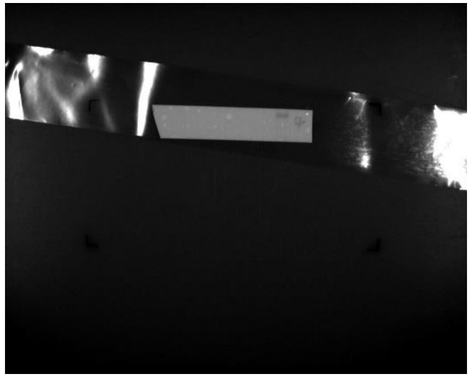

CDKL3

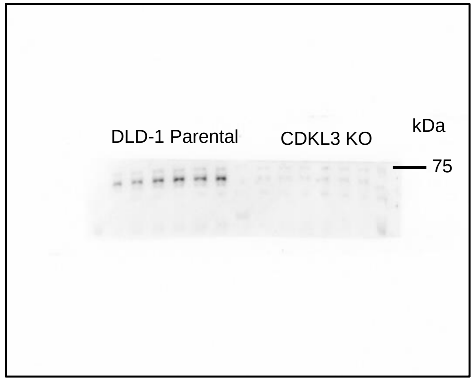

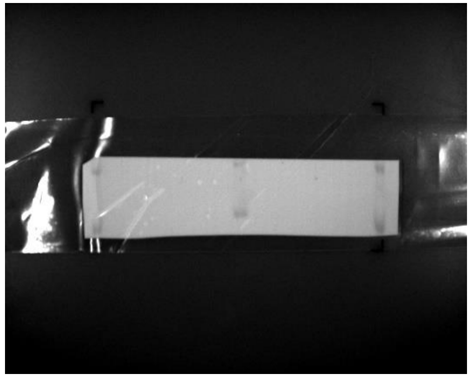

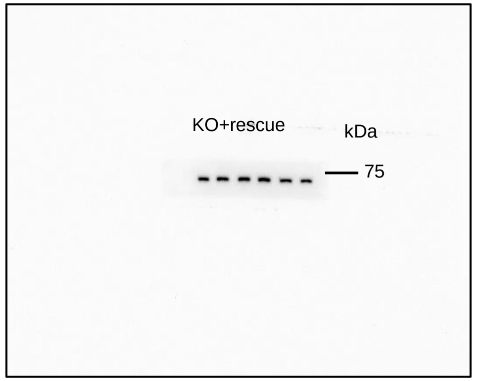

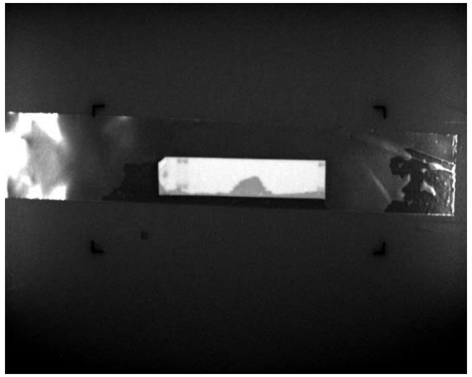

Full unedited blot for Supplemental Figure 3B

α-tubulin

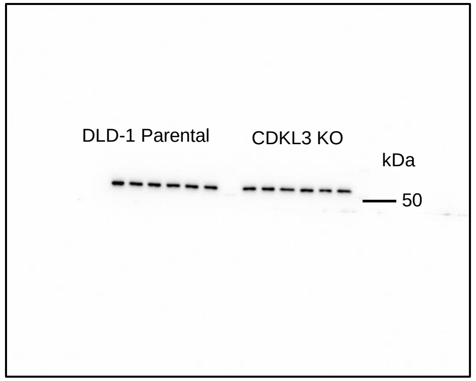

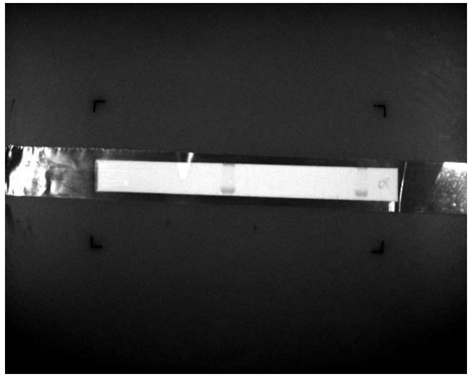

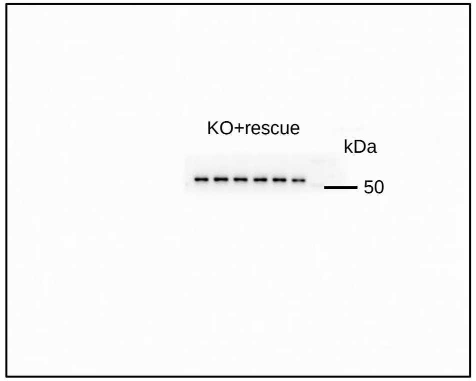

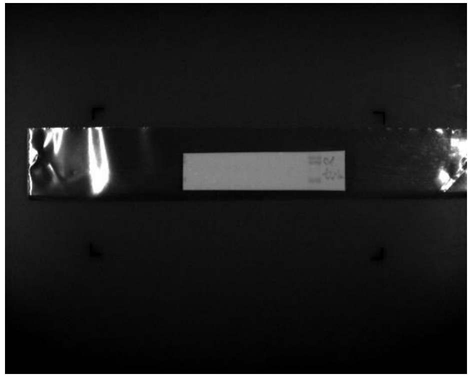

Full unedited blot for Supplemental Figure 3C

V5-Ub

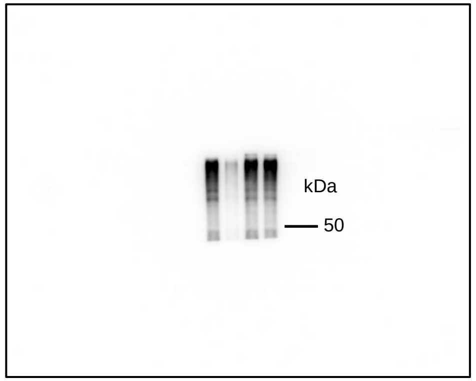

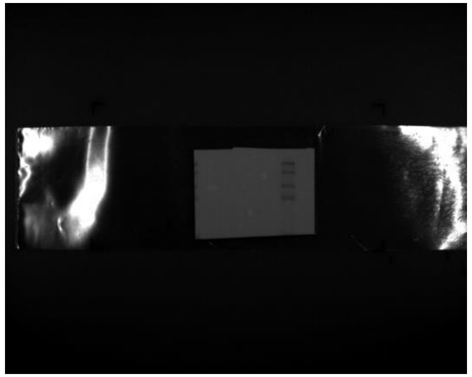

MYC-
CDK4

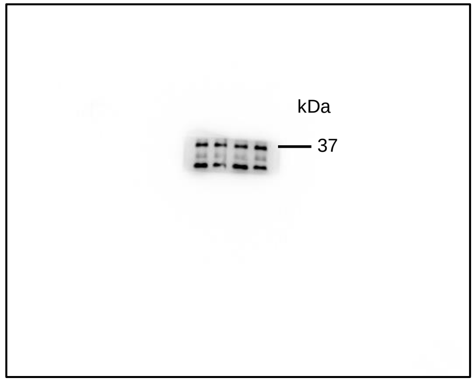

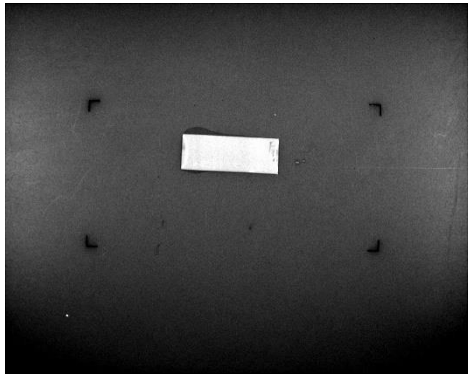

FLAG-
CDKL3

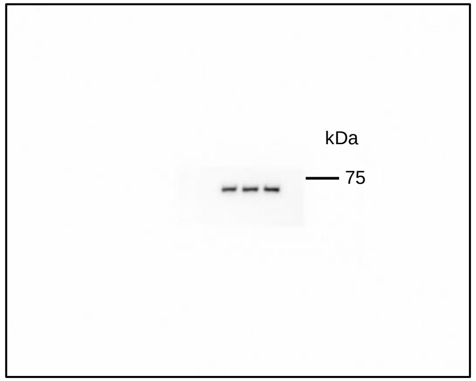

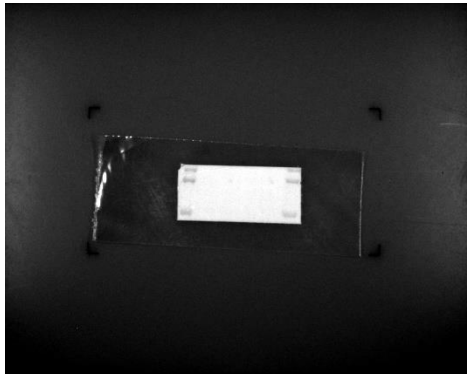

MYC-
CDK4

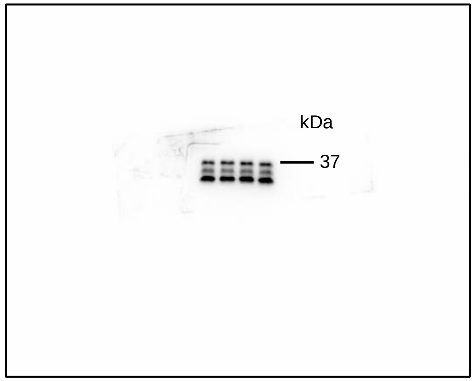

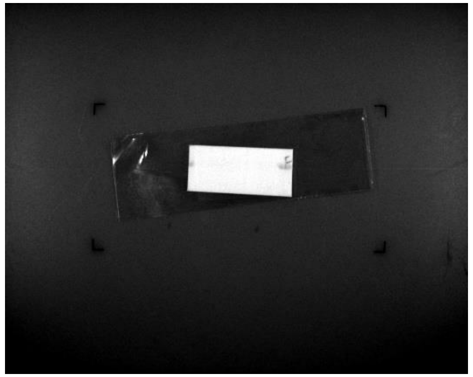

Full unedited blot for Supplemental Figure 3D

CDK4

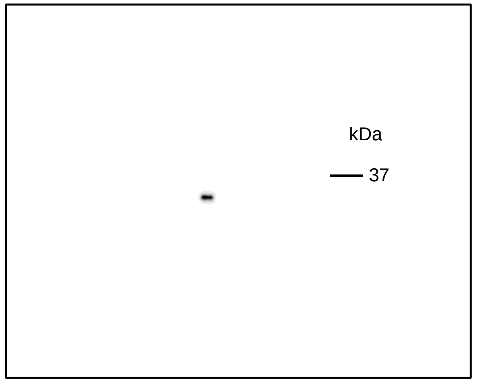

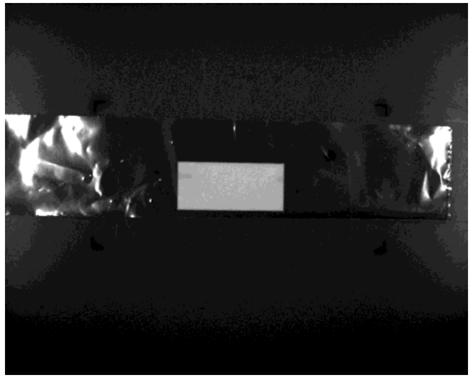

GAPDH

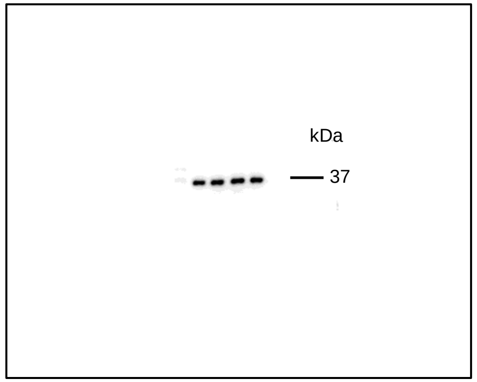

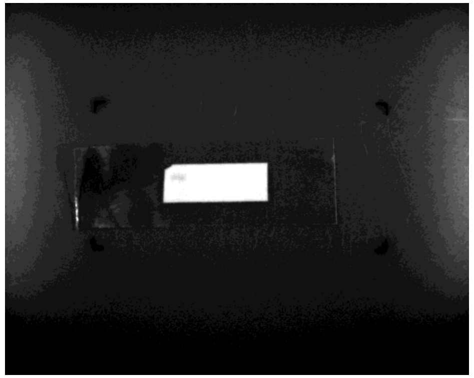

Full unedited blot for Supplemental Figure 3E

CDK4

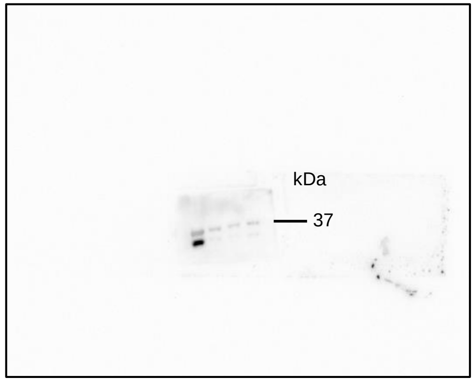

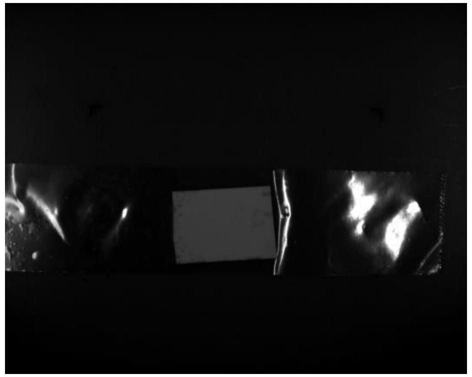

GAPDH

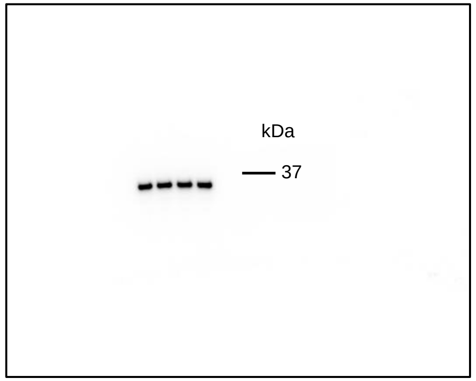

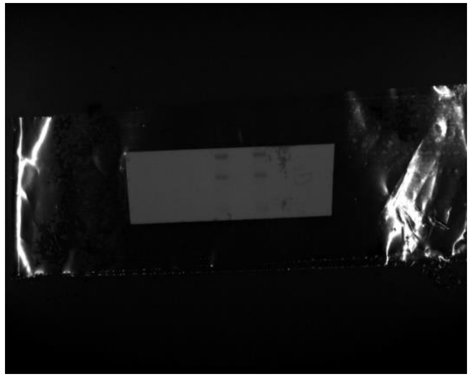

Full unedited blot for Supplemental Figure 3F

CDK4

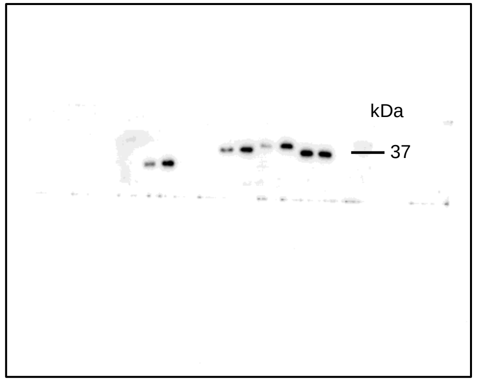

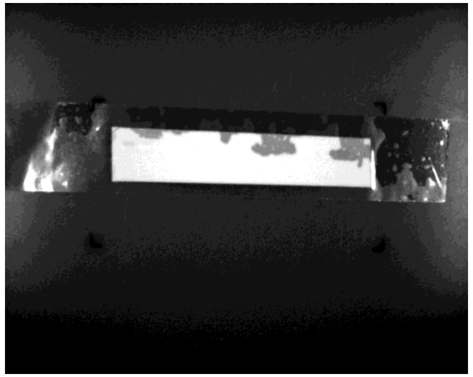

GAPDH

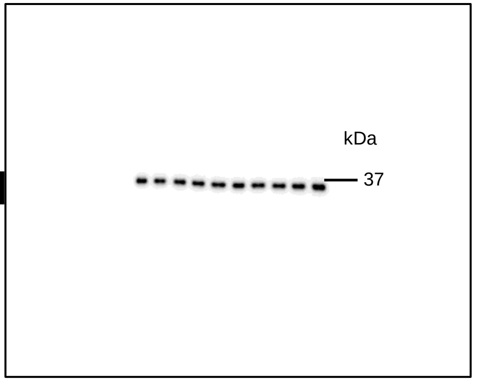

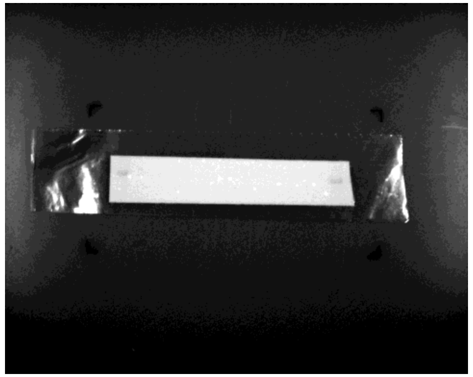

Full unedited blot for Supplemental Figure 3G

CDK4

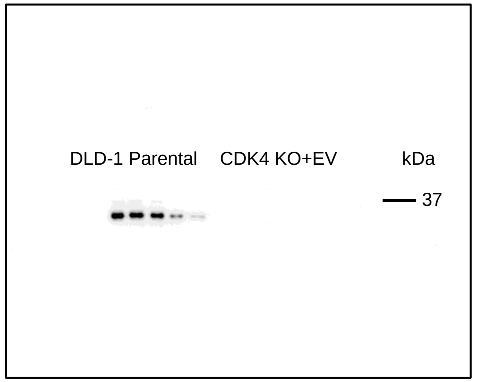

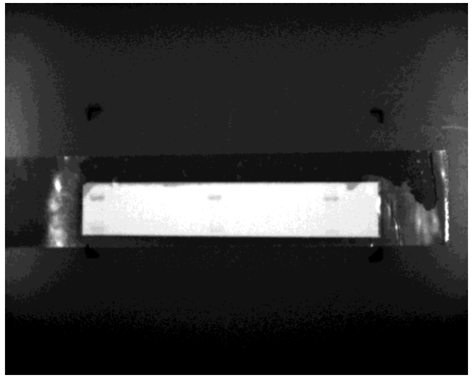

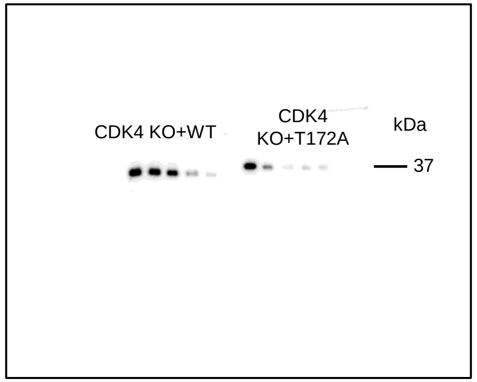

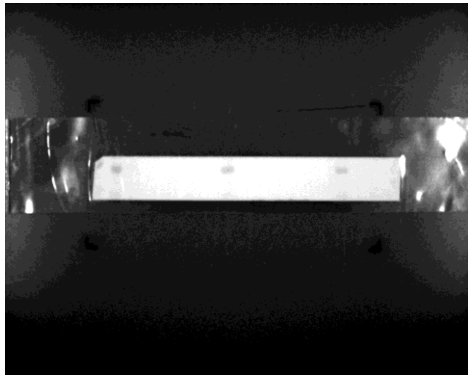

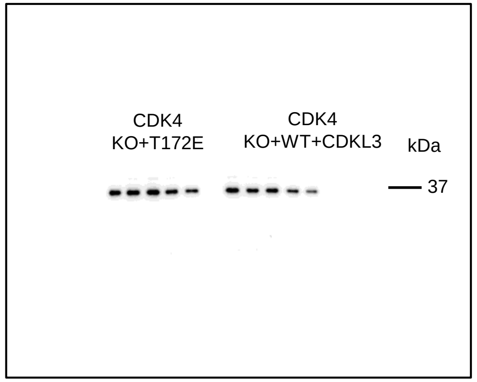

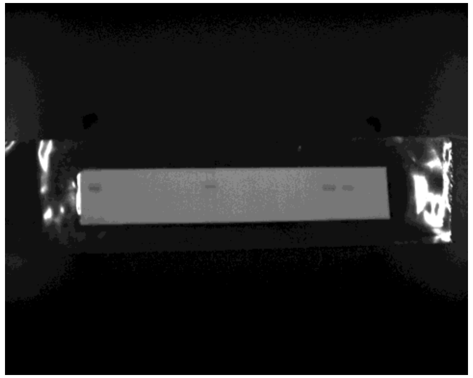

Full unedited blot for Supplemental Figure 3G

MYC-CDKL3

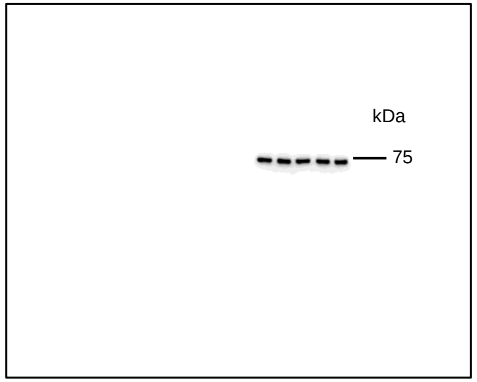

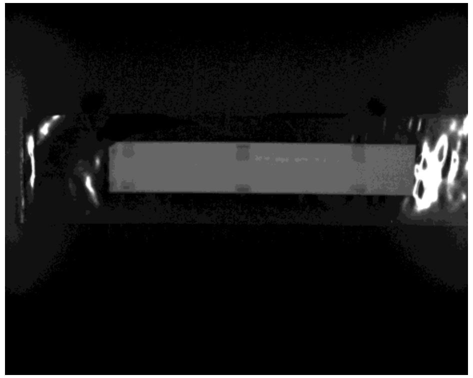

GAPDH

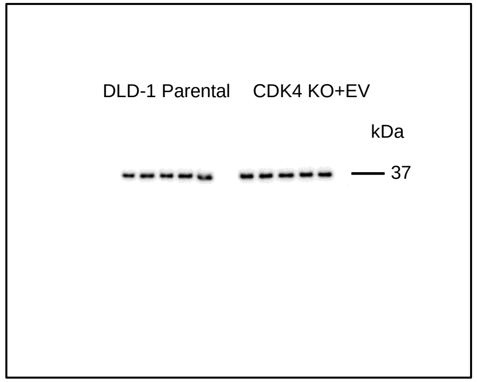

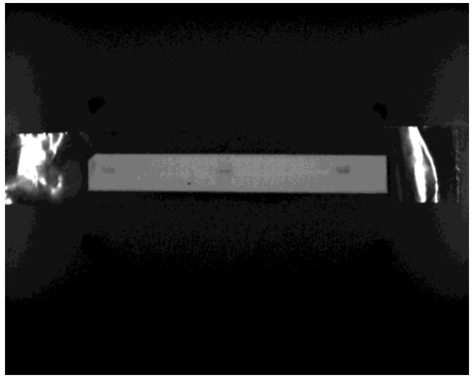

GAPDH

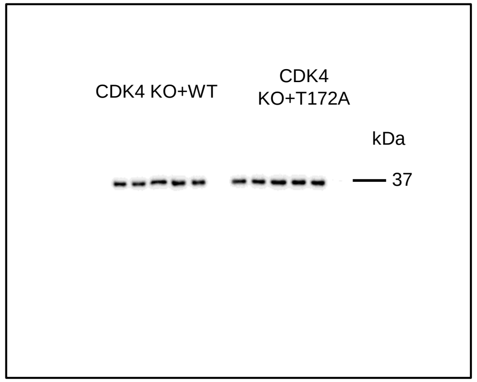

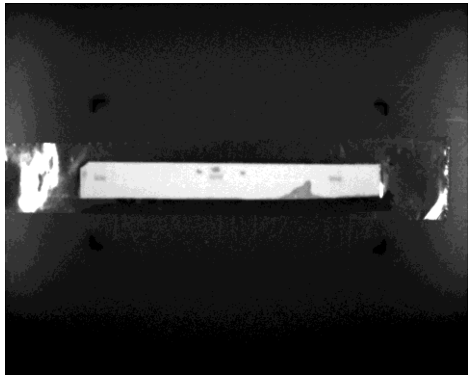

GAPDH

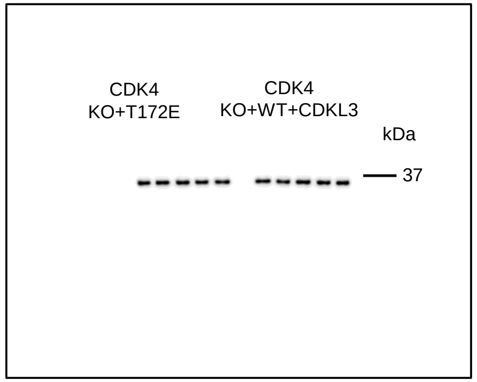

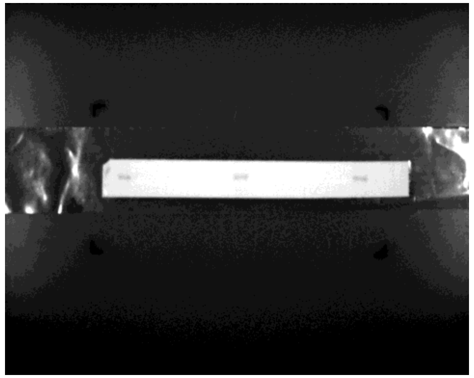

Full unedited blot for Supplemental Figure 3H

MYC-CDK4

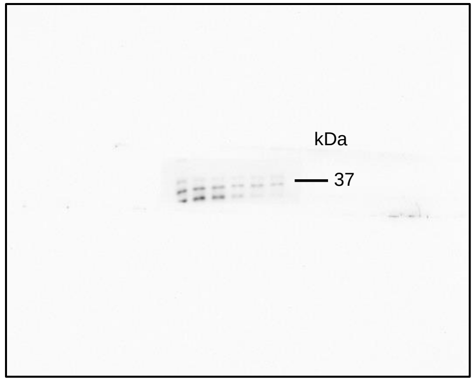

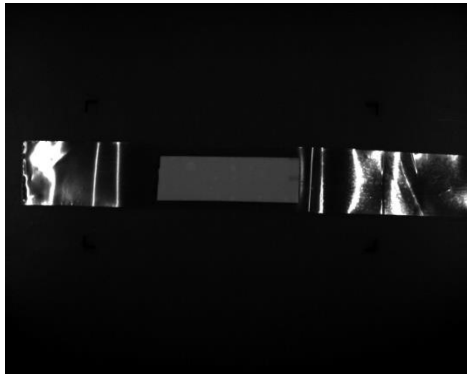

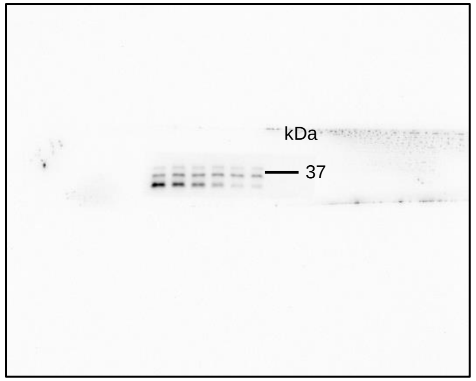

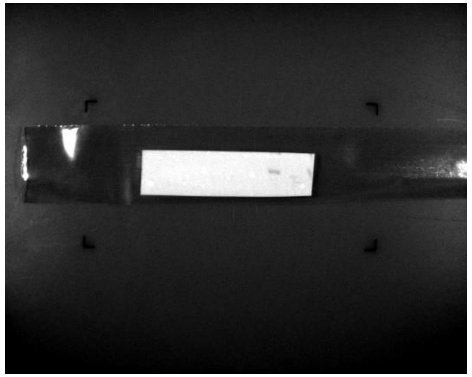

Full unedited blot for Supplemental Figure 3H

MYC-CDK4

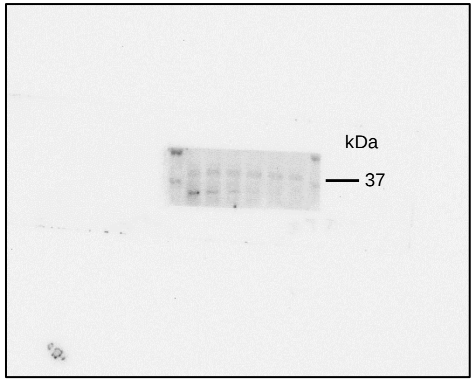

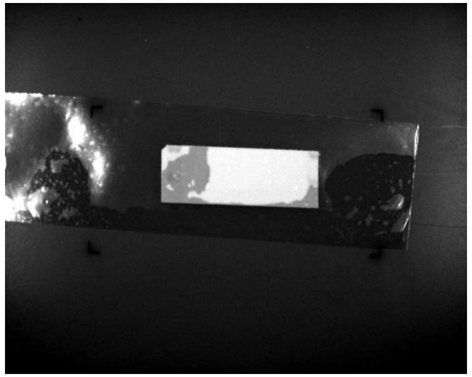

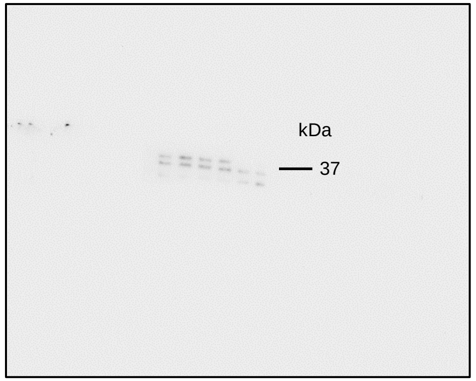

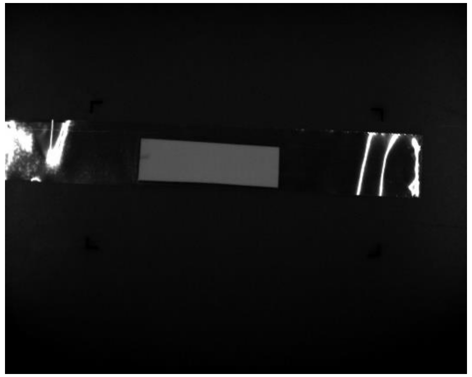

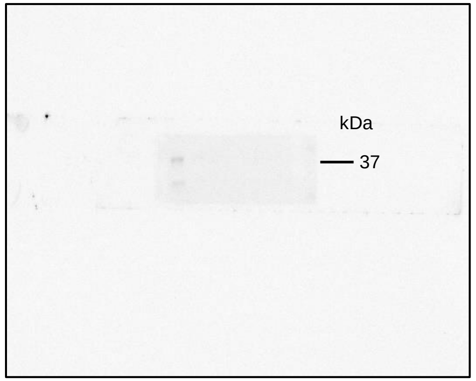

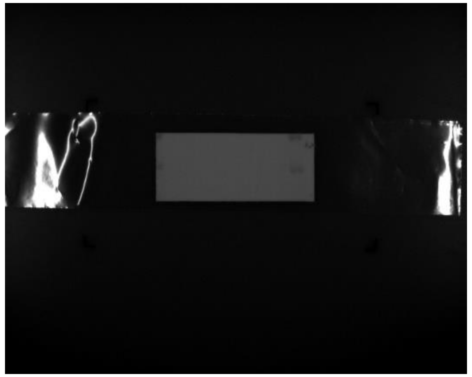

Full unedited blot for Supplemental Figure 3H

FLAG-CDKL3

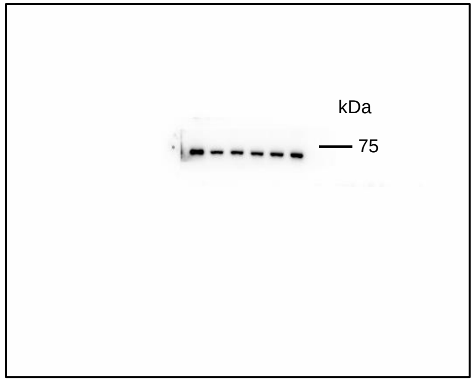

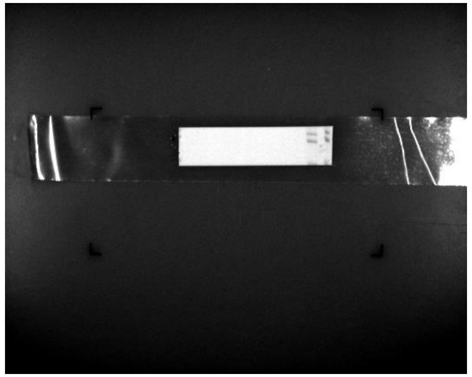

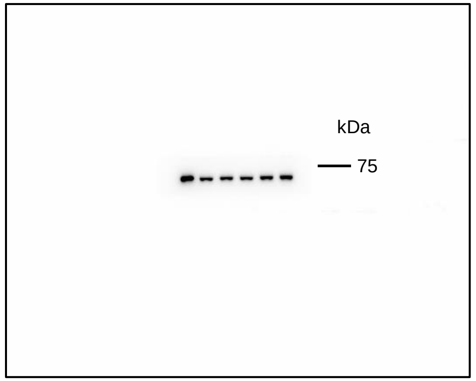

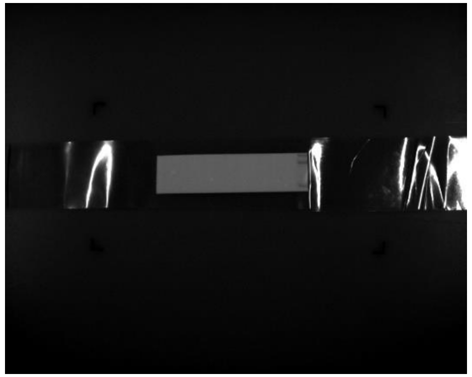

Full unedited blot for Supplemental Figure 3H

GAPDH

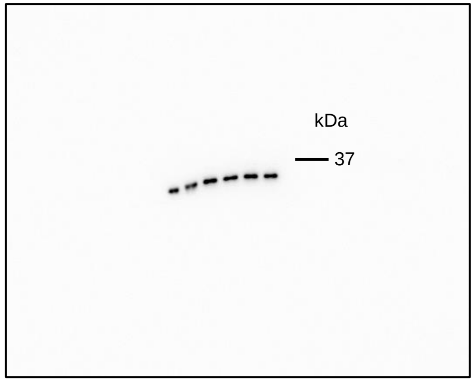

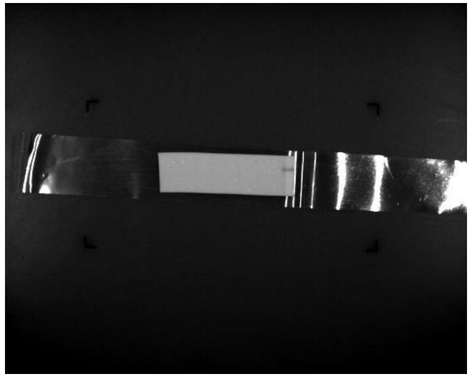

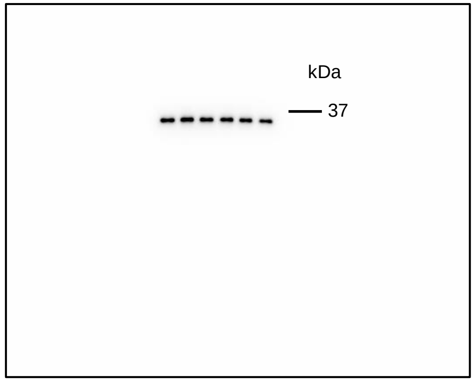

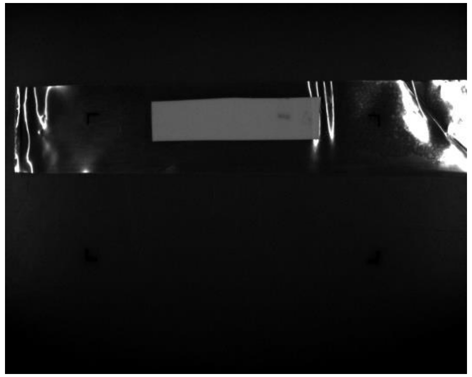

Full unedited blot for Supplemental Figure 3H

GAPDH

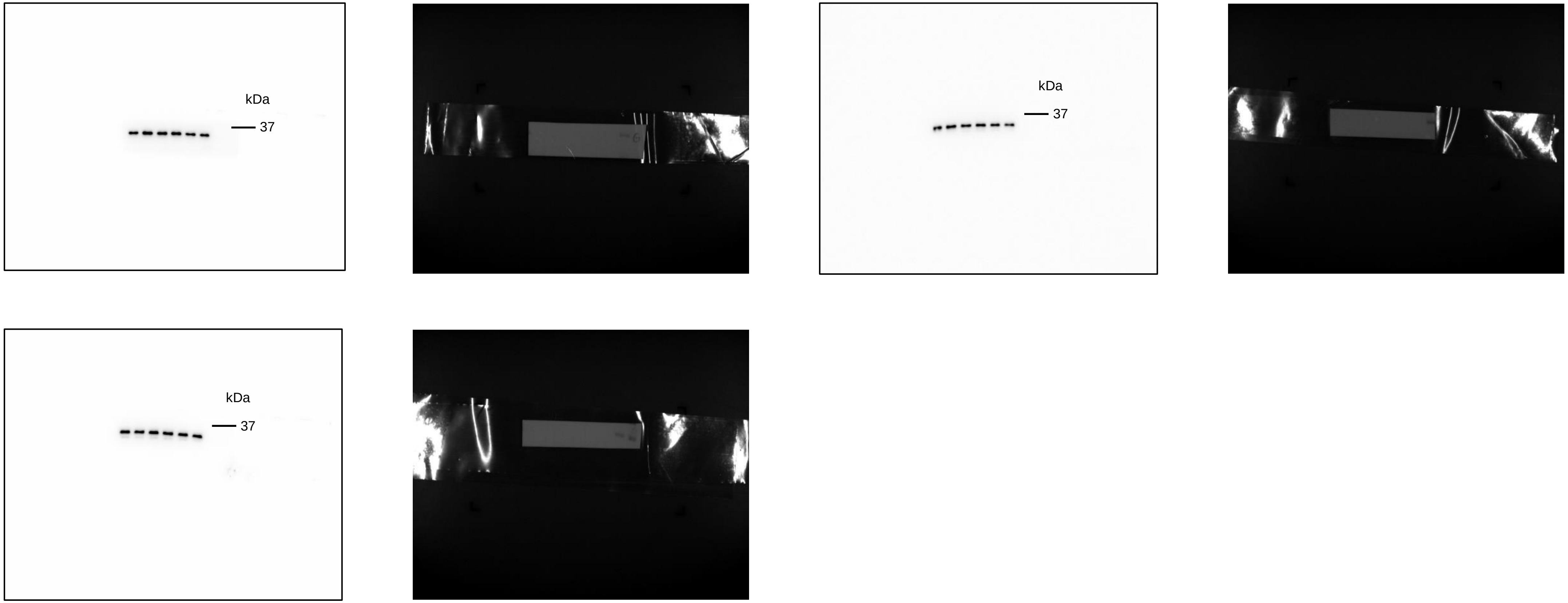

Full unedited blot for Supplemental Figure 3J

CDK4

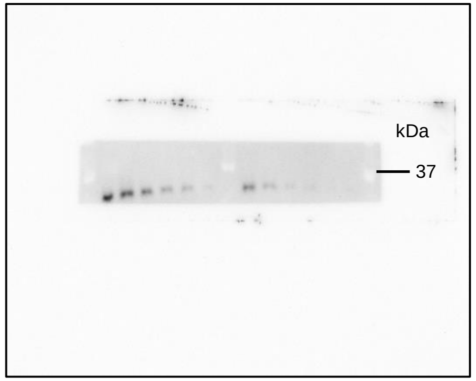

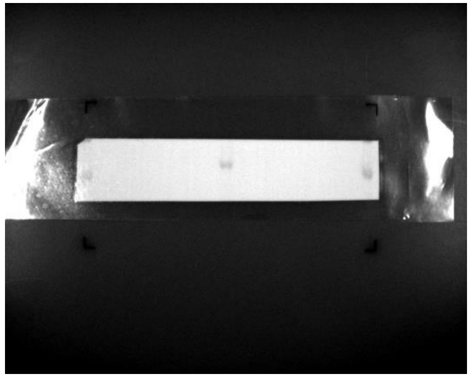

CDKL3

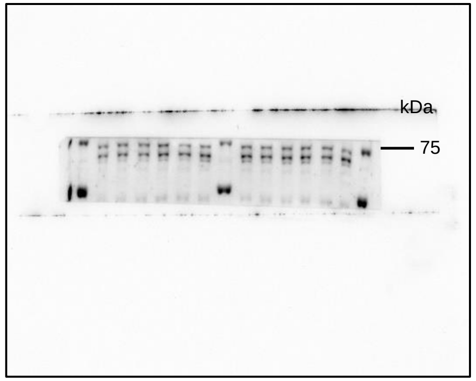

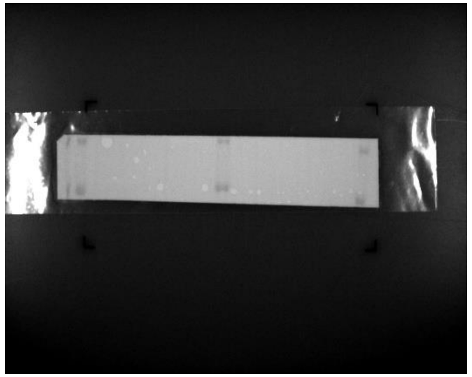

cyclinA2

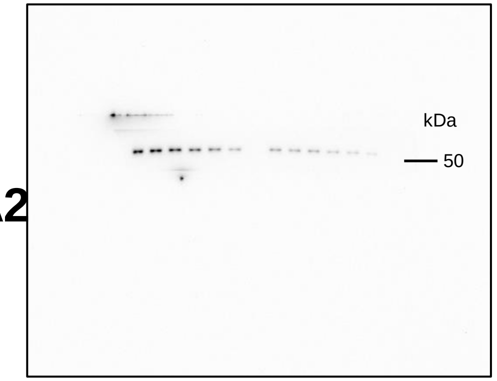

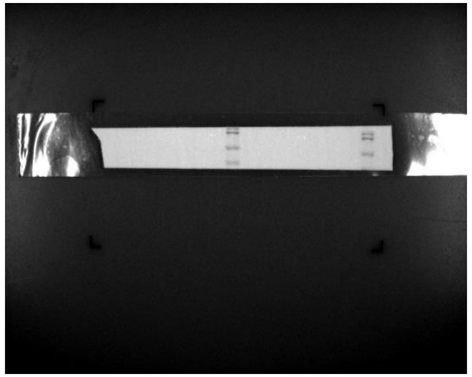

GAPDH

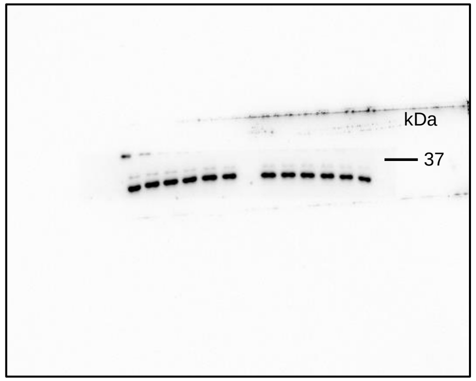

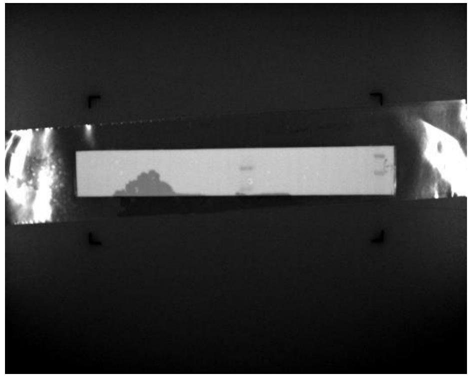

Full unedited blot for Supplemental Figure 3J

CDK4

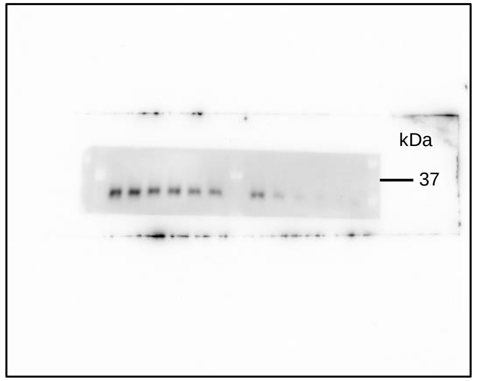

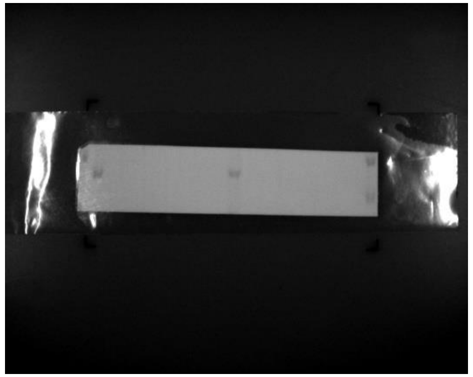

CDKL3

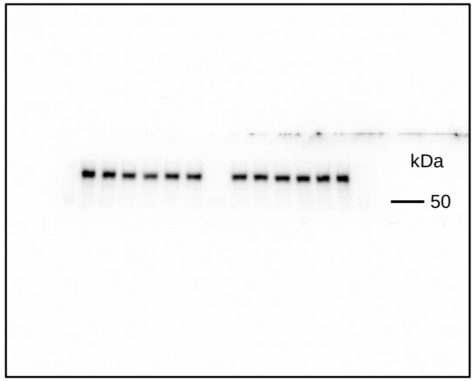

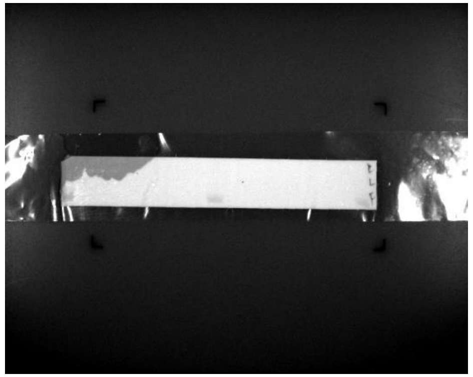

cyclinA

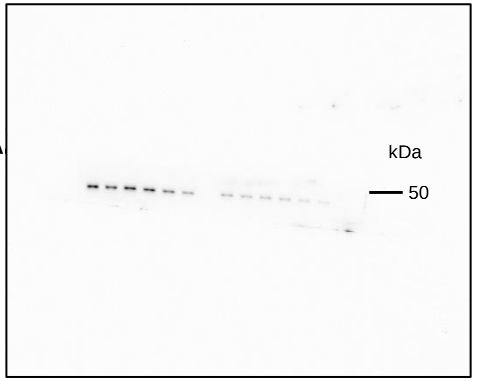

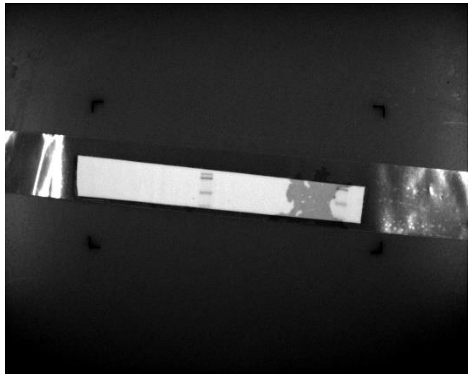

GAPDH

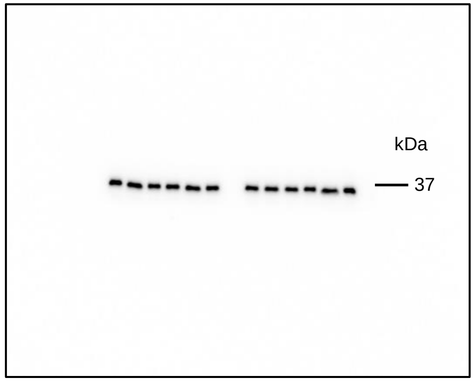

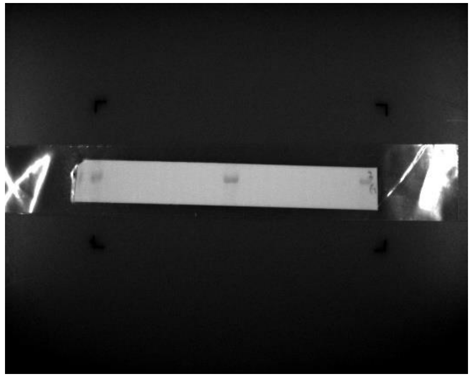

Full unedited blot for Supplemental Figure 3K

cyclinD1

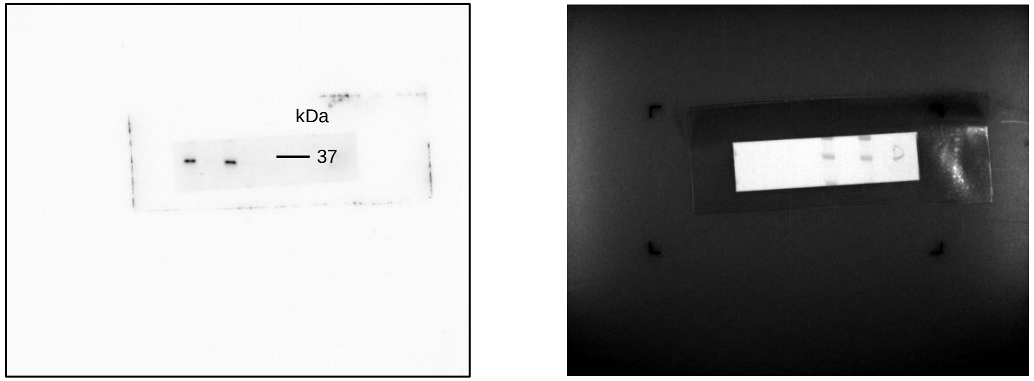

cyclinD2

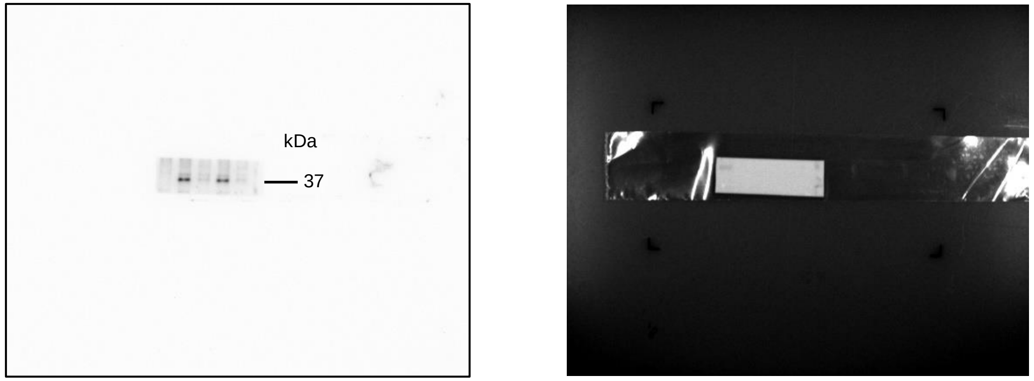

cyclinD3

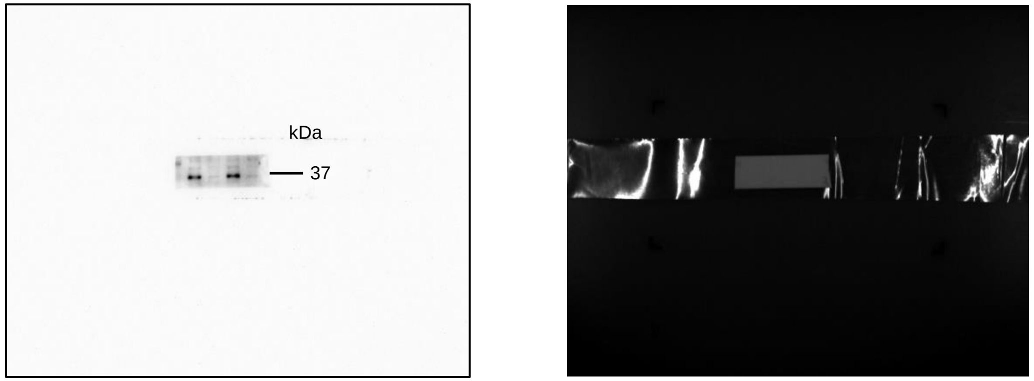

α-tubulin

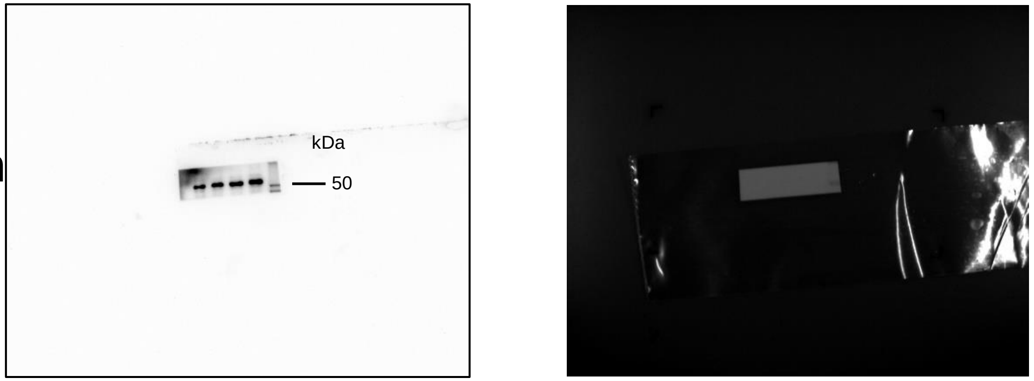

Full unedited blot for Supplemental Figure 3L

CDK4

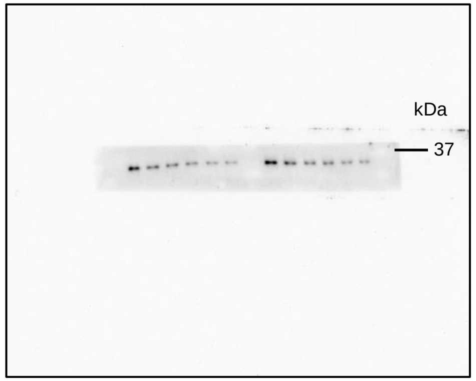

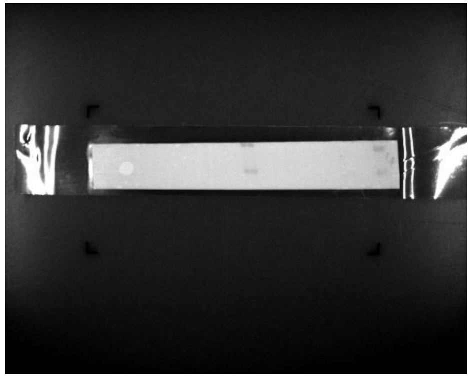

CDKL3

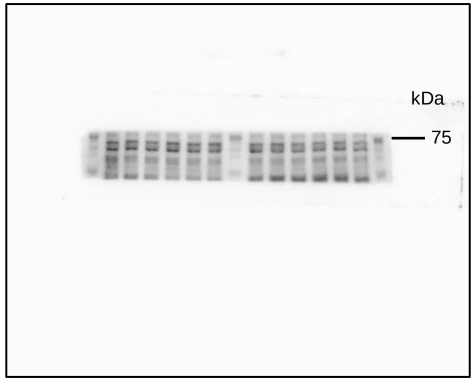

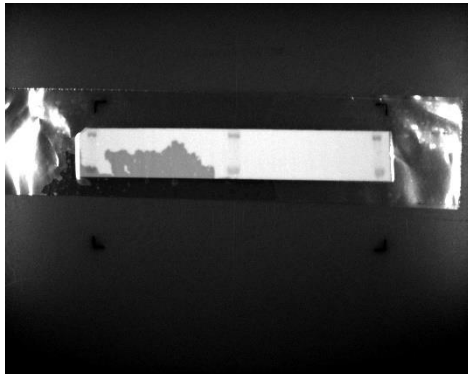

α-tubulin

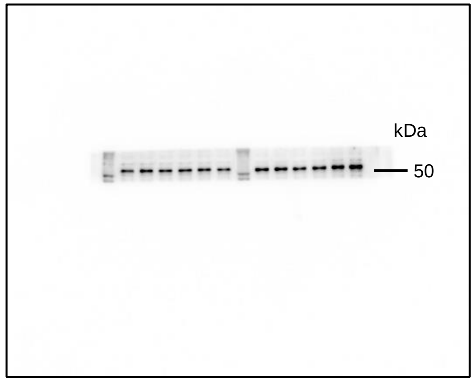

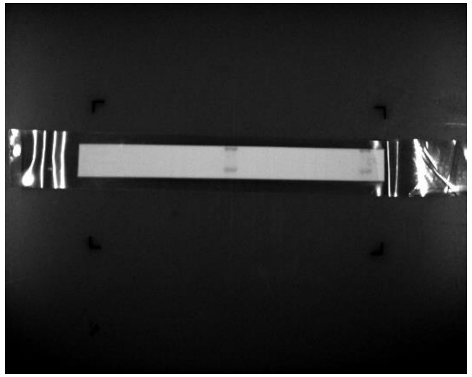

Full unedited blot for Supplemental Figure 3L

CDK4

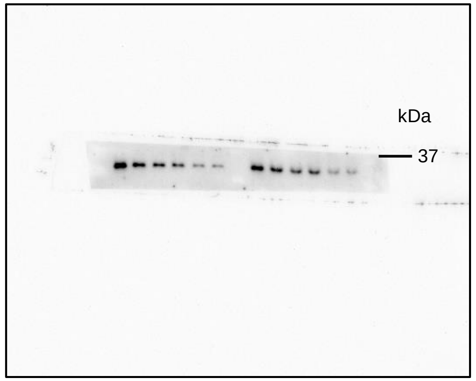

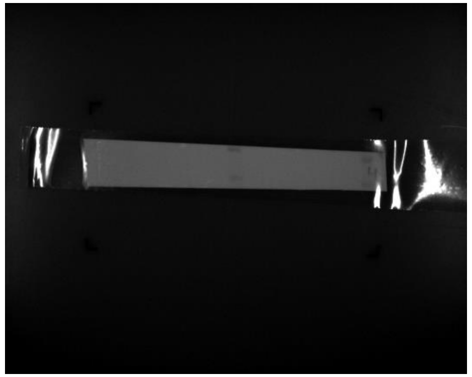

FLAG
-CDKL3

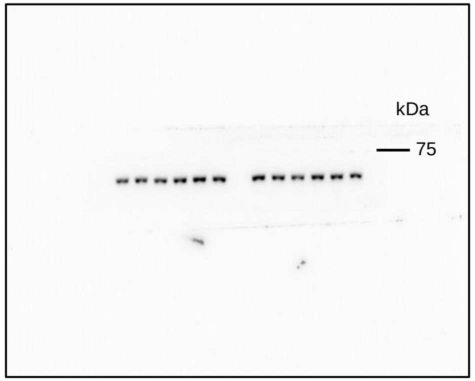

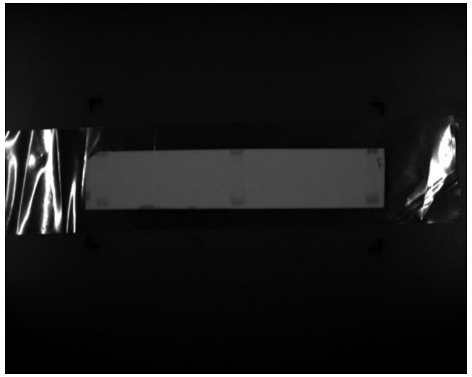

α-tubulin

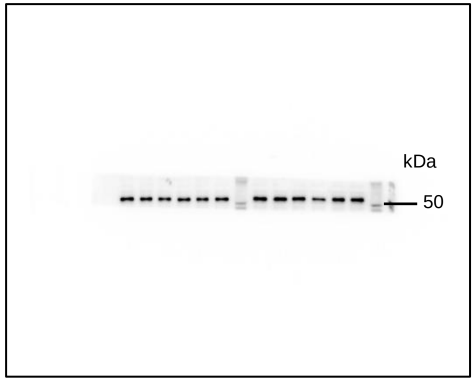

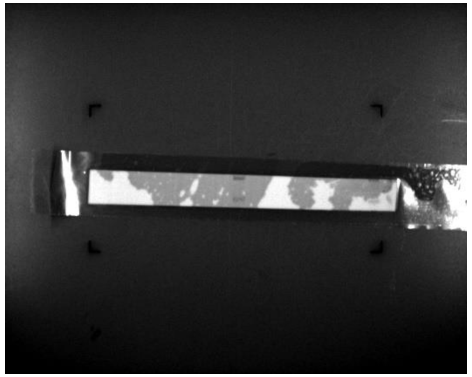

Full unedited blot for Supplemental Figure 3Q

pRb

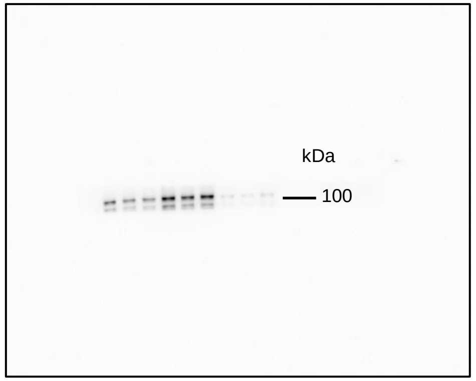

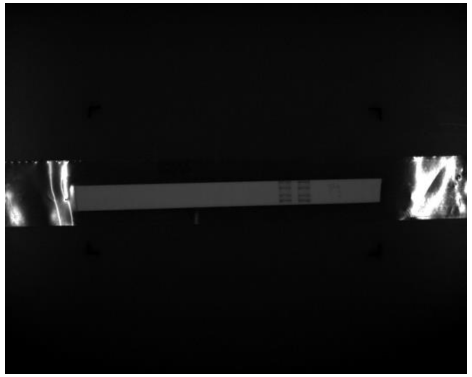

Rb

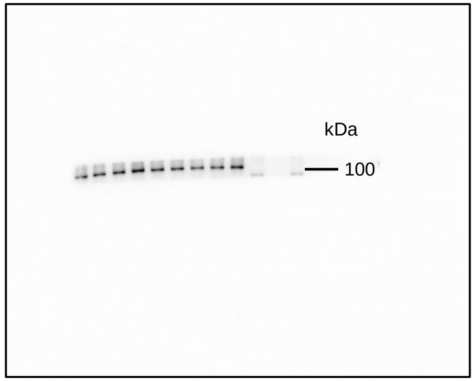

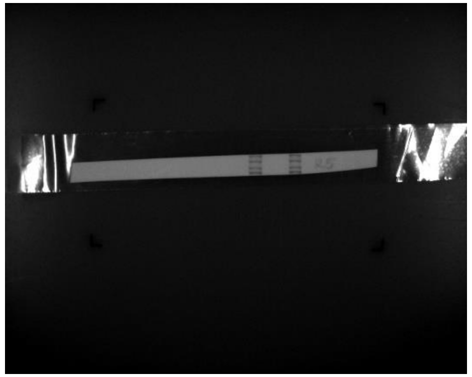

Full unedited blot for Supplemental Figure 3Q

CDKL3

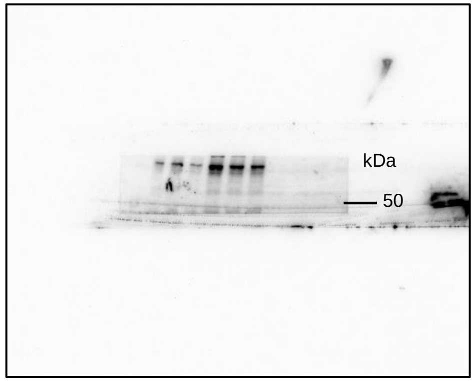

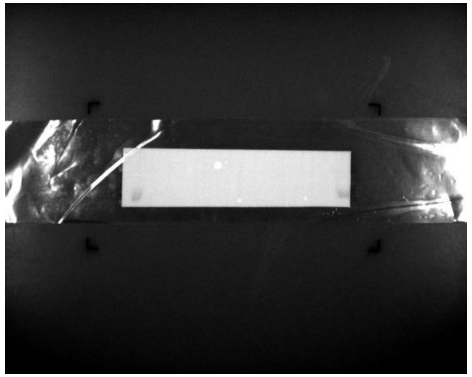

CDK4

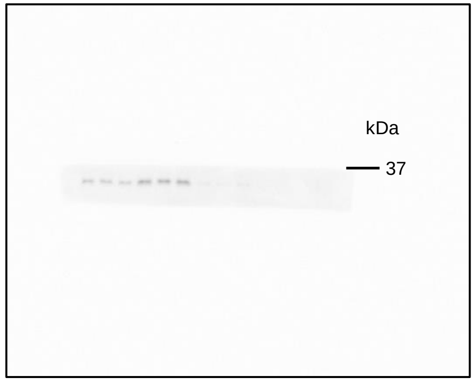

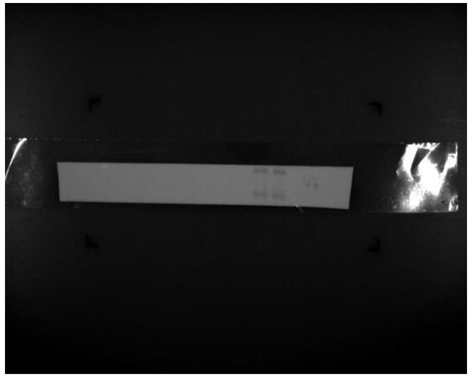

α-tubulin

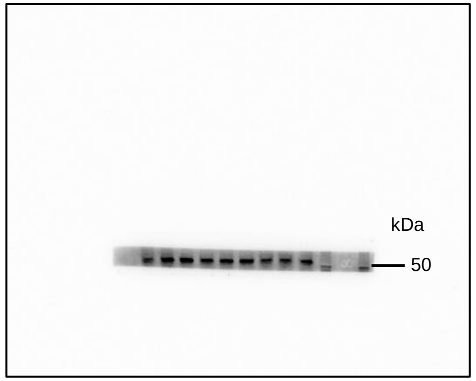

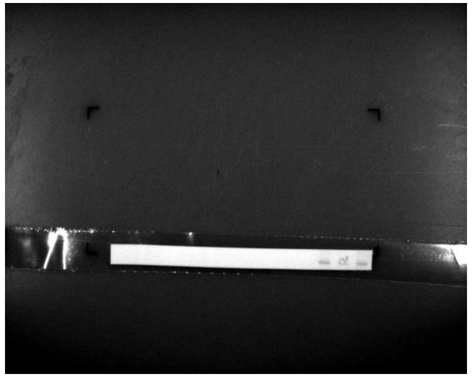

Full unedited blot for Supplemental Figure 4B

Trim28

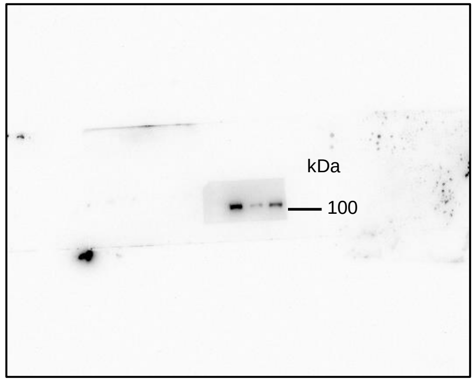

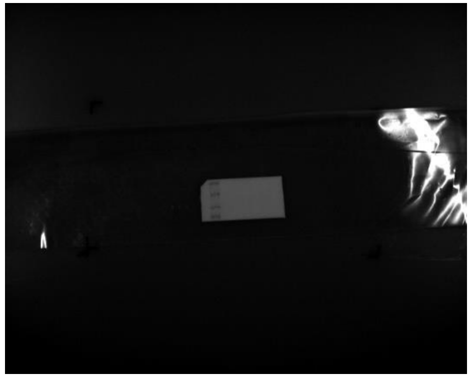

GAPDH

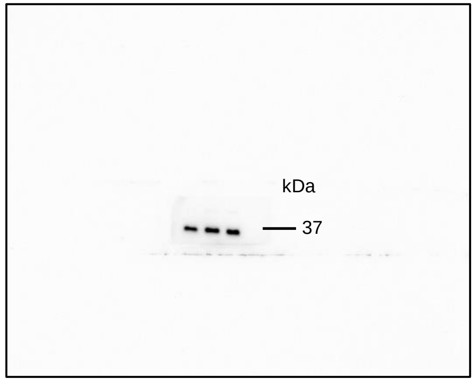

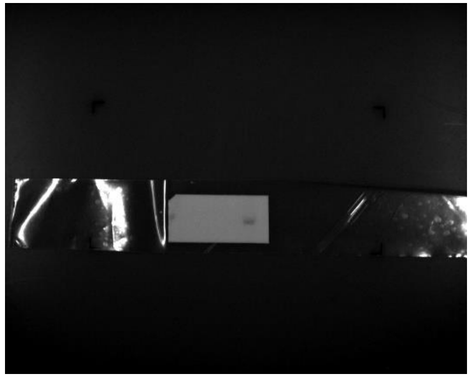

Full unedited blot for Supplemental Figure 4D

Trim28

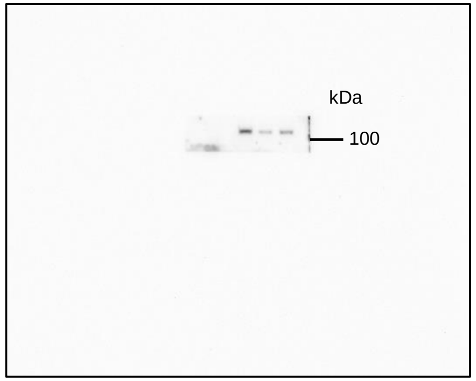

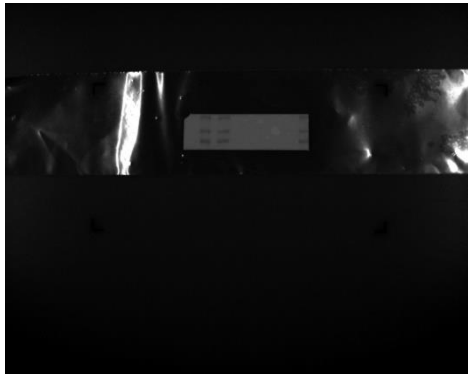

GAPDH

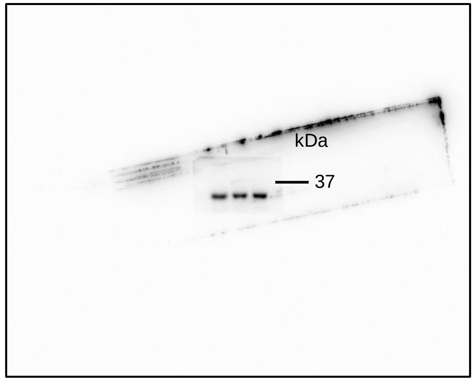

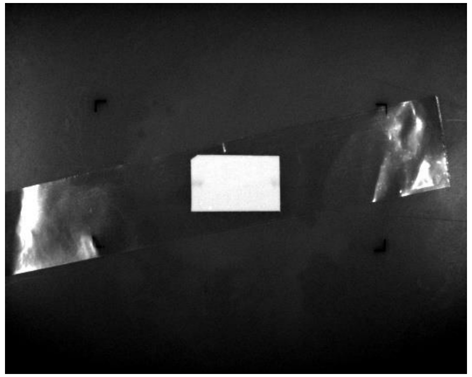

Full unedited blot for Supplemental Figure 4E

CDK4

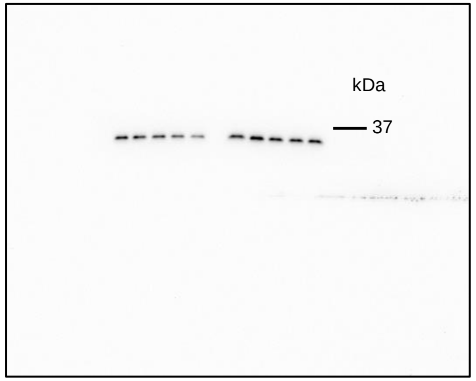

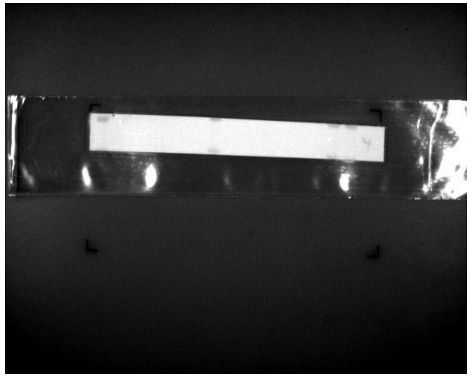

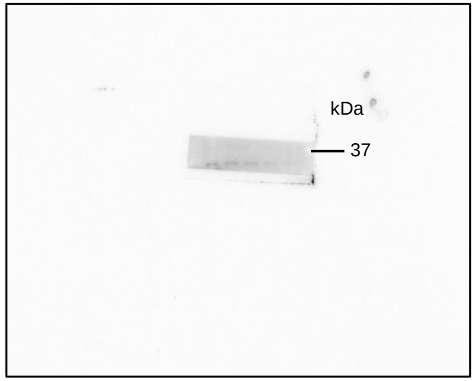

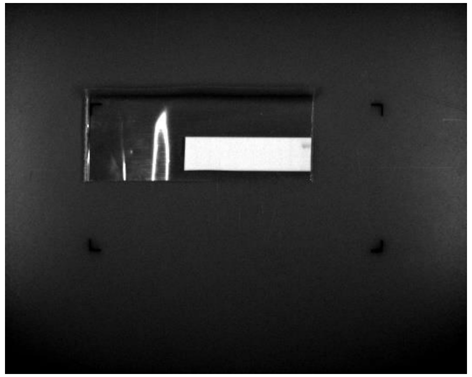

Trim28

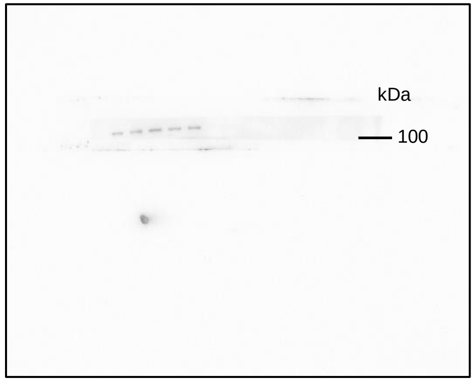

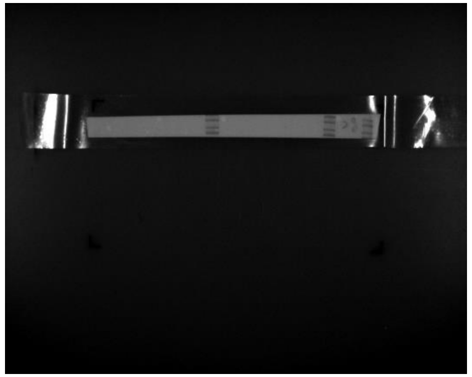

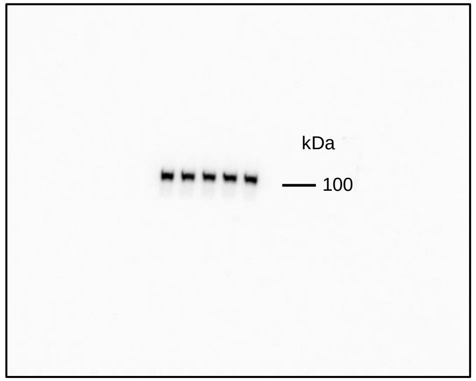

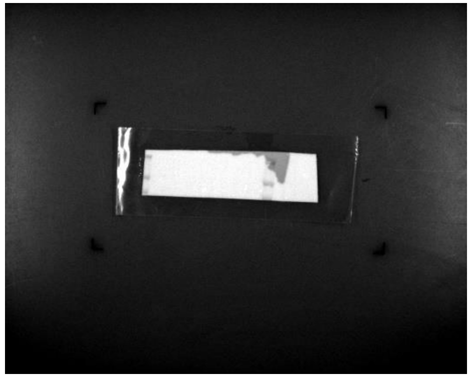

Full unedited blot for Supplemental Figure 4E

α-tubulin

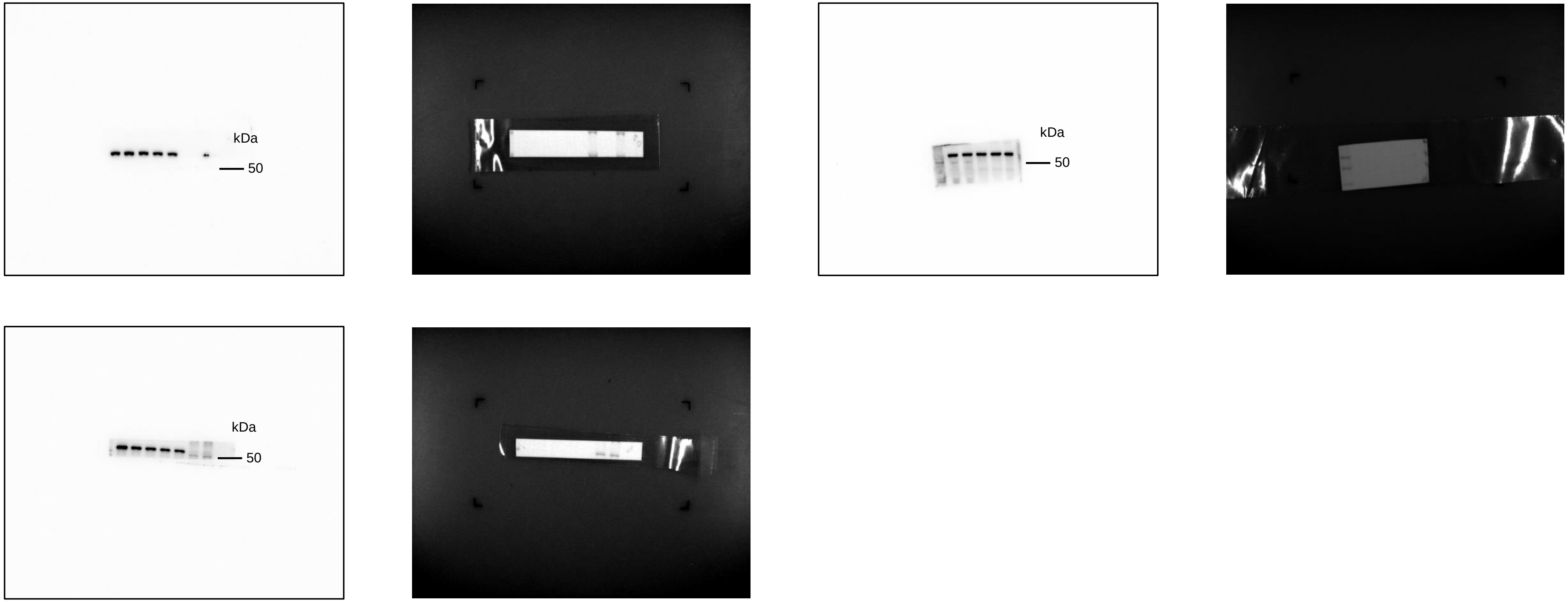

Full unedited blot for Supplemental Figure 5B

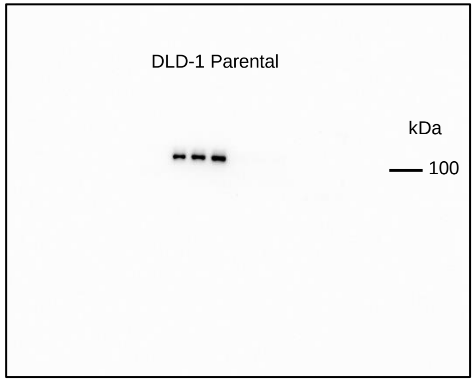

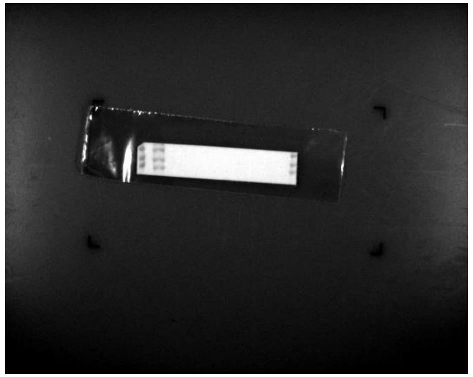

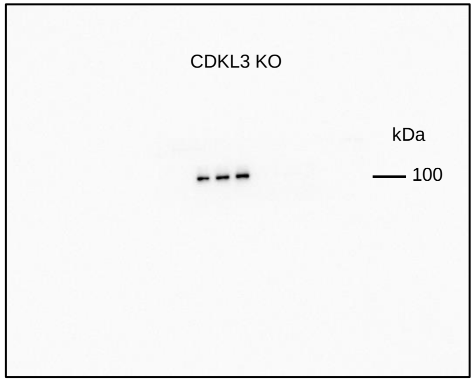

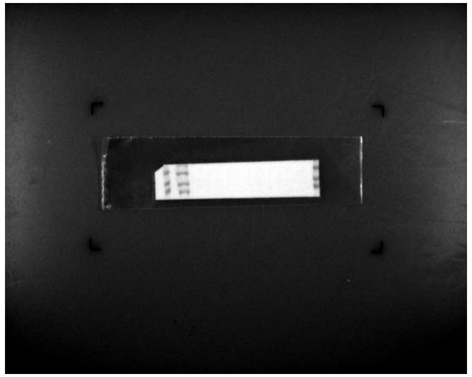

pRb

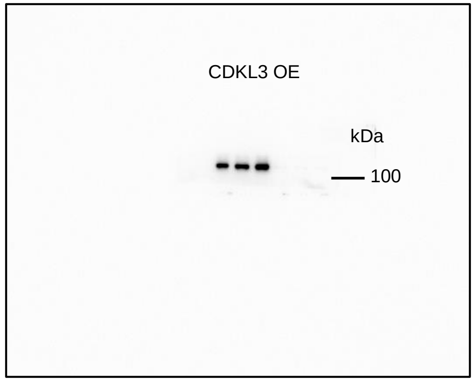

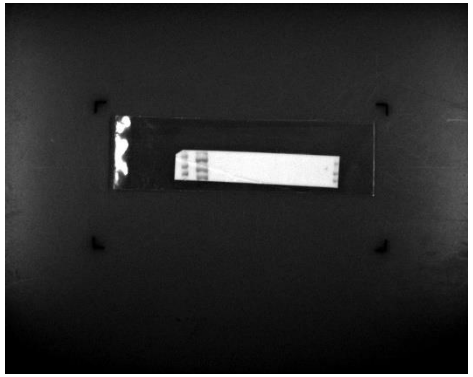

Full unedited blot for Supplemental Figure 5B

Rb

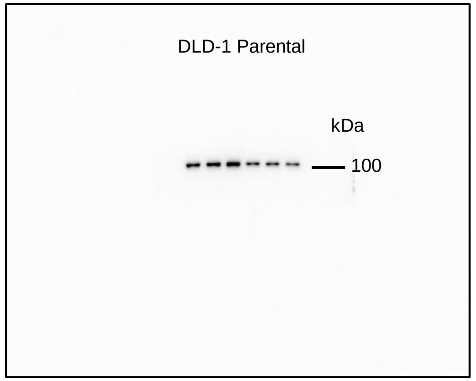

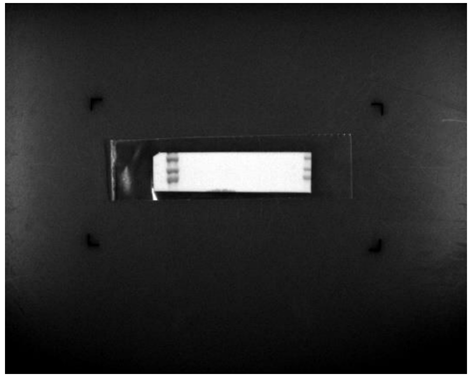

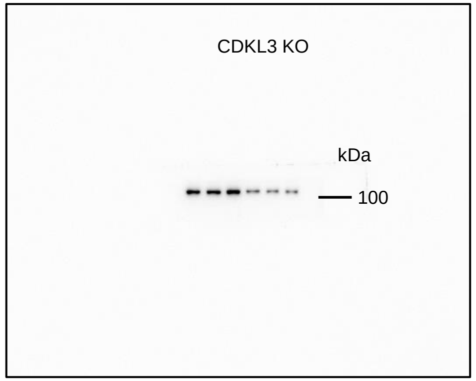

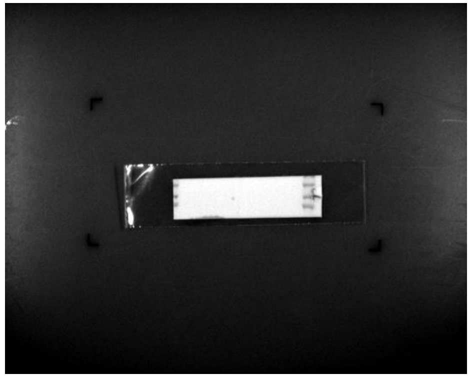

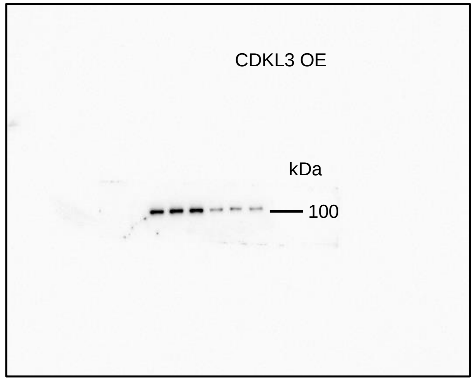

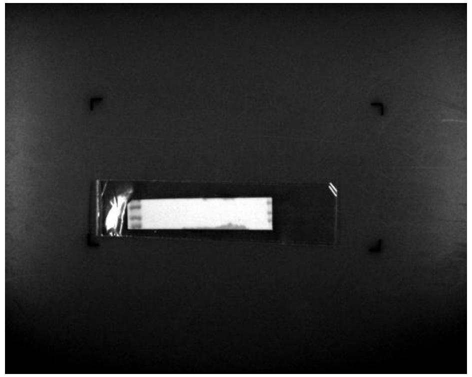

Full unedited blot for Supplemental Figure 5B

CDKL3

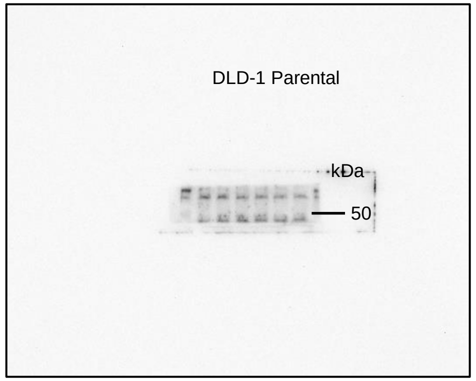

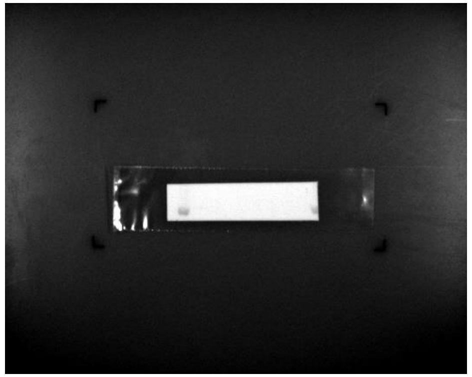

CDKL3

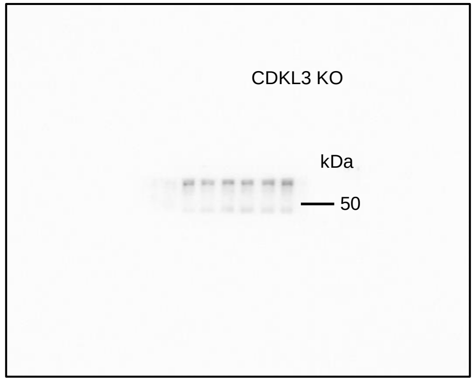

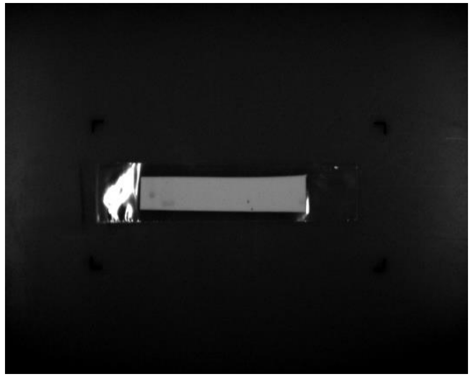

FLAG-CDKL3

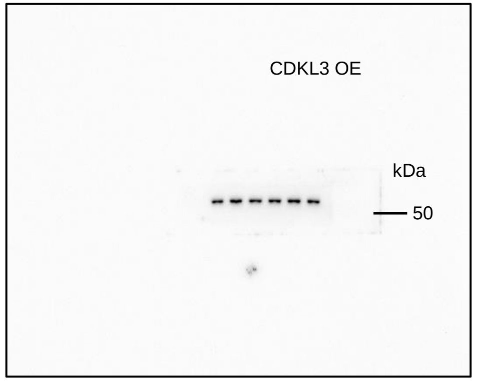

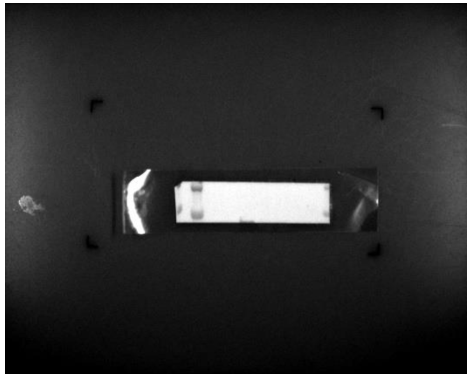

cyclinA2

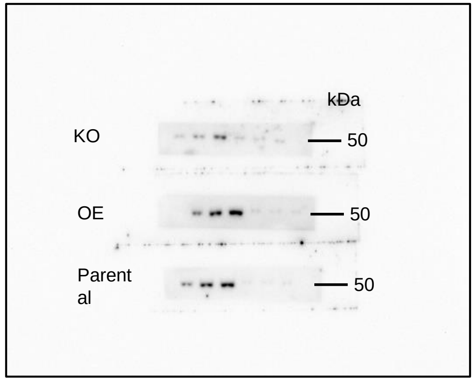

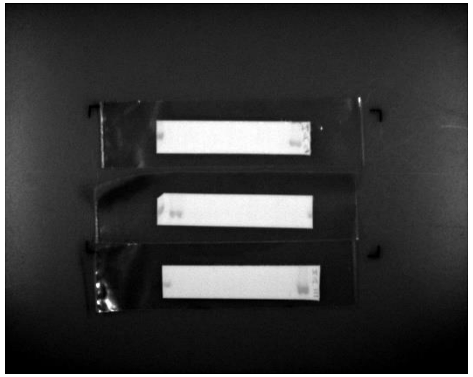

Full unedited blot for Supplemental Figure 5B

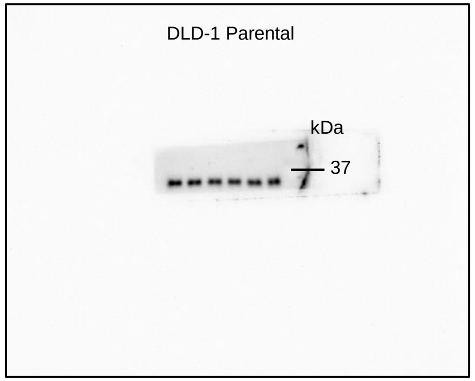

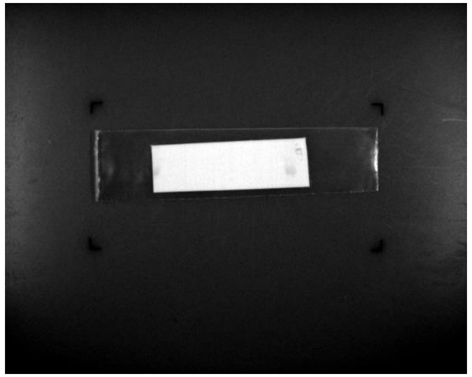

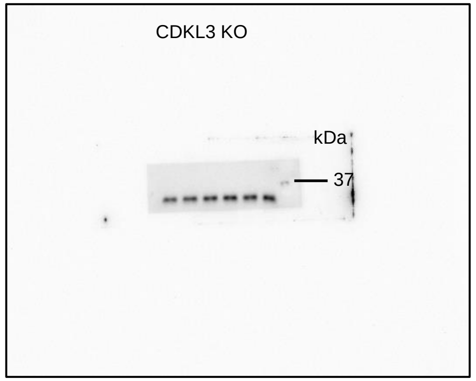

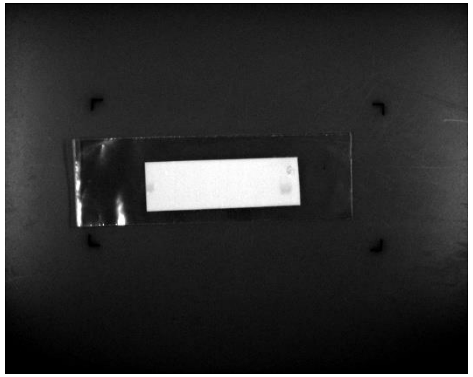

GAPDH

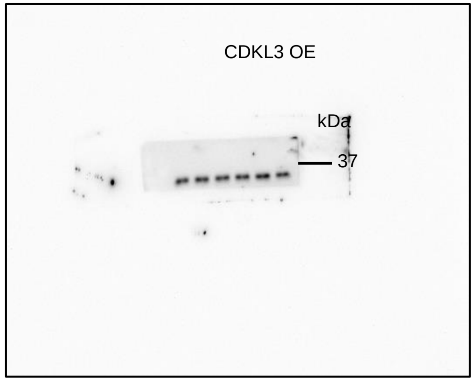

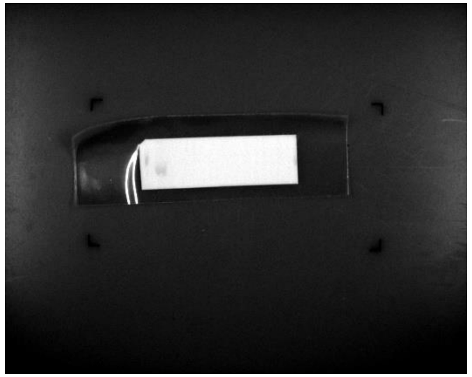

Full unedited blot for Supplemental Figure 5C

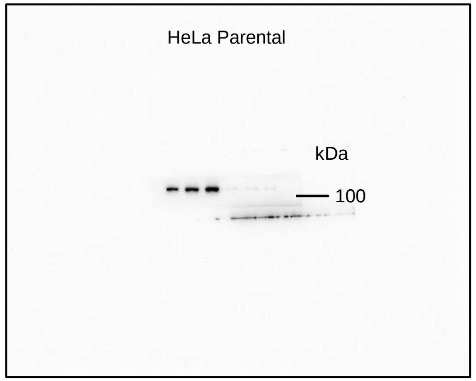

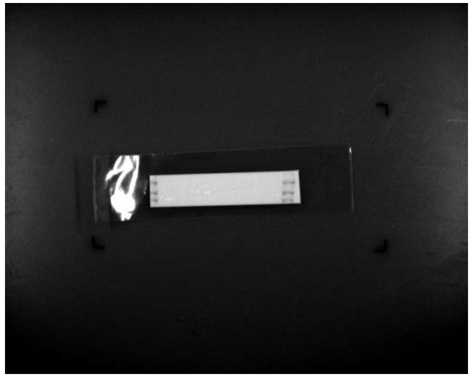

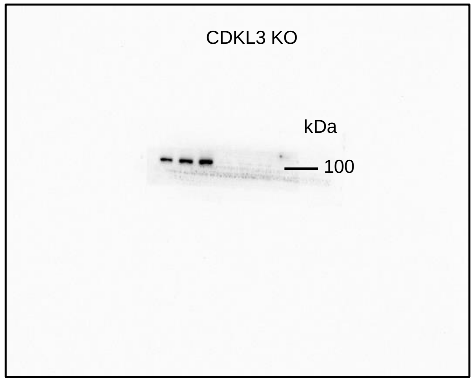

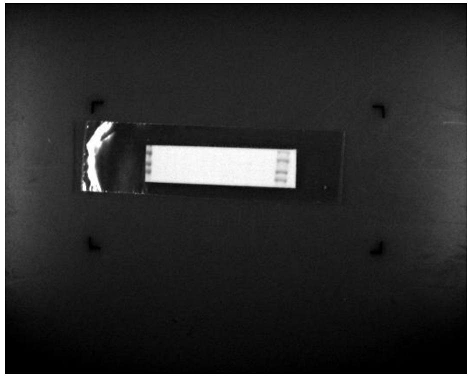

pRb

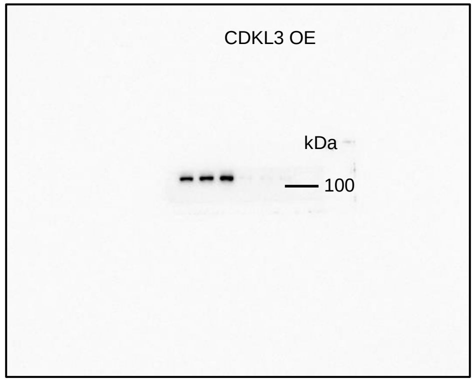

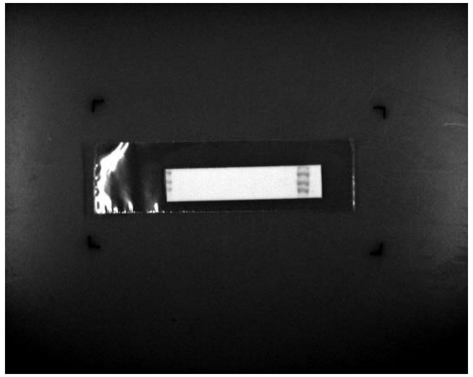

Full unedited blot for Supplemental Figure 5C

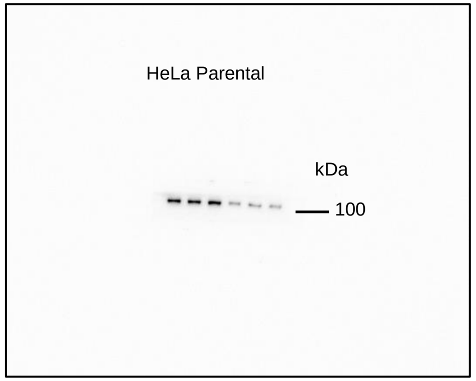

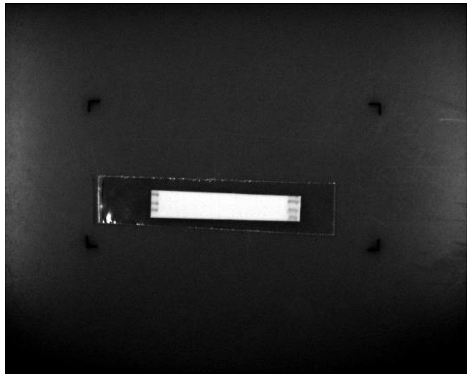

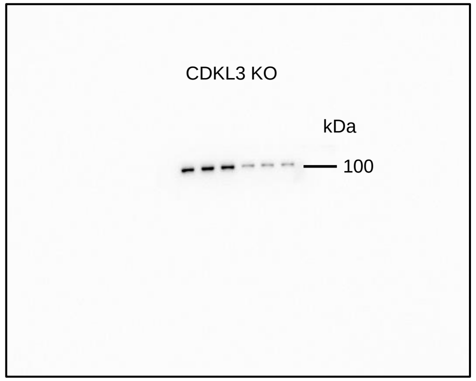

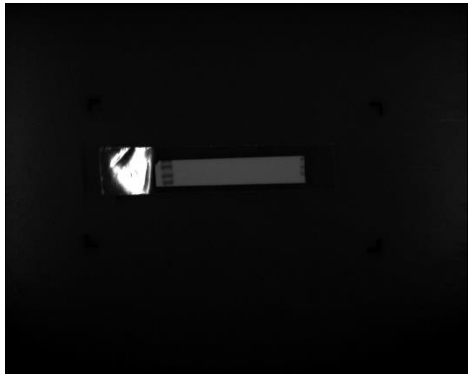

Rb

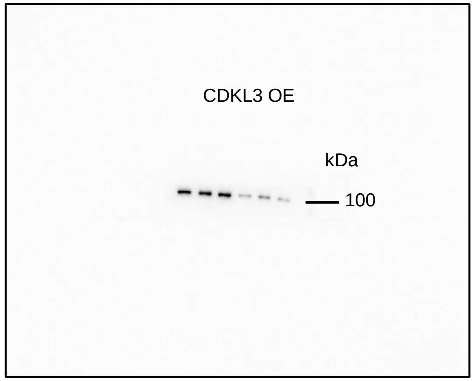

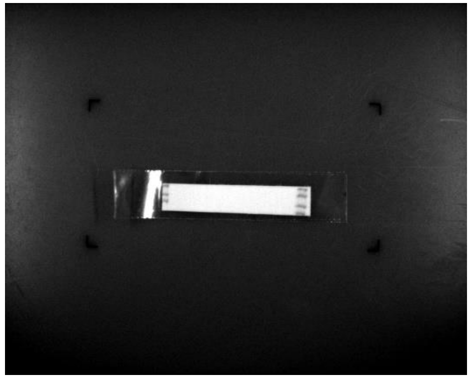

Full unedited blot for Supplemental Figure 5C

CDKL3

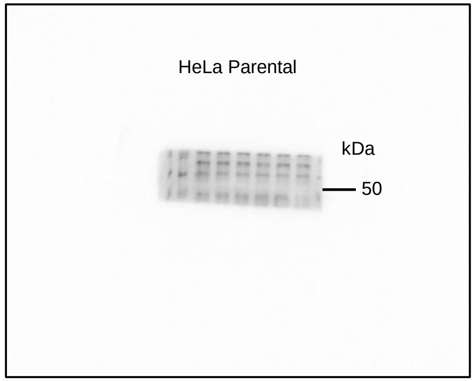

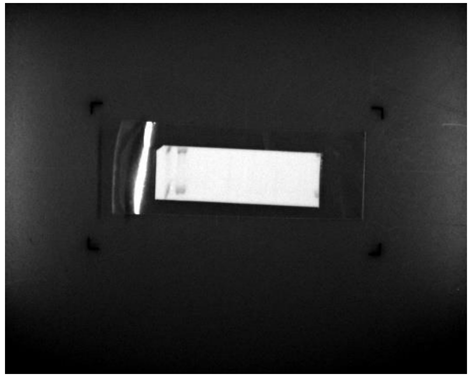

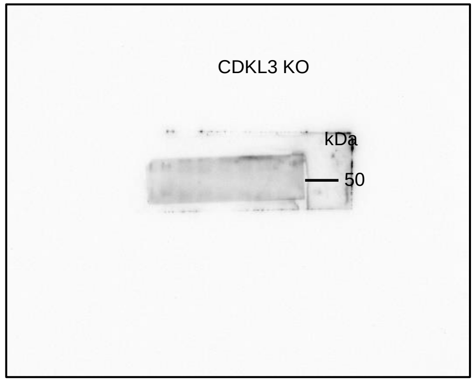

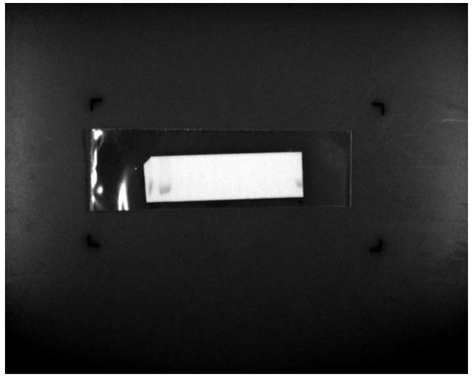

FLAG-
CDKL3

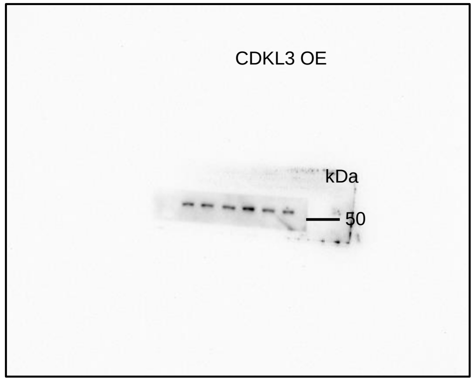

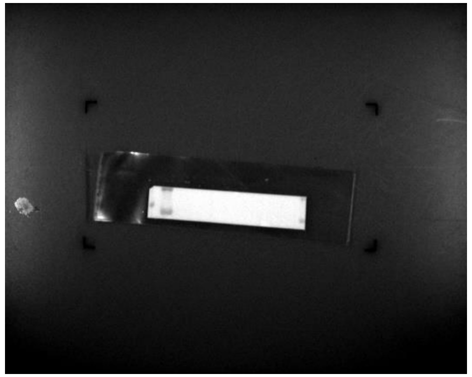

Full unedited blot for Supplemental Figure 5C

cyclinA2

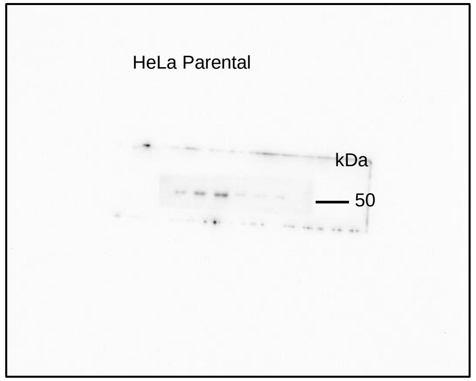

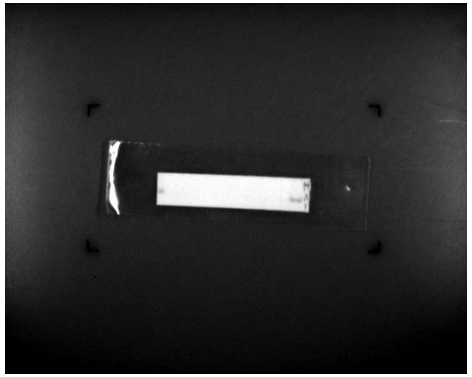

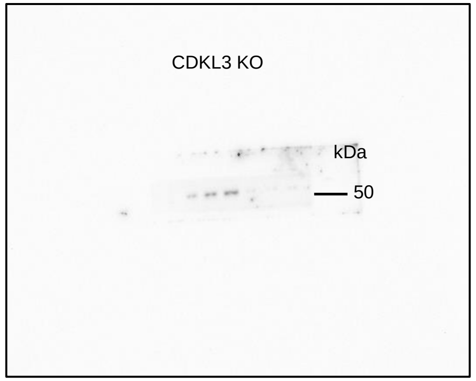

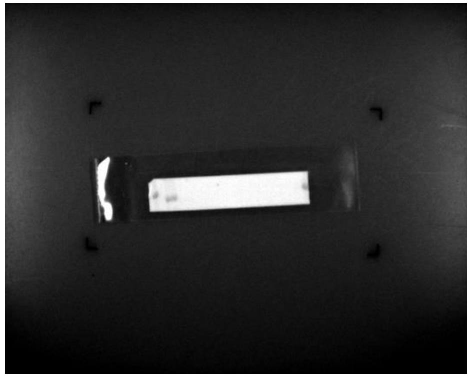

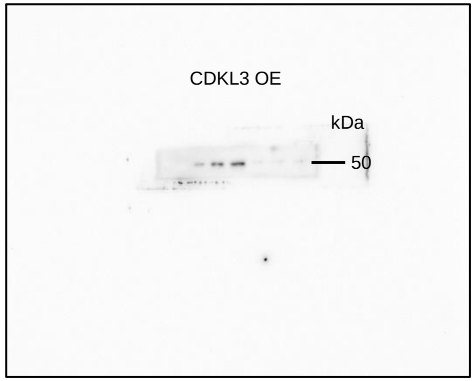

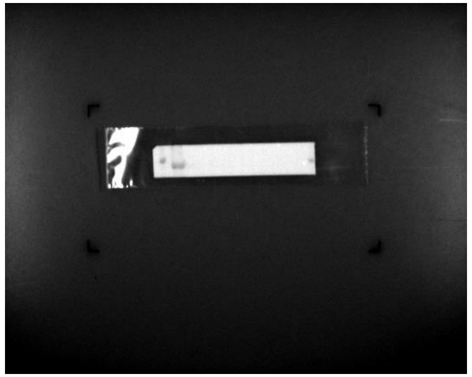

Full unedited blot for Supplemental Figure 5C

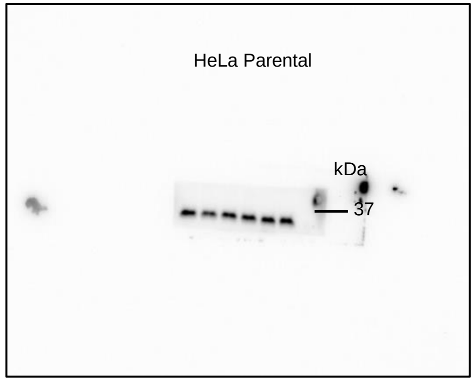

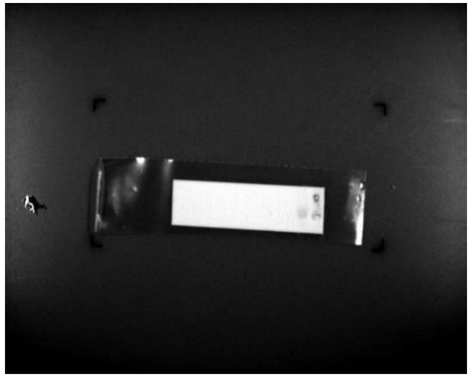

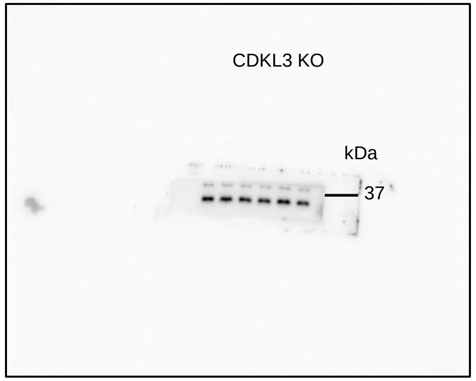

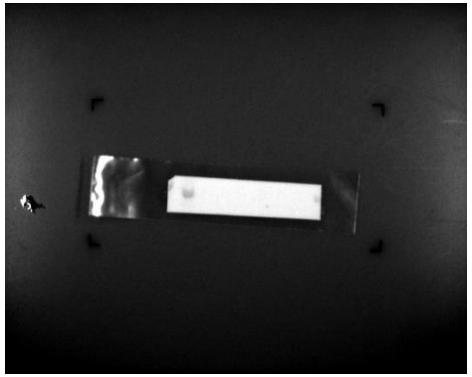

GAPDH

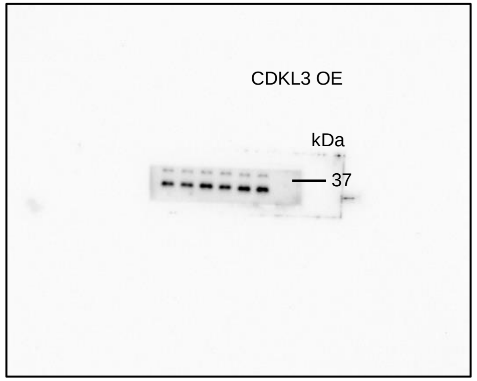

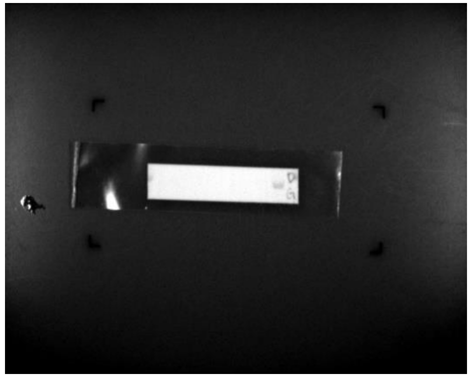

Full unedited blot for Supplemental Figure 5E

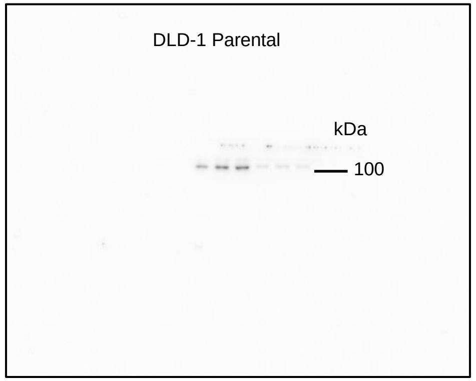

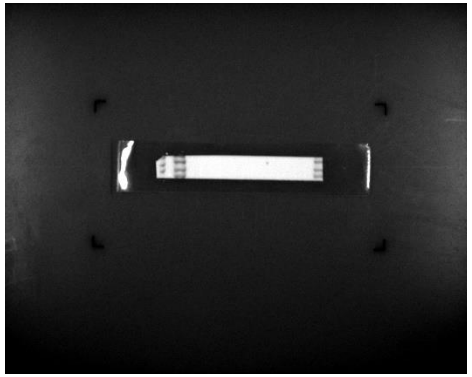

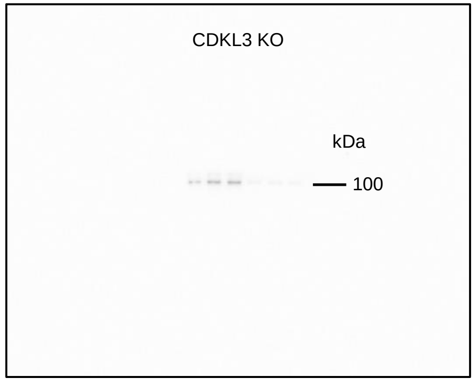

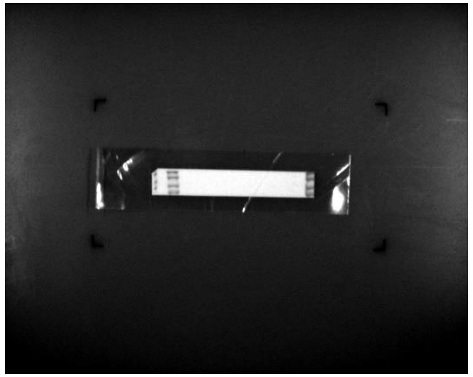

pRb

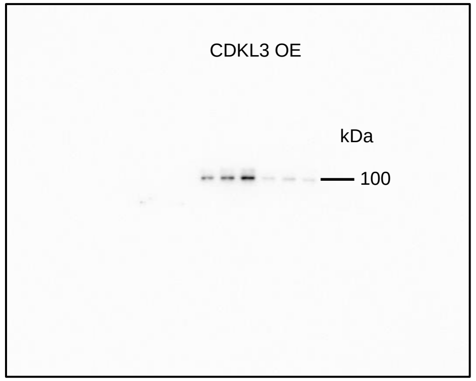

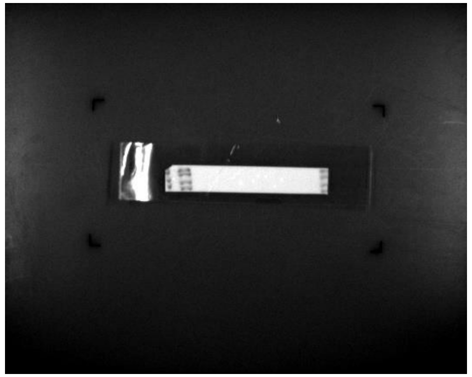

Full unedited blot for Supplemental Figure 5E

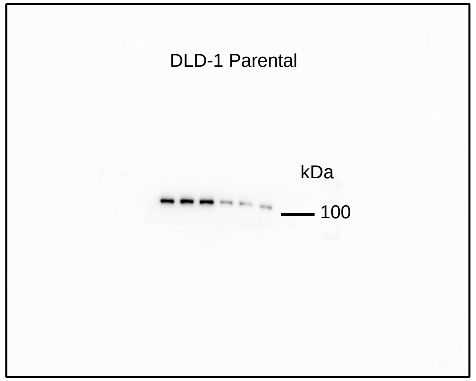

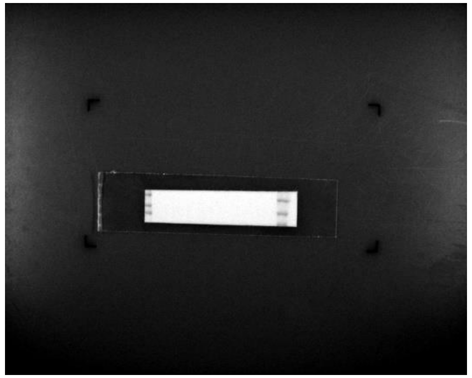

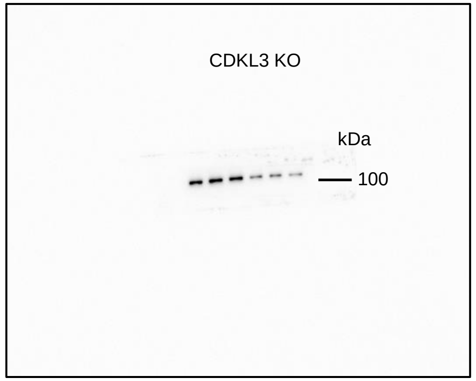

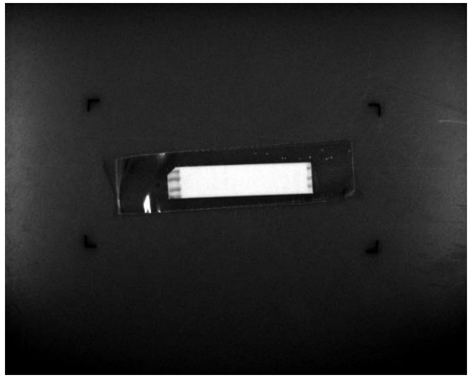

Rb

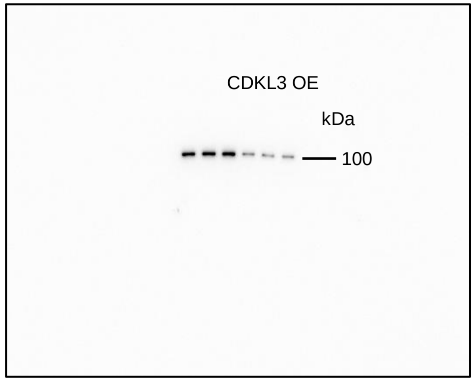

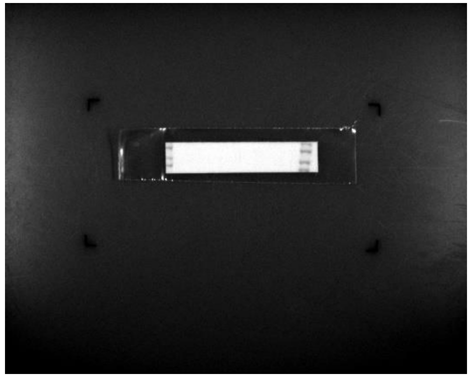

Full unedited blot for Supplemental Figure 5E

CDKL3

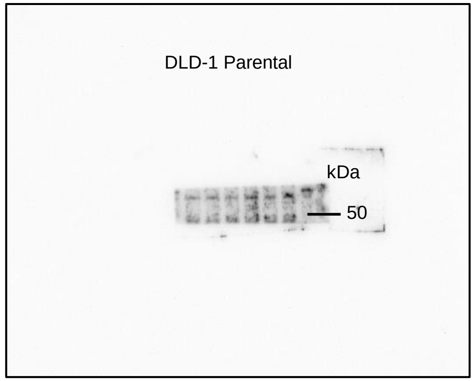

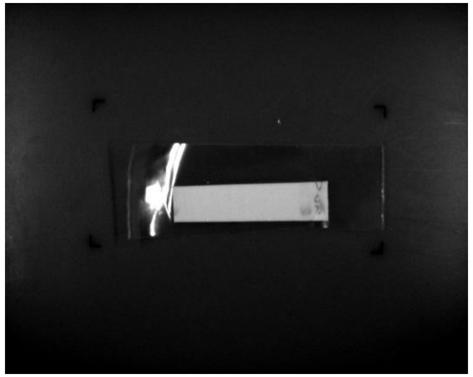

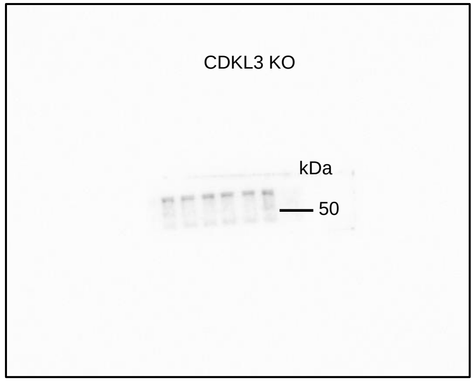

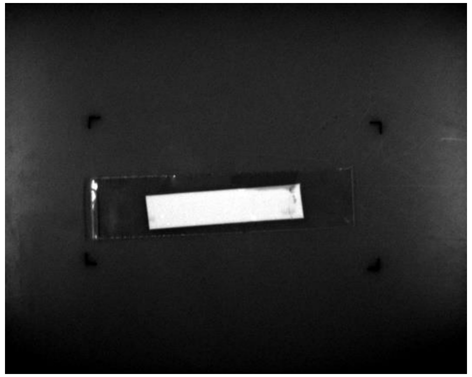

FLAG-
CDKL3

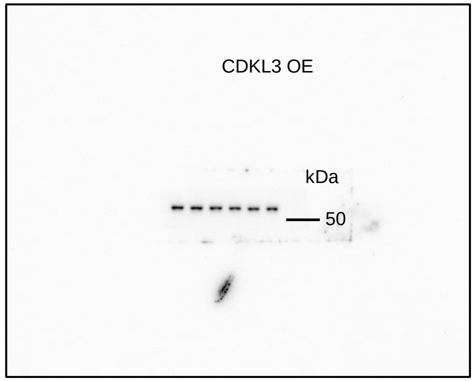

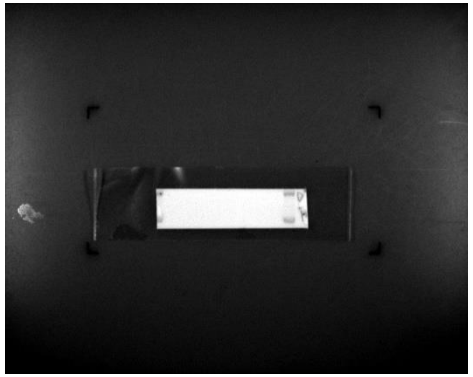

Full unedited blot for Supplemental Figure 5E

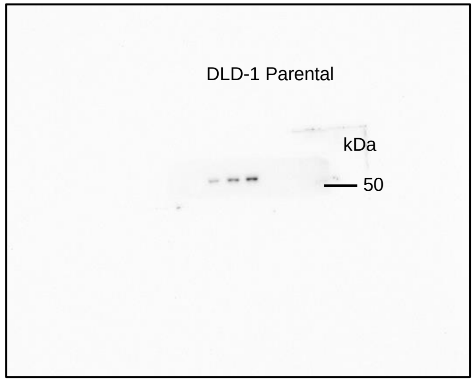

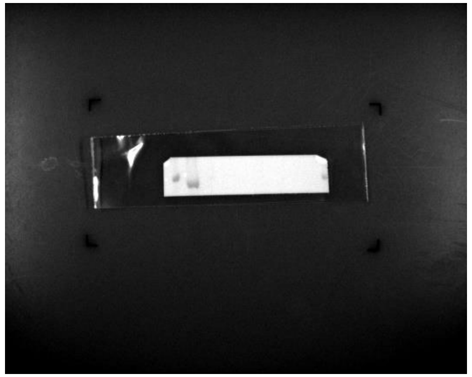

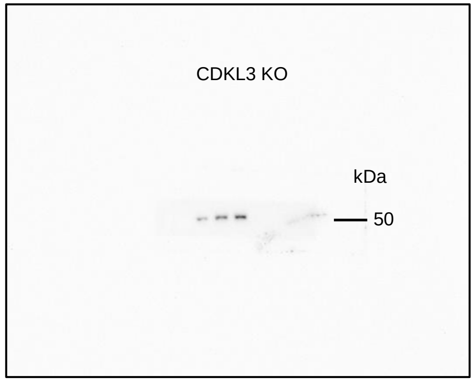

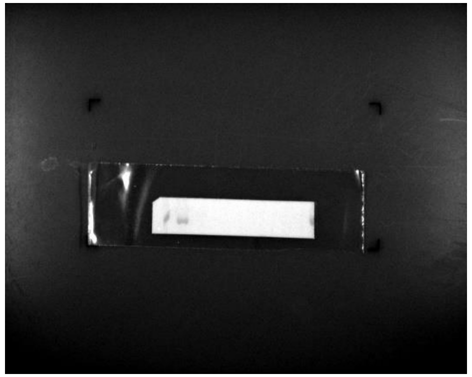

cyclinA2

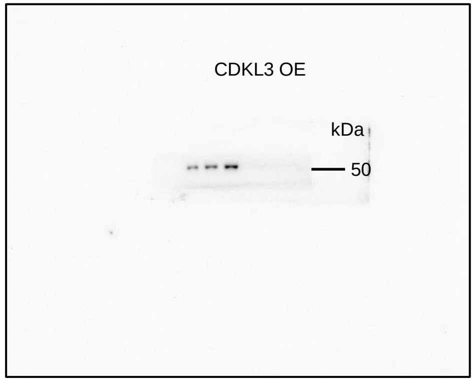

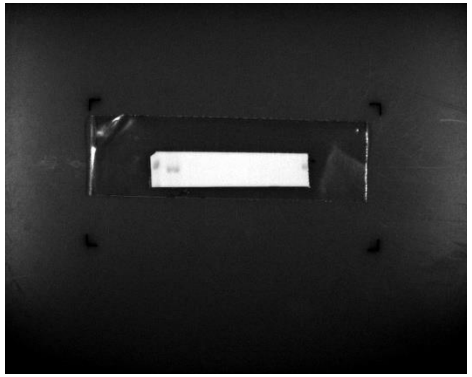

Full unedited blot for Supplemental Figure 5E

GAPDH

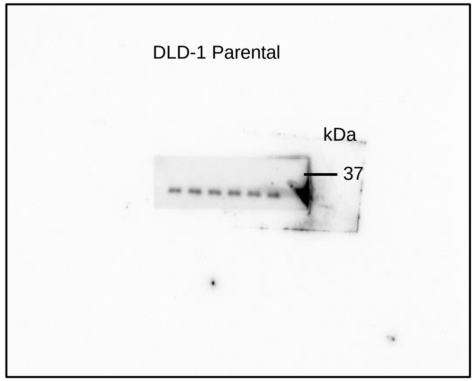

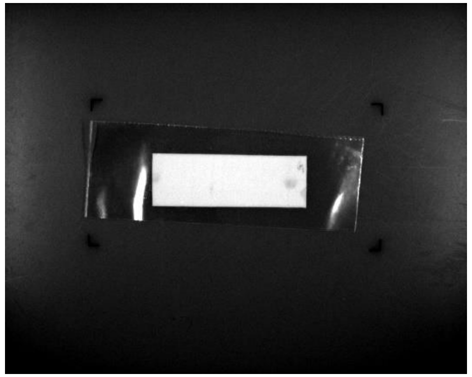

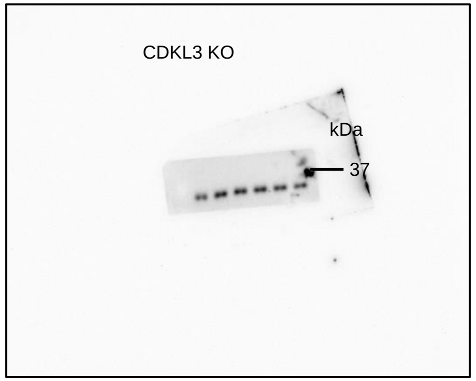

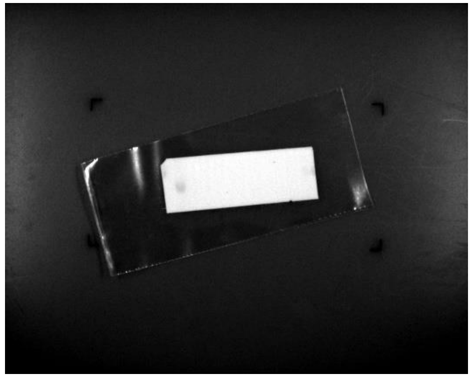

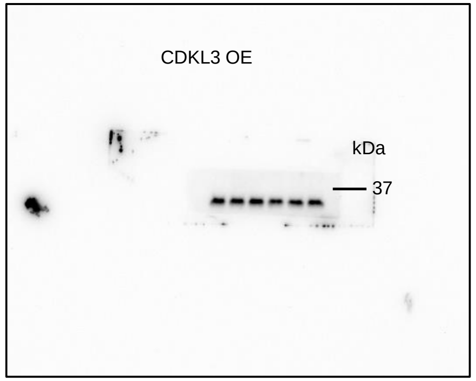

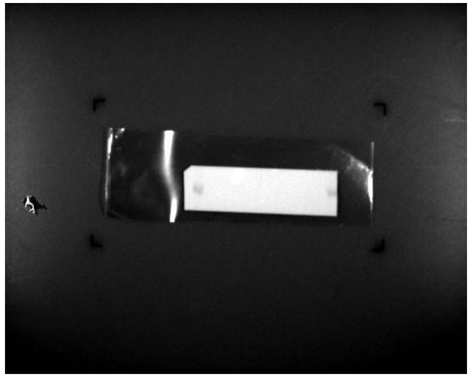

Full unedited blot for Supplemental Figure 5F

pRb

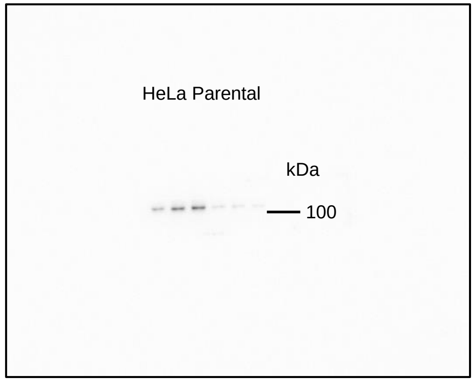

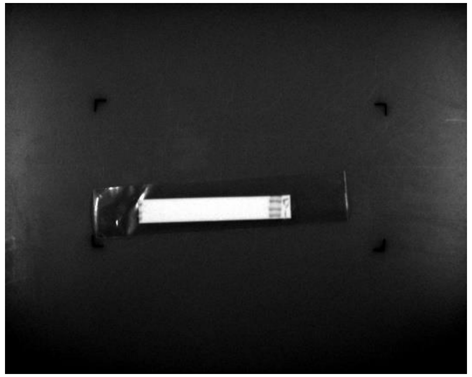

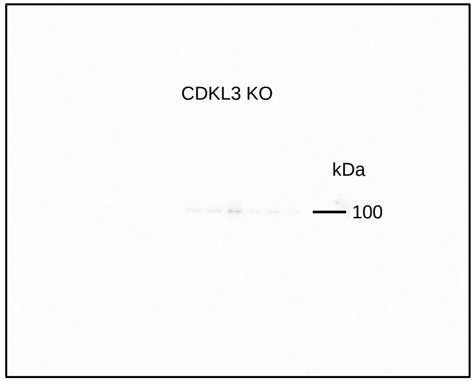

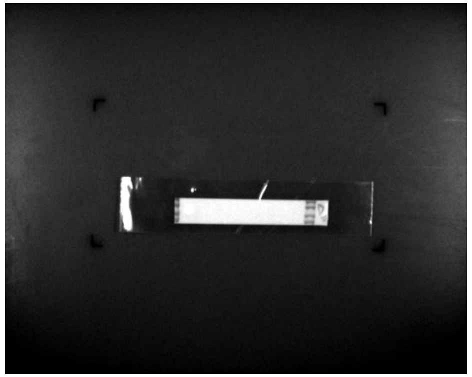

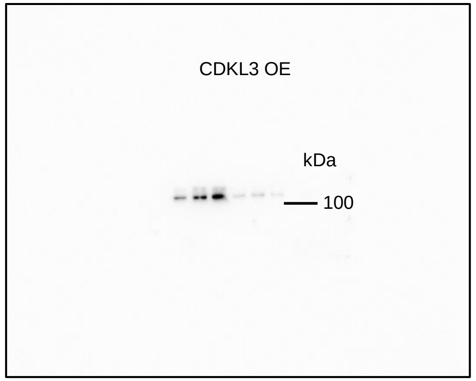

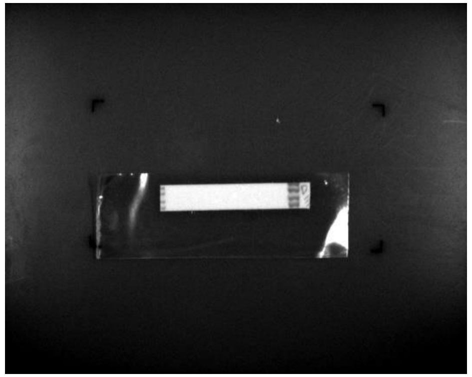

Full unedited blot for Supplemental Figure 5F

Rb

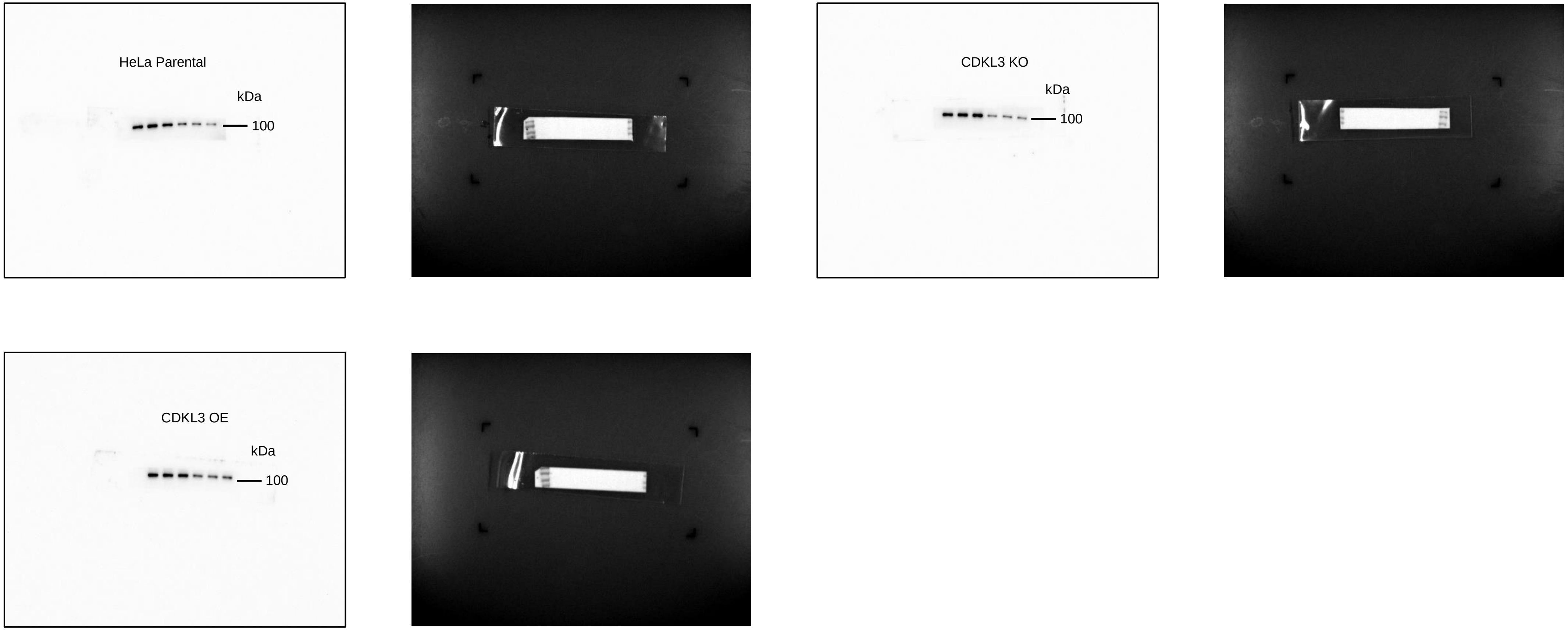

Full unedited blot for Supplemental Figure 5F

CDKL3

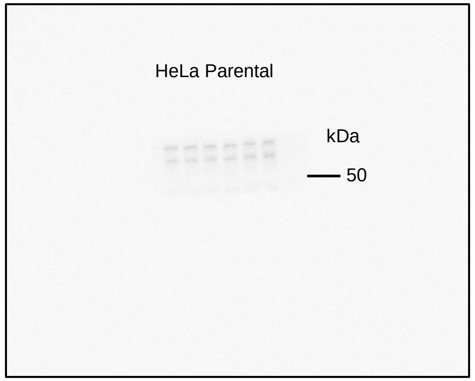

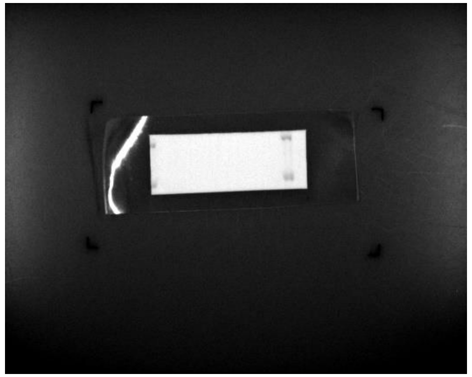

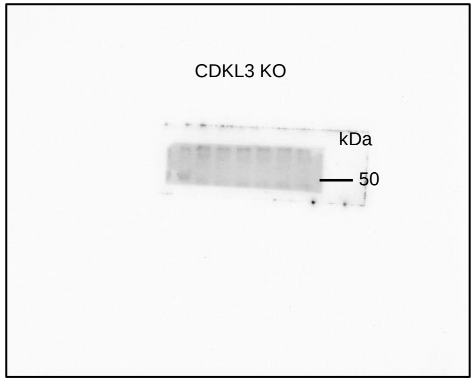

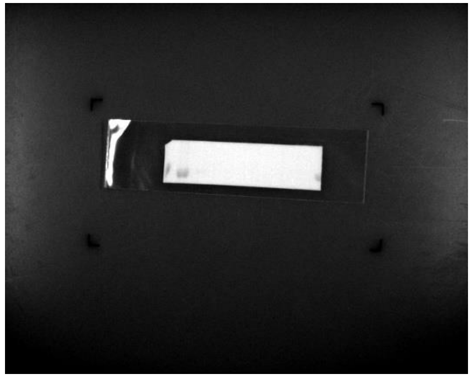

FLAG-CDKL3

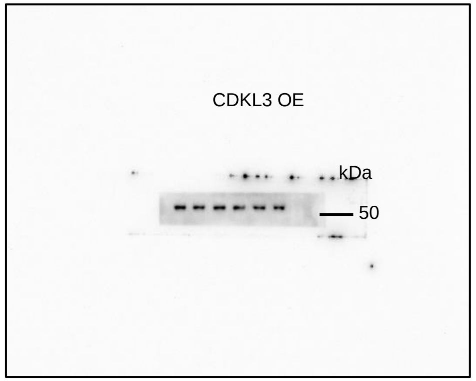

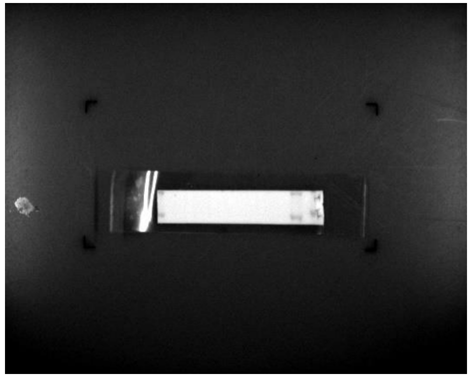

Full unedited blot for Supplemental Figure 5F

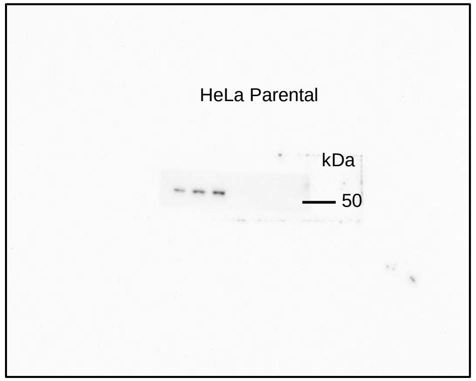

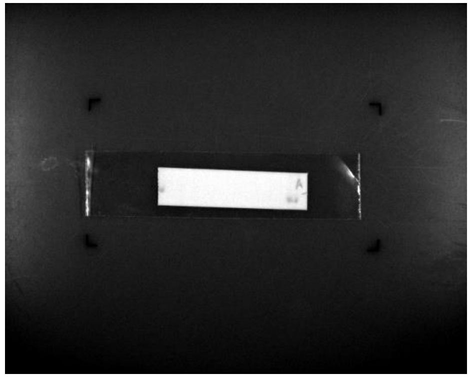

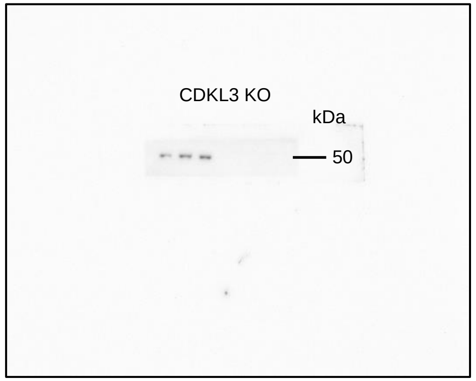

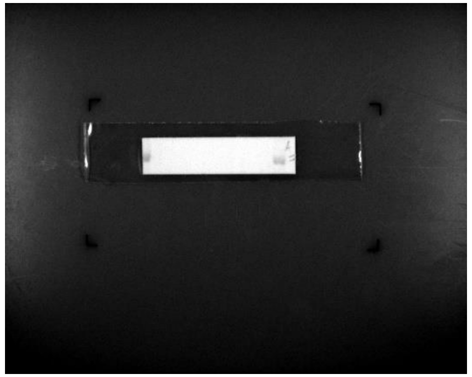

cyclinA2

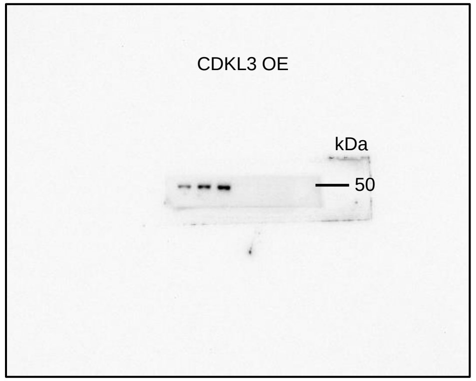

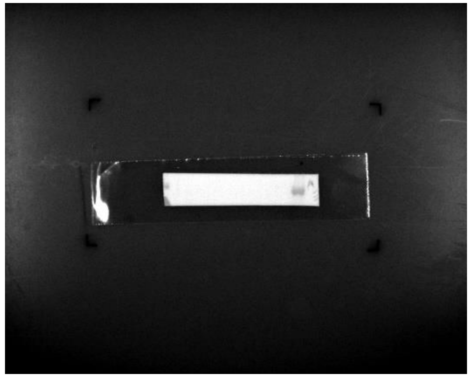

Full unedited blot for Supplemental Figure 5F

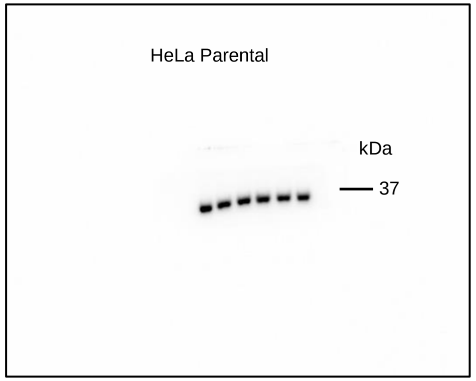

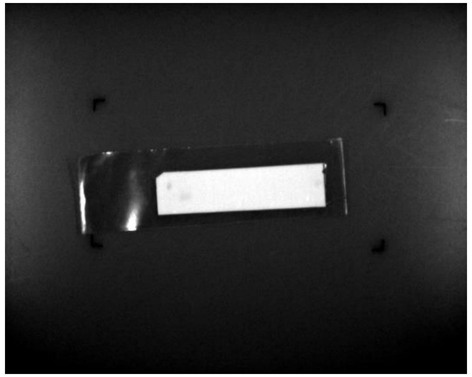

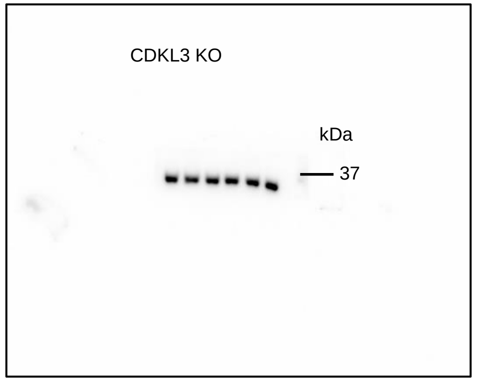

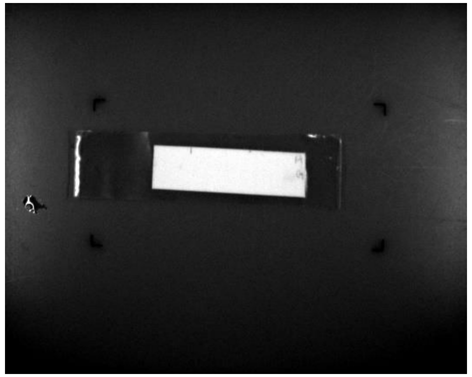

GAPDH

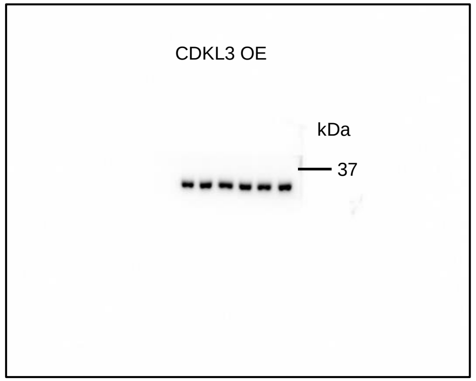

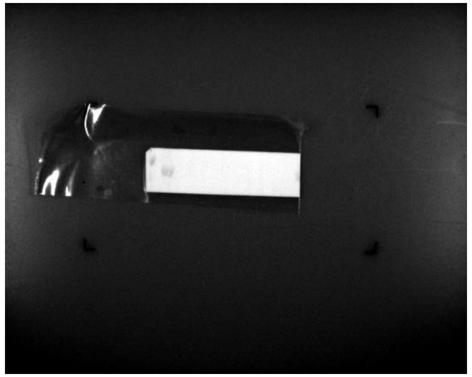

Full unedited blot for Supplemental Figure 6J

CDKL3

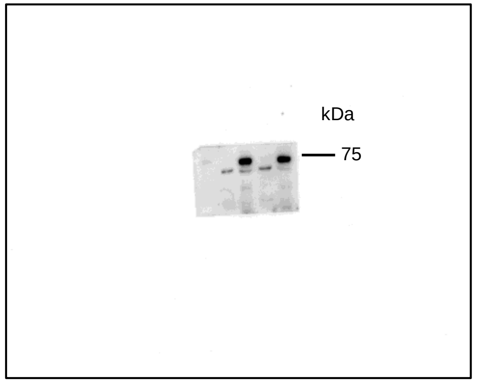

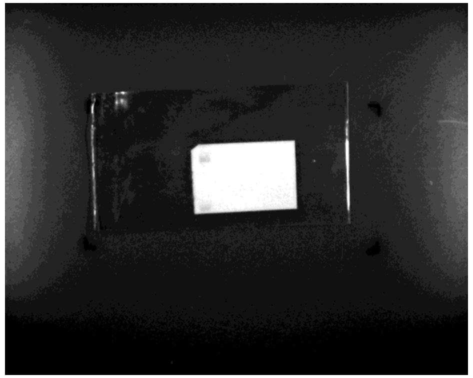

FLAG-
CDKL3

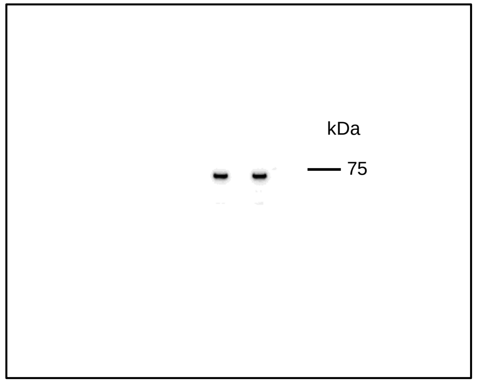

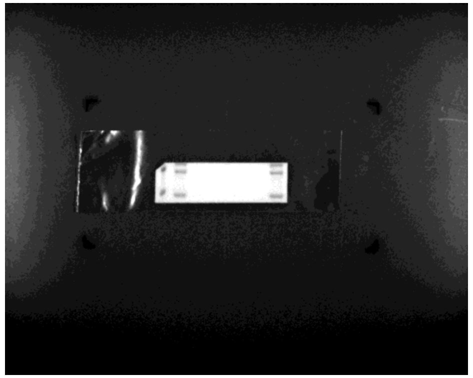

GAPDH

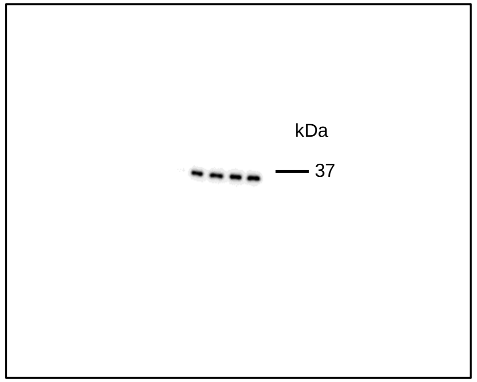

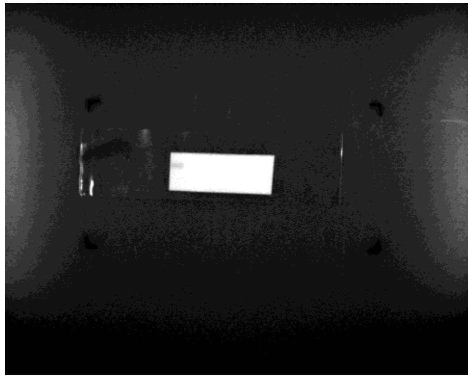

Full unedited blot for Supplemental Figure 6K

CDK4

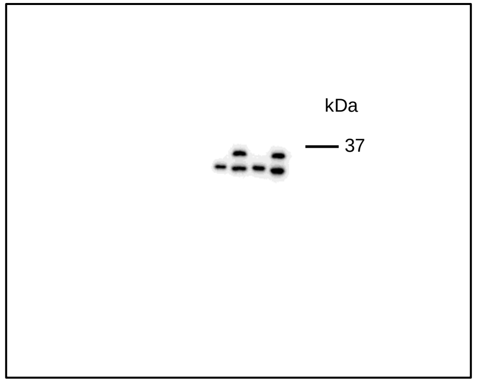

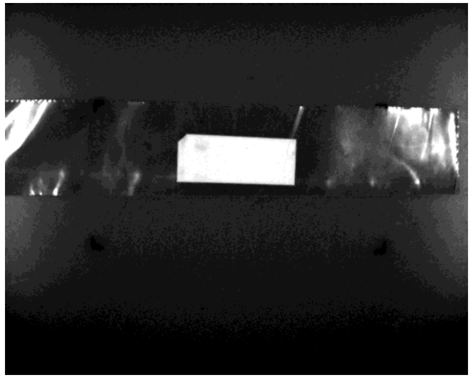

FLAG-
CDK4

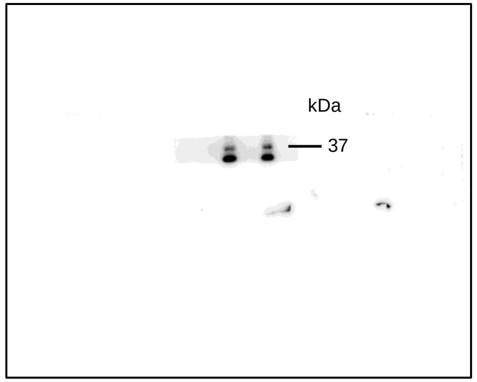

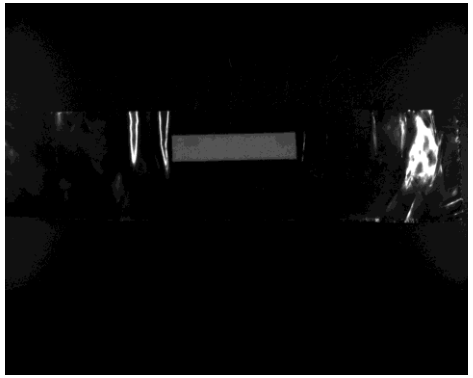

GAPDH

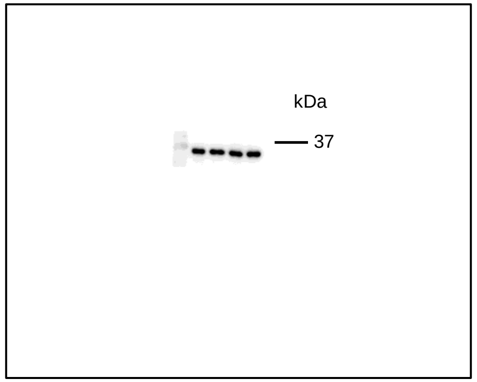

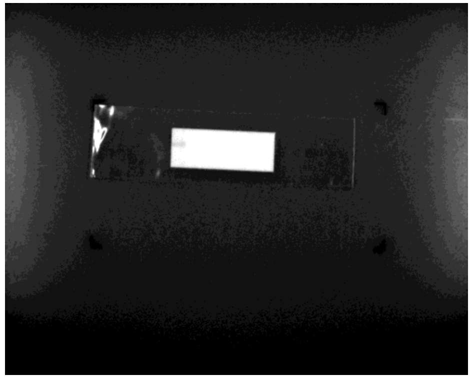

Full unedited blot for Supplemental Figure 6L

Rb

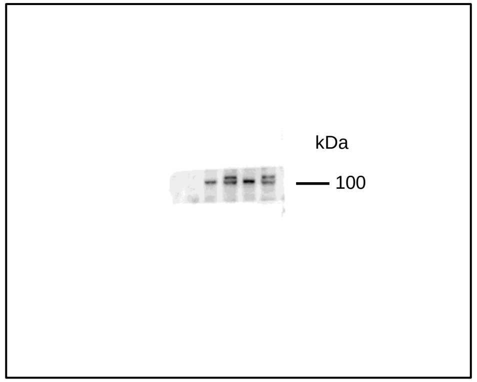

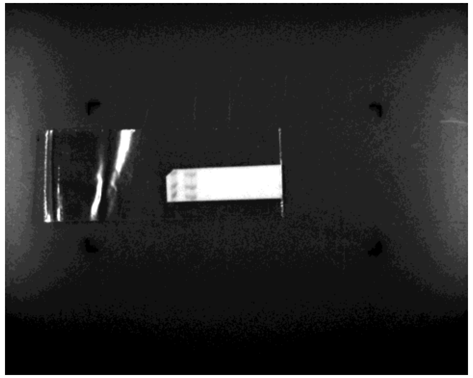

HA-Rb

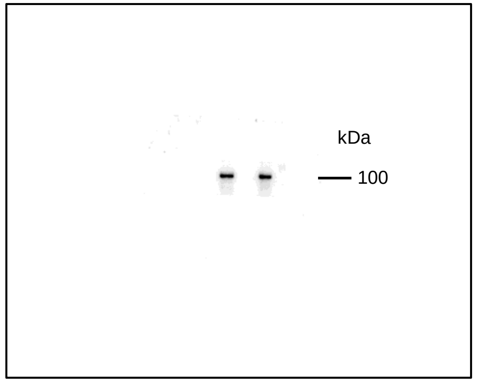

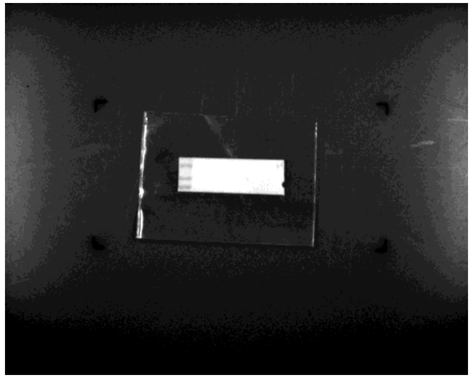

GAPDH

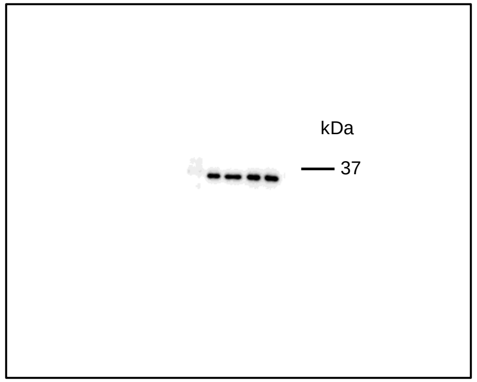

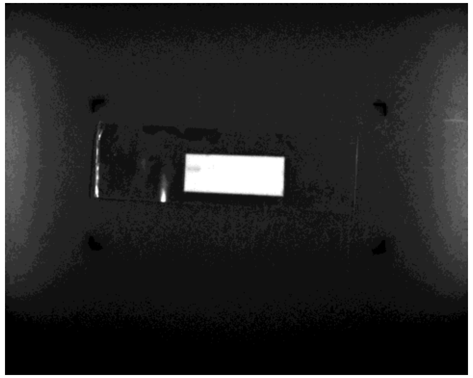

Full unedited blot for Supplemental Figure 6M

CDK4

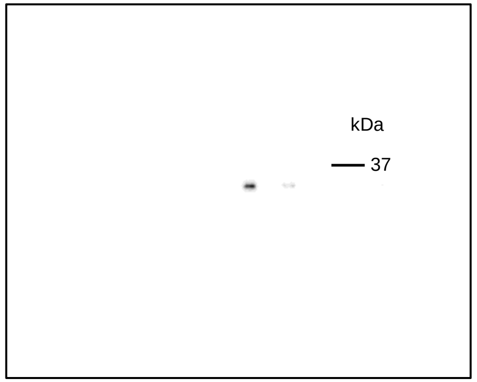

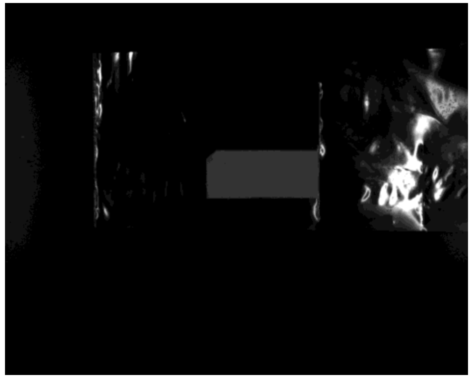

GAPDH

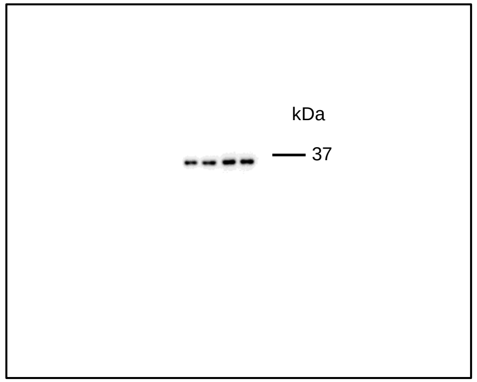

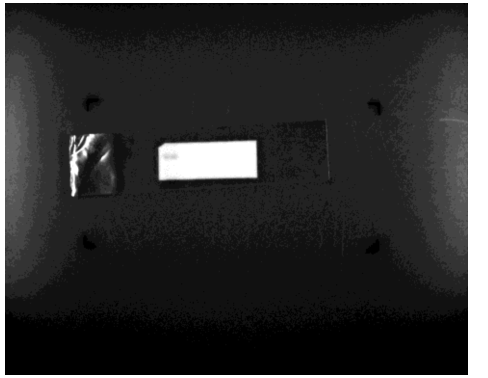

Full unedited blot for Supplemental Figure 6N

Rb

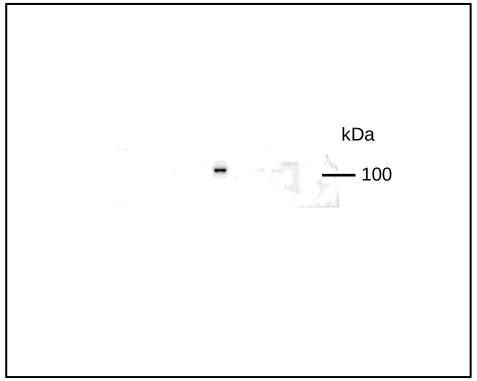

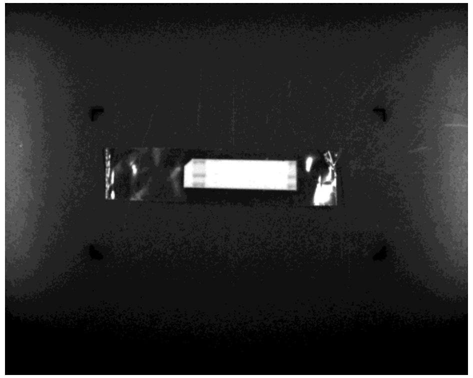

GAPDH

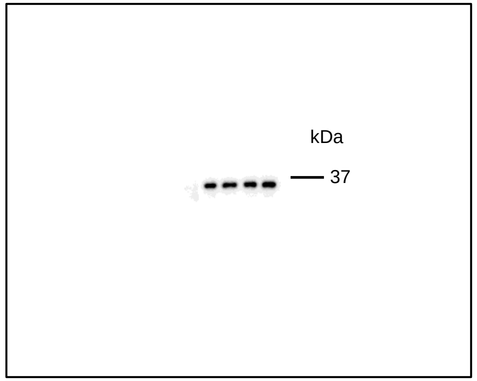

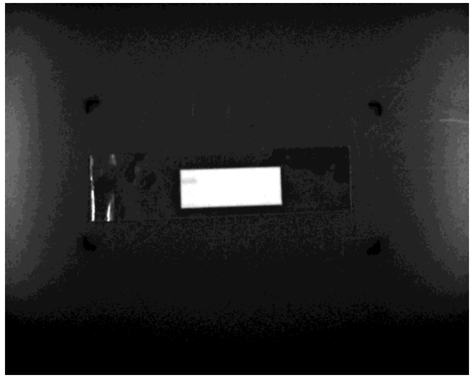

Full unedited blot for Supplemental Figure 6O

Rb

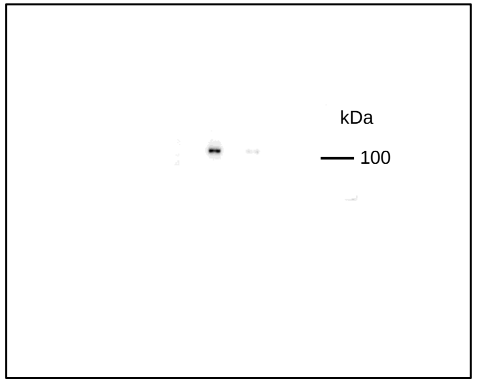

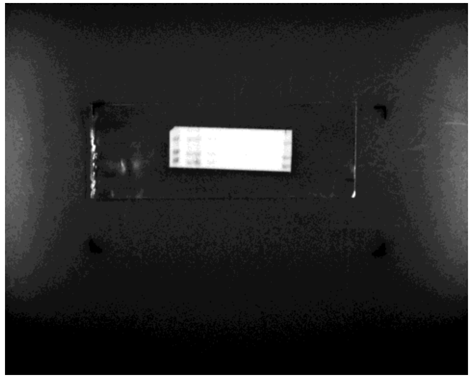

GAPDH

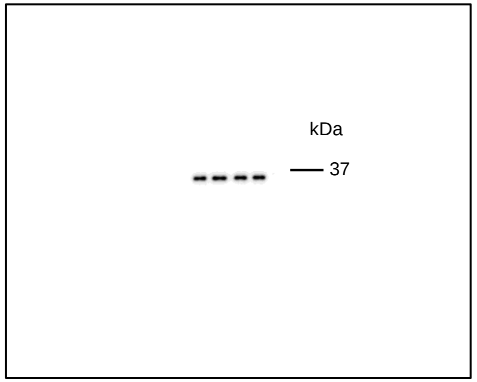

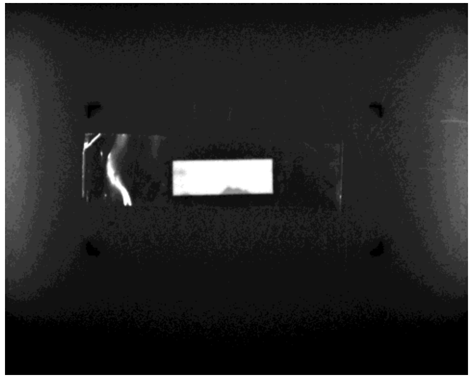

Full unedited blot for Supplemental Figure 7A

pRb

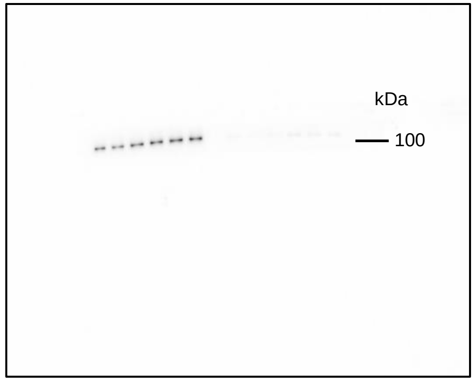

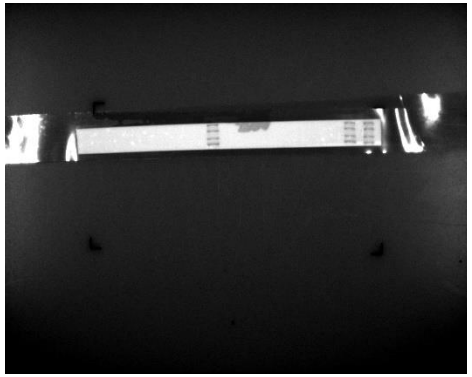

Rb

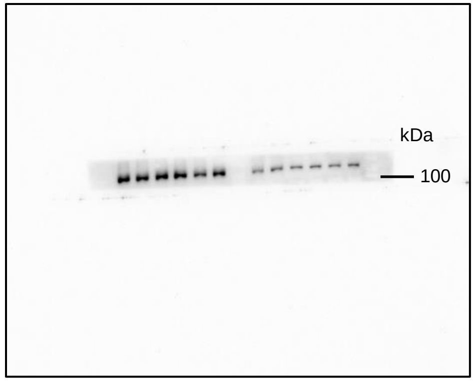

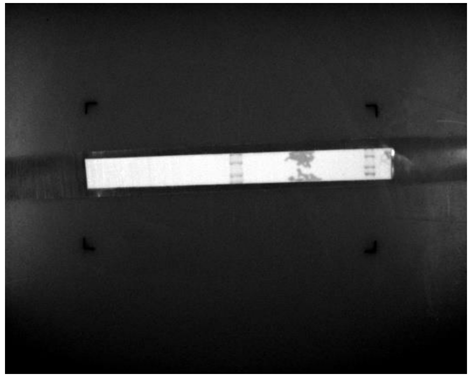

Full unedited blot for Supplemental Figure 7A

cyclinA2

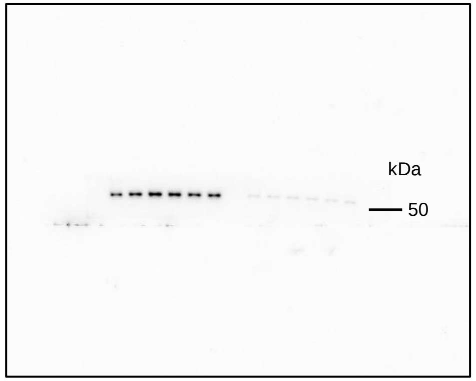

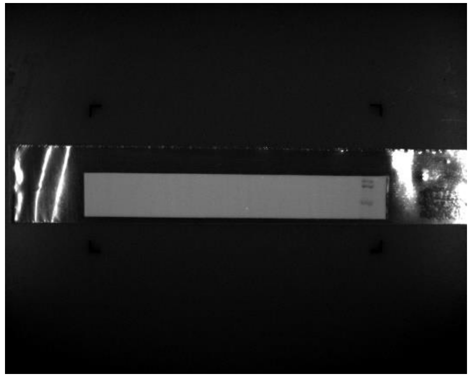

CDK4

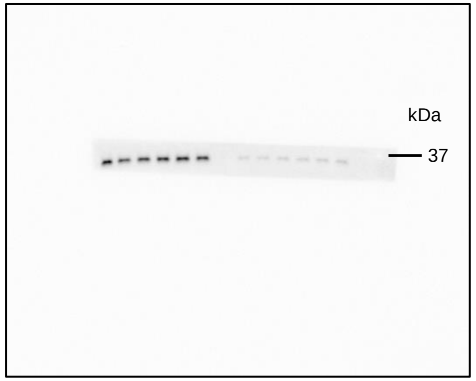

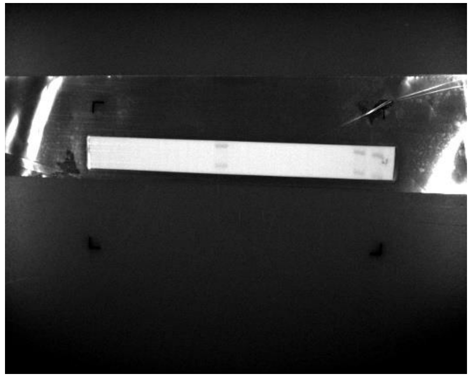

GAPDH

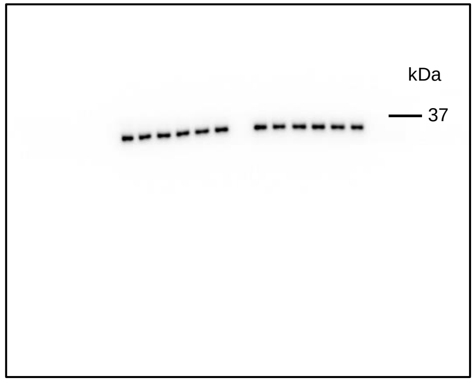

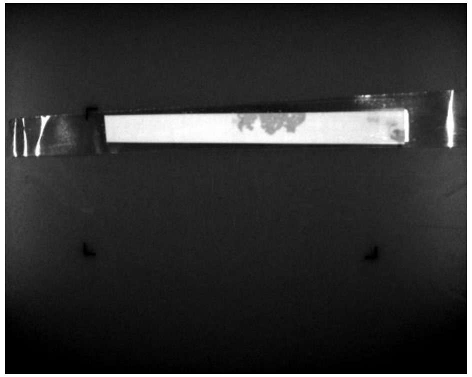

Full unedited blot for Supplemental Figure 7F

pRb

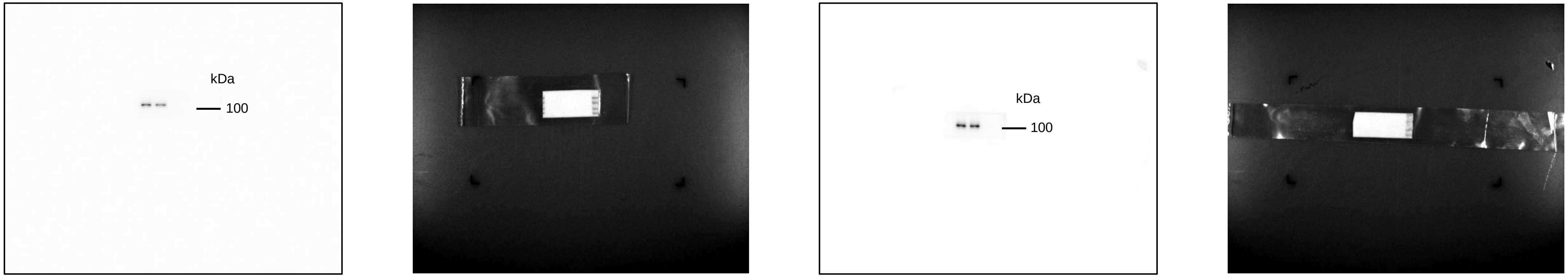

Rb

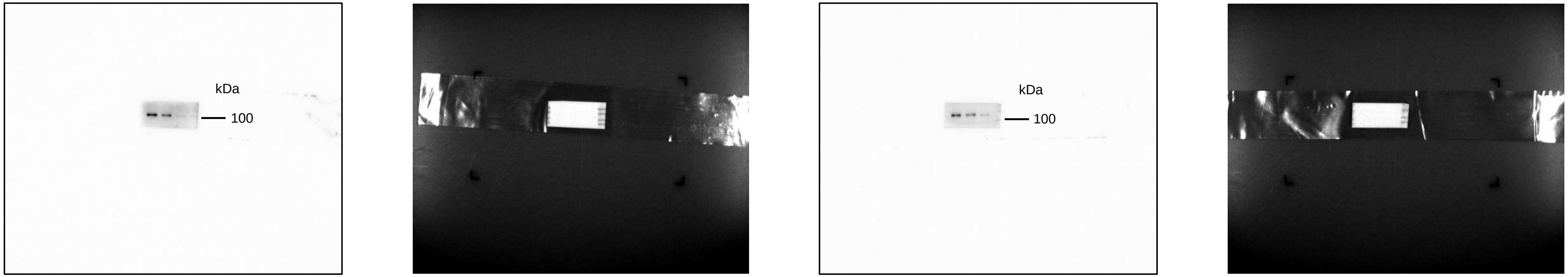

Full unedited blot for Supplemental Figure 7F

cyclinA2

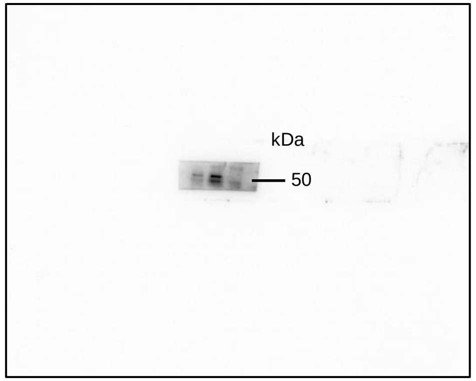

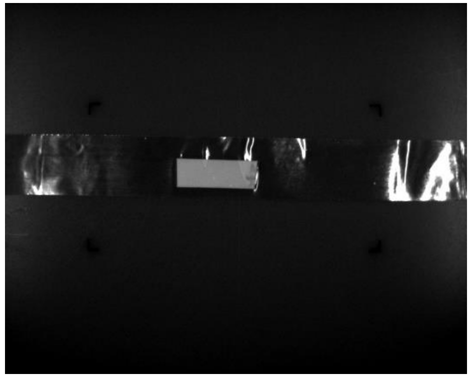

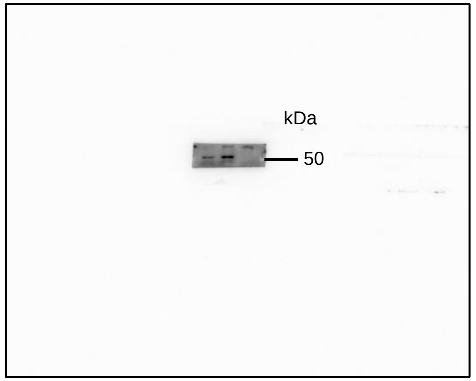

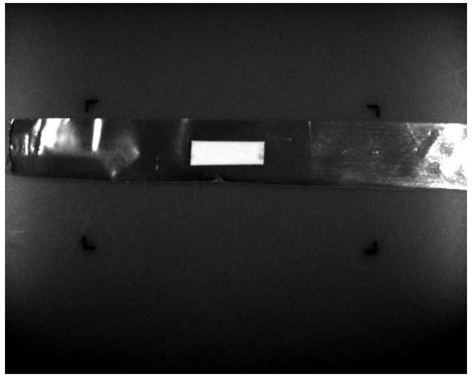

cyclinD1

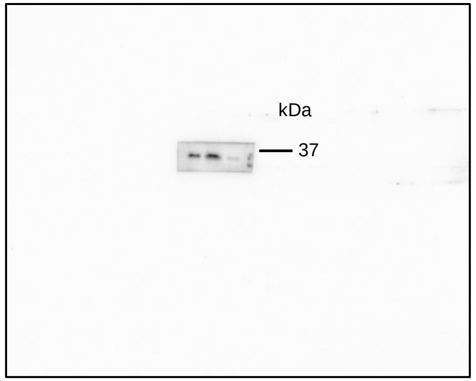

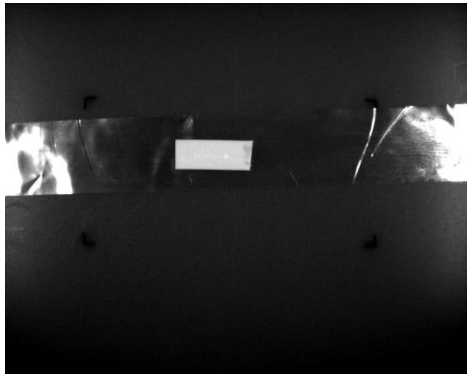

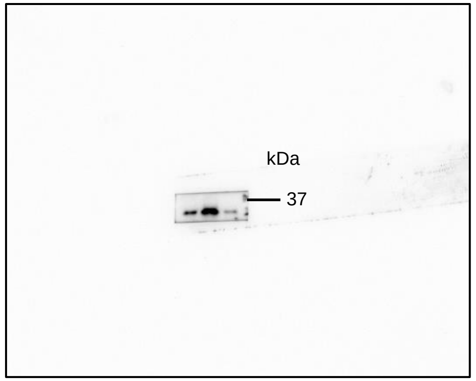

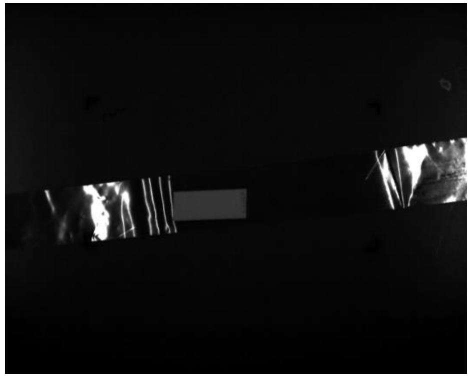

Full unedited blot for Supplemental Figure 7F

cyclinE1

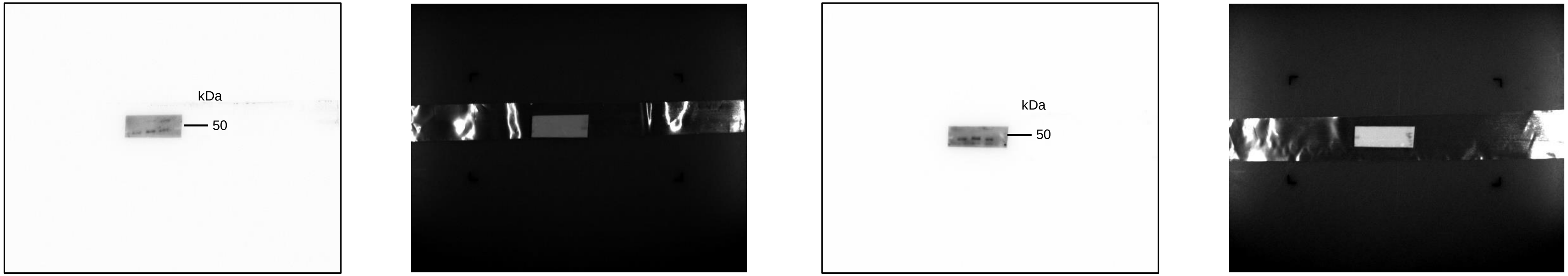

CDK4

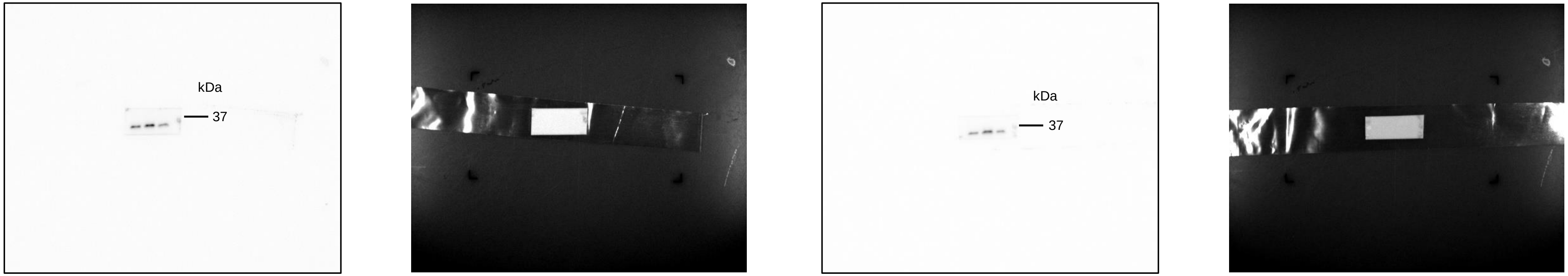

Full unedited blot for Supplemental Figure 7F

CDK6

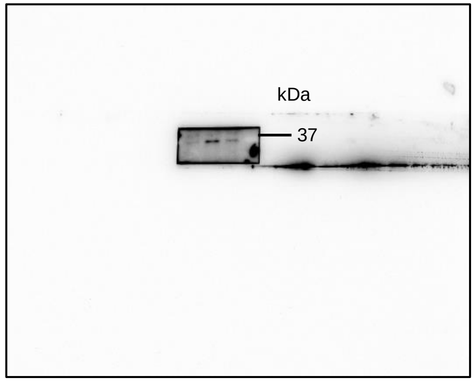

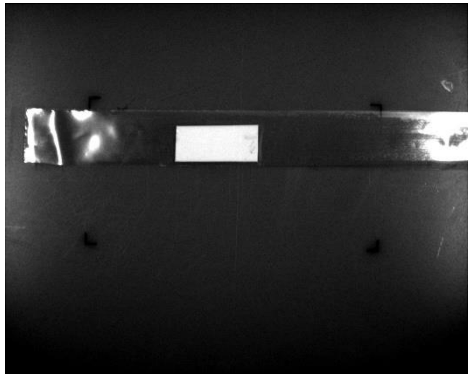

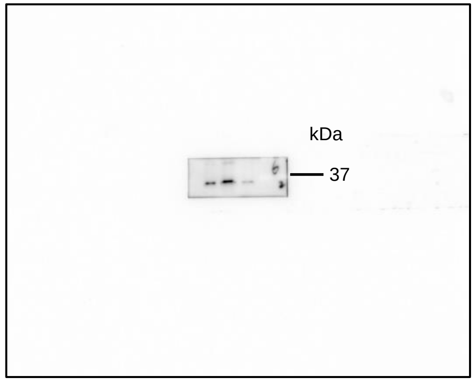

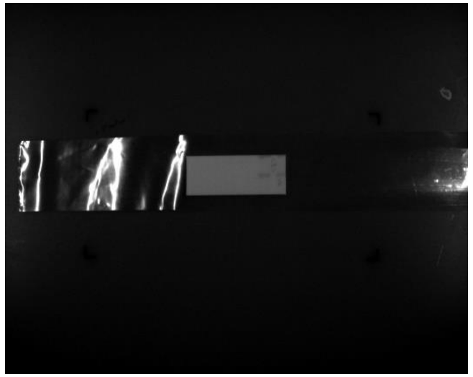

GAPDH

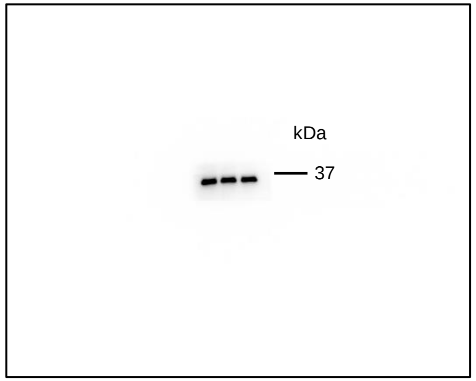

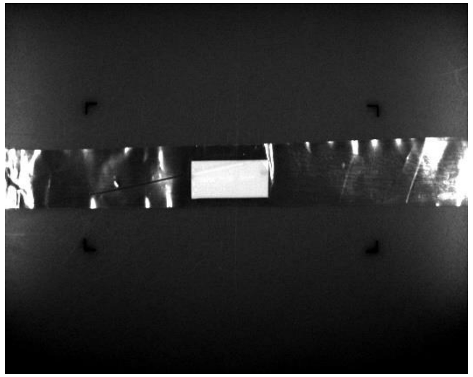

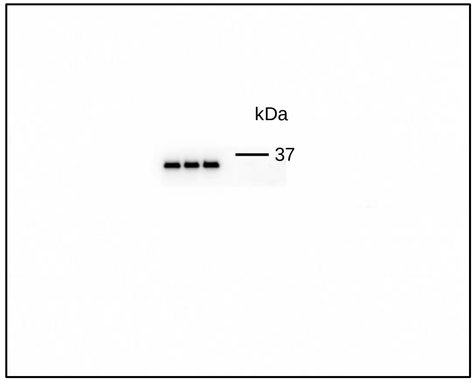

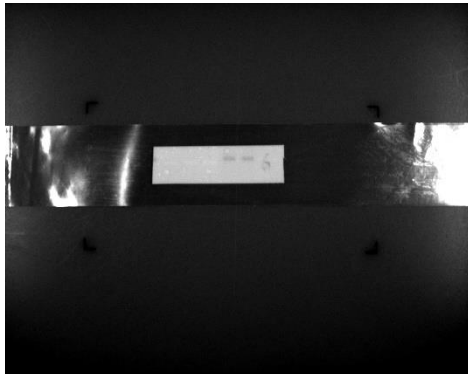

Full unedited blot for Supplemental Figure 7J

pRb

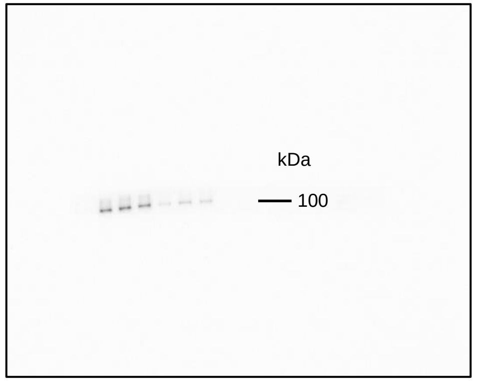

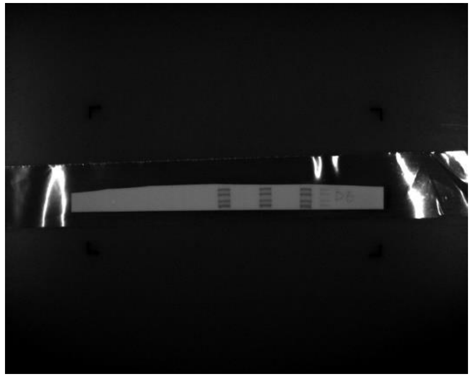

Rb

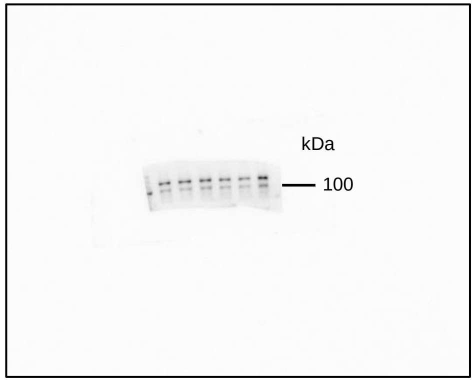

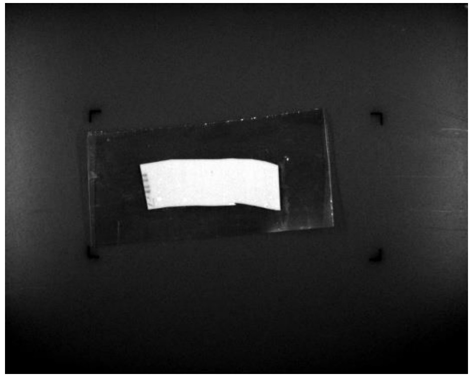

Full unedited blot for Supplemental Figure 7J

CDKL3

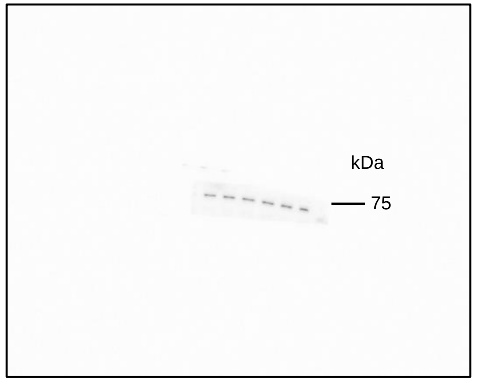

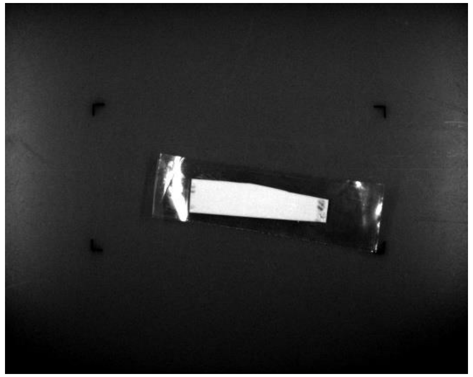

CDK4

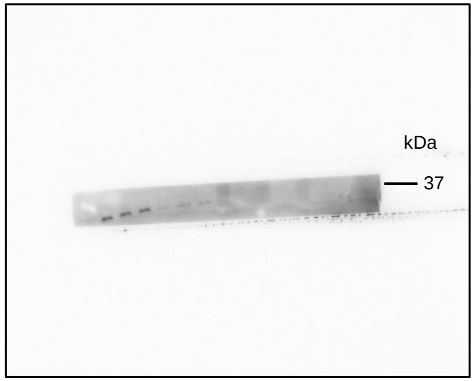

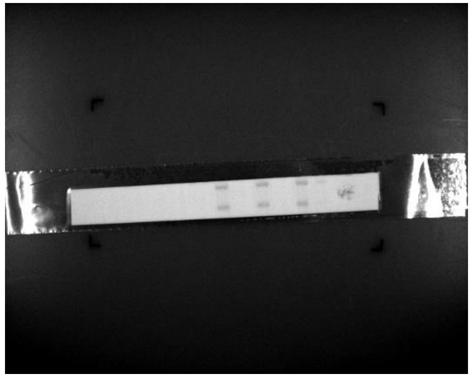

α-tubulin

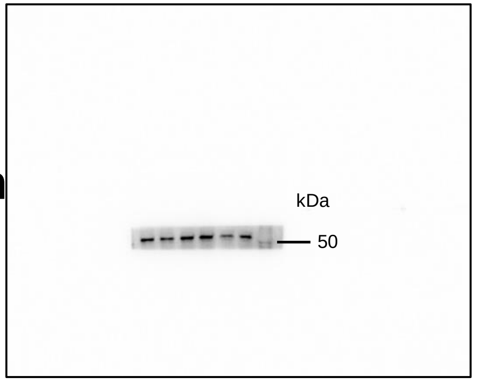

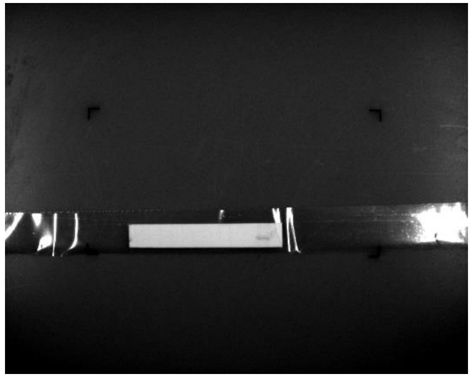

Full unedited blot for Supplemental Figure 8O

pRb

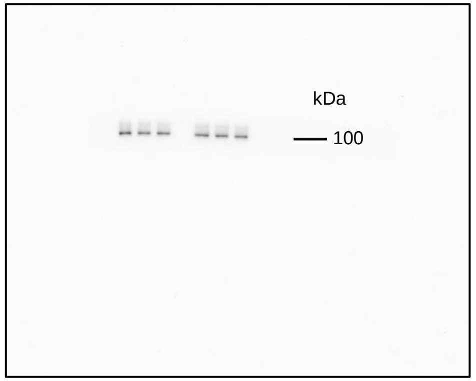

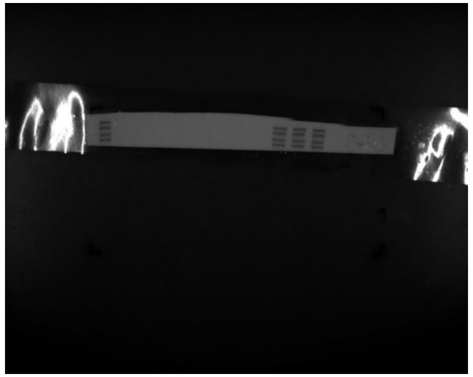

Rb

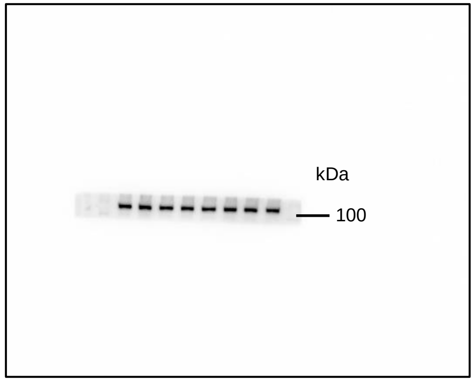

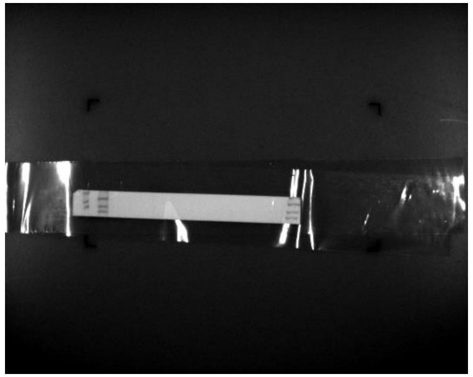

CDK4

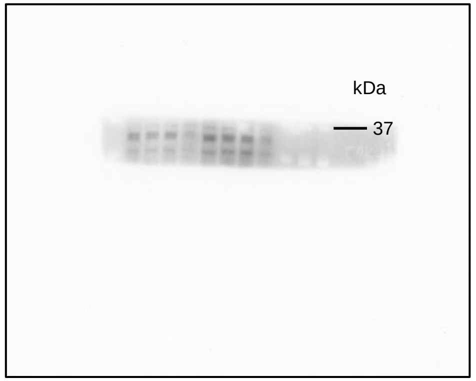

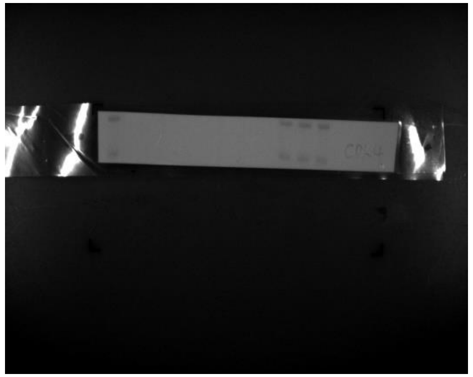

α-tubulin

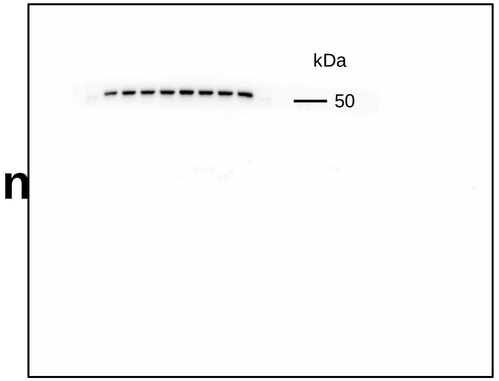

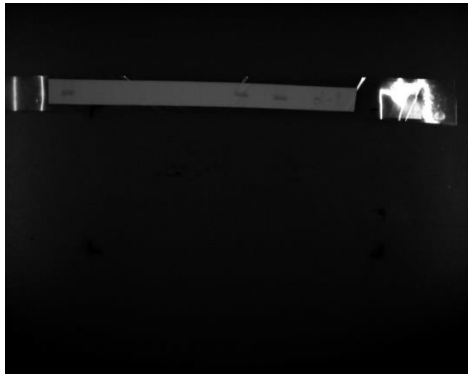

Full unedited blot for Supplemental Figure 8R

CDKL3

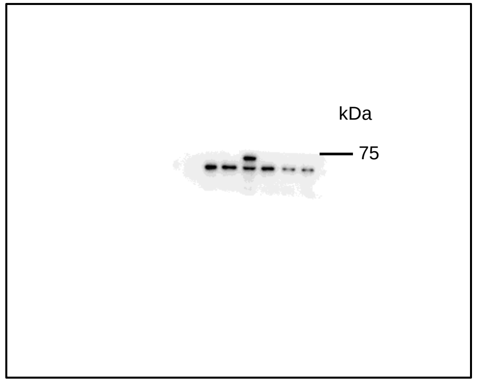

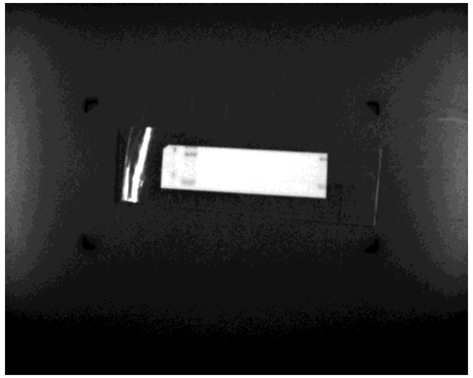

FLAG

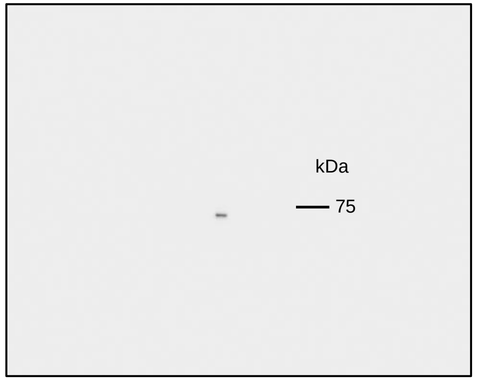

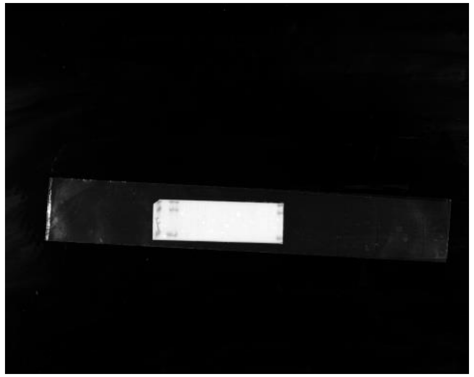

GAPDH

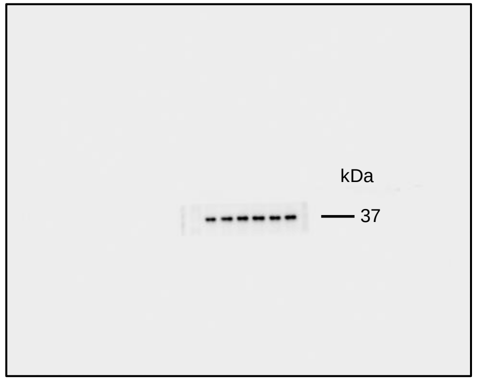

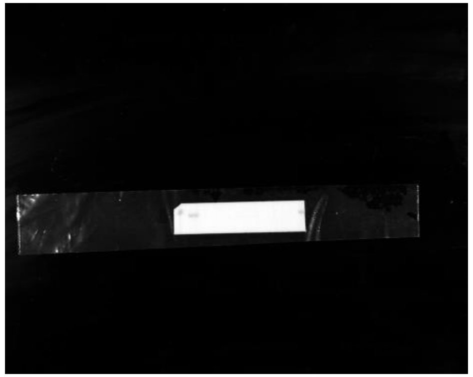

Full unedited blot for Supplemental Figure 8S

CDK4

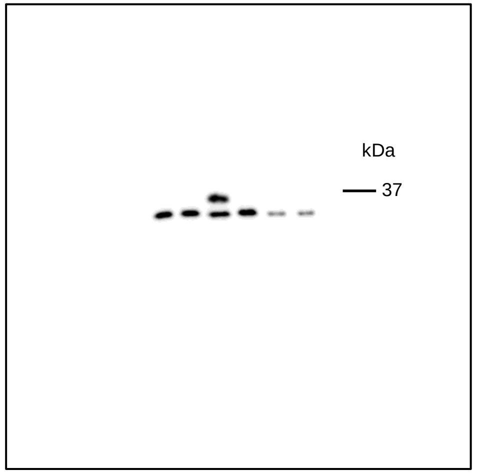

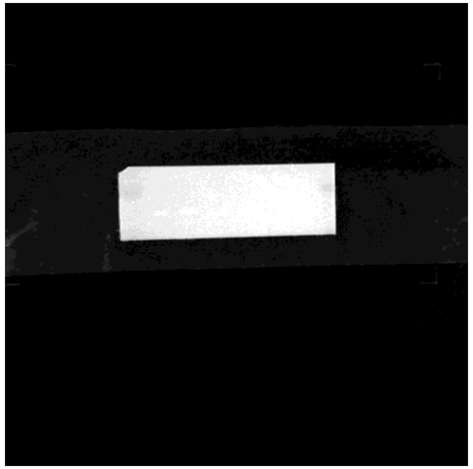

FLAG

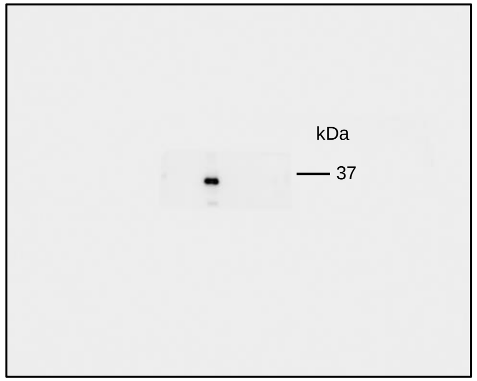

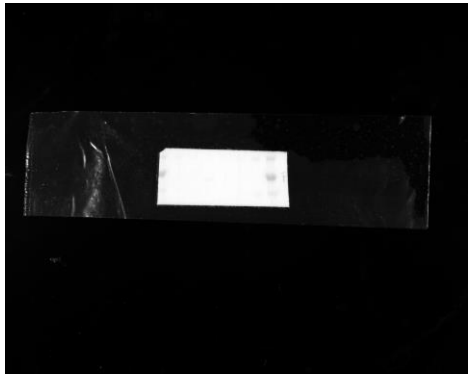

GAPDH

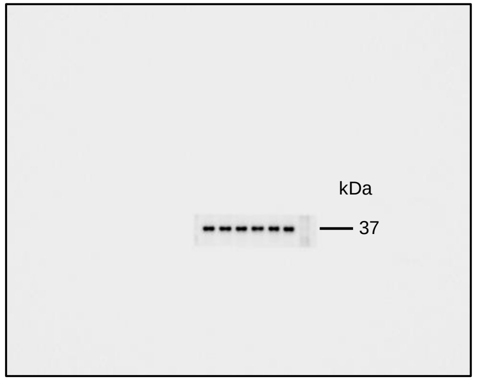

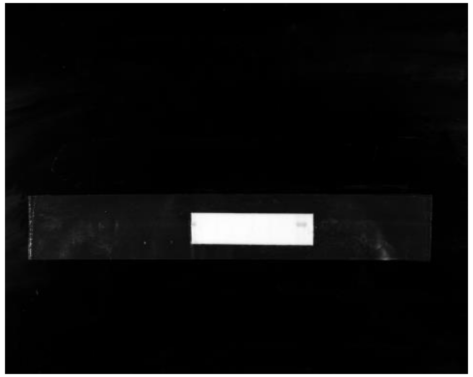

Full unedited blot for Supplemental Figure 8T

Rb

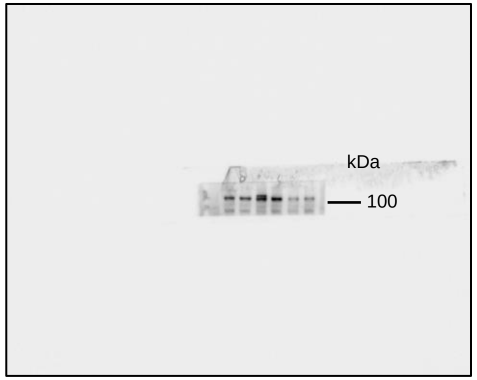

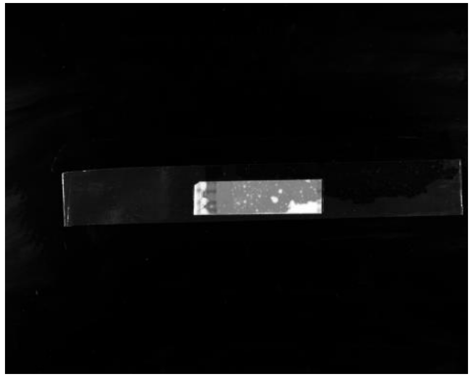

HA

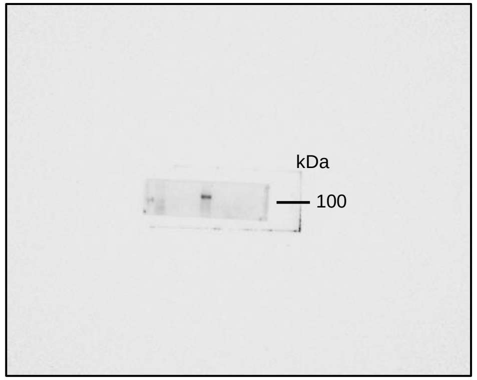

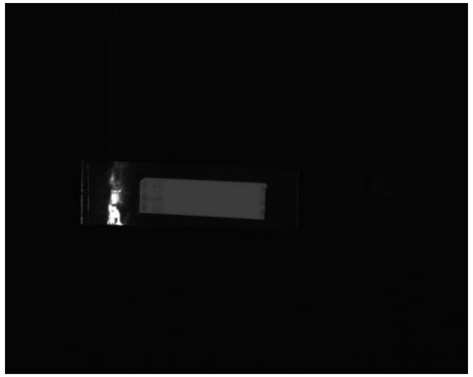

GAPDH

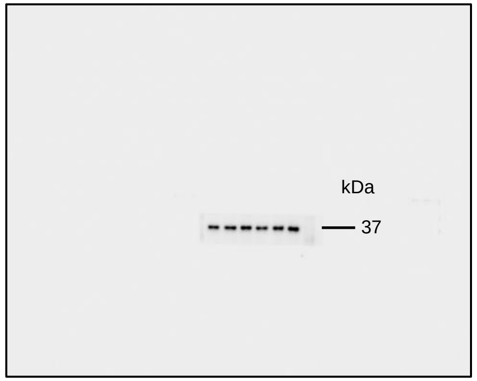

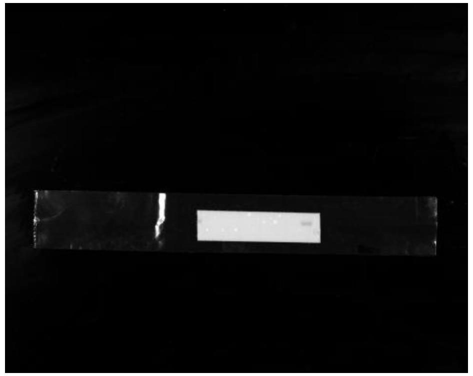

Supplement: Unedited blot and gel images [file jci-134-178428-s138.pdf]
